# Global estimates of the fitness advantage of SARS-CoV-2 variant Omicron

**DOI:** 10.1093/ve/veac089

**Published:** 2022-10-07

**Authors:** Christiaan van Dorp, Emma Goldberg, Ruian Ke, Nick Hengartner, Ethan Romero-Severson

**Affiliations:** Theoretical Biology and Biophysics (T-6), Los Alamos National Laboratory, P.O. Box 1663, MS P280, Los Alamos NM 87544, USA; Theoretical Biology and Biophysics (T-6), Los Alamos National Laboratory, P.O. Box 1663, MS P280, Los Alamos NM 87544, USA; Theoretical Biology and Biophysics (T-6), Los Alamos National Laboratory, P.O. Box 1663, MS P280, Los Alamos NM 87544, USA; Theoretical Biology and Biophysics (T-6), Los Alamos National Laboratory, P.O. Box 1663, MS P280, Los Alamos NM 87544, USA; Department of Pathology & Cell Biology, Columbia University Irving Medical Center, 630 West 168th Street, Mailbox 23 New York, NY 10032, USA

**Keywords:** SARS-CoV-2 variants, selection effects, mixed effects model, omicron variant

## Abstract

New variants of SARS-CoV-2 show remarkable heterogeneity in their relative fitness over both time and space. In this paper we extend the tools available for estimating the selection strength for new SARS-CoV-2 variants to a hierarchical, mixed-effects, renewal equation model. This formulation allows us to estimate selection effects at the global level while incorporating both measured and unmeasured heterogeneity among countries. Applying this model to the spread of Omicron in forty countries, we find evidence for very strong but very heterogeneous selection effects. To test whether this heterogeneity is explained by differences in the immune landscape, we considered several measures of vaccination rates and recent population-level infection as covariates, finding moderately strong, statistically significant effects. We also found a significant positive correlation between the selection advantage of Delta and Omicron at the country level, suggesting that other region-specific explanatory variables of fitness differences do exist. Our method is implemented in the Stan programming language, can be run on standard consumer-grade computing resources, and will be straightforward to apply to future variants.

## 1. Introduction

Since its emergence, the *Severe acute respiratory syndrome coronavirus 2 (SARS-CoV-2)* has continuously generated genetic variants that increase its transmission, e.g. D614G and variants of concerns such as Alpha and Delta, causing waves of coronavirus disease 2019 (COVID-19) surges across the globe (Korber et al., 2020; Davies et al., 2021; Volz et al., 2021; Dhar et al., 2021). Omicron, first detected in November 2021 in South Africa and Botswana, quickly spread globally, displaced Delta, and became the dominant variant in most countries. Initial studies on data from South Africa suggested that Omicron has a substantially higher transmission fitness than Delta, especially in individuals recovered from COVID-19 (Pulliam et al., 2022; Viana et al., 2022; Pearson et al., 2021). Experiments measuring plasma neutralization activity from vaccinated individuals demonstrated lower neutralizing antibody activities against Omicron even in fully vaccinated individuals (Schmidt et al., 2022; Collie et al., 2022; Cele et al., 2021; Lu et al., 2021; Planas et al., 2021); however, a booster shot increased neutralizing antibody activity substantially (Schmidt et al., 2022; Nemet et al., 2022; Planas et al., 2021). This suggests that Omicron is able to evade immunity induced in vaccinated individuals, providing a likely explanation for the rapid surge in Omicron in countries where a large proportion of individuals are fully vaccinated. Previous work has also quantified the reproductive number or relative growth advantage of Omicron in particular countries or settings, showing it to exceed that of previous emerging variants (Pearson et al., 2021; Weil et al., 2022) and to be greater among vaccinated individuals (Paton et al., 2022).

Although it is clear that Omicron has higher transmission fitness than Delta, we still lack a global perspective on the magnitude of this advantage and its heterogeneity among countries. Analyses of other SARS-CoV-2 variants have shown large differences in their relative fitness among different countries and US states (van Dorp et al., 2021; Figgins and Bedford, 2021). It is expected that the transmission advantage of Omicron is also heterogeneous across countries, due to the difference in circulating variants in each country, the different immunological landscapes arising from natural infection and vaccination, the different non-pharmaceutical intervention strategies adopted in different countries, and potentially many other factors. Unknown, however, is the extent to which among-country heterogeneity is actually explained by any identified factors.

To quantify the transmission advantage of Omicron across many countries and explore possible causes of its heterogeneity, we analyzed case count and variant frequency data with a renewal model (as Figgins and Bedford, 2021; Park et al., 2022a) that is hierarchical across countries (as Paton et al., 2022; van Dorp et al., 2021) and includes country-level covariates (as Paton et al., 2022), while allowing for a shorter generation time of Omicron (which could otherwise bias the results, cf. Pearson et al., 2021). We found that across forty countries, a portion of the heterogeneity in Omicron’s fitness could be explained by immunological-related covariates. Additionally, we found some correlation across countries between the selective advantages of Omicron and Delta, suggesting that fixed country-level effects may exist. More generally, we demonstrate the efficacy of a hierarchical model structure in quantifying the overall variant fitness.

## 2. Methods

### 2.1 Data

Variant counts per country per day were obtained from the Global Initiative on Sharing Avian Influenza Data (GISAID) metadata (Elbe and Buckland-Merrett, 2017; Global Initiative on Sharing All Influenza Data, 2008), downloaded on 11 March 2022. The known problematic samples were removed according to the Nextstrain ‘ncov’ quality control pipeline (Hadfield et al., 2018; Nextstrain, 2022). For each analysis we chose one focal variant—Delta or Omicron, as assigned in the GISAID metadata—and grouped all other variants into an ‘other’ category.

Many countries showed a pattern in which a few early cases of a focal variant were followed by many days without it, before an eventual strong rise in variant frequency. Early stochastic dynamics like this are expected, but they lead to poor fits of our deterministic model. (In particular, the logistic shape of the variant’s frequency becomes too shallow, leading to underestimates of the strength of selection.) To avoid this left tail in the data, we began the time series for each focal variant in each country on the first day at which three criteria were met: a variant frequency on that day of at least 10 per cent, at least 10 cumulative variant cases, and at least 0.05 per cent cumulative of the eventual number of variant cases. For Omicron, start dates were all in November and December 2021. For Delta, start dates ranged from January to July 2021. For each country, we ended the time series 9 weeks after it began for Omicron and 15 weeks after it began for Delta; the Delta time series was longer because its rise was slower than Omicron’s. (The model is not sensitive to the end date, provided the focal variant has not yet been replaced by another and the whole population has not yet been infected.) We further required that a country has at least 21 days with any sequence data and at least 1,000 sequences of the focal variant and of any other variants. This yielded forty countries for Omicron and fifty-six countries for Delta.

To use the prevalence of Delta as a covariate for Omicron’s relative fitness, we computed for each country the proportion of variant cases that were Delta in the week prior to Omicron’s arrival. We also considered the vaccination status and two proxies for country-level immunological status: excess deaths and number of cases occurring before the first day of the study period for each country. Vaccination data were obtained from Our World in Data (OWID) (Ritchie et al., 2020), downloaded on 6 January 2022. The percentage of fully vaccinated people (‘fullvax’ covariate) was defined as the number of fully vaccinated people from OWD divided by the 2020 population size multiplied by 100, and the percentage of boosted people (‘boostvax’ covariate) was defined as the number of booster shots administered divided by the population size in 2020 multiplied by 100. The excess deaths data were also obtained from OWD (Ritchie et al., 2020) and were normalized by the population size in 2020 multiplied by 100 (‘death’ covariate). The ‘cases’ covariate was computed as the sum of reported cases in the period before the first day of the study period for each country. For the immunological status proxy variables, we consider both a short (2 weeks) and long period (25 weeks) preceding the first day of the study period. Distributions of covariates across countries are shown in Fig. S1.

### 2.2 Model

Previously (van Dorp et al., 2021), we used a hierarchical logistic regression model for the increase in frequency of a new variant over time. As we noted there, this model may be sensitive to spatial and temporal variation in the effective reproduction number because the region-specific effects represent the difference in growth rates of the two competing variants. This means that when the incidence is growing (effective reproduction number $R_e \gt 1$), we would estimate a larger selective advantage than in an otherwise identical region where the incidence is decreasing ($R_e \lt 1$). The effective reproduction number within a region can also vary significantly over time, for instance due to implemented non-pharmaceutical interventions in anticipation of a recently discovered variant. To account for these confounding effects, we make use of regional case count data *C*_*t*_ to estimate the time-varying effective reproduction number $R^{\mathrm{wt}}_t = R_t$ of the wild-type variant. We assume that the reproduction number of the mutant (focal variant) is given by $R^{\mathrm{mt}}_t = (1+s) R_t$. The incidence of wild-type ($y^{\mathrm{wt}}_t$) and mutant ($y^{\mathrm{mt}}_t$) variants is governed by the discrete-time renewal equation

(1) \begin{align*} y^{\mathrm{wt}}_t &= R_t \sum_{t^{\prime}= 1}^{\infty} y^{\mathrm{wt}}_{t-t^{\prime}} K^{\mathrm{wt}}_{t^{\prime}} \\ y^{\mathrm{mt}}_t &= (1+s)R_t \sum_{t^{\prime}= 1}^{\infty} y^{\mathrm{mt}}_{t-t^{\prime}} K^{\mathrm{mt}}_{t^{\prime}} .\end{align*}

Here $K^{\mathrm{wt}}$ and $K^{\mathrm{mt}}$ denote the (discrete) probability mass functions of the generation time *T*_*G*_ of wild-type and mutant variants, respectively. By allowing for $K^{\mathrm{wt}} \neq K^{\mathrm{mt}}$, we take into account that the generation time can differ between variants. In Table 1 we give an overview of the variables and parameters of the model.

**Table 1.** Components of the discrete renewal model.

Symbol	Description
*Observed quantities*	
*C*_*t*_	Observed cases at time *t*
$F_t^{\mathrm{wt}}$ , $F_t^{\mathrm{mt}}$	Observed sequences at time *t* of wild-type and mutant variants, respectively
*Model variables*	
$y^{\mathrm{wt}}_t$ , $y^{\mathrm{mt}}_t$	Incidence at day *t* of wild-type and mutant variants
$H^{\mathrm{wt}}_t$ , $H^{\mathrm{mt}}_t$	Probability of reporting today if infected *t* days ago
$Y^{\mathrm{wt}}_t$ , $Y^{\mathrm{mt}}_t$	Incidence at day *t* corrected for a delay in reporting
*R*_*t*_	Time-dependent effective reproduction number of the wild-type variant
$K^{\mathrm{wt}}$ , $K^{\mathrm{mt}}$	Probability mass function of the generation time (*T*_*G*_) of each variant
*B*_*t*_, *Z*_*t*_	Wiener process and its independent Gaussian increments determining the trajectory of *R*_*t*_
*p*_*t*_	Fraction of mutant variant at time *t*, as predicted by the model
$p^\mathrm{agr}_w$	Predicted fraction of mutant variant, aggregated over week *w*
*Model parameters*	
*s*	Selection coefficient for the mutant variant
*τ*	Diffusion coefficient
*ϕ*	Dispersion of variant count data
$y_0^\mathrm{wt},\ y_0^\mathrm{mt}$	Initial incidence of wild-type and mutant variants
*b*_0_	Initial mutant frequency
*σ*_*C*_	Standard deviation of case counts
*Constants*	
$\alpha,\ \mu^\mathrm{wt}, \mu^\mathrm{mt}$	Shape and means of distribution of the generation time (*T*_*G*_)

The reproduction number of the wild type (*R*_*t*_) is modeled using a discrete-time geometric Gaussian process

(2) $$ R_t = R_0 \exp\left(\tau B_t - \tfrac12 \tau^2 t \right) $$

where $B_t = \sum_{t^{\prime}= 1}^t Z_{t^{\prime}}$ is the sum of *t* independent standard-normal increments $Z_1, \dots, Z_t$. The diffusion coefficient *τ* determines the randomness of the process, and the term $-\tfrac12 \tau^2 t$ makes sure that *R*_*t*_ has constant expectation. Notice that in this notation *R*_0_ is not the basic reproduction number, but just the effective reproduction number at time *t* = 0.

To link incidence *y*_*t*_ with observed cases *C*_*t*_, we have to compute the expected number of observed cases at each observation time *t*. Let $H^{v}_t$ denote the probability that a person infected *t* days ago with variant $v \in \{\mathrm{wt}, \mathrm{mt}\}$ is counted as positive today. The number of expected cases at time *t* is then equal to

(3) $$ Y^{v}_t = \sum_{t^{\prime}= 1}^{\infty} y^{v}_{t-t^{\prime}} H^{v}_{t^{\prime}}\,,\quad v \in \{\mathrm{wt}, \mathrm{mt}\}. $$

Note that the reporting delay applies to case counts, not sequences; sequence data may be deposited weeks after sampling, but they are dated with the sampling day. As we are primarily interested in the dynamics of the reproduction number and not in the absolute incidence, we will assume that the total reporting probability is equal to one $(\sum_{t=1}^{\infty} H^v_t = 1)$. Notice that we ignore potential differences in the reporting rate between the two variants. Furthermore, we make the simplifying assumption that the probability densities for reporting are identical to the probability densities for the generation time ($H^v_t = K^v_t$, with $v \in \{\mathrm{wt}, \mathrm{mt}\}$).

To model the expected mutant frequency *p*_*t*_, we assume that sequences are taken from individuals that are reported positive at time *t*. Hence, we simply take

(4) $$ p_t = \frac{Y^{\mathrm{mt}}_t}{Y_t^{\mathrm{wt}} + Y_t^{\mathrm{mt}}}. $$

We use a beta-binomial distribution for the likelihood of the observed number of wild-type and mutant sequences. This choice allows for over-dispersion in the variant count data due to biased sampling (e.g. over-reporting of Omicron as it was emerging, Scott et al., 2021), with the caveat it does not remove systematic biases. Let $F^{\mathrm{wt}}_t$ and $F^{\mathrm{mt}}_t$ denote the number of collected sequences at time *t* for wild-type and mutant variants, respectively. We then have

(5) $$ F^{\mathrm{mt}}_t \sim \mathrm{BetaBinom}(F^{\mathrm{mt}}_t + F^{\mathrm{wt}}_t, p_t\phi, (1-p_t)\phi), $$

where *ϕ* determines the dispersion of the distribution. In the limit $\phi \rightarrow \infty$ this converges to a binomial distribution.

We choose a discretized Gamma distribution as generation time distribution with shape parameter *α* = 4 and mean *µ* equal to 6, 4, and 3 days for Alpha, Delta, and Omicron, respectively (Hart et al., 2022a; Hart et al., 2022b; Abbott et al., 2022; Park et al., 2022b). The rate parameter is then given by $\beta = \alpha / \mu$. The discretization is as follows: $K_t \propto \frac{\beta^{\alpha}}{\Gamma(\alpha)}\int_{t-1}^t e^{-\beta x} x^{\alpha-1} dx$ for $t = 1, 2, \dots, W$, where *W* is a cutoff value equal to 15 days. The values *K*_*t*_ are then normalized such that they sum to 1.

To compute the incidence at times $t = 1, \dots, W$, we require the incidence at times $-W+1, -W+2, \dots, 0$. For this, we assume that prior to time *t* = 1 the effective reproduction number was constant and equal to *R*^*v*^_0_. We can then compute the incidence at these prior time points by making the *ansatz*$y^v_{-t} = y^v_0 \exp(-r^v t)$, where *r*^*v*^ is the exponential growth rate of variant *v*. Plugging this into the renewal equation, we get

(6) $$ y^v_0 = R^v_0 \sum_{t=1}^{\infty} y^v_{-t} K^v_t = R^v_0 y^v_0\sum_{t=1}^{\infty}\exp(-r^v t) K^v_t $$

and hence $R^v_0 \mathbb{E}[\exp(-r^v T_G)] = 1$, which is known as the Lotka–Euler equation. We then ignore the fact that we use a discrete-time model and use the moment-generating function of the continuous Gamma distribution, and we can find *r*^*v*^ by solving

(7) $$ R^v_0 \left(1 + \frac{r^v}{\beta}\right)^{-\alpha} = 1. $$

This leads to $r^v = \beta(\sqrt[\alpha]{R_0^v} - 1)$. This calculation is done for both the wild type and mutant, using variant-specific parameters *α* and *β*. The initial incidence for wild type and mutant is parameterized as follows. We introduce parameters *y*_0_ and *b*_0_, denoting the total initial incidence, and the fraction due to the mutant, respectively. Then, we set $y^{\mathrm{mt}}_0 = b_0 y_0$ and $y^{\mathrm{wt}}_0 = (1-b_0) y_0$.

We use a phenomenological log-normal observation model to fit the predicted case counts ($Y_t = Y^{\mathrm{wt}}_t + Y^{\mathrm{mt}}_t$) to the observed case counts $C_t \sim \mathrm{Lognormal}(\log(Y_t), \sigma_C)$. To remove weekend patterns in under-reporting in the data, we aggregate the daily observations (case counts and number of sequences collected for both variants) to the week level. The same aggregation is done for the model predictions of these observations, so that we can compute the likelihood of the aggregated observations. For the aggregation of the predicted frequencies, we used a weighted average, thereby accounting for the fact that case counts can vary markedly within a week. More precisely, the aggregated predicted mutant frequency $p^{\mathrm{agr}}_w$ at week *w* is equal to

(8) $$ p^{\mathrm{agr}}_w = \sum_{t=7(w-1)+1}^{7w} p_{t} \frac{Y_{t}}{Y_w^{\mathrm{agr}}}, $$

where $Y_w^{\mathrm{agr}}$ is the aggregated predicted case count at week *w*.

As we wish to estimate effects of covariates on the selective advantage in different regions, we use a Bayesian mixed-effects model (or hierarchical model) in which region-specific parameters are determined by unobserved random effects and possibly fixed effects dependent on the covariates. The selective advantage, *s*, can thus take a different value for each region. The *z*-scores of the covariates described above are collected in the design matrix *M*, and we write *w*_*s*_ for the vector of weights of these covariates. We assume that $1+s$ has a log-normal distribution with location $\mu_s + M w_s$ and scale *σ*_*s*_. The dispersion parameter and initial incidence similarly vary among countries, with hyperparameters *µ*_*ϕ*_, $\mu_{y_0}$, and $\sigma_{y_0}$, but for these parameters only random effects are modeled.

The model is summarized in Tables 1 and 2. We focus our results on the selective advantage, *s*, of the focal variant in each country and on the mean of its distribution across countries. We implemented the model in Stan (Stan Development Team, 2021).

**Table 2.** Priors of the Bayesian mixed-effects model.

Parameter	Prior	Hyper-prior
*Region-specific parameters*		
Selective advantage (*s*)	$1+s \sim \mathrm{Lognormal}$ $(\mu_s + M w_s, \sigma_s)$	$\mu_s \sim \mathcal{N}(0,10)$ , $\sigma_s \sim \mathrm{Exp}(0.1)$
Initial mutant frequency (*b*_0_)	$b_0 \sim \mathrm{Exp}(10)$	–
Dispersion parameter (*ϕ*)	$1/\phi \sim \mathrm{Exp}(1/\mu_{\phi})$	$\mu_{\phi} \sim \mathrm{Exp}(100)$
Initial incidence (*y*_0_)	$y_0 \sim \mathrm{Lognormal}$ $(\mu_{y_0}, \sigma_{y_0})$	$\mu_{y_0} \sim \mathcal{N}(0,10)$ , $\sigma_{y_0} \sim \mathrm{Exp}(0.1)$
Initial reproduction number (*R*_0_)	$R_0 \sim \mathrm{Lognormal}$ $(0,1)$	–
*Global parameters*		
Diffusion reproduction number (*τ*)	$\tau \sim \mathrm{Exp}(1)$	–
Standard deviation cases (*σ*_*C*_)	$\sigma_C \sim \mathrm{Exp}(1)$	–
Covariate weight vector (*w*_*s*_)	$w_s \sim \mathcal{N}(0,10)$	–

## 3. Results and Discussion

**Figure 1.**
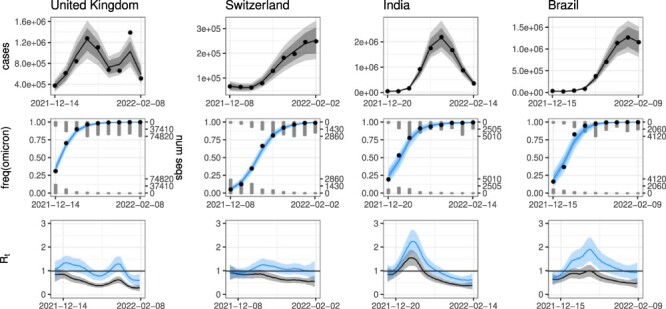
Example model fits to the rise of Omicron. (Fits for all countries are shown in Fig. S2.) Top row: case counts, binned by week. Middle row: variant data, also binned by week. Number of sequences is shown by gray histograms for Omicron at the top and all other variants at the bottom. The observed frequency of Omicron is shown by the black dots, and model predictions are in blue. Bottom row: growth rates, predicted by day. Model-predicted growth rates are shown for the epidemic as a whole (both variants with their true frequencies; blue) and for only the non-Omicron variant(s) (as if the Omicron frequency were zero; gray). Model predictions are shown with solid lines (median), dark shading (95 per cent credible interval [CrI], which includes uncertainty in the model parameters), and light shading (95 per cent posterior predictive interval, which additionally includes sampling noise).

Our model outputs case counts, variant frequencies, and epidemic growth rates over time shown in Fig. 1 for Omicron in selected countries and Fig. S2 and Fig. S3 for Omicron and Delta in all countries. Overall, the model fit the data very well, with, for Omicron, a mean absolute error in the variant frequency of only 2.5 percentage points for data aggregated by week, or 5.3 percentage points for daily data (Fig. S4). Fits for Delta were only slightly worse, with mean absolute errors of 5.8 and 8.6 percentage points by week and day, respectively. Aggregating up to the week scale addressed nearly all the structural anomalies in the epidemiological data, notably non-uniform reporting by day of week, without altering the total number of cases. Even for remaining anomalous data (e.g. the spike in UK case counts due to sudden reporting of past reinfections, Fig. 1), the model predicted a reasonably smooth estimate of the trend.

The selection coefficient inferred for a focal variant is influenced by its generation time (serial interval) relative to the background variants against which it is competing. In particular, other methods that assume the same generation time for the focal and other variants (e.g. van Dorp et al., 2021; Chen et al., 2021) will underestimate the strength of selection when the focal variant has a shorter generation time, as did both Delta and Omicron (Park et al., 2022a). For example, our main results use a mean generation time for Omicron of 3 days, yielding a point estimate of 0.69 for the overall selective advantage of Omicron. Decreasing the mean generation time to 2 days, consistent with some early studies (Abbott et al., 2022), decreases this value to 0.64 (Fig. S5). Note, however, that this is still far larger than zero, arguing that shorter generation times are not solely responsible for the increased fitness of Omicron. Conversely, assuming that the Omicron generation time is the same as the background variants (4 days, because most were Delta) increases the value to 0.84, substantially overstating the selective advantage.

### 3.1 Describing heterogeneity in selective advantage

**Figure 2.**
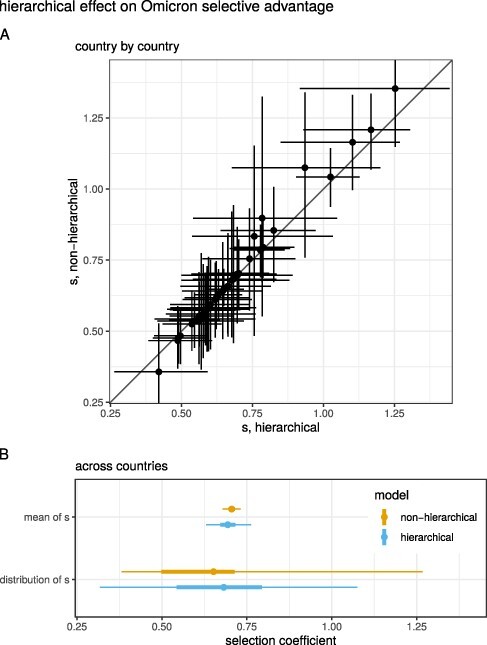
(A) Estimates of the selection coefficient, *s*, for each country, under the hierarchical or nonhierarchical models. Points and lines show the median and 95 per cent CrI. The slope of the data is steeper than the 1:1 line, meaning that the estimates are less extreme for the hierarchical model. (B) Summaries of estimates for the mean value of *s* for Omicron and for the distribution of values of *s* across countries, computed from the hierarchical or nonhierarchical models. Points show the median or mean, and thick and thin lines show the 50 per cent and 95 per cent CrI or CI.

Comparing the model-fitted estimates of Omicron’s selective advantage, *s*, among countries shows wide variation in their values (Fig. 2A). This is consistent with previous work for other variants, which also found large heterogeneity among countries and US states (van Dorp et al., 2021; Figgins and Bedford, 2021). Our model assumes that the ratio of reproduction numbers ($1+s$) for each country is drawn from a log-normal distribution, which is simultaneously estimated from the data. This hierarchical structure minimizes the influence of structural biases in any one country’s data, leading to a more robust and interpretable estimate of both the overall selection effect and the heterogeneity at country level.

To examine the effect of the hierarchical model structure on our results, we additionally fit a nonhierarchical equivalent of our model in which *s* for each country is not affected by other countries (Fig. 2). We highlight two outcomes from this comparison: estimates for individual countries and conclusions drawn across countries. First, with both the hierarchical and nonhierarchical models, we found substantial heterogeneity in the selection coefficient, *s*, at the country level for Omicron (Fig. 2A). In the hierarchical model, more extreme values of *s*—which might arise from real effects or data quality issues—are reined in by the moderating effects of other countries. Second, there are different ways to summarize the overall selective advantage of Omicron, encompassing the heterogeneity across countries (Fig. 2B). One approach is to provide a typical value. For the hierarchical model this is the mean of the higher-level distribution, which has an associated uncertainty (0.69 [0.63, 0.76] 95 per cent CrI). For the nonhierarchical model, this could be the mean of the country-level point estimates of *s*, with uncertainty summarized as the standard error of the mean (0.71 [0.68, 0.73] 95 per cent confidence interval [CI]). Another approach is to describe the variation across countries. For the hierarchical model this is the higher-level distribution itself, with $s \sim \mathrm{Lognormal}(\mu_s, \sigma_s) - 1$ (Table 2), including the uncertainties on those parameters (0.68 [0.32, 1.08] 95 per cent CrI). For the nonhierarchical model, we summarized *s* across countries by concatenating the Markov chain Monte Carlo samples from *s* for all countries (0.65 [0.38, 1.27] 95 per cent CrI). Overall, regardless of the statistics used, Omicron has a very large selective advantage, and its typical value can be estimated much more precisely than the variation among countries.

### 3.2 Explaining heterogeneity in selective advantage

With so much variation in *s* among countries, we asked whether causes of that variation could be identified. We looked for associations between country-level *s* and other country-level attributes, both in the model structure and in the model outputs.

**Figure 3.**
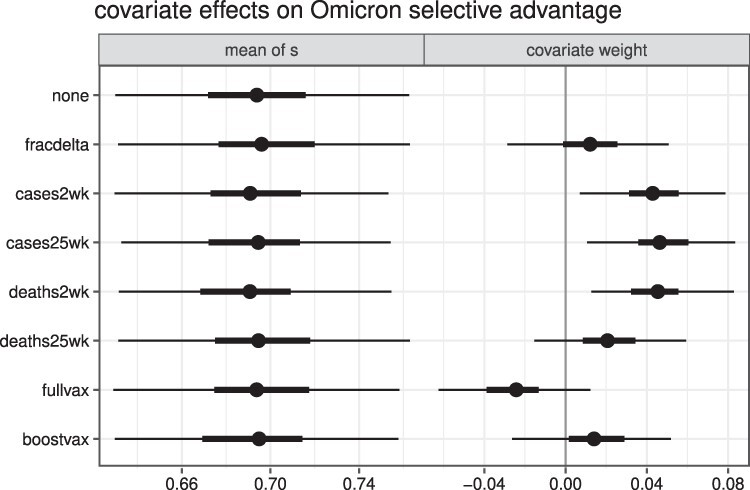
Effects of each of seven country-level covariates on *s*. The median, 50 per cent, and 95 per cent CrI for the mean of the hierarchical distribution (left) and the estimated weight of the covariate in the model are plotted. Covariate weights are shown on a standardized, transformed scale, but the natural scales are shown in Fig. S1, and the effect magnitudes on the natural scales are discussed in the results text.

Because Omicron has been shown to evade immunity from prior infection or vaccination, we used our mixed-effects model to test whether the extent of immunity in a country at the time of Omicron’s arrival explained variation in Omicron’s selective advantage. Three of our four proxy variables for natural immunity—based on the numbers of cases or deaths in the weeks leading up to Omicron’s arrival in the country—showed a significant effect (Fig. 3). The signs of the effects mean that Omicron had a stronger selective advantage in countries with more natural immunity, consistent with the idea that the part of Omicron’s fitness advantage is immune escape. To understand the effect size, for each significant covariate, we compared the predicted *s* in a hypothetical population where the covariate was 0 against a hypothetical population where the covariate took the highest empirical value in the data (2.11 per cent for ‘cases2wk’, 10.21 per cent for ‘cases25wk’, and 0.03 per cent for ‘deaths2wk’). This comparison yielded a difference in *s* of 0.26, 0.30, and 0.33, respectively. Neither past cases or excess deaths are ideal proxies for the population-level immunological status of different countries, due to differences in age-stratified infection and death risk in addition to likely heterogeneity in case detection rate between and within countries. However, the significant correlation of both recent cases and excess deaths with increasing *s* suggests that country-level immunological status could partially explain the differences in selection effects.

The selective advantage we estimate for each focal variant is by definition relative to the fitnesses of the preexisting variants against which it is competing. Modeling all variants explicitly would substantially expand the complexity of our model, so we instead explored this topic in three simpler ways. First, we hypothesized that Omicron might have a lesser selective advantage when it was competing primarily against Delta (than against other variants, which were outcompeted by Delta in much of the world), but including the prevalence of Delta as a covariate showed no significant effect (Fig. 3). Second, we wondered if the higher selective advantage of Omicron in some countries could be explained by the prevalence of later Omicron subvariants in those countries, but we found no association between selective advantage and the genetic composition of Omicron (Fig. S6). And third, we compared our selection estimates against the amount of diversity in the preexisting, background variant landscapes, as measured by either the number of unique Pango lineages (variant richness) or the probability that any two sequences drawn at random are from different Pango lineages (Simpson’s $1-D$, which incorporates abundances) (Fig. S7). We found a consistently positive correlation between background diversity and the point estimate of *s*, although it was only significant in some cases. One interpretation is that higher-diversity background variant communities lack a relatively high-fitness variant and thus pose less competition for the invading focal variant. We also wondered if uncertainty in the *s* for a country could be driven by diversity in its background variants, but we found no correlation between these quantities (Fig. S7).

**Figure 4.**
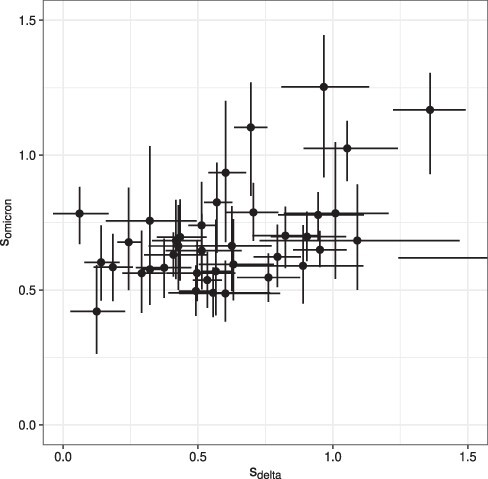
Estimates of *s* for each country, for Omicron and Delta. The median and 95% CrI are shown as points and lines respectively. One country not fully shown has *s*_delta_ = 1.98. Note that even though Omicron universally outcompeted Delta, *s*_omicron_ can be less than *s*_delta_ because the selective advantage quantifies the fitness of the focal variant relative to the fitness of the background variant(s). Data are provided in Table S1.

Alternatively, variation among countries could be explained not by the landscape specifically faced by Omicron, but instead by national systemic differences. With so many possible fixed country-level covariates that are likely strongly correlated, identifying specific causes is fraught. However, if any such factors do play a consistent role, we would expect their effects to be seen for variants in addition to Omicron. We therefore compared country-specific median estimates of *s* for Omicron with those for Delta and found a significant correlation (Pearson’s correlation *r* = 0.37, *P* = 0.02; Spearman’s correlation *ρ* = 0.40, *P* = 0.01; Fig. 4). We thus suggest that systematic differences between countries play some role in explaining the selective advantage of new variants.

### 3.3 Conclusion

We found a very large selective advantage for Omicron (growth rate nearly 1.7× that of the previously circulating variants, mostly Delta), but also very large heterogeneity among countries (the 95 per cent CrI spans 1.3× to 2.0×). With selection coefficients this large, it is important to recall that their magnitude is also affected by the absolute growth rate (van Dorp et al., 2021; Chen et al., 2021). We thus used not only variant counts and a model of variant frequency dynamics, but also a renewal model that included case count data (as did Figgins and Bedford, 2021; Park et al., 2022a). Additionally, we found that the selective advantage for Omicron was robust to changes in the generation time assumptions, i.e. the advantage of Omicron is not due solely to a shorter generation time.

Our hierarchical modeling approach provides a natural way to express both the overall advantage of a variant and its distribution across countries. Previous work has used a similar strategy for smaller geographic scales (Paton et al., 2022) and for mutations across lineages (Obermeyer et al., 2022). The hierarchical approach is particularly useful for global-scale analyses, in which one would like to include data from all countries even though they have vastly different data qualities and quantities. It also extends well to multiple levels, allowing, for example, a future analysis that includes heterogeneity among states within countries, in addition to among countries.

Our mixed-effects framework allows one to test hypotheses about country-level properties that might explain differences in selective advantage among countries. That such fixed properties exist is supported by the significant positive correlation between selection strength for Delta and Omicron at the country level. Identifying potential causal systemic differences may require a different approach, however, as there are many possible factors—population density, national wealth, type of governance, or health-care system, to name just a few—and likely strong correlations between them.

Previous work, both laboratory assays (Lu et al., 2021; Cele et al., 2021; Planas et al., 2021) and population-level studies (Pearson et al., 2021; Paton et al., 2022; Pulliam et al., 2022), shows that Omicron better escapes natural and vaccine-induced immunity than do other variants. Consistent with this and despite using covariates that are highly imperfect proxies for true population-level immunity, we found a higher selection coefficient for Omicron in countries with more immunity.

Future studies of SARS-CoV-2 variant dynamics could focus on scientific explanations for heterogeneity in the spread of different variants and why some countries seem to slow the spread of new variants. The modeling framework and code presented in this paper facilitates this type of work by allowing for joint estimation of selection effects and explanatory variables.

## Supplementary Material

veac089_Supp

## Data Availability

All data used in this study are publicly available. The scripts used for data processing and statistical inference can be found at https://github.com/eeg-lanl/sarscov2-selection.
