# Supplementary material for: Global estimates of the fitness advantage of SARS-CoV-2 variant Omicron
Source: Virus Evol. 2022 Oct 7;8(2):veac089. doi: 10.1093/ve/veac089 (PMC9615435; doi:10.1093/ve/veac089)
Supplement: veac089_Supp [file veac089_supp.zip › suppl_data/delta-fits.pdf]

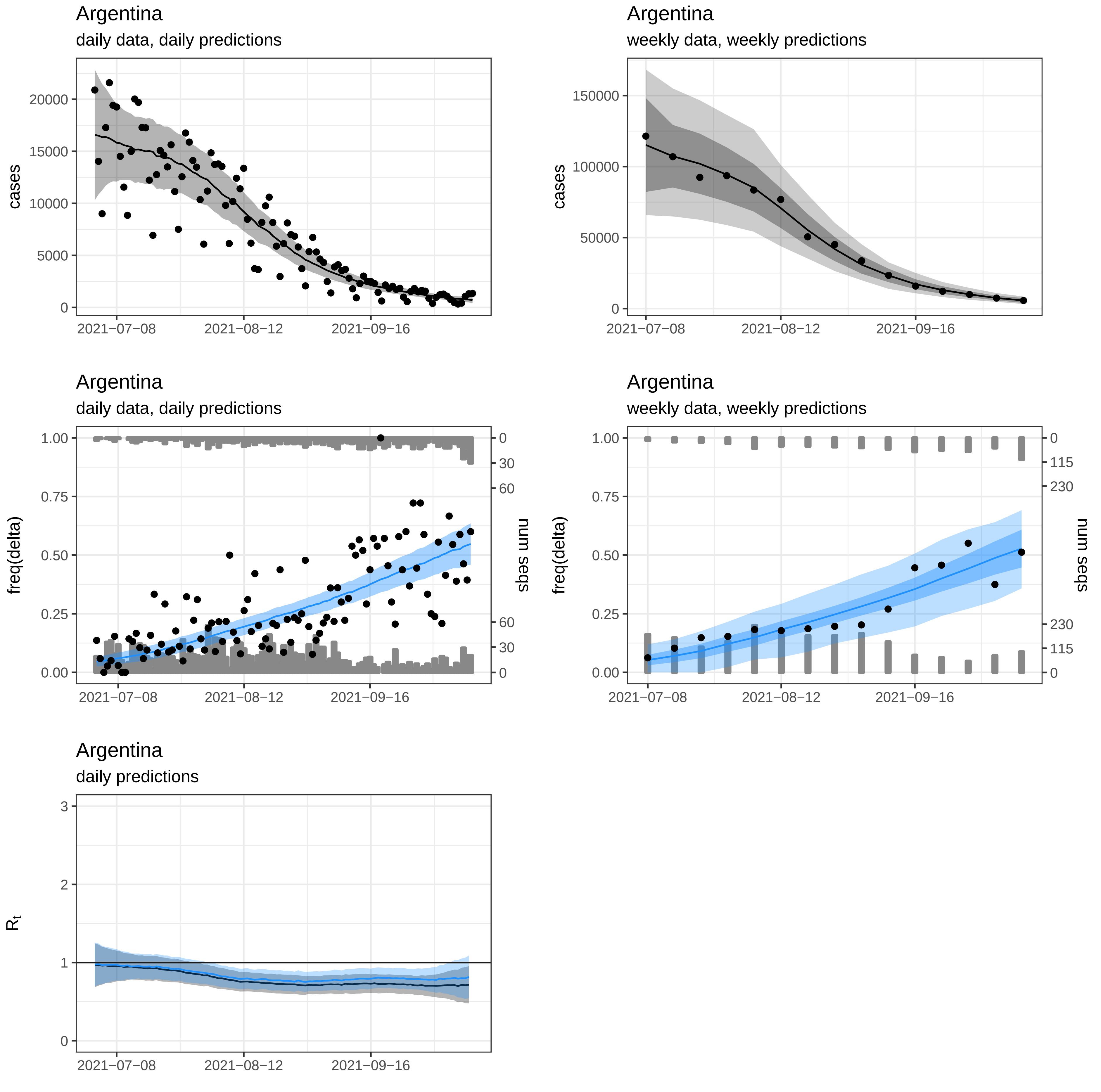
Argentina
daily data, daily predictions
cases
20000
15000
10000
5000
0
2021−07−08
2021−08−12
2021−09−16
Argentina
weekly data, weekly predictions
cases
150000
100000
50000
0
2021−07−08
2021−08−12
2021−09−16
Argentina
daily data, daily predictions
freq(delta)
1.00
0.75
0.50
0.25
0.00
num seqs
0
30
60
60
30
0
2021−07−08
2021−08−12
2021−09−16
Argentina
weekly data, weekly predictions
freq(delta)
1.00
0.75
0.50
0.25
0.00
num seqs
0
115
230
230
115
0
2021−07−08
2021−08−12
2021−09−16
Argentina
daily predictions
R_t
3
2
1
0
2021−07−08
2021−08−12
2021−09−16

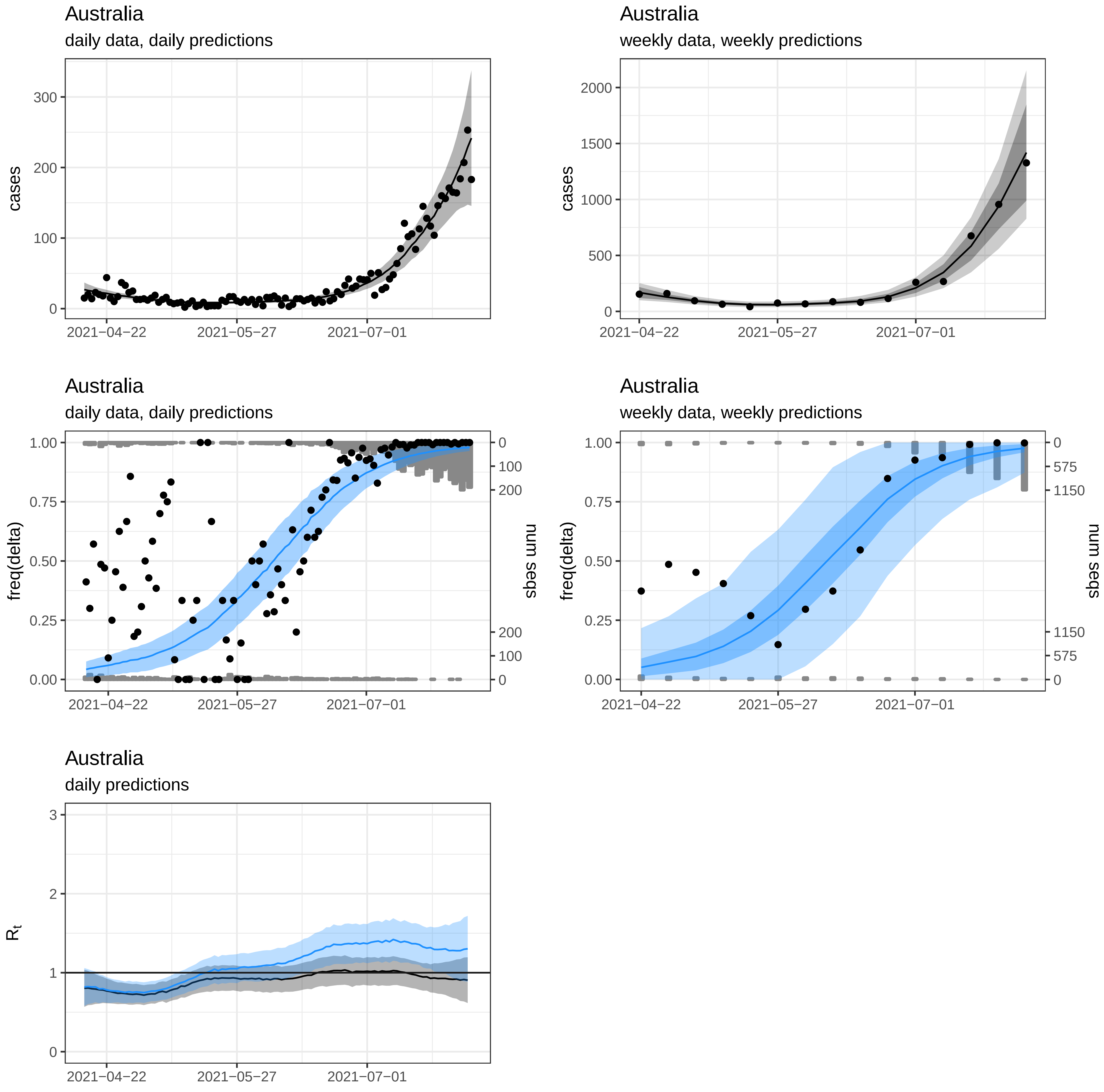
Australia
daily data, daily predictions
cases
300
200
100
0
2021−04−22
2021−05−27
2021−07−01
Australia
weekly data, weekly predictions
cases
2000
1500
1000
500
0
2021−04−22
2021−05−27
2021−07−01
Australia
daily data, daily predictions
freq(delta)
1.00
0.75
0.50
0.25
0.00
num seqs
0
100
200
200
100
0
2021−04−22
2021−05−27
2021−07−01
Australia
weekly data, weekly predictions
freq(delta)
1.00
0.75
0.50
0.25
0.00
num seqs
0
575
1150
1150
575
0
2021−04−22
2021−05−27
2021−07−01
Australia
daily predictions
R_t
3
2
1
0
2021−04−22
2021−05−27
2021−07−01

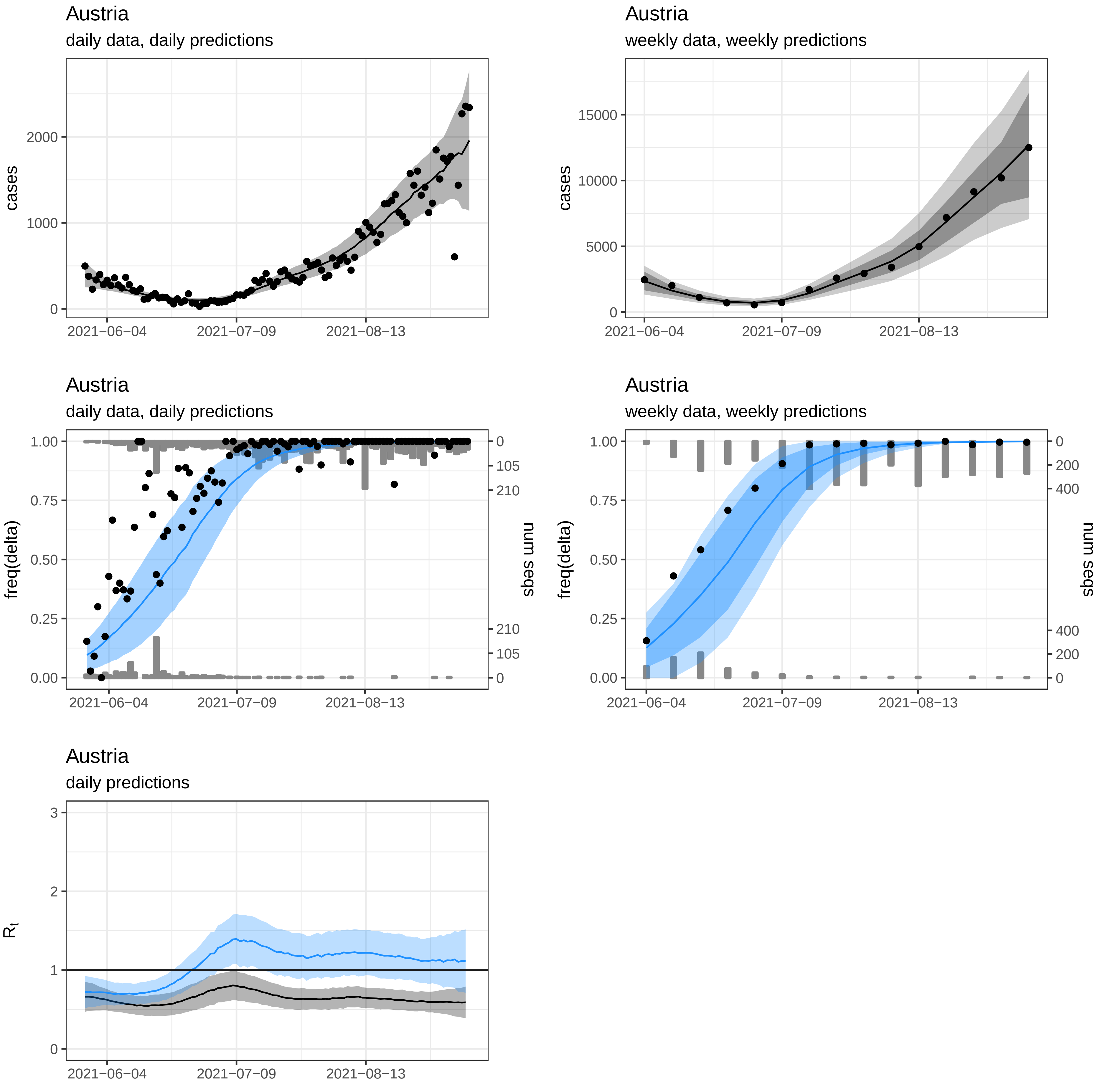
Austria
daily data, daily predictions
cases
2000
1000
0
2021−06−04
2021−07−09
2021−08−13
Austria
weekly data, weekly predictions
cases
15000
10000
5000
0
2021−06−04
2021−07−09
2021−08−13
Austria
daily data, daily predictions
freq(delta)
1.00
0.75
0.50
0.25
0.00
num seqs
0
105
210
210
105
0
2021−06−04
2021−07−09
2021−08−13
Austria
weekly data, weekly predictions
freq(delta)
1.00
0.75
0.50
0.25
0.00
num seqs
0
200
400
400
200
0
2021−06−04
2021−07−09
2021−08−13
Austria
daily predictions
R_t
3
2
1
0
2021−06−04
2021−07−09
2021−08−13

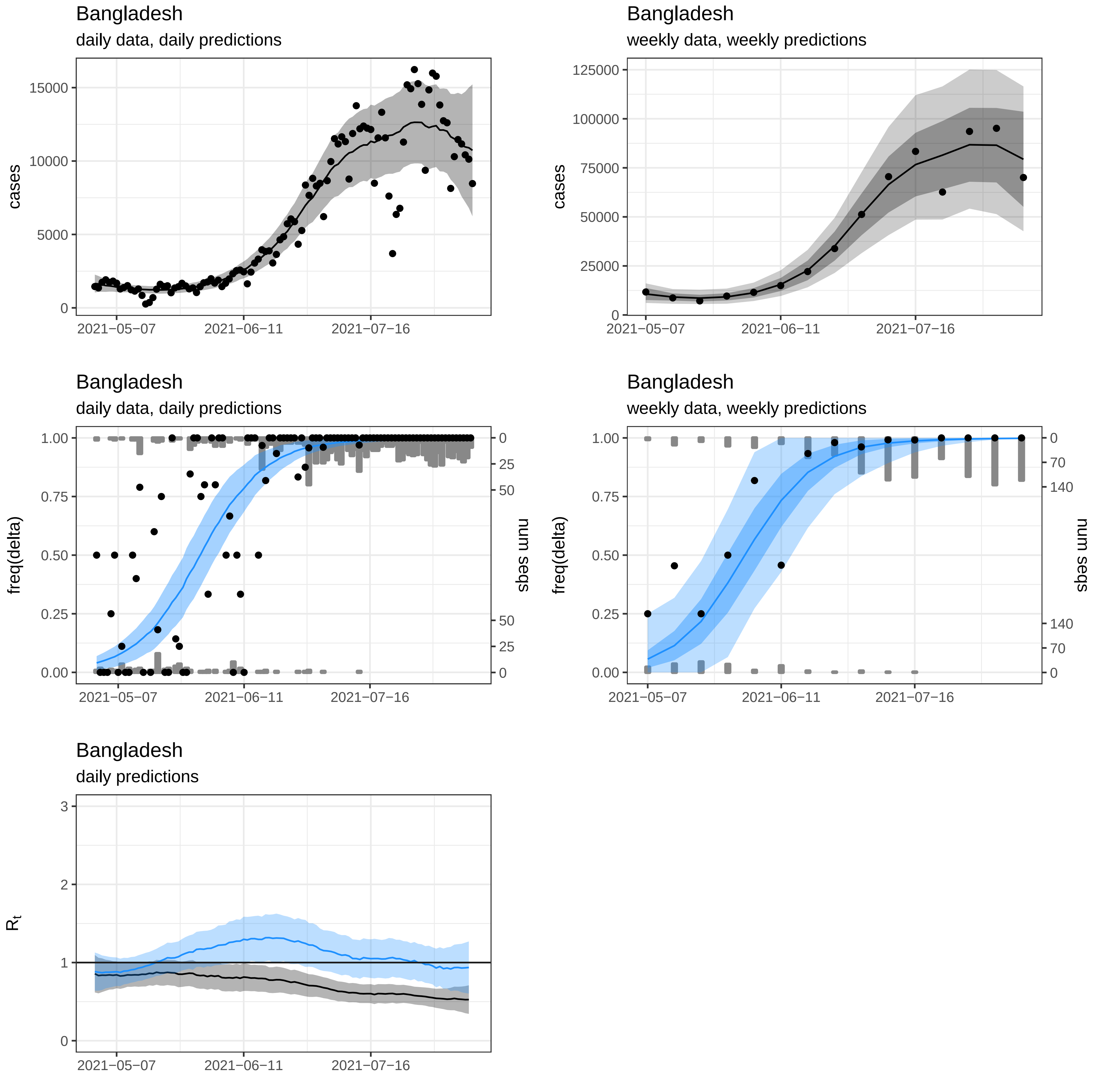

Bangladesh
daily data, daily predictions
cases
0
5000
10000
15000
2021−05−07
2021−06−11
2021−07−16
Bangladesh
weekly data, weekly predictions
cases
0
25000
50000
75000
100000
125000
2021−05−07
2021−06−11
2021−07−16
Bangladesh
daily data, daily predictions
freq(delta)
0.00
0.25
0.50
0.75
1.00
num seqs
0
25
50
2021−05−07
2021−06−11
2021−07−16
Bangladesh
weekly data, weekly predictions
freq(delta)
0.00
0.25
0.50
0.75
1.00
num seqs
0
70
140
2021−05−07
2021−06−11
2021−07−16
Bangladesh
daily predictions
R_t
0
1
2
3
2021−05−07
2021−06−11
2021−07−16

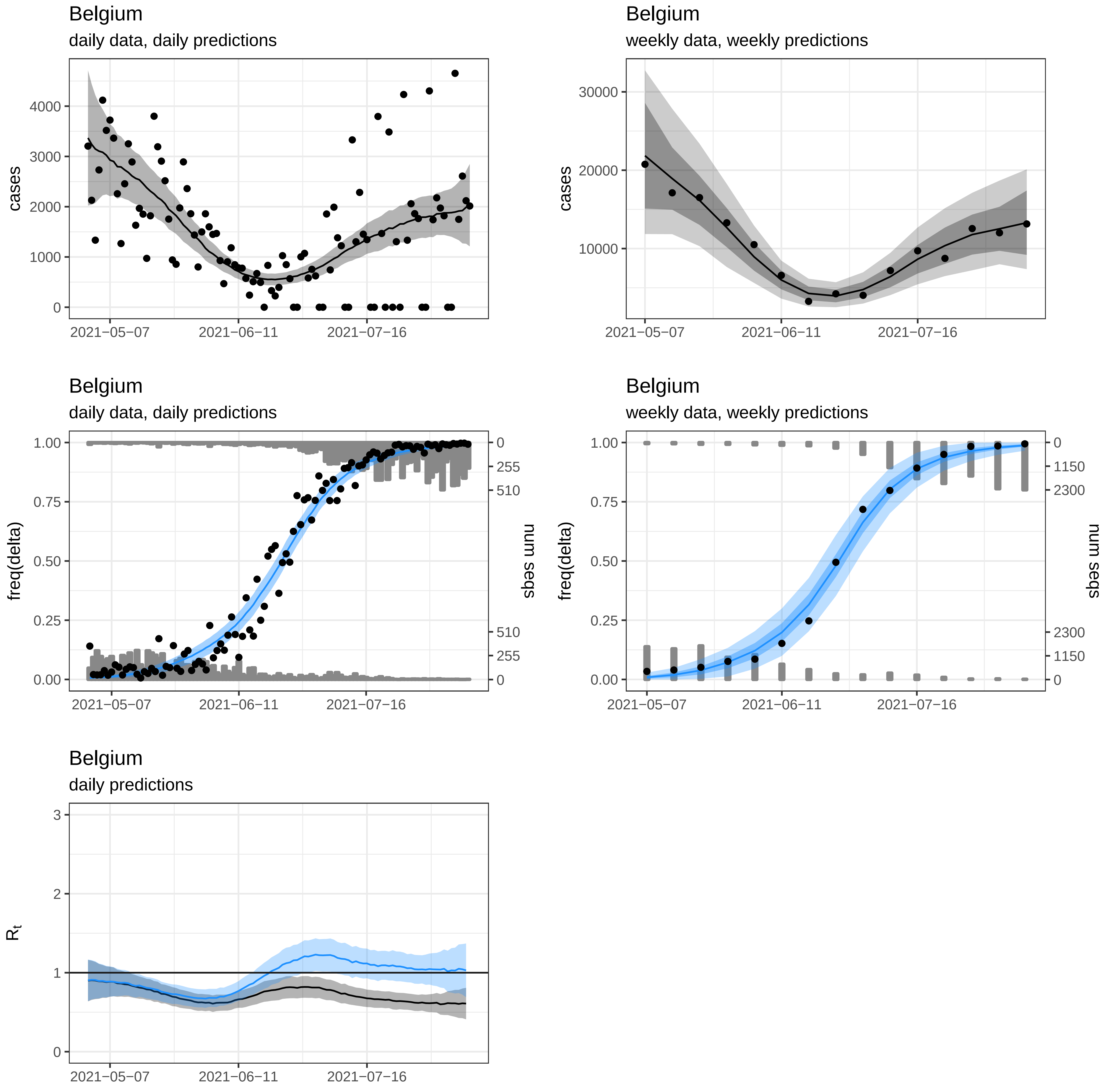
Belgium
daily data, daily predictions
cases
4000
3000
2000
1000
0
2021−05−07
2021−06−11
2021−07−16
Belgium
weekly data, weekly predictions
cases
30000
20000
10000
2021−05−07
2021−06−11
2021−07−16
Belgium
daily data, daily predictions
freq(delta)
1.00
0.75
0.50
0.25
0.00
num seqs
0
255
510
510
255
0
2021−05−07
2021−06−11
2021−07−16
Belgium
weekly data, weekly predictions
freq(delta)
1.00
0.75
0.50
0.25
0.00
num seqs
0
1150
2300
2300
1150
0
2021−05−07
2021−06−11
2021−07−16
Belgium
daily predictions
R_t
3
2
1
0
2021−05−07
2021−06−11
2021−07−16

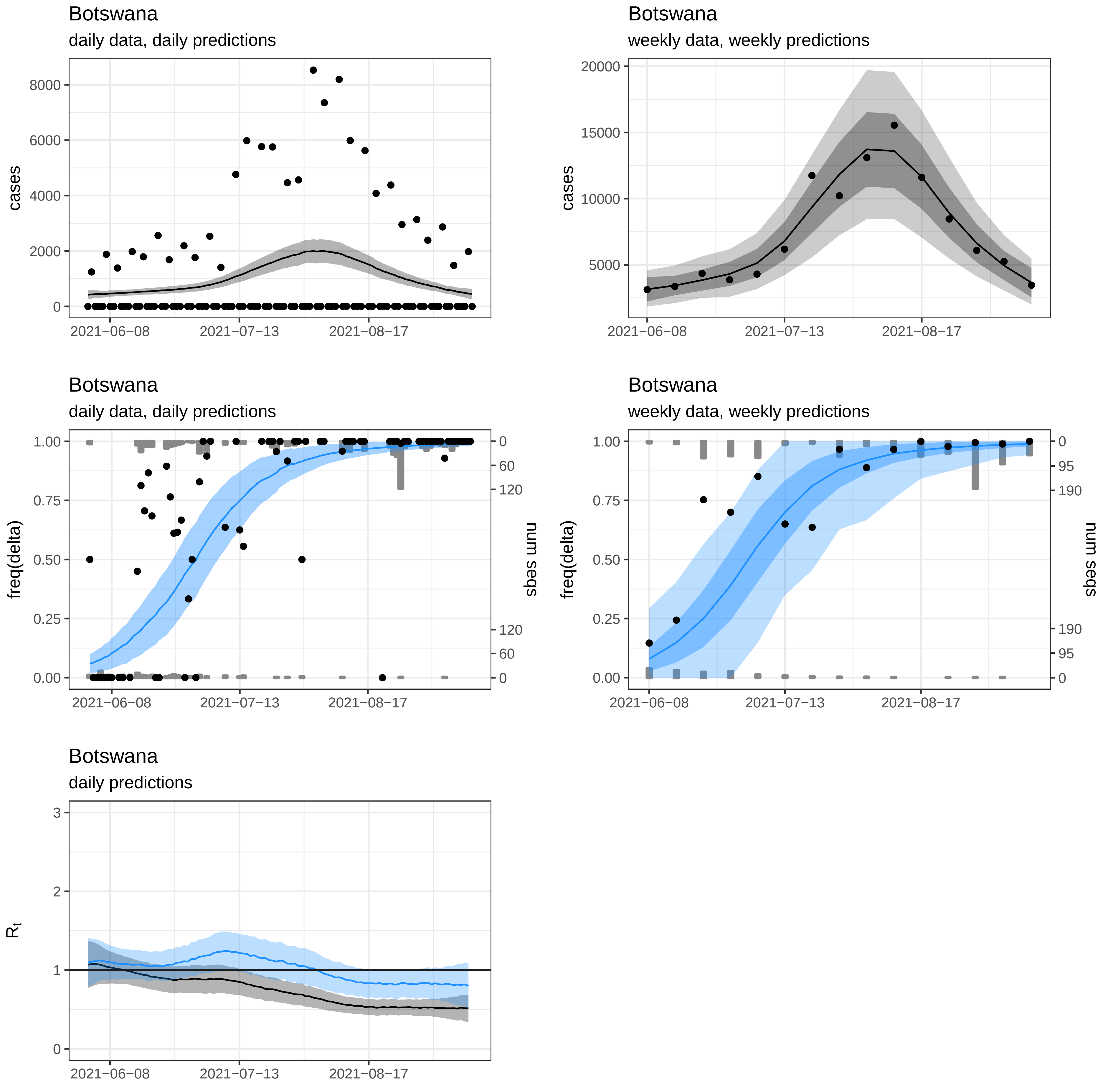

Botswana
daily data, daily predictions
cases
8000
6000
4000
2000
0
2021−06−08
2021−07−13
2021−08−17
Botswana
weekly data, weekly predictions
cases
20000
15000
10000
5000
2021−06−08
2021−07−13
2021−08−17
Botswana
daily data, daily predictions
freq(delta)
1.00
0.75
0.50
0.25
0.00
num seqs
0
60
120
120
60
0
2021−06−08
2021−07−13
2021−08−17
Botswana
weekly data, weekly predictions
freq(delta)
1.00
0.75
0.50
0.25
0.00
num seqs
0
95
190
190
95
0
2021−06−08
2021−07−13
2021−08−17
Botswana
daily predictions
R_t
3
2
1
0
2021−06−08
2021−07−13
2021−08−17

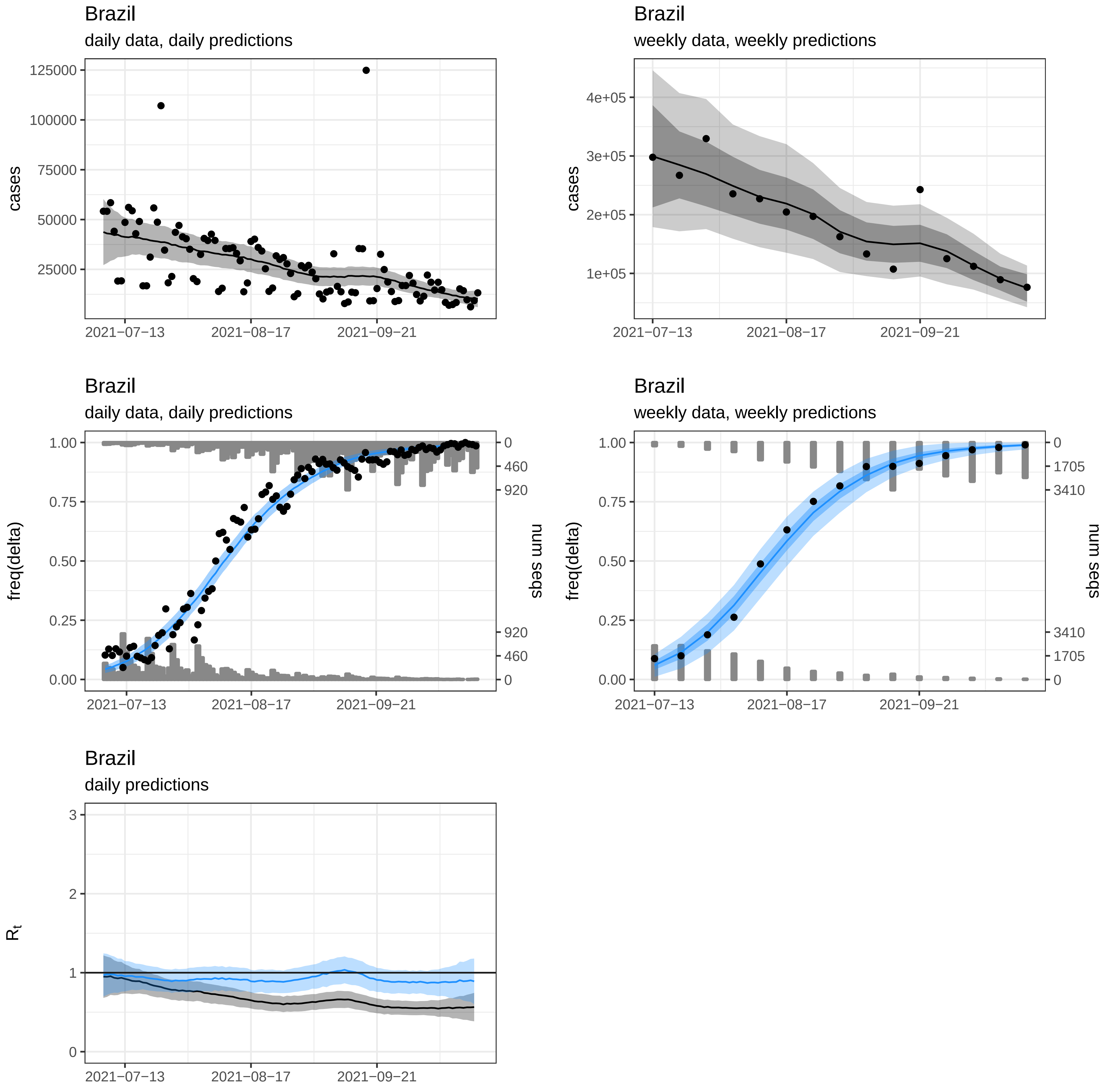
Brazil
daily data, daily predictions
cases
125000
100000
75000
50000
25000
2021−07−13
2021−08−17
2021−09−21
Brazil
weekly data, weekly predictions
cases
4e+05
3e+05
2e+05
1e+05
2021−07−13
2021−08−17
2021−09−21
Brazil
daily data, daily predictions
freq(delta)
1.00
0.75
0.50
0.25
0.00
num seqs
0
460
920
920
460
0
2021−07−13
2021−08−17
2021−09−21
Brazil
weekly data, weekly predictions
freq(delta)
1.00
0.75
0.50
0.25
0.00
num seqs
0
1705
3410
3410
1705
0
2021−07−13
2021−08−17
2021−09−21
Brazil
daily predictions
R_t
3
2
1
0
2021−07−13
2021−08−17
2021−09−21

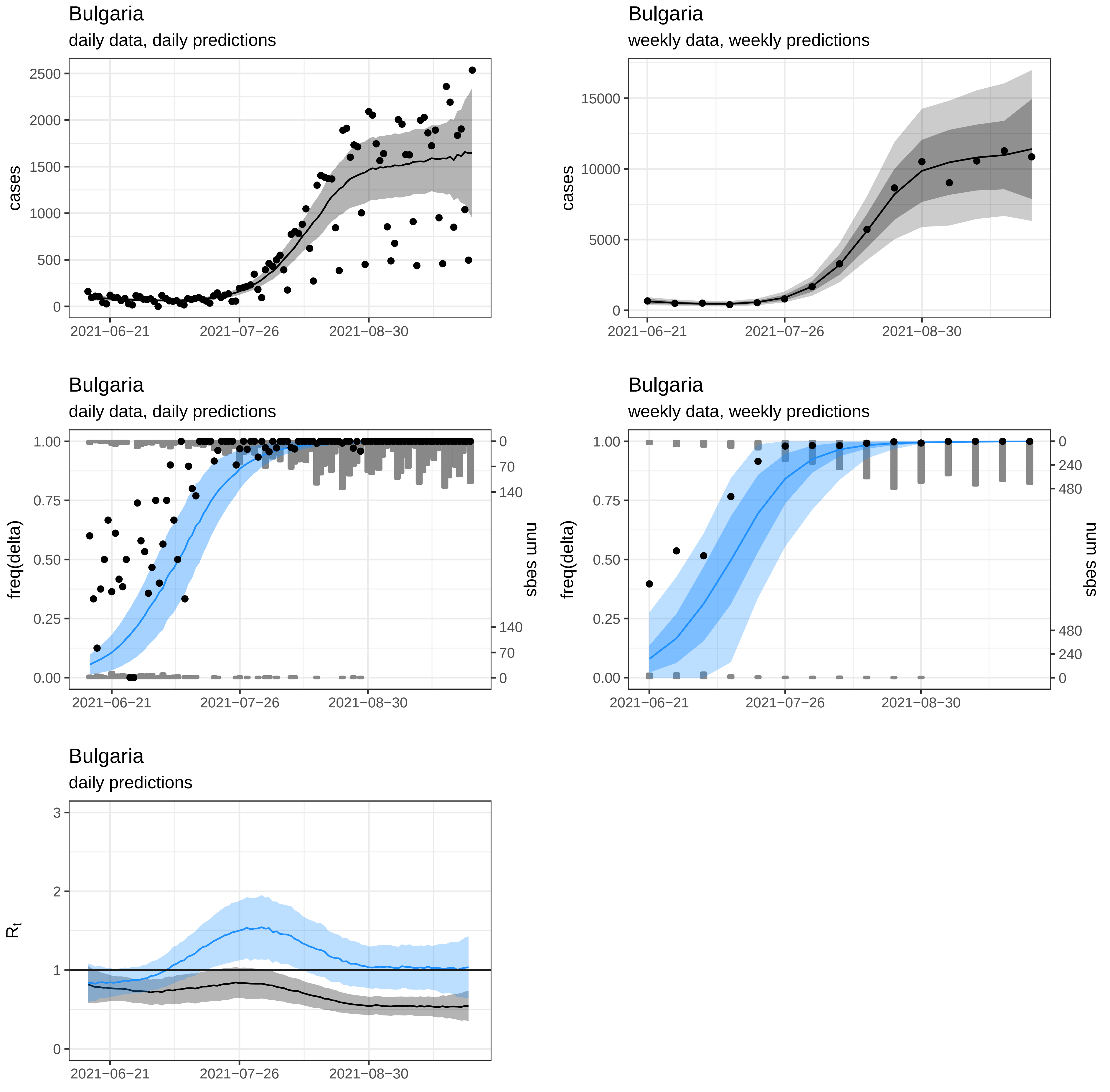
Bulgaria
daily data, daily predictions
cases
2500
2000
1500
1000
500
0
2021−06−21
2021−07−26
2021−08−30
Bulgaria
weekly data, weekly predictions
cases
15000
10000
5000
0
2021−06−21
2021−07−26
2021−08−30
Bulgaria
daily data, daily predictions
freq(delta)
1.00
0.75
0.50
0.25
0.00
num seqs
0
70
140
140
70
0
2021−06−21
2021−07−26
2021−08−30
Bulgaria
weekly data, weekly predictions
freq(delta)
1.00
0.75
0.50
0.25
0.00
num seqs
0
240
480
480
240
0
2021−06−21
2021−07−26
2021−08−30
Bulgaria
daily predictions
R_t
3
2
1
0
2021−06−21
2021−07−26
2021−08−30

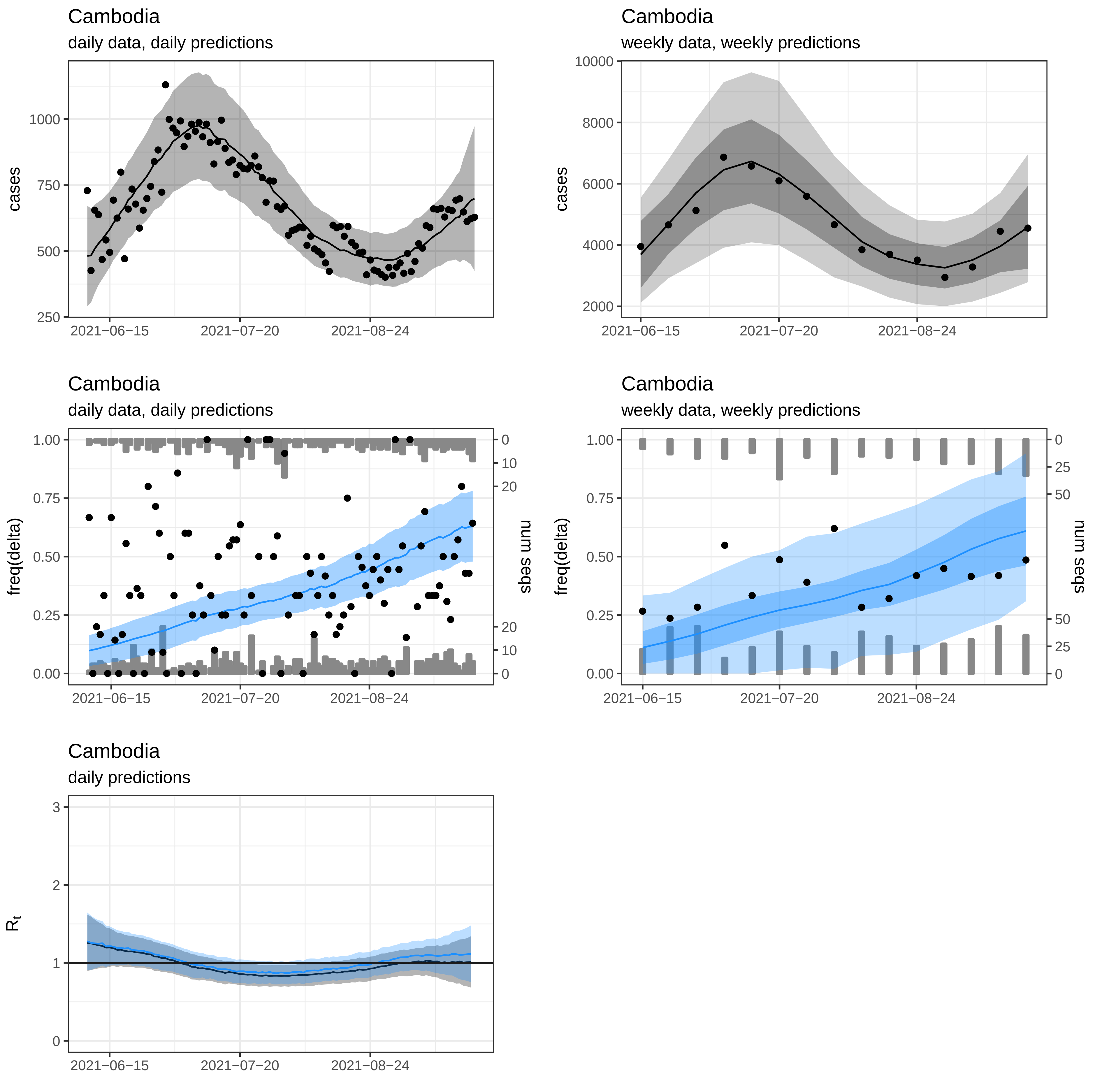
Cambodia
daily data, daily predictions
cases
250
500
750
1000
2021−06−15
2021−07−20
2021−08−24
Cambodia
weekly data, weekly predictions
cases
2000
4000
6000
8000
10000
2021−06−15
2021−07−20
2021−08−24
Cambodia
daily data, daily predictions
freq(delta)
0.00
0.25
0.50
0.75
1.00
num seqs
0
10
20
2021−06−15
2021−07−20
2021−08−24
Cambodia
weekly data, weekly predictions
freq(delta)
0.00
0.25
0.50
0.75
1.00
num seqs
0
25
50
2021−06−15
2021−07−20
2021−08−24
Cambodia
daily predictions
R_t
0
1
2
3
2021−06−15
2021−07−20
2021−08−24

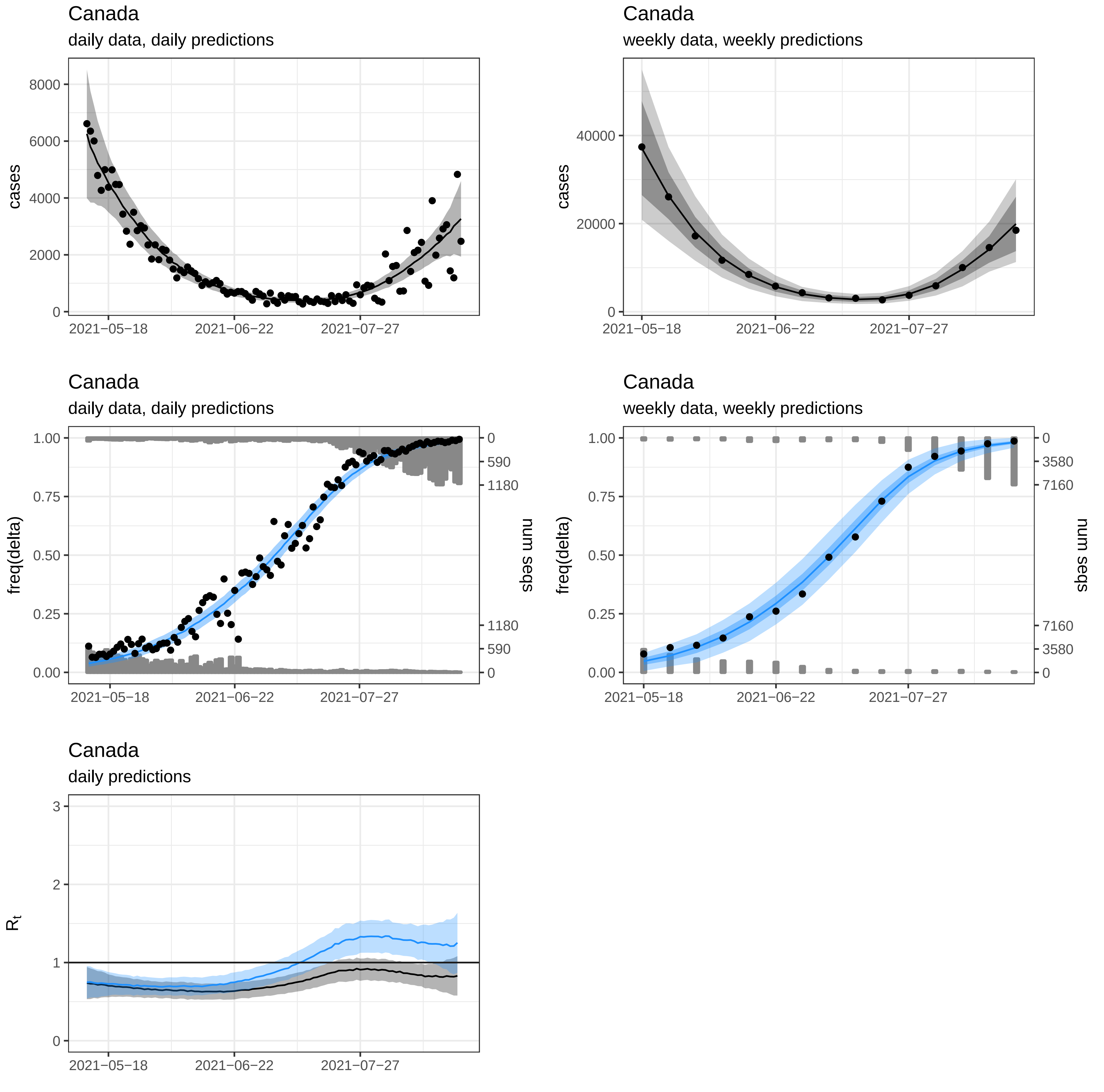

Canada
daily data, daily predictions
cases
8000
6000
4000
2000
0
2021−05−18
2021−06−22
2021−07−27
Canada
weekly data, weekly predictions
cases
40000
20000
0
2021−05−18
2021−06−22
2021−07−27
Canada
daily data, daily predictions
freq(delta)
1.00
0.75
0.50
0.25
0.00
num seqs
0
590
1180
1180
590
0
2021−05−18
2021−06−22
2021−07−27
Canada
weekly data, weekly predictions
freq(delta)
1.00
0.75
0.50
0.25
0.00
num seqs
0
3580
7160
7160
3580
0
2021−05−18
2021−06−22
2021−07−27
Canada
daily predictions
R_t
3
2
1
0
2021−05−18
2021−06−22
2021−07−27

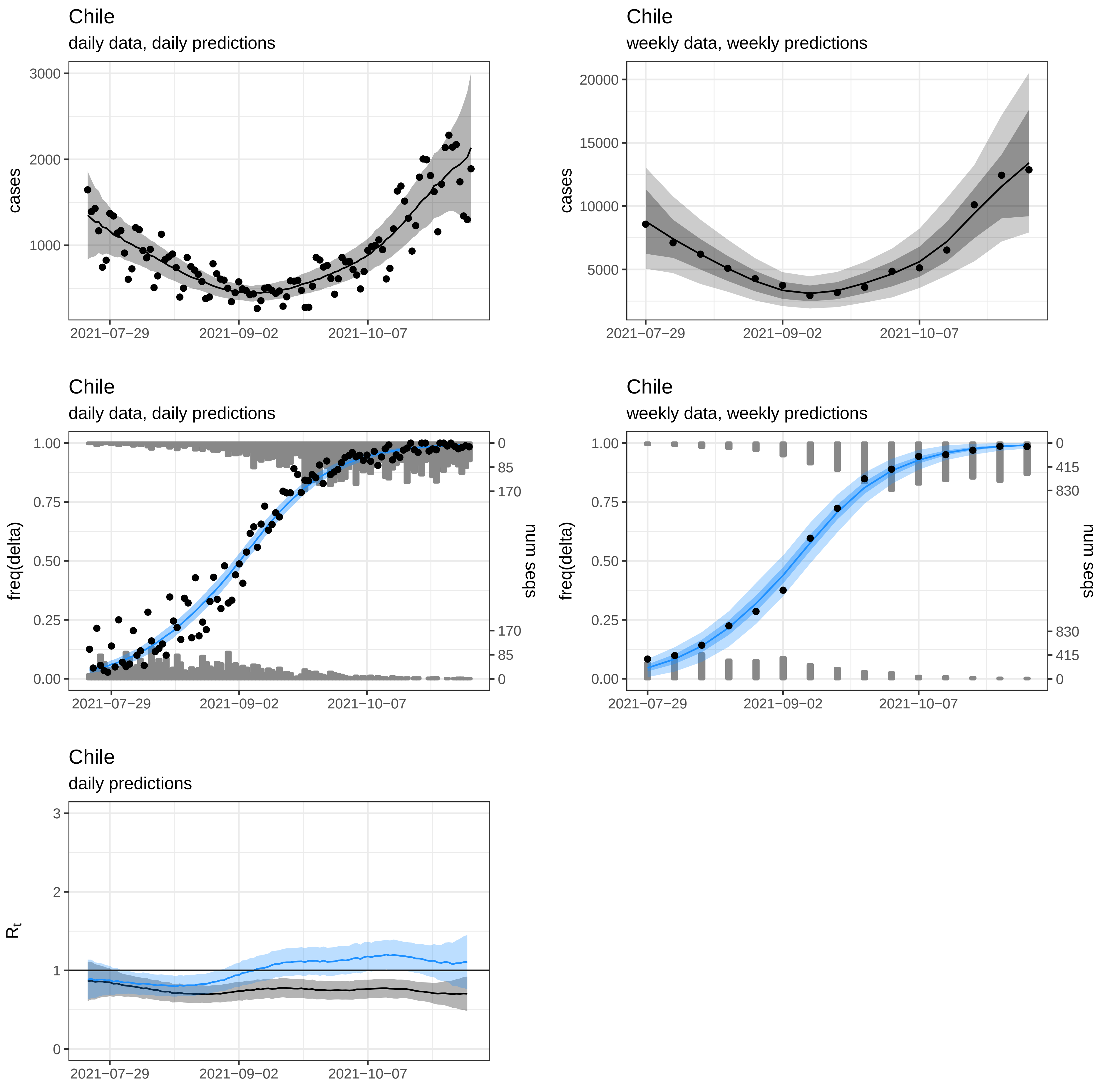
Chile
daily data, daily predictions
cases
3000
2000
1000
2021−07−29
2021−09−02
2021−10−07
Chile
weekly data, weekly predictions
cases
20000
15000
10000
5000
2021−07−29
2021−09−02
2021−10−07
Chile
daily data, daily predictions
freq(delta)
1.00
0.75
0.50
0.25
0.00
num seqs
0
85
170
170
85
0
2021−07−29
2021−09−02
2021−10−07
Chile
weekly data, weekly predictions
freq(delta)
1.00
0.75
0.50
0.25
0.00
num seqs
0
415
830
830
415
0
2021−07−29
2021−09−02
2021−10−07
Chile
daily predictions
R_t
3
2
1
0
2021−07−29
2021−09−02
2021−10−07

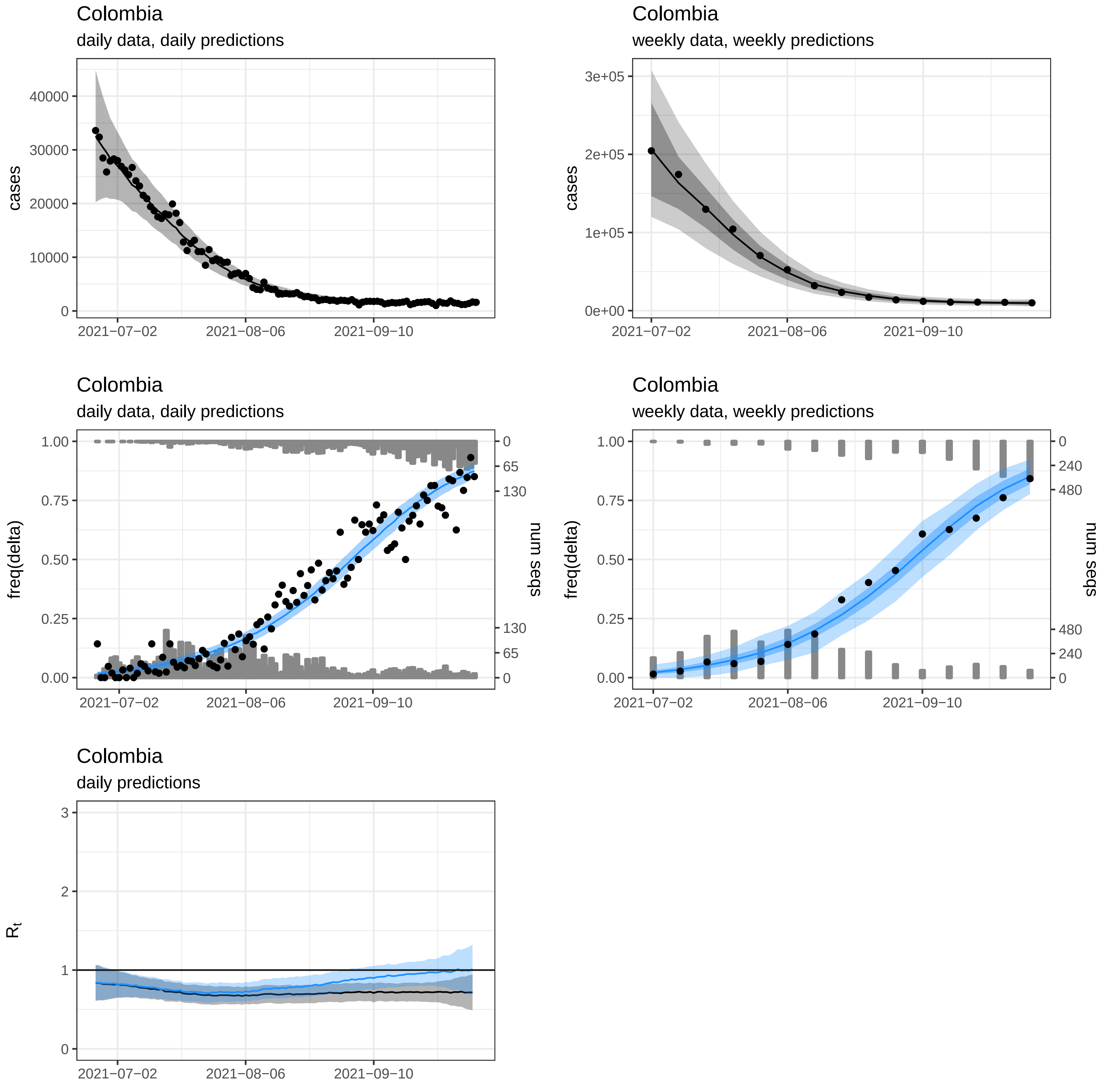
Colombia
daily data, daily predictions
cases
40000
30000
20000
10000
0
2021−07−02
2021−08−06
2021−09−10
Colombia
weekly data, weekly predictions
cases
3e+05
2e+05
1e+05
0e+00
2021−07−02
2021−08−06
2021−09−10
Colombia
daily data, daily predictions
freq(delta)
1.00
0.75
0.50
0.25
0.00
num seqs
0
65
130
130
65
0
2021−07−02
2021−08−06
2021−09−10
Colombia
weekly data, weekly predictions
freq(delta)
1.00
0.75
0.50
0.25
0.00
num seqs
0
240
480
480
240
0
2021−07−02
2021−08−06
2021−09−10
Colombia
daily predictions
R t
3
2
1
0
2021−07−02
2021−08−06
2021−09−10

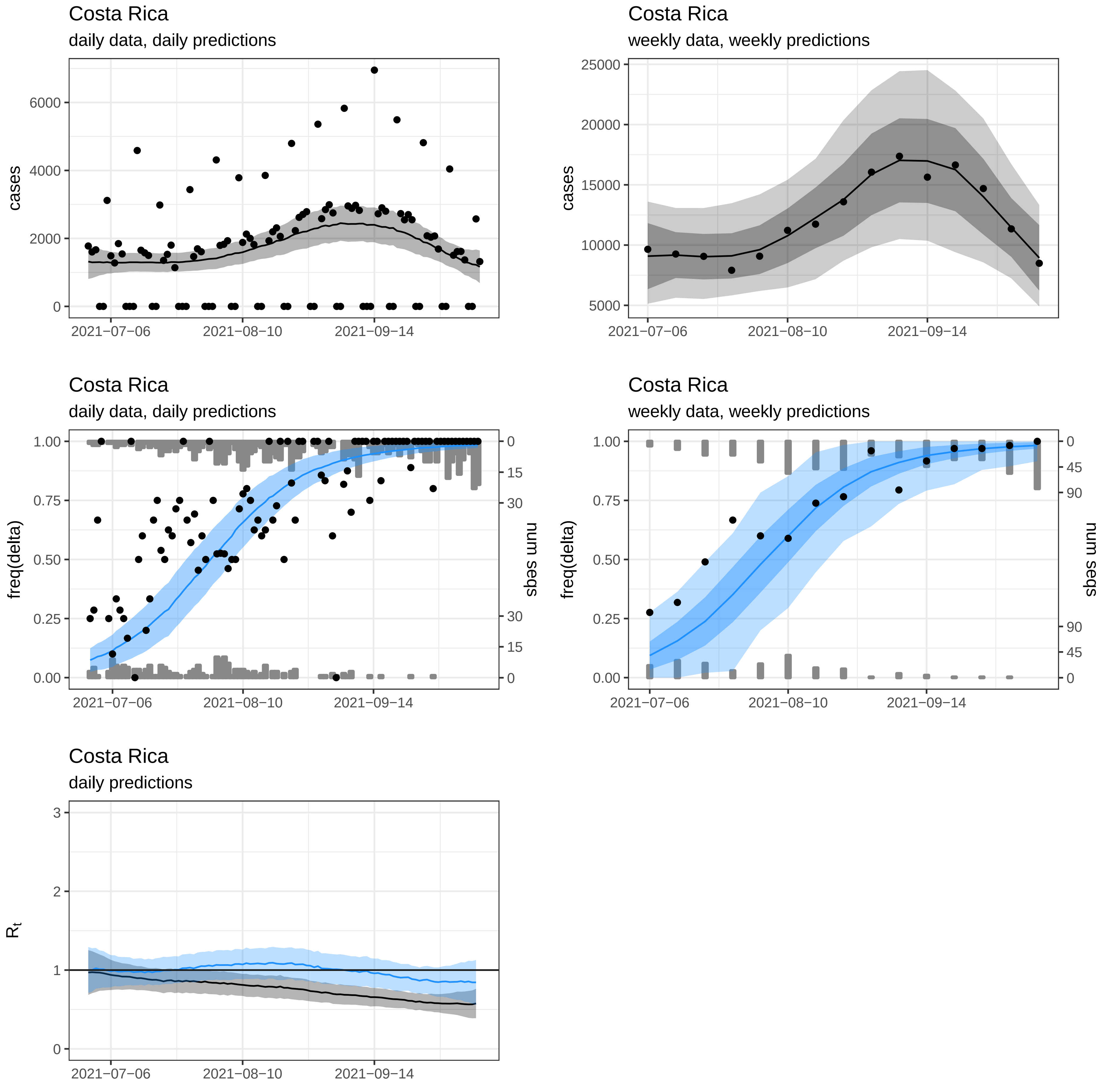
Costa Rica
daily data, daily predictions
cases
0
2000
4000
6000
2021−07−06
2021−08−10
2021−09−14
Costa Rica
weekly data, weekly predictions
cases
5000
10000
15000
20000
25000
2021−07−06
2021−08−10
2021−09−14
Costa Rica
daily data, daily predictions
freq(delta)
0.00
0.25
0.50
0.75
1.00
num seqs
0
15
30
30
15
0
2021−07−06
2021−08−10
2021−09−14
Costa Rica
weekly data, weekly predictions
freq(delta)
0.00
0.25
0.50
0.75
1.00
num seqs
0
45
90
90
45
0
2021−07−06
2021−08−10
2021−09−14
Costa Rica
daily predictions
R_t
0
1
2
3
2021−07−06
2021−08−10
2021−09−14

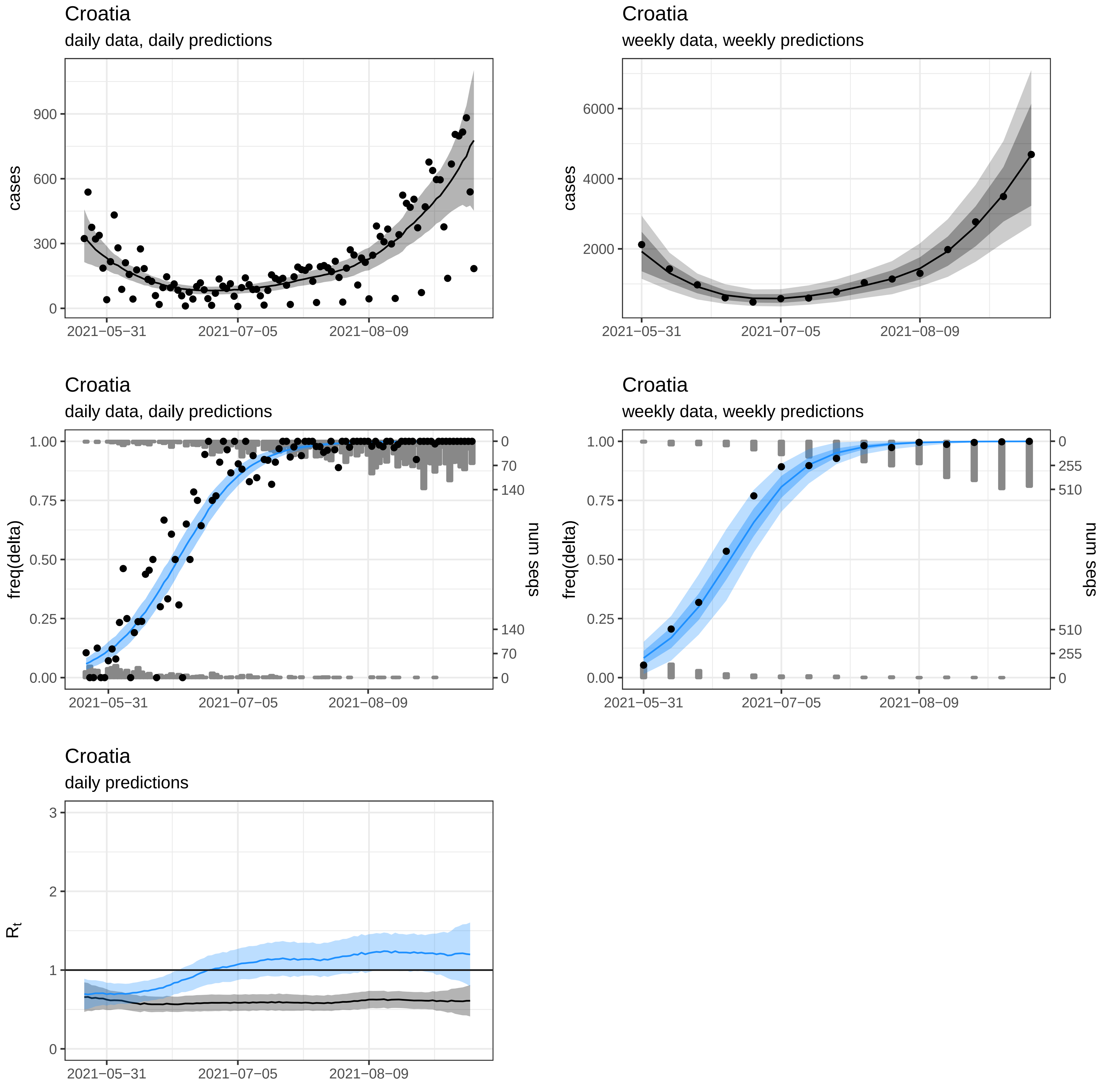
Croatia
daily data, daily predictions
cases
0
300
600
900
2021−05−31
2021−07−05
2021−08−09
Croatia
weekly data, weekly predictions
cases
2000
4000
6000
2021−05−31
2021−07−05
2021−08−09
Croatia
daily data, daily predictions
freq(delta)
0.00
0.25
0.50
0.75
1.00
num seqs
0
70
140
2021−05−31
2021−07−05
2021−08−09
Croatia
weekly data, weekly predictions
freq(delta)
0.00
0.25
0.50
0.75
1.00
num seqs
0
255
510
2021−05−31
2021−07−05
2021−08−09
Croatia
daily predictions
R_t
0
1
2
3
2021−05−31
2021−07−05
2021−08−09

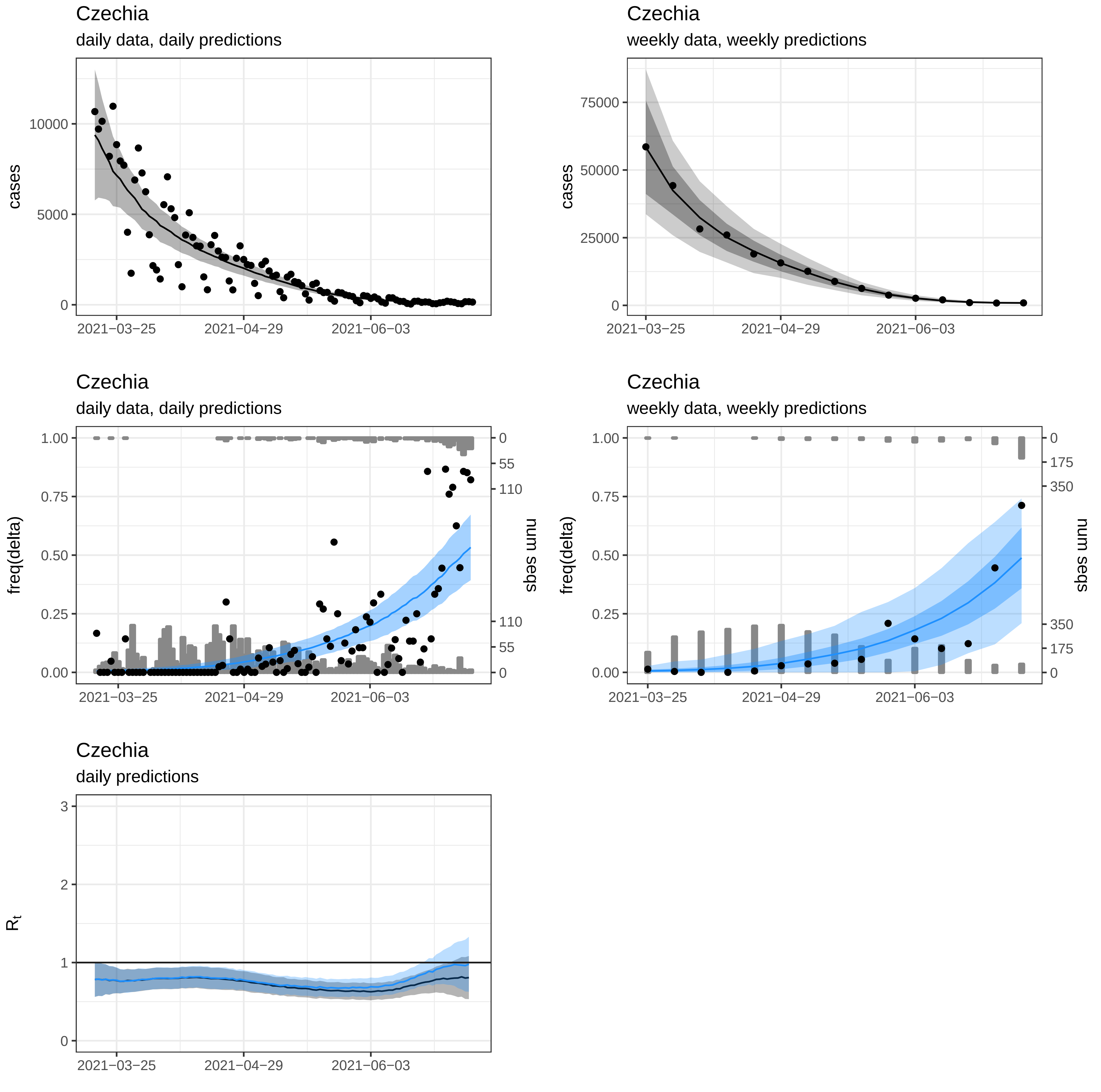
Czechia
daily data, daily predictions
cases
10000
5000
0
2021−03−25
2021−04−29
2021−06−03
Czechia
weekly data, weekly predictions
cases
75000
50000
25000
0
2021−03−25
2021−04−29
2021−06−03
Czechia
daily data, daily predictions
freq(delta)
1.00
0.75
0.50
0.25
0.00
num seqs
0
55
110
110
55
0
2021−03−25
2021−04−29
2021−06−03
Czechia
weekly data, weekly predictions
freq(delta)
1.00
0.75
0.50
0.25
0.00
num seqs
0
175
350
350
175
0
2021−03−25
2021−04−29
2021−06−03
Czechia
daily predictions
R_t
3
2
1
0
2021−03−25
2021−04−29
2021−06−03

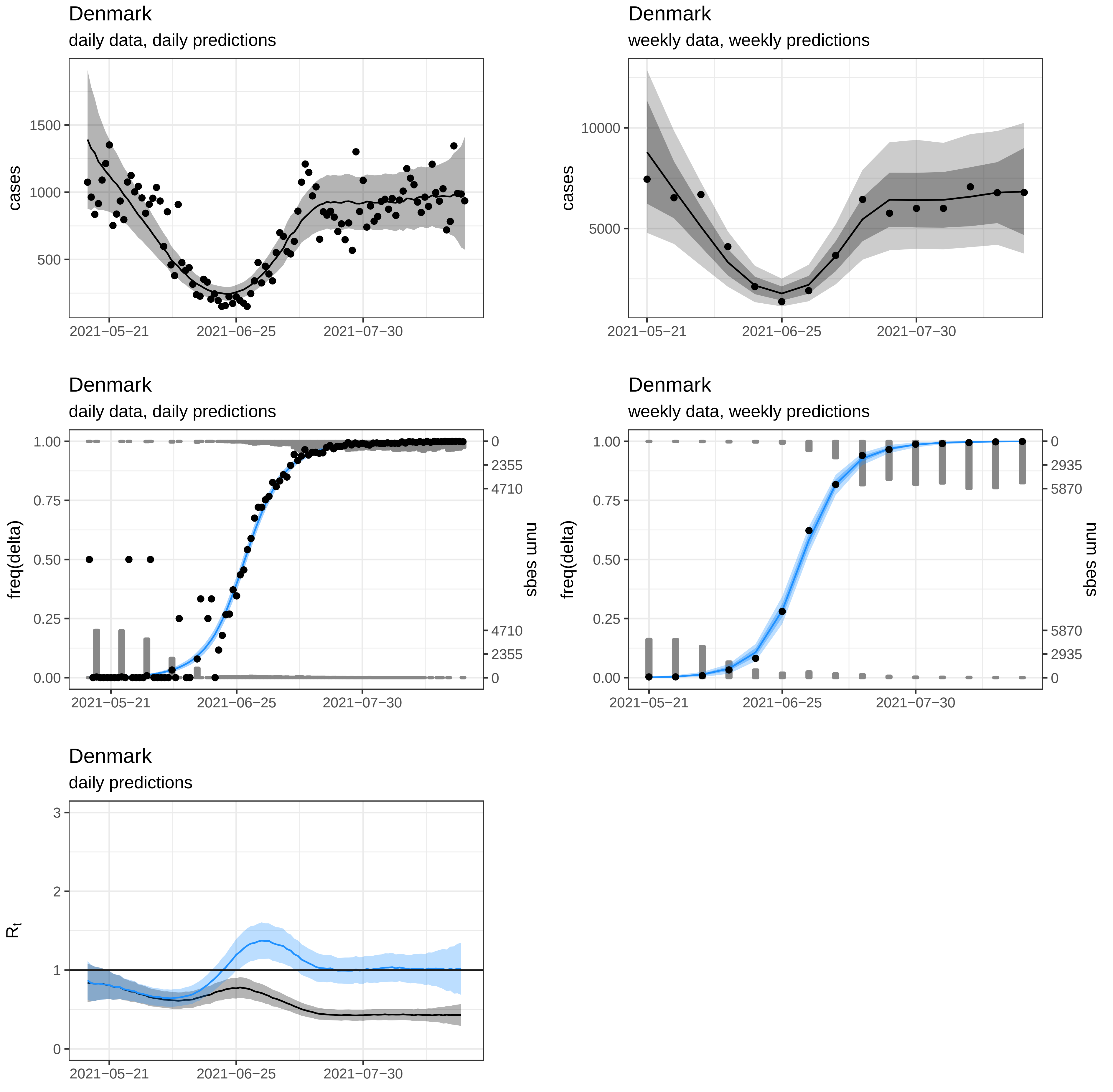
Denmark
daily data, daily predictions
cases
1500
1000
500
2021−05−21
2021−06−25
2021−07−30
Denmark
weekly data, weekly predictions
cases
10000
5000
2021−05−21
2021−06−25
2021−07−30
Denmark
daily data, daily predictions
freq(delta)
1.00
0.75
0.50
0.25
0.00
num seqs
0
2355
4710
4710
2355
0
2021−05−21
2021−06−25
2021−07−30
Denmark
weekly data, weekly predictions
freq(delta)
1.00
0.75
0.50
0.25
0.00
num seqs
0
2935
5870
5870
2935
0
2021−05−21
2021−06−25
2021−07−30
Denmark
daily predictions
R_t
3
2
1
0
2021−05−21
2021−06−25
2021−07−30

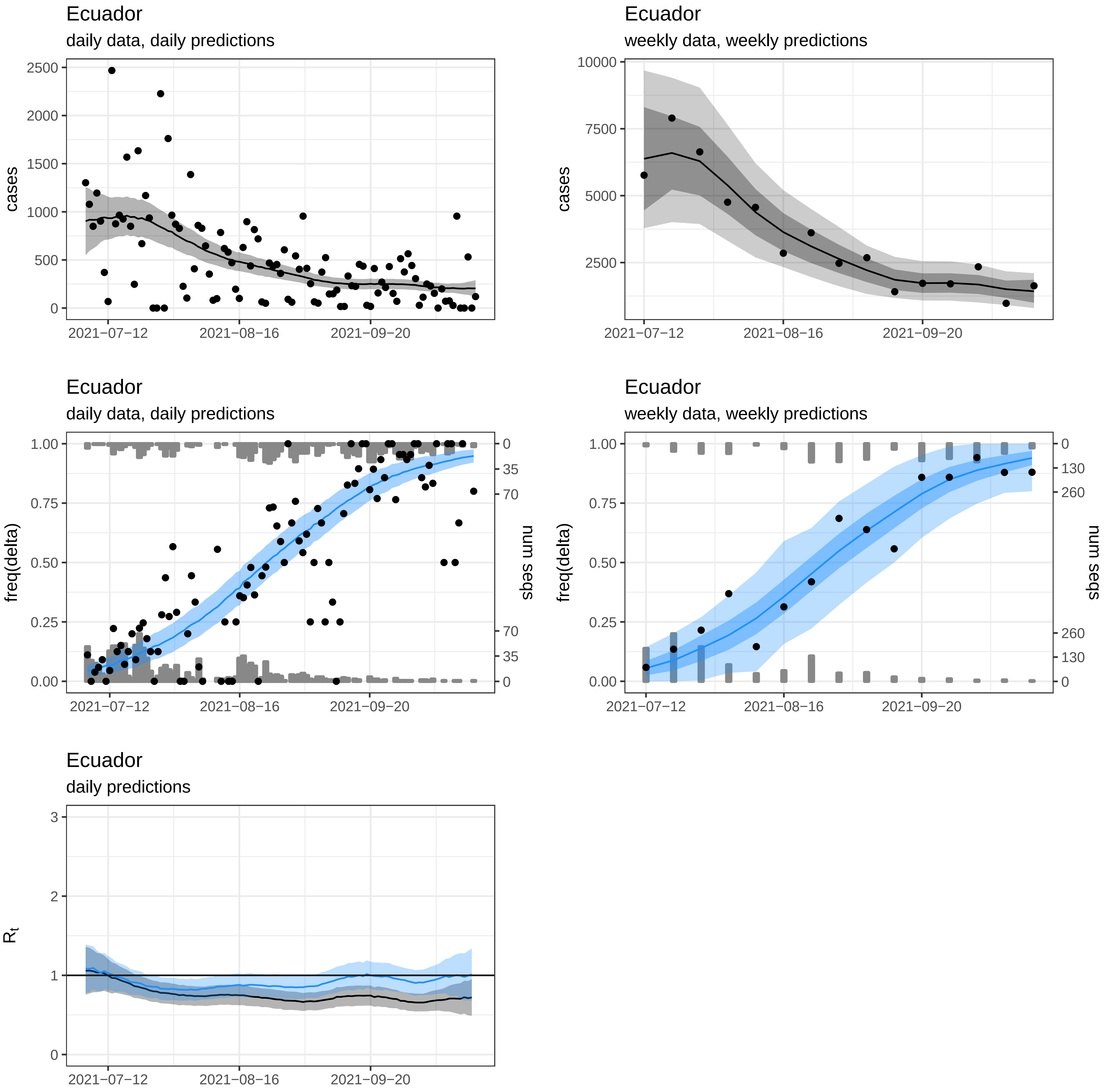
Ecuador
daily data, daily predictions
cases
2500
2000
1500
1000
500
0
2021−07−12
2021−08−16
2021−09−20
Ecuador
weekly data, weekly predictions
cases
10000
7500
5000
2500
2021−07−12
2021−08−16
2021−09−20
Ecuador
daily data, daily predictions
freq(delta)
1.00
0.75
0.50
0.25
0.00
num seqs
0
35
70
70
35
0
2021−07−12
2021−08−16
2021−09−20
Ecuador
weekly data, weekly predictions
freq(delta)
1.00
0.75
0.50
0.25
0.00
num seqs
0
130
260
260
130
0
2021−07−12
2021−08−16
2021−09−20
Ecuador
daily predictions
R_t
3
2
1
0
2021−07−12
2021−08−16
2021−09−20

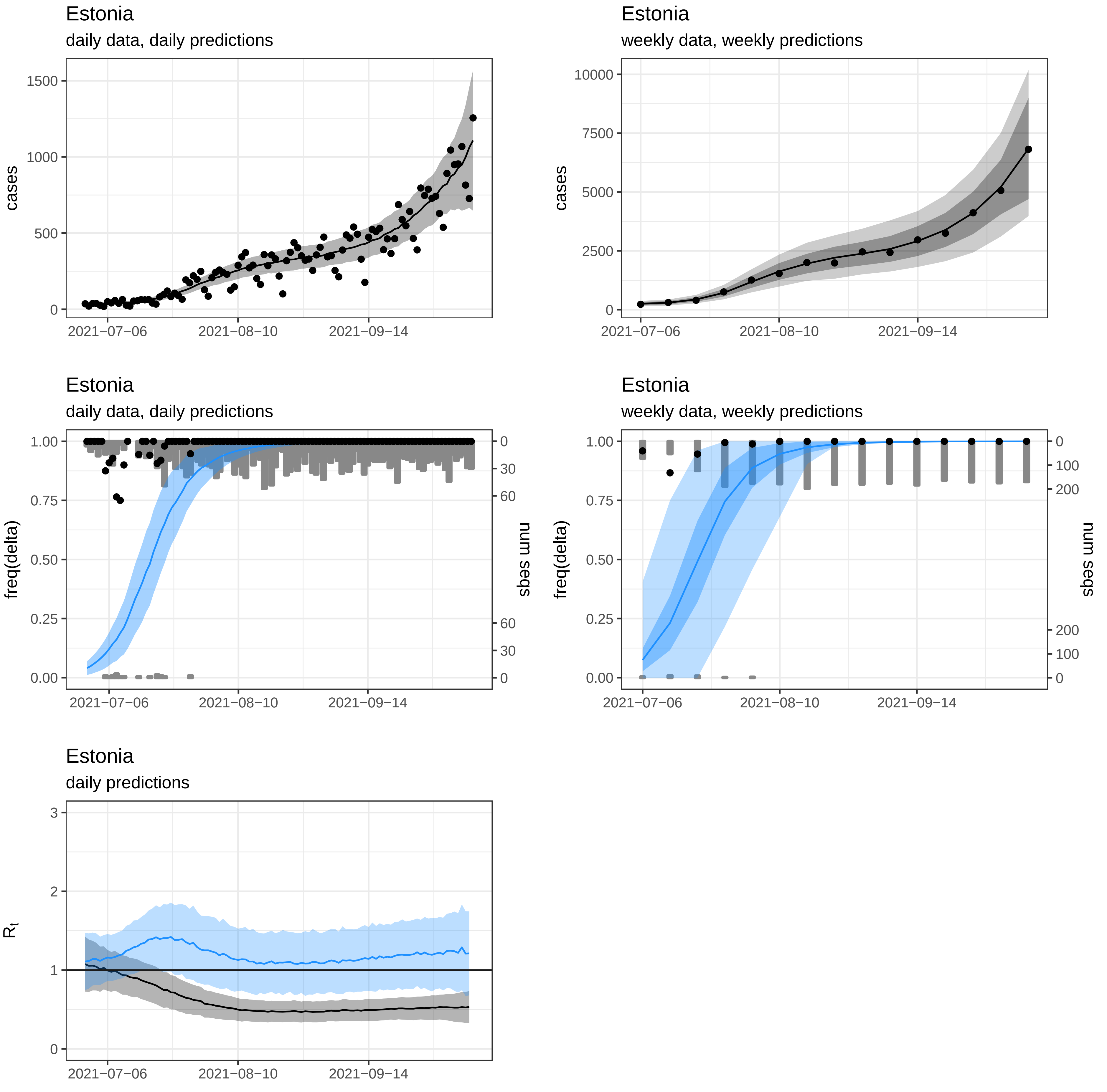
Estonia
daily data, daily predictions
cases
1500
1000
500
0
2021−07−06
2021−08−10
2021−09−14
Estonia
weekly data, weekly predictions
cases
10000
7500
5000
2500
0
2021−07−06
2021−08−10
2021−09−14
Estonia
daily data, daily predictions
freq(delta)
1.00
0.75
0.50
0.25
0.00
num seqs
0
30
60
60
30
0
2021−07−06
2021−08−10
2021−09−14
Estonia
weekly data, weekly predictions
freq(delta)
1.00
0.75
0.50
0.25
0.00
num seqs
0
100
200
200
100
0
2021−07−06
2021−08−10
2021−09−14
Estonia
daily predictions
R_t
3
2
1
0
2021−07−06
2021−08−10
2021−09−14

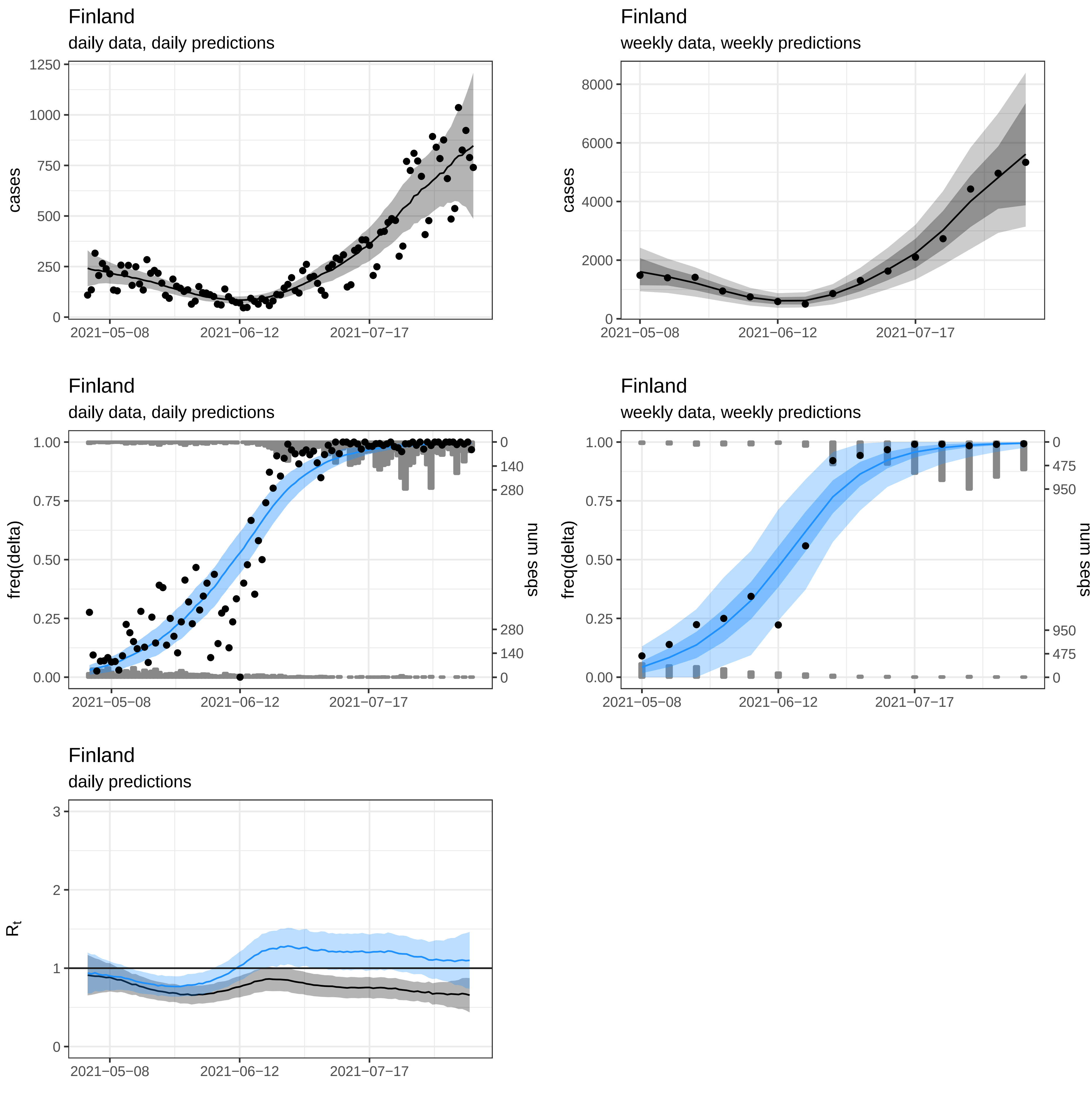

Finland
daily data, daily predictions
cases
1250
1000
750
500
250
0
2021−05−08
2021−06−12
2021−07−17
Finland
weekly data, weekly predictions
cases
8000
6000
4000
2000
0
2021−05−08
2021−06−12
2021−07−17
Finland
daily data, daily predictions
freq(delta)
1.00
0.75
0.50
0.25
0.00
num seqs
0
140
280
280
140
0
2021−05−08
2021−06−12
2021−07−17
Finland
weekly data, weekly predictions
freq(delta)
1.00
0.75
0.50
0.25
0.00
num seqs
0
475
950
950
475
0
2021−05−08
2021−06−12
2021−07−17
Finland
daily predictions
R_t
3
2
1
0
2021−05−08
2021−06−12
2021−07−17

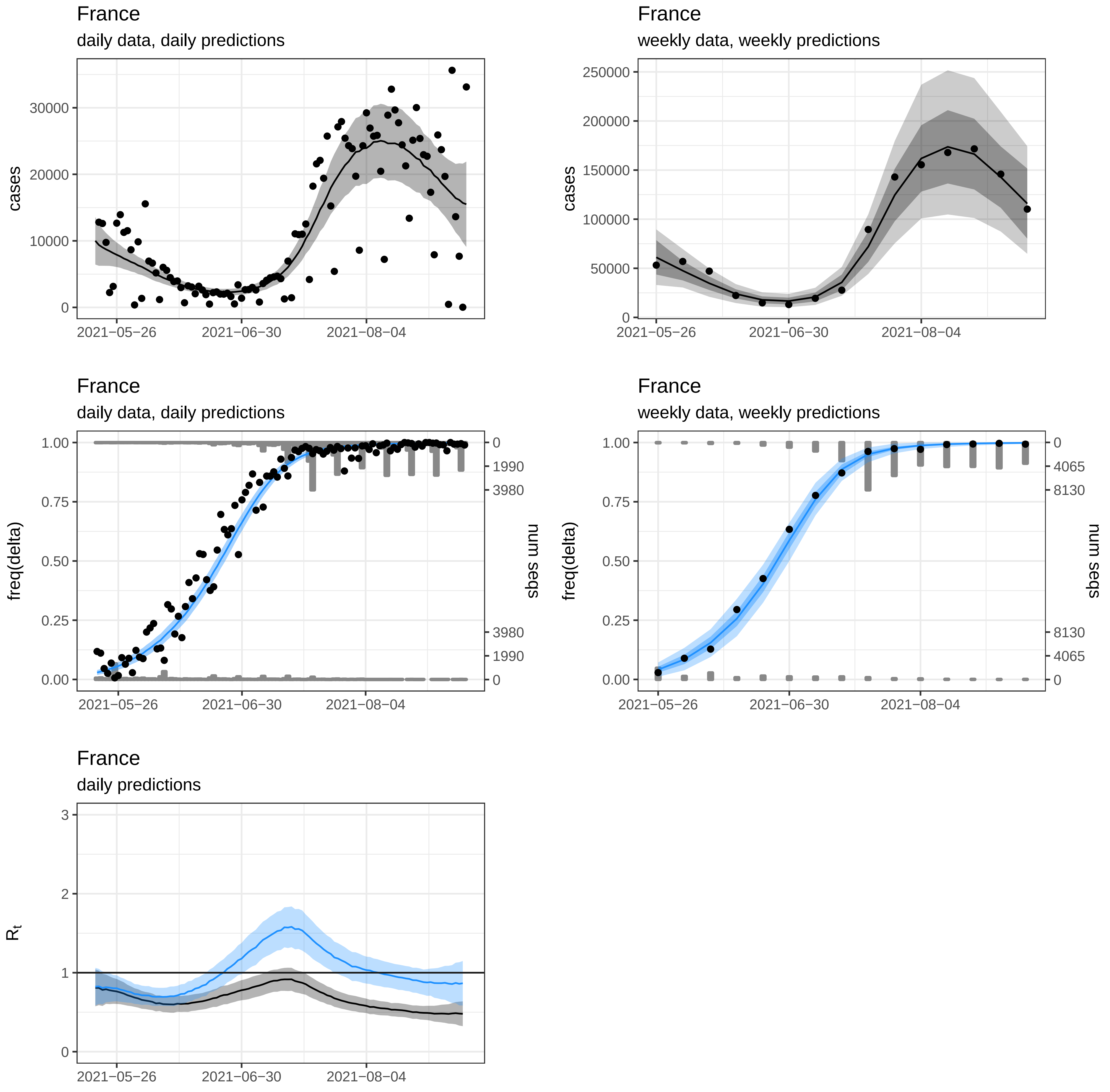
France
daily data, daily predictions
cases
30000
20000
10000
0
2021−05−26
2021−06−30
2021−08−04
France
weekly data, weekly predictions
cases
250000
200000
150000
100000
50000
0
2021−05−26
2021−06−30
2021−08−04
France
daily data, daily predictions
freq(delta)
1.00
0.75
0.50
0.25
0.00
num seqs
0
1990
3980
3980
1990
0
2021−05−26
2021−06−30
2021−08−04
France
weekly data, weekly predictions
freq(delta)
1.00
0.75
0.50
0.25
0.00
num seqs
0
4065
8130
8130
4065
0
2021−05−26
2021−06−30
2021−08−04
France
daily predictions
R_t
3
2
1
0
2021−05−26
2021−06−30
2021−08−04

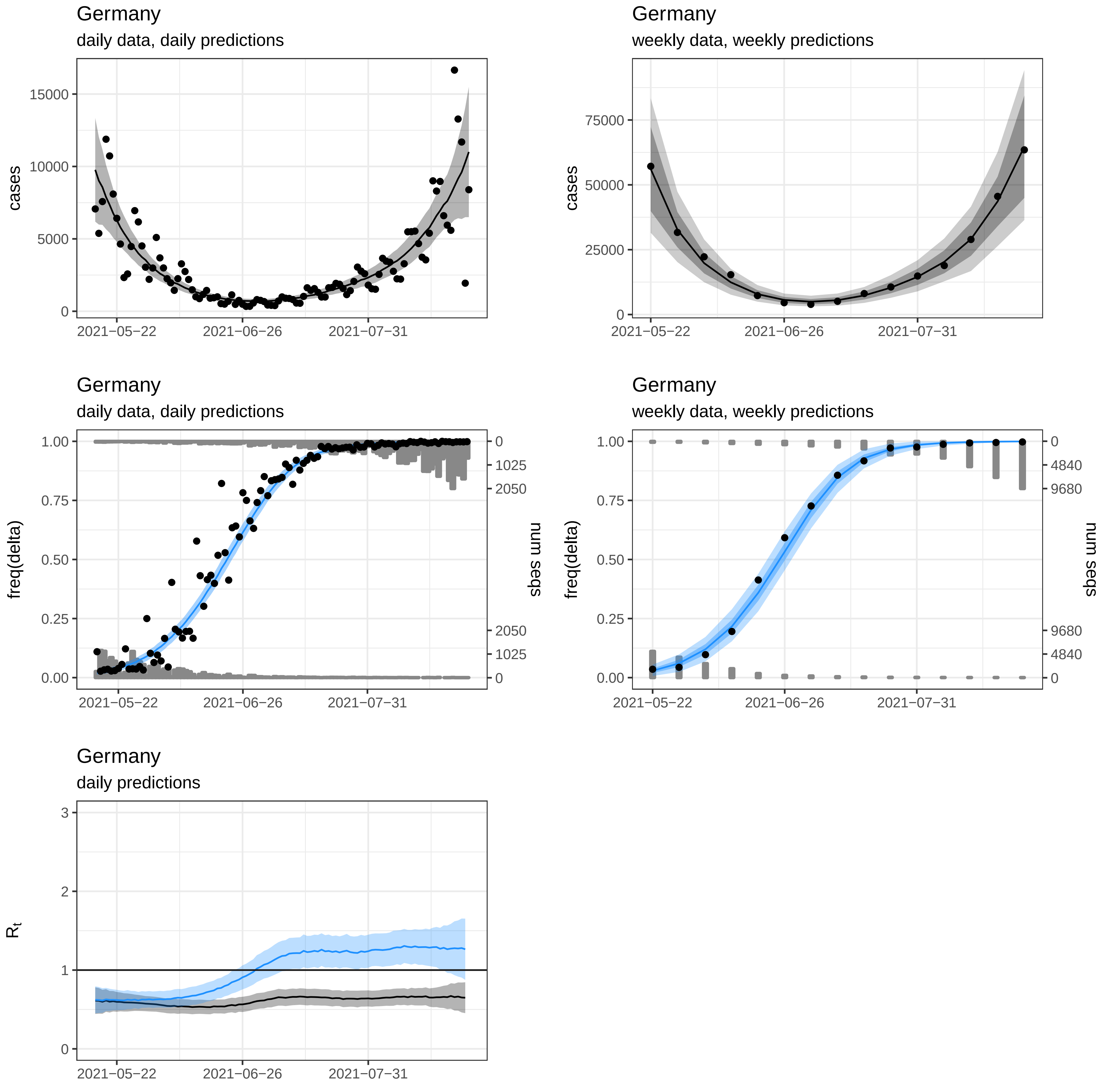

Germany
daily data, daily predictions
cases
15000
10000
5000
0
2021−05−22
2021−06−26
2021−07−31
Germany
weekly data, weekly predictions
cases
75000
50000
25000
0
2021−05−22
2021−06−26
2021−07−31
Germany
daily data, daily predictions
freq(delta)
1.00
0.75
0.50
0.25
0.00
num seqs
0
1025
2050
2050
1025
0
2021−05−22
2021−06−26
2021−07−31
Germany
weekly data, weekly predictions
freq(delta)
1.00
0.75
0.50
0.25
0.00
num seqs
0
4840
9680
9680
4840
0
2021−05−22
2021−06−26
2021−07−31
Germany
daily predictions
R_t
3
2
1
0
2021−05−22
2021−06−26
2021−07−31

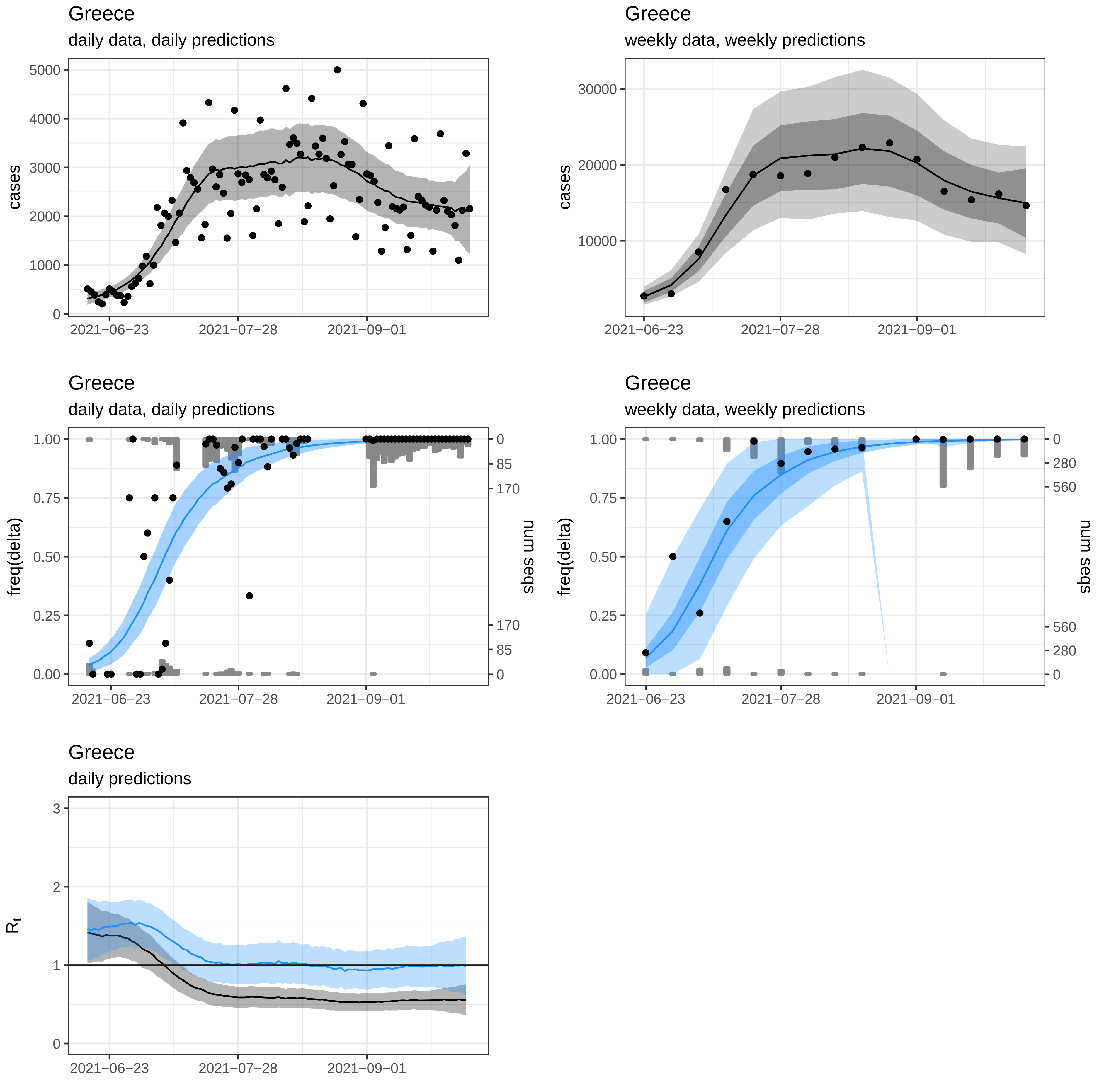

Greece
daily data, daily predictions
cases
5000
4000
3000
2000
1000
0
2021−06−23
2021−07−28
2021−09−01
Greece
weekly data, weekly predictions
cases
30000
20000
10000
2021−06−23
2021−07−28
2021−09−01
Greece
daily data, daily predictions
freq(delta)
1.00
0.75
0.50
0.25
0.00
num seqs
0
85
170
170
85
0
2021−06−23
2021−07−28
2021−09−01
Greece
weekly data, weekly predictions
freq(delta)
1.00
0.75
0.50
0.25
0.00
num seqs
0
280
560
560
280
0
2021−06−23
2021−07−28
2021−09−01
Greece
daily predictions
R_t
3
2
1
0
2021−06−23
2021−07−28
2021−09−01

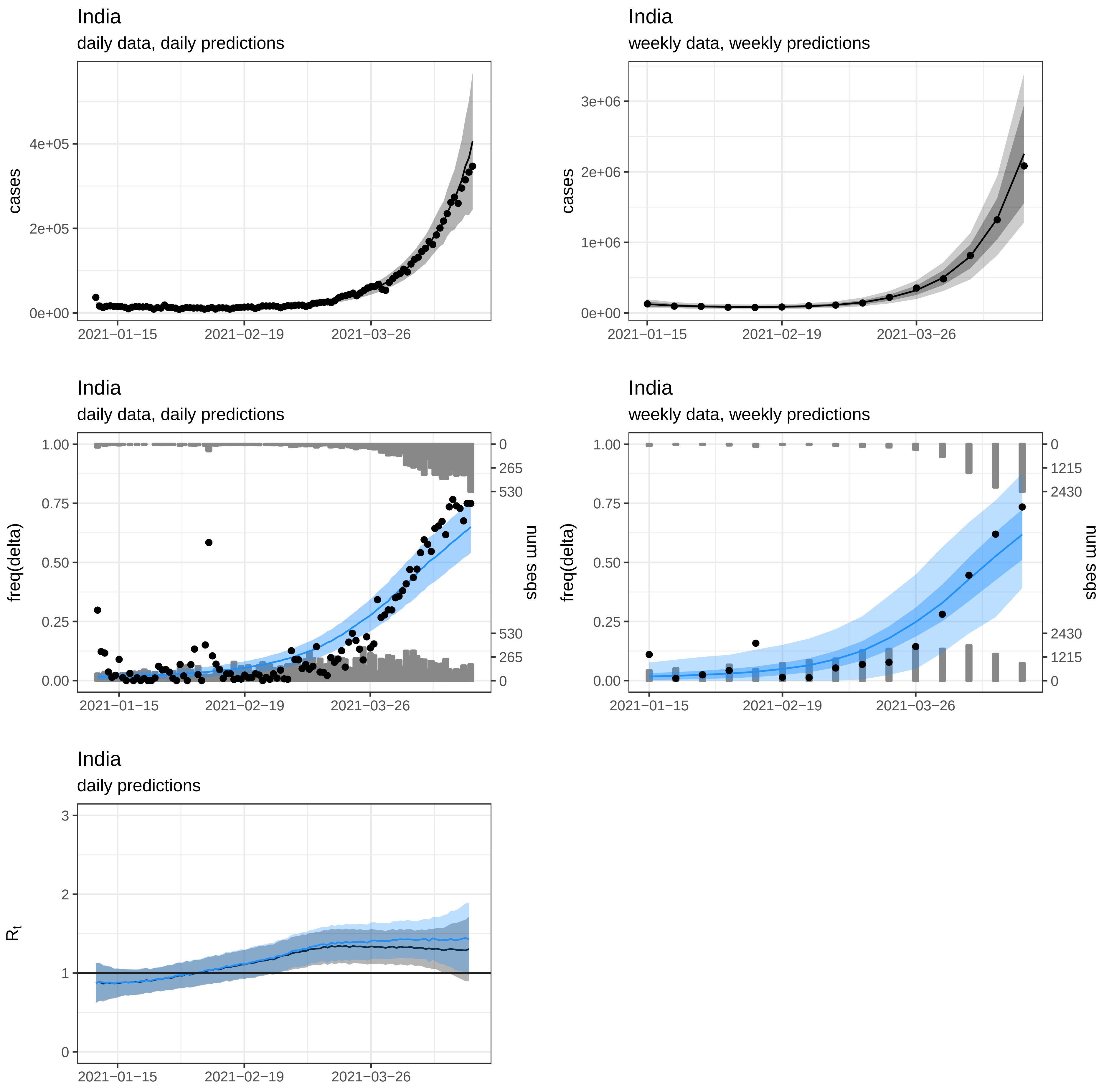

India
daily data, daily predictions
cases
4e+05
2e+05
0e+00
2021−01−15
2021−02−19
2021−03−26
India
weekly data, weekly predictions
cases
3e+06
2e+06
1e+06
0e+00
2021−01−15
2021−02−19
2021−03−26
India
daily data, daily predictions
freq(delta)
1.00
0.75
0.50
0.25
0.00
num seqs
0
265
530
530
265
0
2021−01−15
2021−02−19
2021−03−26
India
weekly data, weekly predictions
freq(delta)
1.00
0.75
0.50
0.25
0.00
num seqs
0
1215
2430
2430
1215
0
2021−01−15
2021−02−19
2021−03−26
India
daily predictions
R_t
3
2
1
0
2021−01−15
2021−02−19
2021−03−26

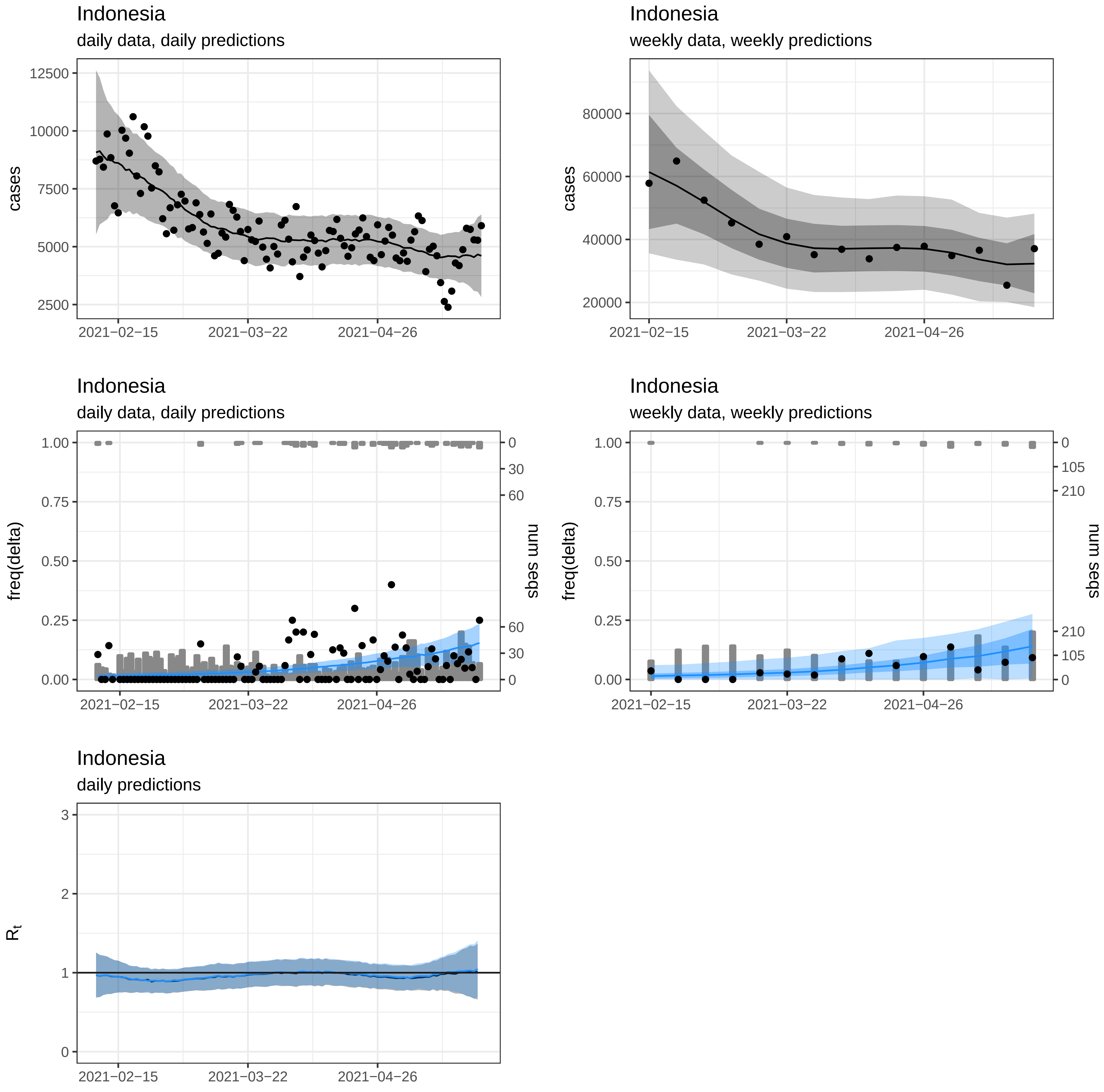
Indonesia
daily data, daily predictions
cases
12500
10000
7500
5000
2500
2021−02−15
2021−03−22
2021−04−26
Indonesia
weekly data, weekly predictions
cases
80000
60000
40000
20000
2021−02−15
2021−03−22
2021−04−26
Indonesia
daily data, daily predictions
freq(delta)
1.00
0.75
0.50
0.25
0.00
num seqs
0
30
60
60
30
0
2021−02−15
2021−03−22
2021−04−26
Indonesia
weekly data, weekly predictions
freq(delta)
1.00
0.75
0.50
0.25
0.00
num seqs
0
105
210
210
105
0
2021−02−15
2021−03−22
2021−04−26
Indonesia
daily predictions
R_t
3
2
1
0
2021−02−15
2021−03−22
2021−04−26

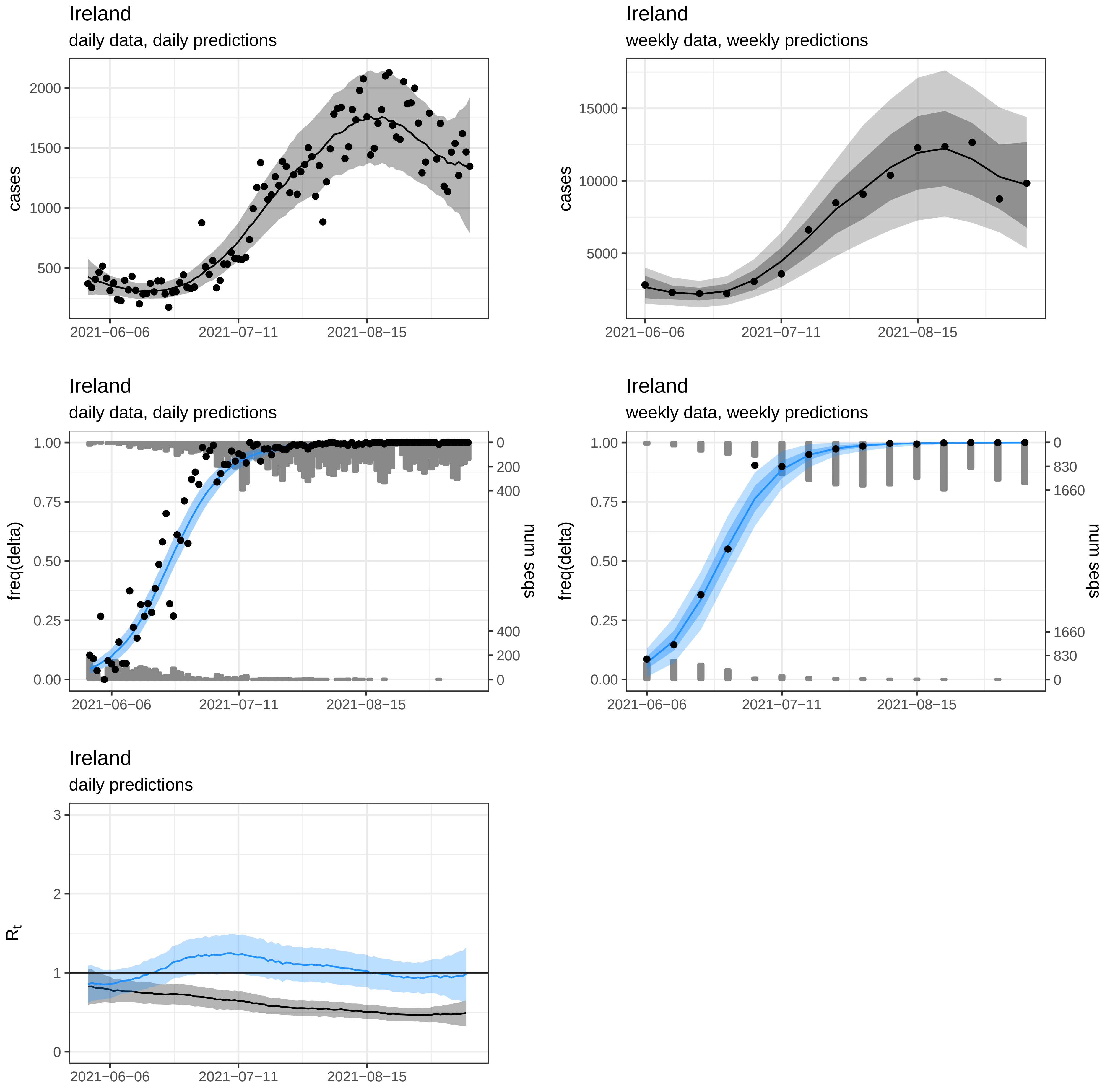
Ireland
daily data, daily predictions
cases
2000
1500
1000
500
2021−06−06
2021−07−11
2021−08−15
Ireland
weekly data, weekly predictions
cases
15000
10000
5000
2021−06−06
2021−07−11
2021−08−15
Ireland
daily data, daily predictions
freq(delta)
1.00
0.75
0.50
0.25
0.00
num seqs
0
200
400
400
200
0
2021−06−06
2021−07−11
2021−08−15
Ireland
weekly data, weekly predictions
freq(delta)
1.00
0.75
0.50
0.25
0.00
num seqs
0
830
1660
1660
830
0
2021−06−06
2021−07−11
2021−08−15
Ireland
daily predictions
R_t
3
2
1
0
2021−06−06
2021−07−11
2021−08−15

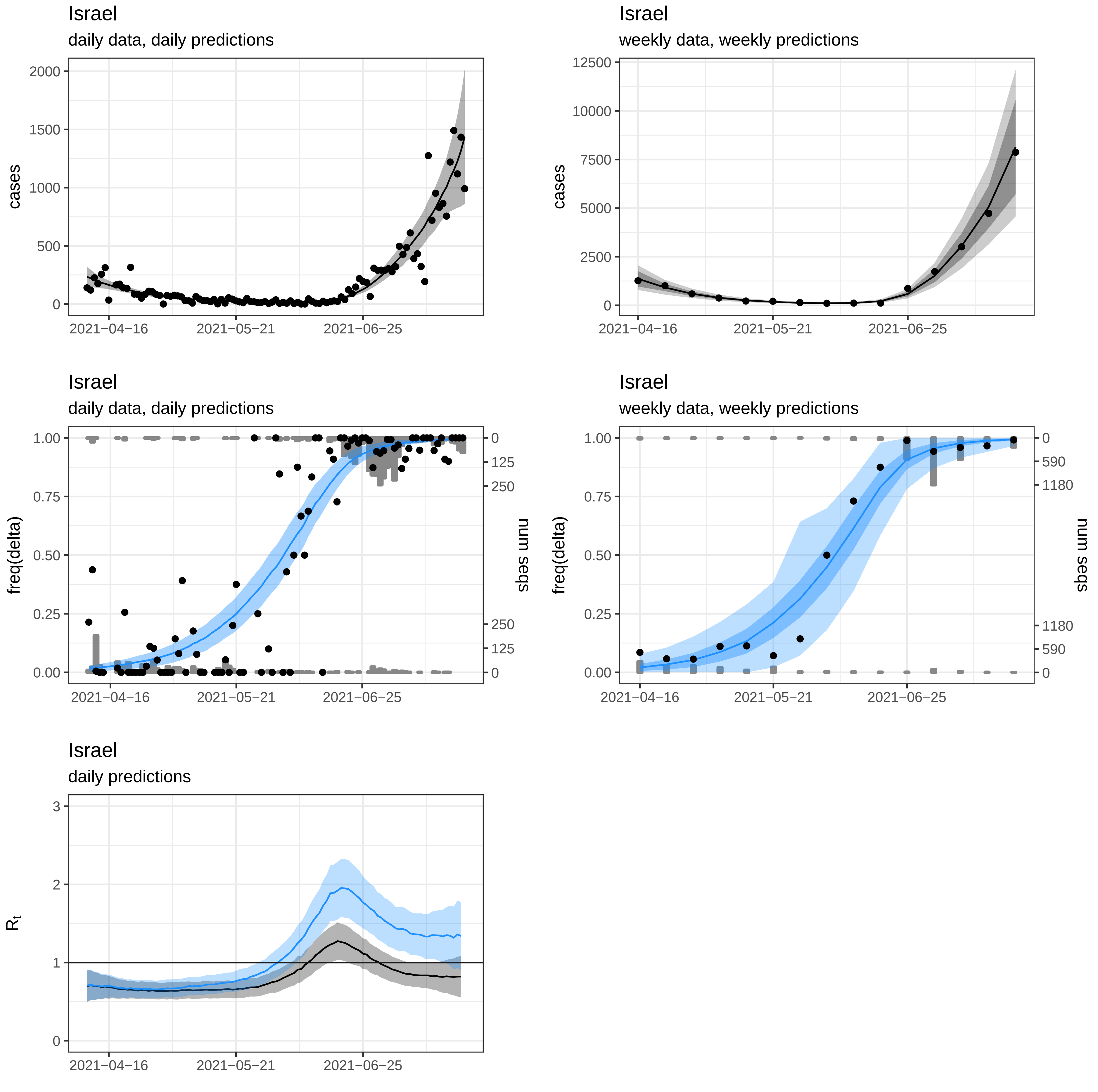
Israel
daily data, daily predictions
cases
2000
1500
1000
500
0
2021−04−16
2021−05−21
2021−06−25
Israel
weekly data, weekly predictions
cases
12500
10000
7500
5000
2500
0
2021−04−16
2021−05−21
2021−06−25
Israel
daily data, daily predictions
freq(delta)
1.00
0.75
0.50
0.25
0.00
num seqs
0
125
250
250
125
0
2021−04−16
2021−05−21
2021−06−25
Israel
weekly data, weekly predictions
freq(delta)
1.00
0.75
0.50
0.25
0.00
num seqs
0
590
1180
1180
590
0
2021−04−16
2021−05−21
2021−06−25
Israel
daily predictions
R_t
3
2
1
0
2021−04−16
2021−05−21
2021−06−25

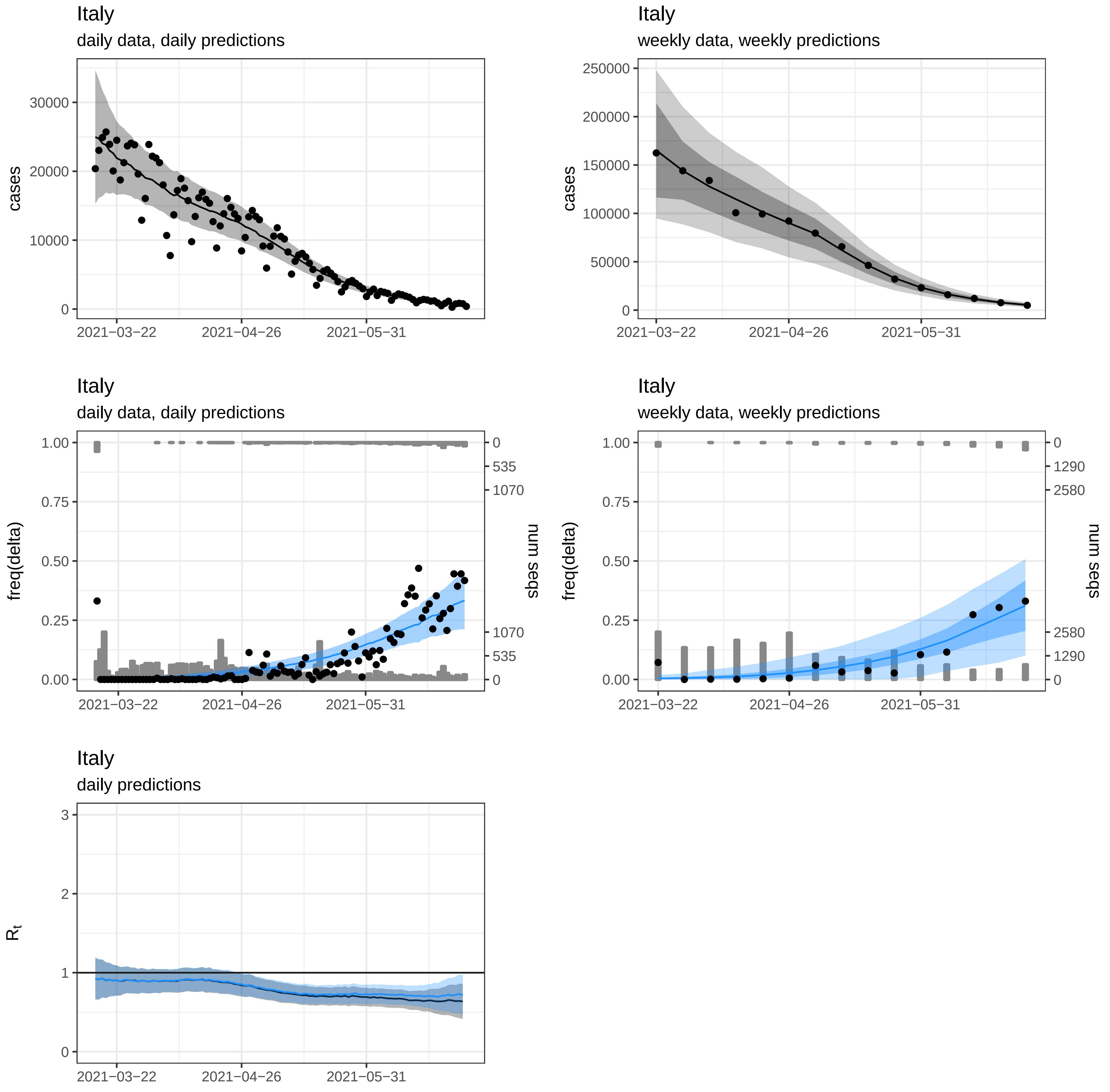

Italy
daily data, daily predictions
cases
30000
20000
10000
0
2021−03−22
2021−04−26
2021−05−31
Italy
weekly data, weekly predictions
cases
250000
200000
150000
100000
50000
0
2021−03−22
2021−04−26
2021−05−31
Italy
daily data, daily predictions
freq(delta)
1.00
0.75
0.50
0.25
0.00
num seqs
0
535
1070
1070
535
0
2021−03−22
2021−04−26
2021−05−31
Italy
weekly data, weekly predictions
freq(delta)
1.00
0.75
0.50
0.25
0.00
num seqs
0
1290
2580
2580
1290
0
2021−03−22
2021−04−26
2021−05−31
Italy
daily predictions
R_t
3
2
1
0
2021−03−22
2021−04−26
2021−05−31

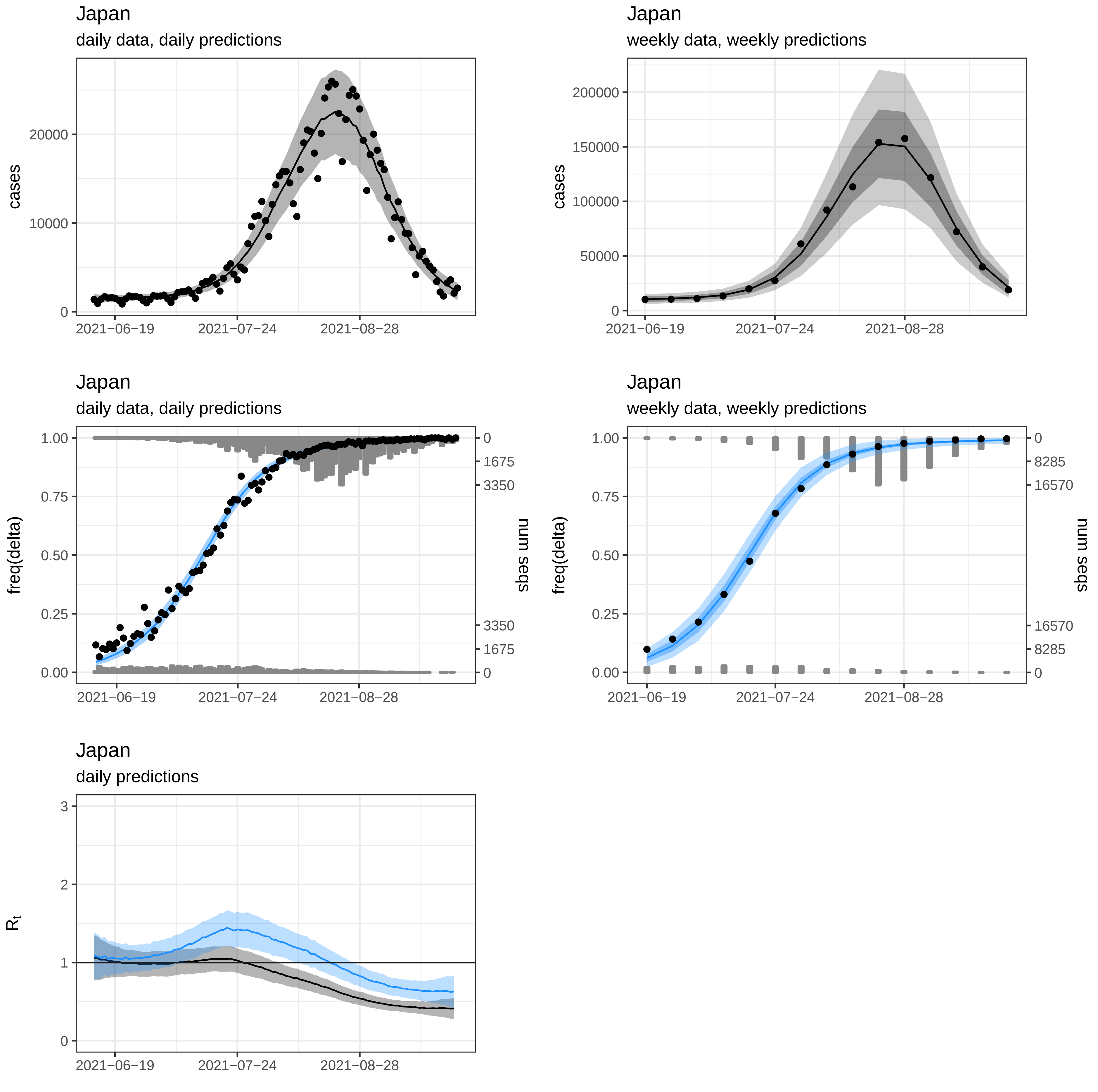

Japan
daily data, daily predictions
cases
20000
10000
0
2021−06−19
2021−07−24
2021−08−28
Japan
weekly data, weekly predictions
cases
200000
150000
100000
50000
0
2021−06−19
2021−07−24
2021−08−28
Japan
daily data, daily predictions
freq(delta)
1.00
0.75
0.50
0.25
0.00
num seqs
0
1675
3350
3350
1675
0
2021−06−19
2021−07−24
2021−08−28
Japan
weekly data, weekly predictions
freq(delta)
1.00
0.75
0.50
0.25
0.00
num seqs
0
8285
16570
16570
8285
0
2021−06−19
2021−07−24
2021−08−28
Japan
daily predictions
R_t
3
2
1
0
2021−06−19
2021−07−24
2021−08−28

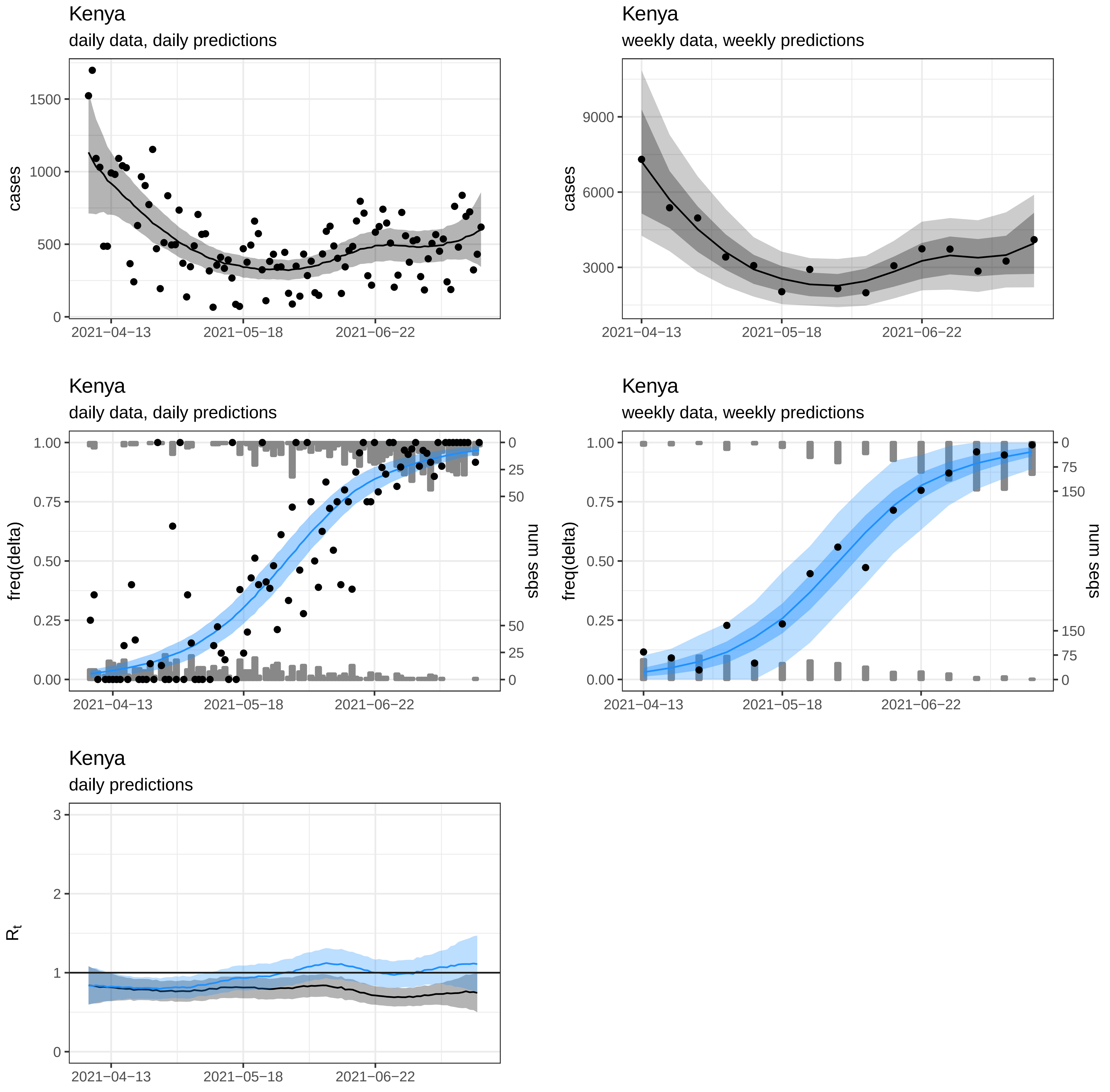

Kenya
daily data, daily predictions
cases
1500
1000
500
0
2021−04−13
2021−05−18
2021−06−22
Kenya
weekly data, weekly predictions
cases
9000
6000
3000
2021−04−13
2021−05−18
2021−06−22
Kenya
daily data, daily predictions
freq(delta)
1.00
0.75
0.50
0.25
0.00
num seqs
0
25
50
50
25
0
2021−04−13
2021−05−18
2021−06−22
Kenya
weekly data, weekly predictions
freq(delta)
1.00
0.75
0.50
0.25
0.00
num seqs
0
75
150
150
75
0
2021−04−13
2021−05−18
2021−06−22
Kenya
daily predictions
R_t
3
2
1
0
2021−04−13
2021−05−18
2021−06−22

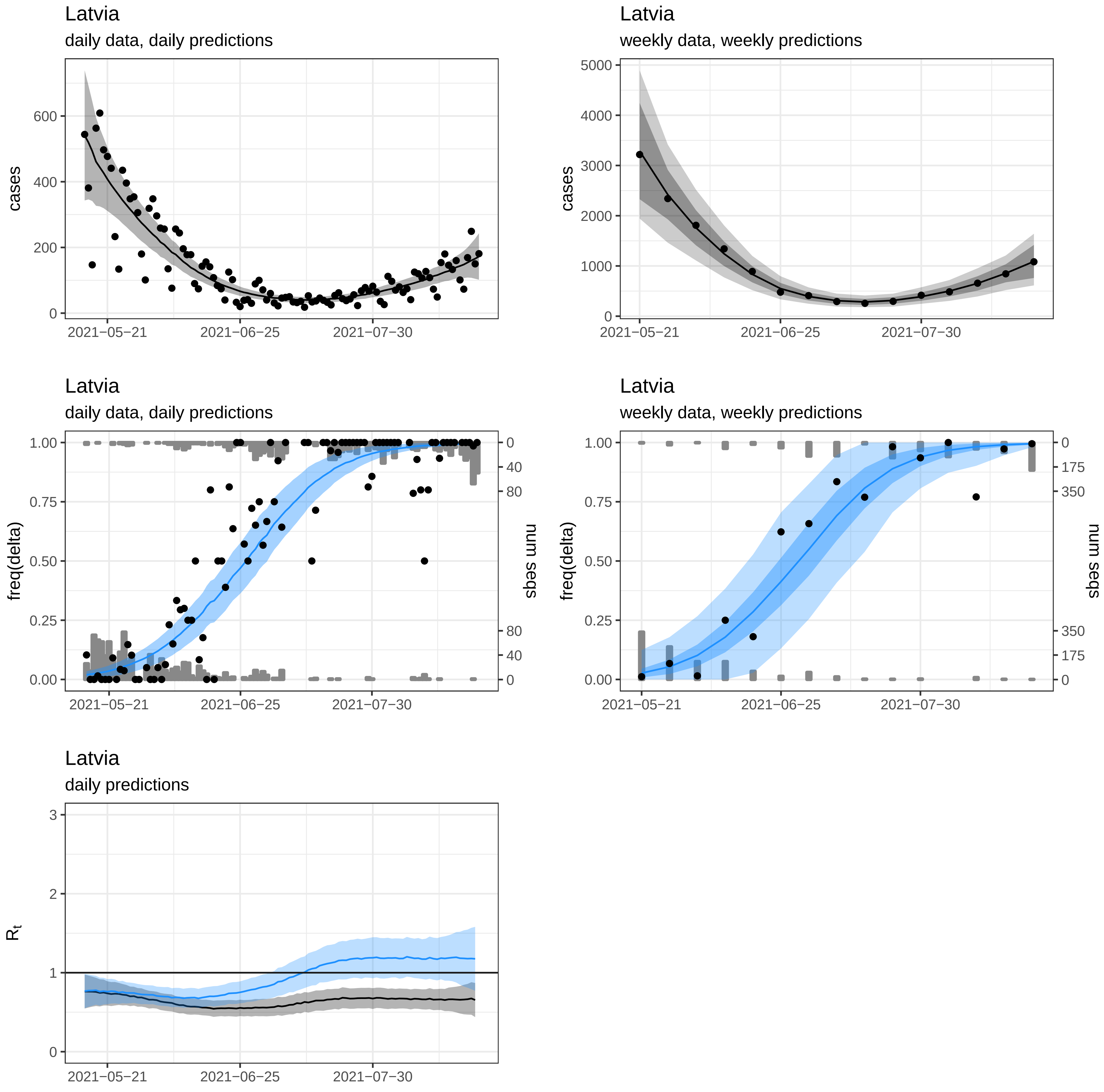
Latvia
daily data, daily predictions
cases
600
400
200
0
2021−05−21
2021−06−25
2021−07−30
Latvia
weekly data, weekly predictions
cases
5000
4000
3000
2000
1000
0
2021−05−21
2021−06−25
2021−07−30
Latvia
daily data, daily predictions
freq(delta)
1.00
0.75
0.50
0.25
0.00
num seqs
0
40
80
80
40
0
2021−05−21
2021−06−25
2021−07−30
Latvia
weekly data, weekly predictions
freq(delta)
1.00
0.75
0.50
0.25
0.00
num seqs
0
175
350
350
175
0
2021−05−21
2021−06−25
2021−07−30
Latvia
daily predictions
R_t
3
2
1
0
2021−05−21
2021−06−25
2021−07−30

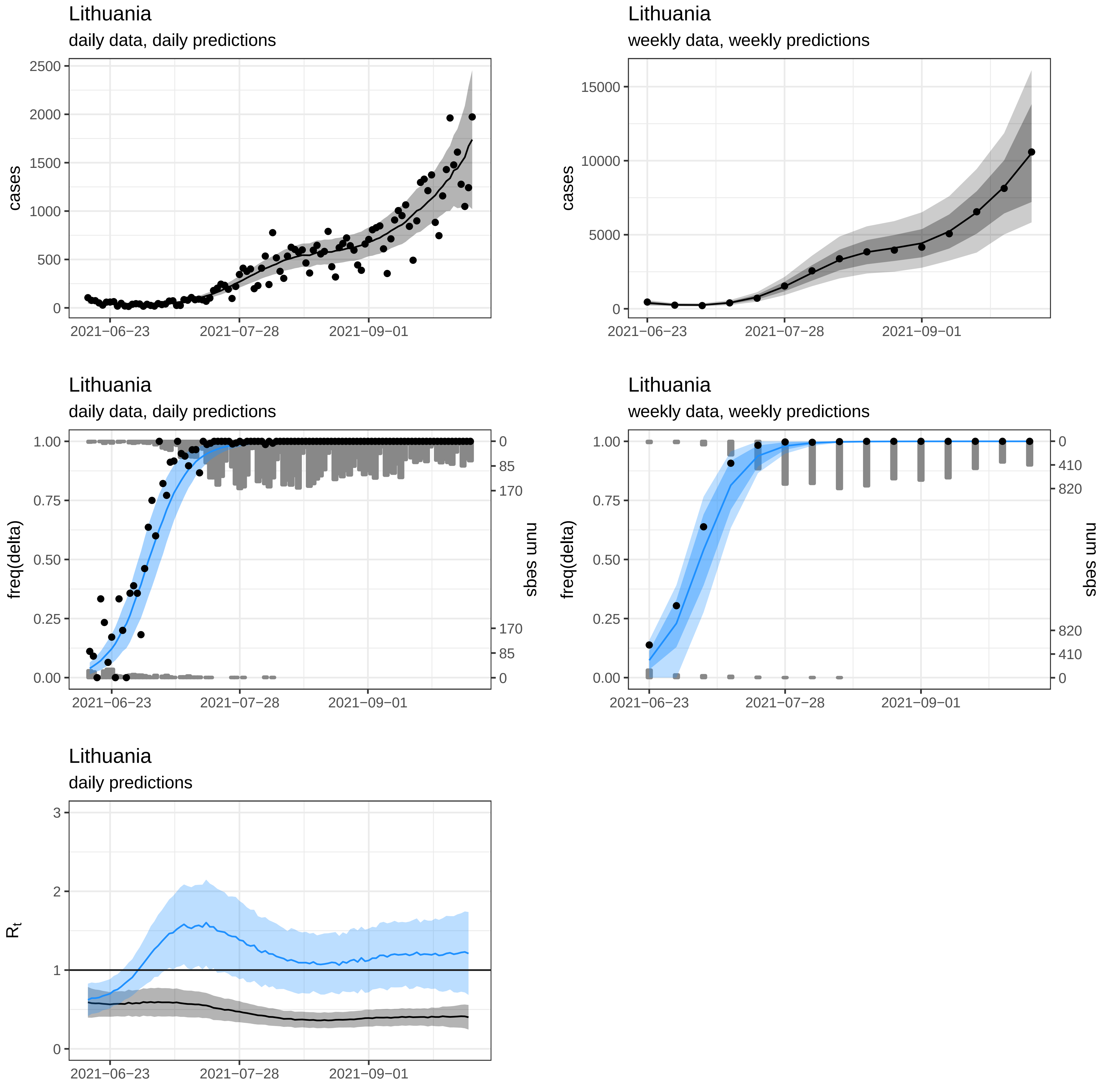
Lithuania
daily data, daily predictions
cases
2500
2000
1500
1000
500
0
2021−06−23
2021−07−28
2021−09−01
Lithuania
weekly data, weekly predictions
cases
15000
10000
5000
0
2021−06−23
2021−07−28
2021−09−01
Lithuania
daily data, daily predictions
freq(delta)
1.00
0.75
0.50
0.25
0.00
num seqs
0
85
170
170
85
0
2021−06−23
2021−07−28
2021−09−01
Lithuania
weekly data, weekly predictions
freq(delta)
1.00
0.75
0.50
0.25
0.00
num seqs
0
410
820
820
410
0
2021−06−23
2021−07−28
2021−09−01
Lithuania
daily predictions
R_t
3
2
1
0
2021−06−23
2021−07−28
2021−09−01

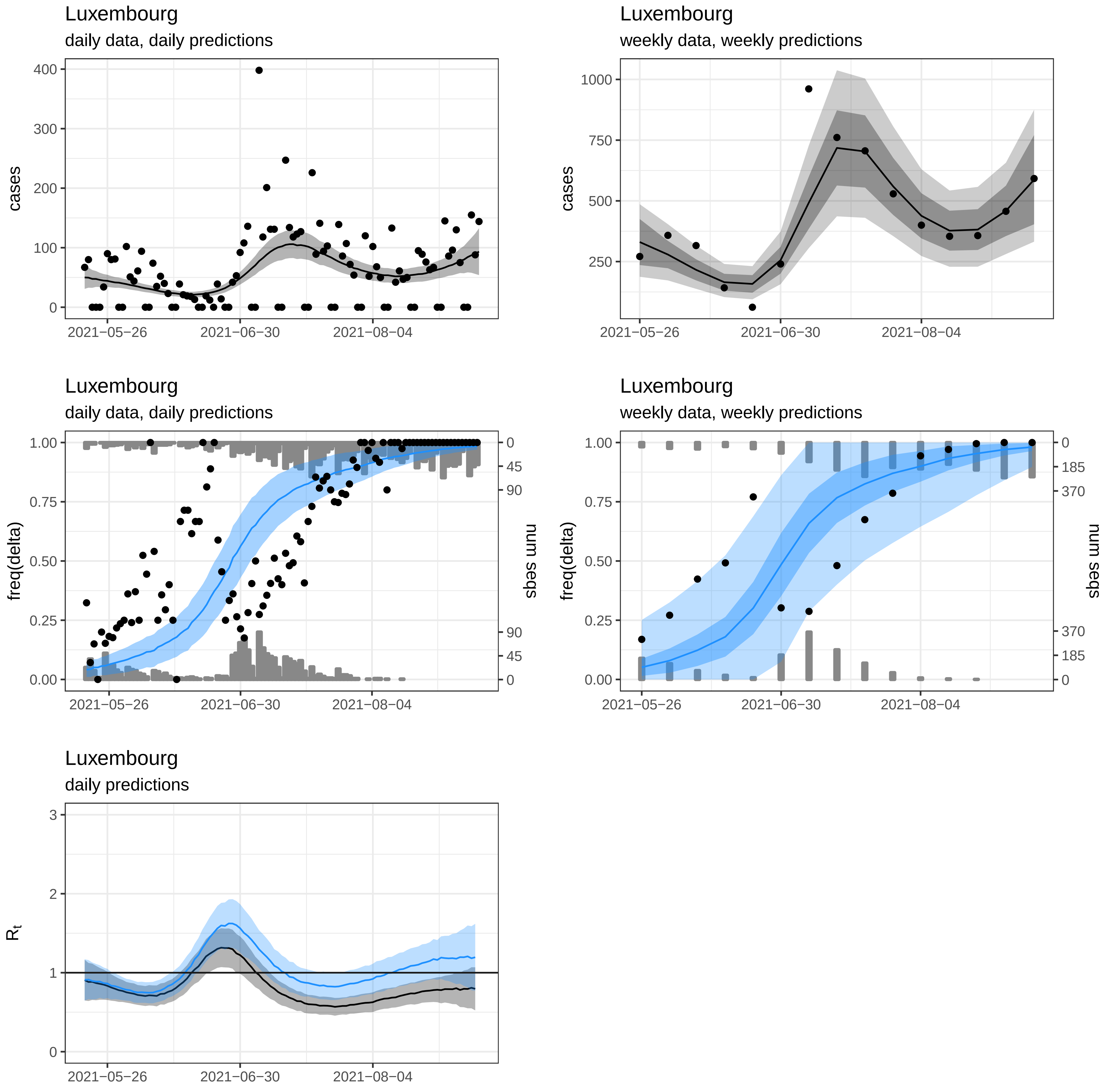
Luxembourg
daily data, daily predictions
cases
400
300
200
100
0
2021−05−26
2021−06−30
2021−08−04
Luxembourg
weekly data, weekly predictions
cases
1000
750
500
250
2021−05−26
2021−06−30
2021−08−04
Luxembourg
daily data, daily predictions
freq(delta)
1.00
0.75
0.50
0.25
0.00
num seqs
0
45
90
90
45
0
2021−05−26
2021−06−30
2021−08−04
Luxembourg
weekly data, weekly predictions
freq(delta)
1.00
0.75
0.50
0.25
0.00
num seqs
0
185
370
370
185
0
2021−05−26
2021−06−30
2021−08−04
Luxembourg
daily predictions
R_t
3
2
1
0
2021−05−26
2021−06−30
2021−08−04

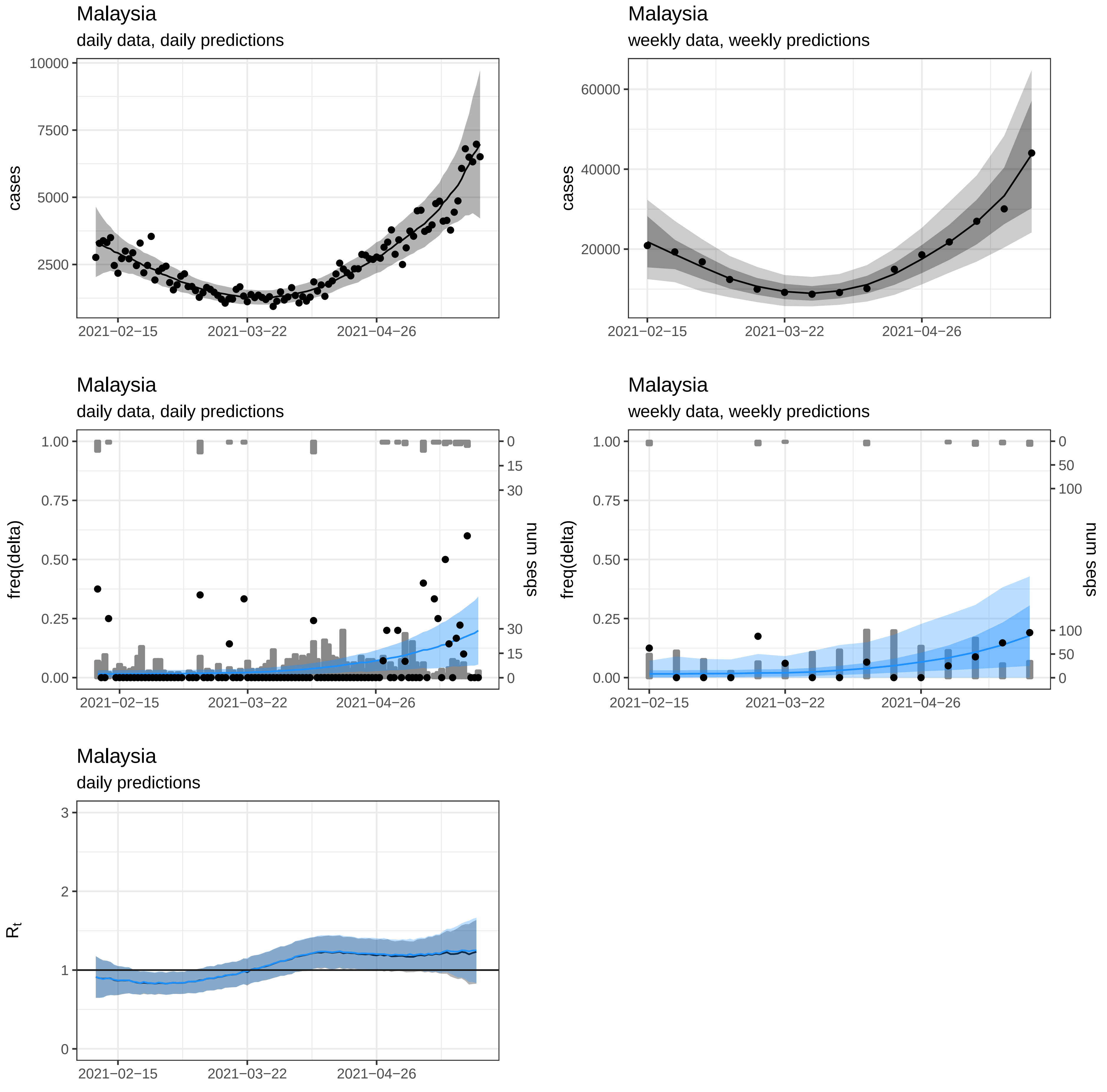

Malaysia
daily data, daily predictions
cases
10000
7500
5000
2500
2021−02−15
2021−03−22
2021−04−26
Malaysia
weekly data, weekly predictions
cases
60000
40000
20000
2021−02−15
2021−03−22
2021−04−26
Malaysia
daily data, daily predictions
freq(delta)
1.00
0.75
0.50
0.25
0.00
num seqs
0
15
30
30
15
0
2021−02−15
2021−03−22
2021−04−26
Malaysia
weekly data, weekly predictions
freq(delta)
1.00
0.75
0.50
0.25
0.00
num seqs
0
50
100
100
50
0
2021−02−15
2021−03−22
2021−04−26
Malaysia
daily predictions
R_t
3
2
1
0
2021−02−15
2021−03−22
2021−04−26

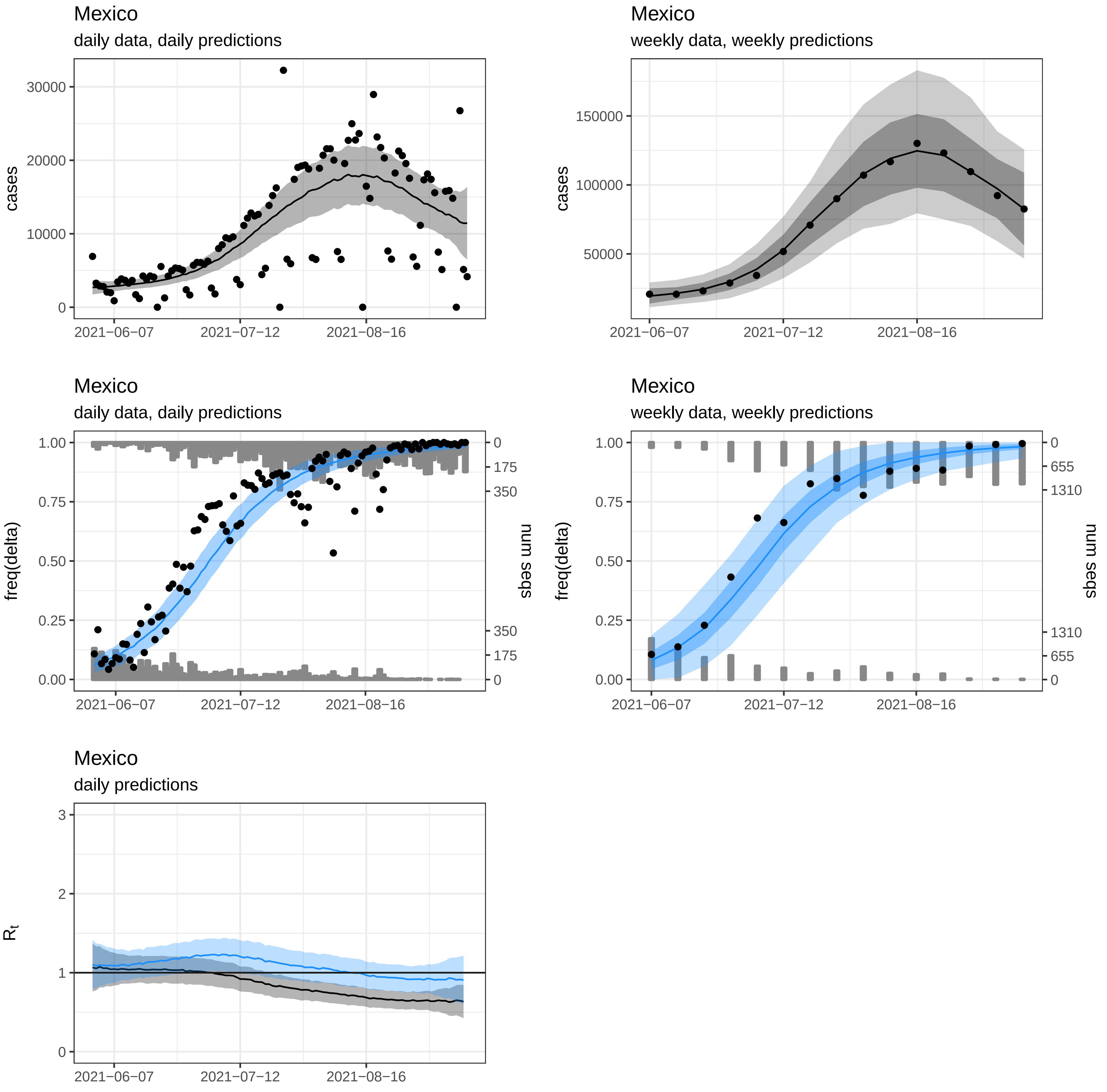
Mexico
daily data, daily predictions
cases
30000
20000
10000
0
2021−06−07
2021−07−12
2021−08−16
Mexico
weekly data, weekly predictions
cases
150000
100000
50000
2021−06−07
2021−07−12
2021−08−16
Mexico
daily data, daily predictions
freq(delta)
1.00
0.75
0.50
0.25
0.00
num seqs
0
175
350
350
175
0
2021−06−07
2021−07−12
2021−08−16
Mexico
weekly data, weekly predictions
freq(delta)
1.00
0.75
0.50
0.25
0.00
num seqs
0
655
1310
1310
655
0
2021−06−07
2021−07−12
2021−08−16
Mexico
daily predictions
R_t
3
2
1
0
2021−06−07
2021−07−12
2021−08−16

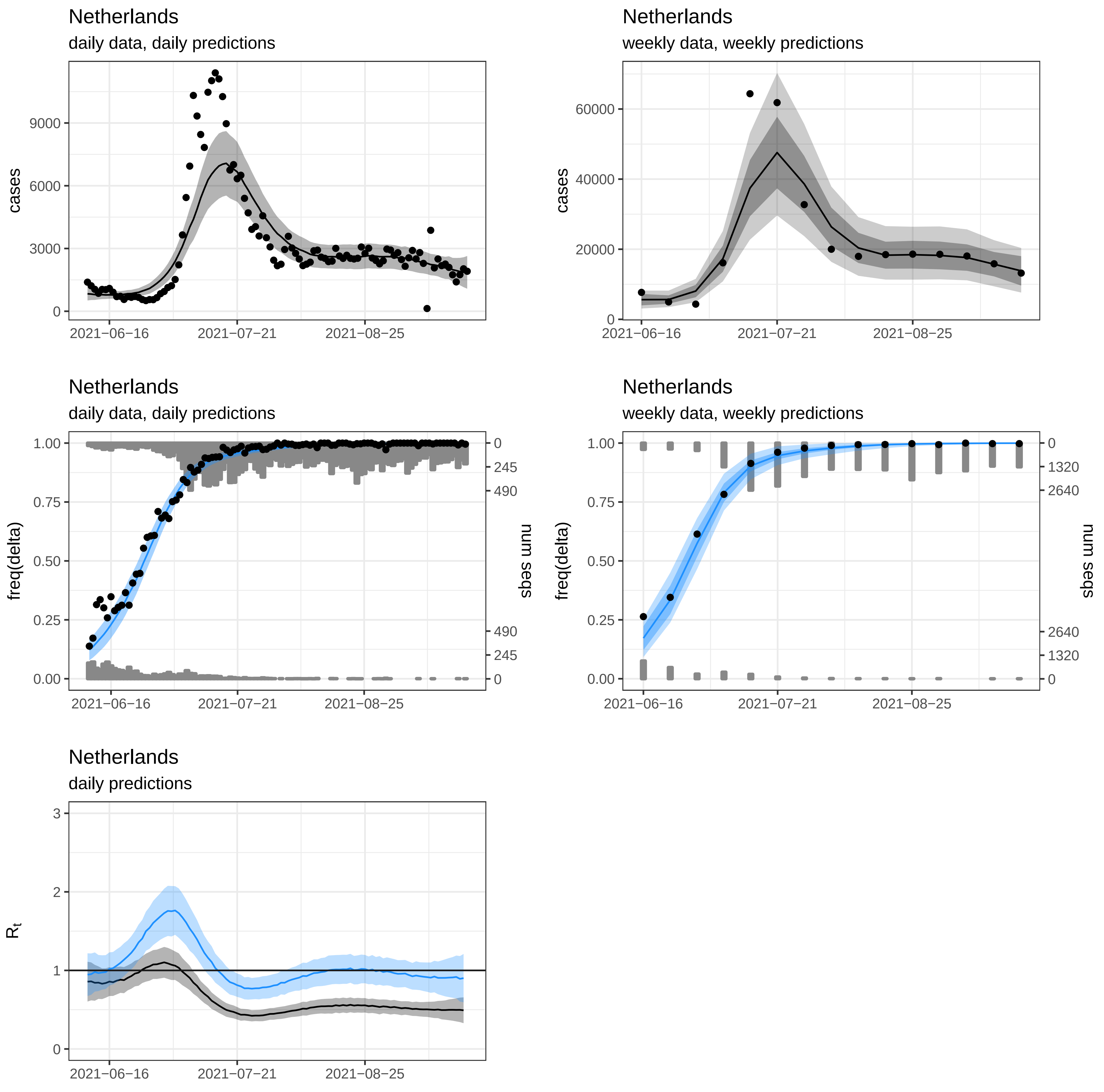

Netherlands
daily data, daily predictions
cases
0
3000
6000
9000
2021−06−16
2021−07−21
2021−08−25
Netherlands
weekly data, weekly predictions
cases
0
20000
40000
60000
2021−06−16
2021−07−21
2021−08−25
Netherlands
daily data, daily predictions
freq(delta)
0.00
0.25
0.50
0.75
1.00
num seqs
0
245
490
2021−06−16
2021−07−21
2021−08−25
Netherlands
weekly data, weekly predictions
freq(delta)
0.00
0.25
0.50
0.75
1.00
num seqs
0
1320
2640
2021−06−16
2021−07−21
2021−08−25
Netherlands
daily predictions
R_t
0
1
2
3
2021−06−16
2021−07−21
2021−08−25

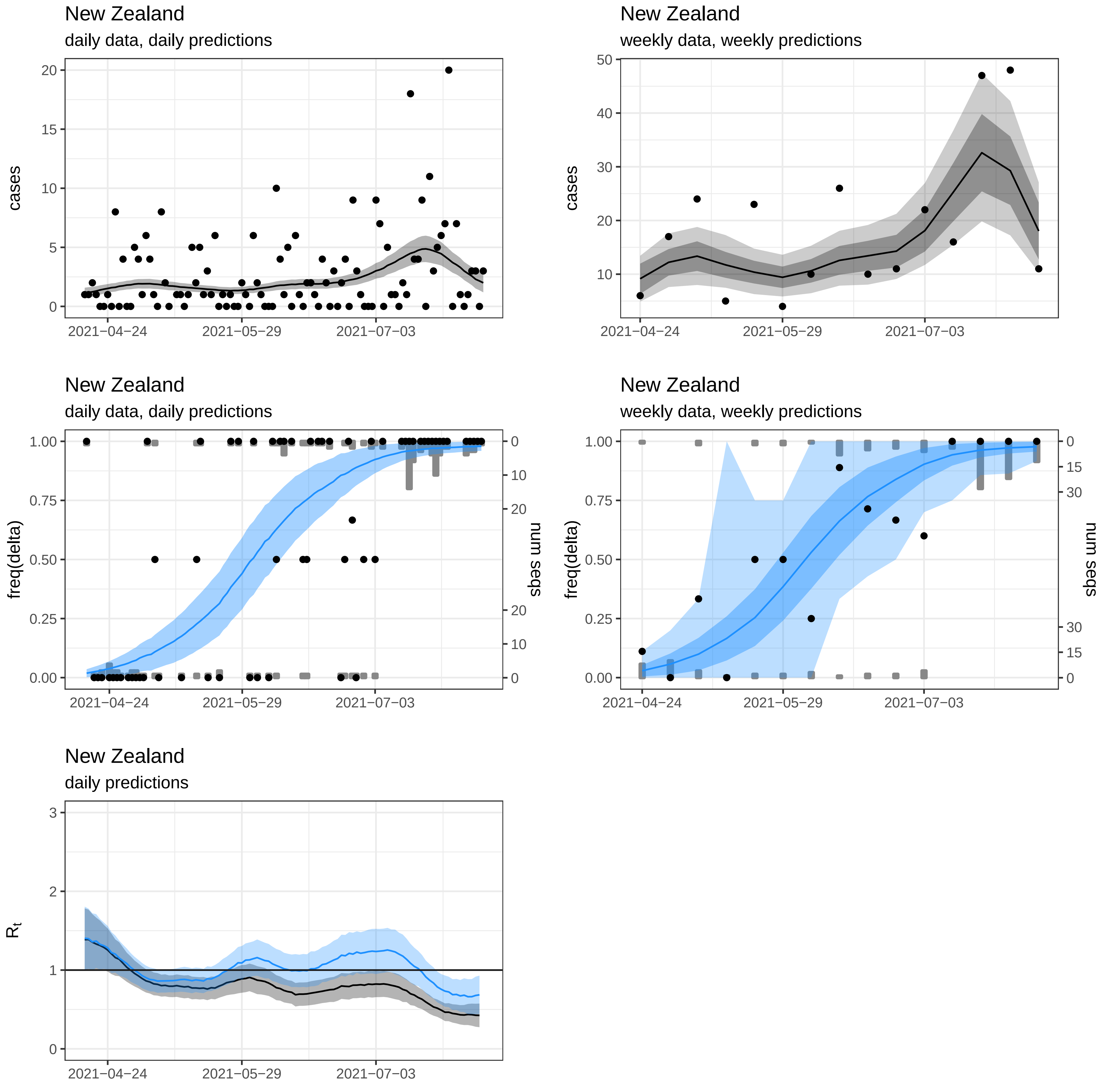

New Zealand
daily data, daily predictions
cases
0
5
10
15
20
2021−04−24
2021−05−29
2021−07−03
New Zealand
weekly data, weekly predictions
cases
10
20
30
40
50
2021−04−24
2021−05−29
2021−07−03
New Zealand
daily data, daily predictions
freq(delta)
0.00
0.25
0.50
0.75
1.00
num seqs
0
10
20
20
10
0
2021−04−24
2021−05−29
2021−07−03
New Zealand
weekly data, weekly predictions
freq(delta)
0.00
0.25
0.50
0.75
1.00
num seqs
0
15
30
30
15
0
2021−04−24
2021−05−29
2021−07−03
New Zealand
daily predictions
R_t
0
1
2
3
2021−04−24
2021−05−29
2021−07−03

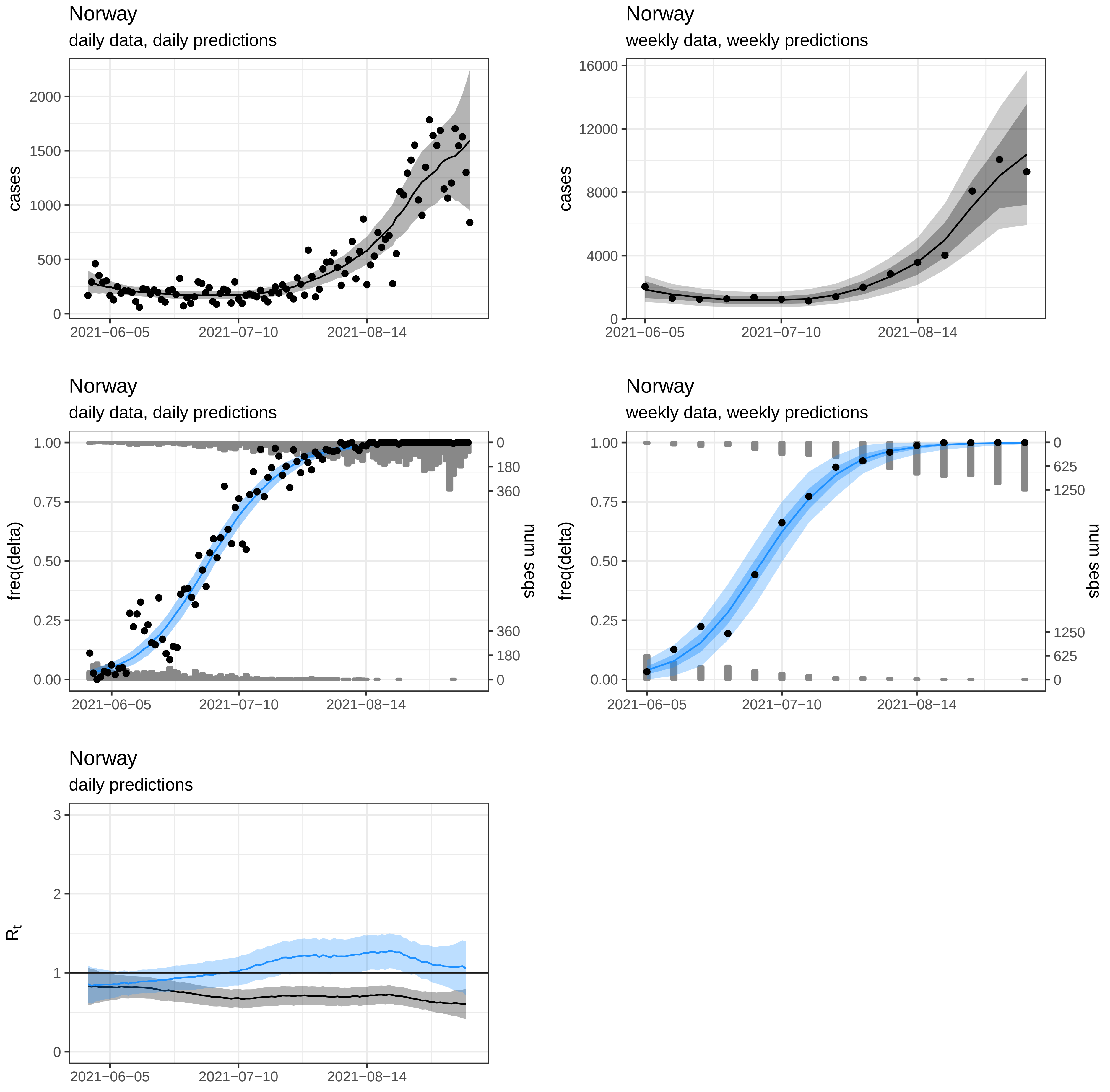
Norway
daily data, daily predictions
cases
0
500
1000
1500
2000
2021−06−05
2021−07−10
2021−08−14
Norway
weekly data, weekly predictions
cases
0
4000
8000
12000
16000
2021−06−05
2021−07−10
2021−08−14
Norway
daily data, daily predictions
freq(delta)
0.00
0.25
0.50
0.75
1.00
num seqs
0
180
360
360
180
0
2021−06−05
2021−07−10
2021−08−14
Norway
weekly data, weekly predictions
freq(delta)
0.00
0.25
0.50
0.75
1.00
num seqs
0
625
1250
1250
625
0
2021−06−05
2021−07−10
2021−08−14
Norway
daily predictions
R_t
0
1
2
3
2021−06−05
2021−07−10
2021−08−14

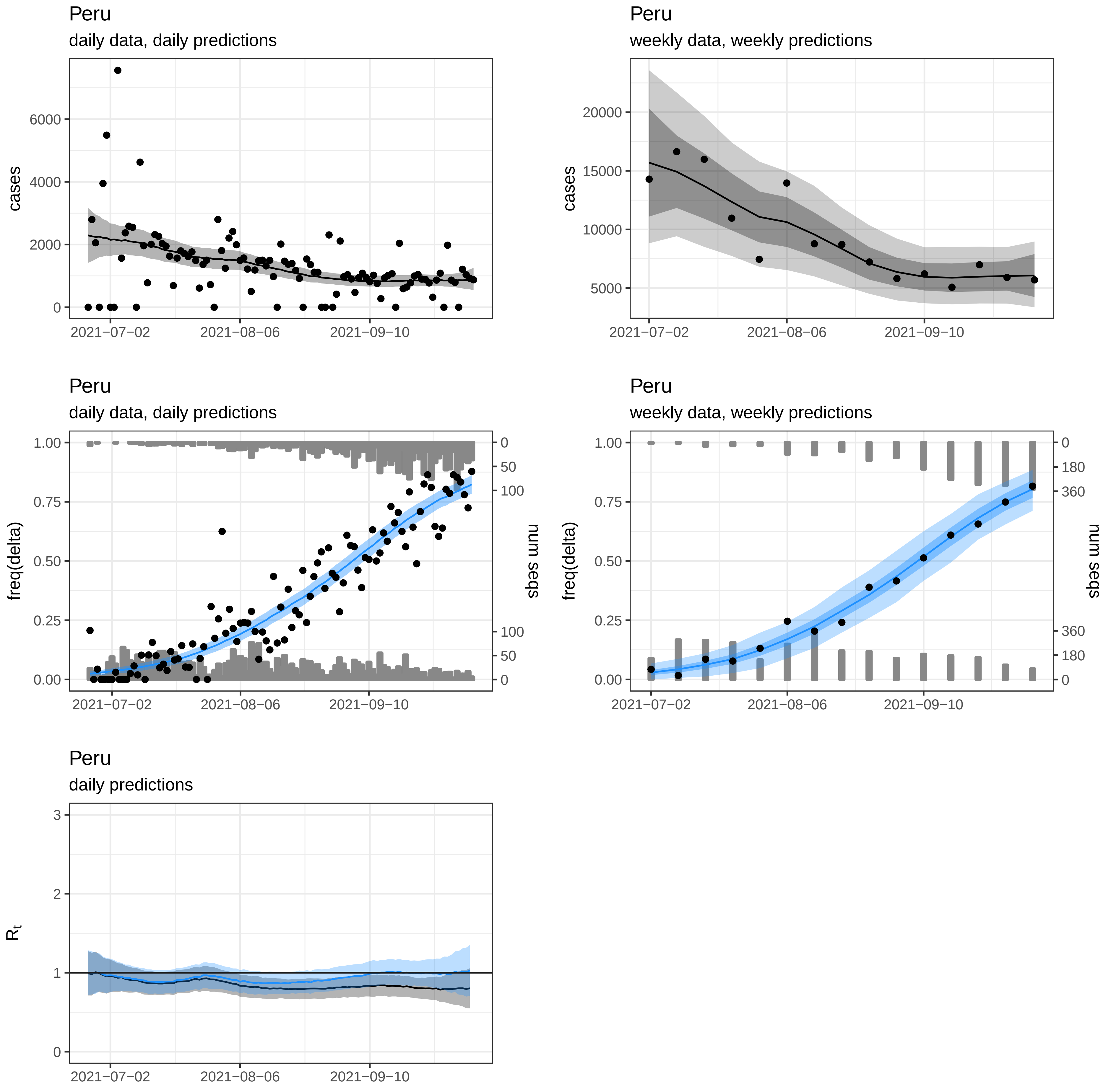
Peru
daily data, daily predictions
cases
0
2000
4000
6000
2021−07−02
2021−08−06
2021−09−10
Peru
weekly data, weekly predictions
cases
5000
10000
15000
20000
2021−07−02
2021−08−06
2021−09−10
Peru
daily data, daily predictions
freq(delta)
0.00
0.25
0.50
0.75
1.00
num seqs
0
50
100
2021−07−02
2021−08−06
2021−09−10
Peru
weekly data, weekly predictions
freq(delta)
0.00
0.25
0.50
0.75
1.00
num seqs
0
180
360
2021−07−02
2021−08−06
2021−09−10
Peru
daily predictions
R_t
0
1
2
3
2021−07−02
2021−08−06
2021−09−10

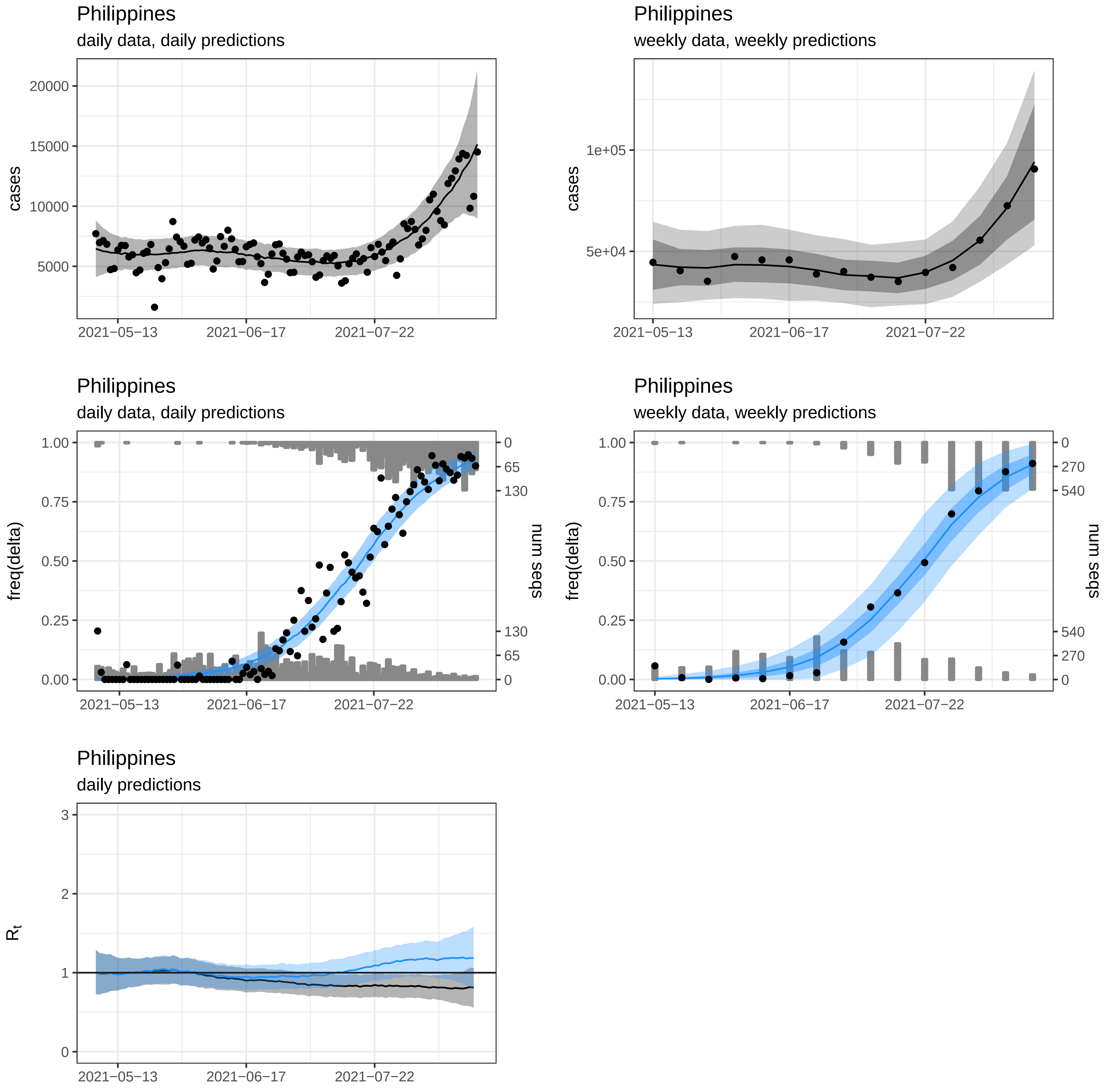
Philippines
daily data, daily predictions
cases
20000
15000
10000
5000
2021−05−13
2021−06−17
2021−07−22
Philippines
weekly data, weekly predictions
cases
1e+05
5e+04
2021−05−13
2021−06−17
2021−07−22
Philippines
daily data, daily predictions
freq(delta)
num seqs
1.00
0.75
0.50
0.25
0.00
0
65
130
2021−05−13
2021−06−17
2021−07−22
Philippines
weekly data, weekly predictions
freq(delta)
num seqs
1.00
0.75
0.50
0.25
0.00
0
270
540
2021−05−13
2021−06−17
2021−07−22
Philippines
daily predictions
R_t
3
2
1
0
2021−05−13
2021−06−17
2021−07−22

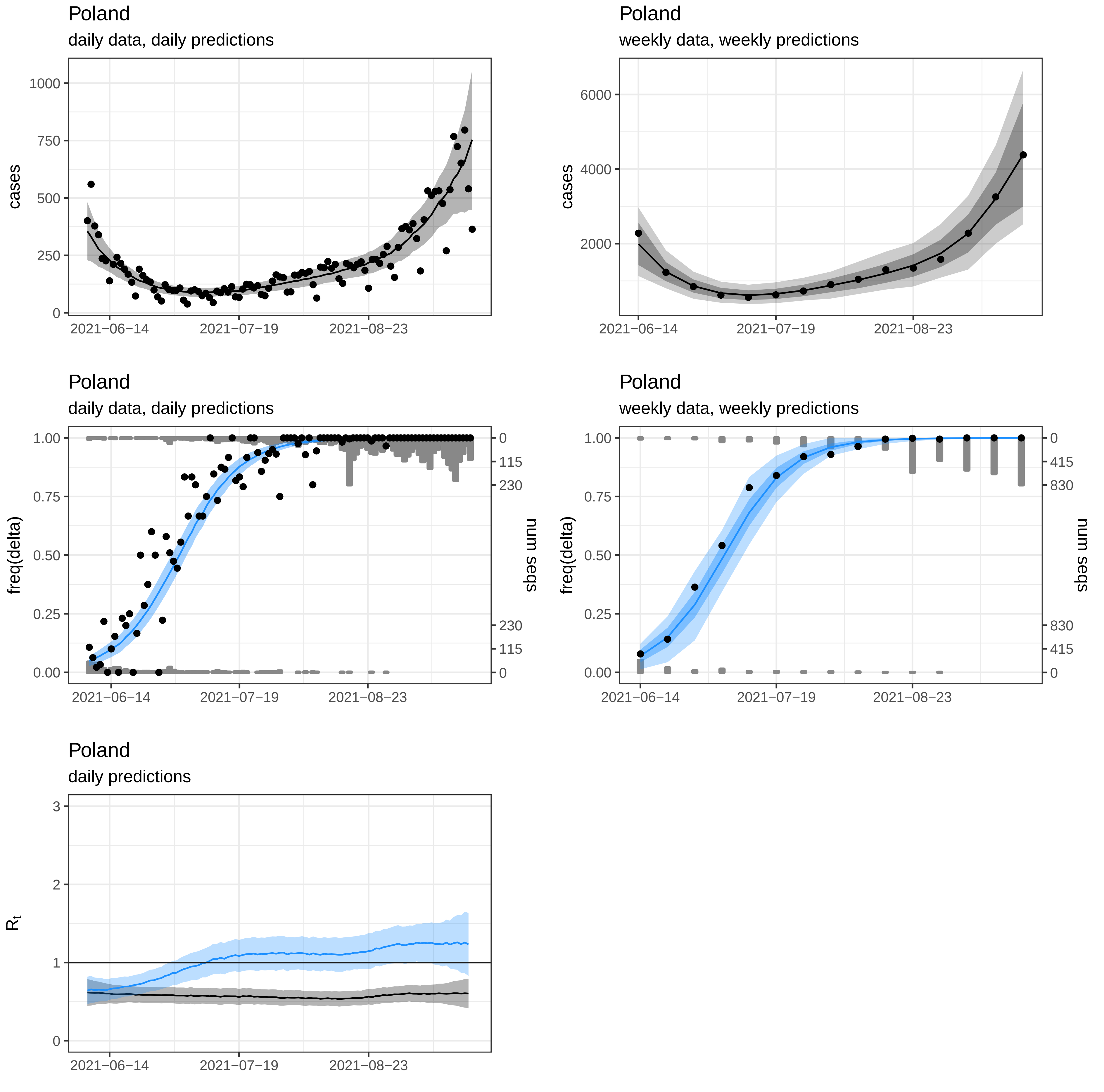

Poland
daily data, daily predictions
cases
1000
750
500
250
0
2021−06−14
2021−07−19
2021−08−23
Poland
weekly data, weekly predictions
cases
6000
4000
2000
2021−06−14
2021−07−19
2021−08−23
Poland
daily data, daily predictions
freq(delta)
1.00
0.75
0.50
0.25
0.00
num seqs
0
115
230
230
115
0
2021−06−14
2021−07−19
2021−08−23
Poland
weekly data, weekly predictions
freq(delta)
1.00
0.75
0.50
0.25
0.00
num seqs
0
415
830
830
415
0
2021−06−14
2021−07−19
2021−08−23
Poland
daily predictions
R_t
3
2
1
0
2021−06−14
2021−07−19
2021−08−23

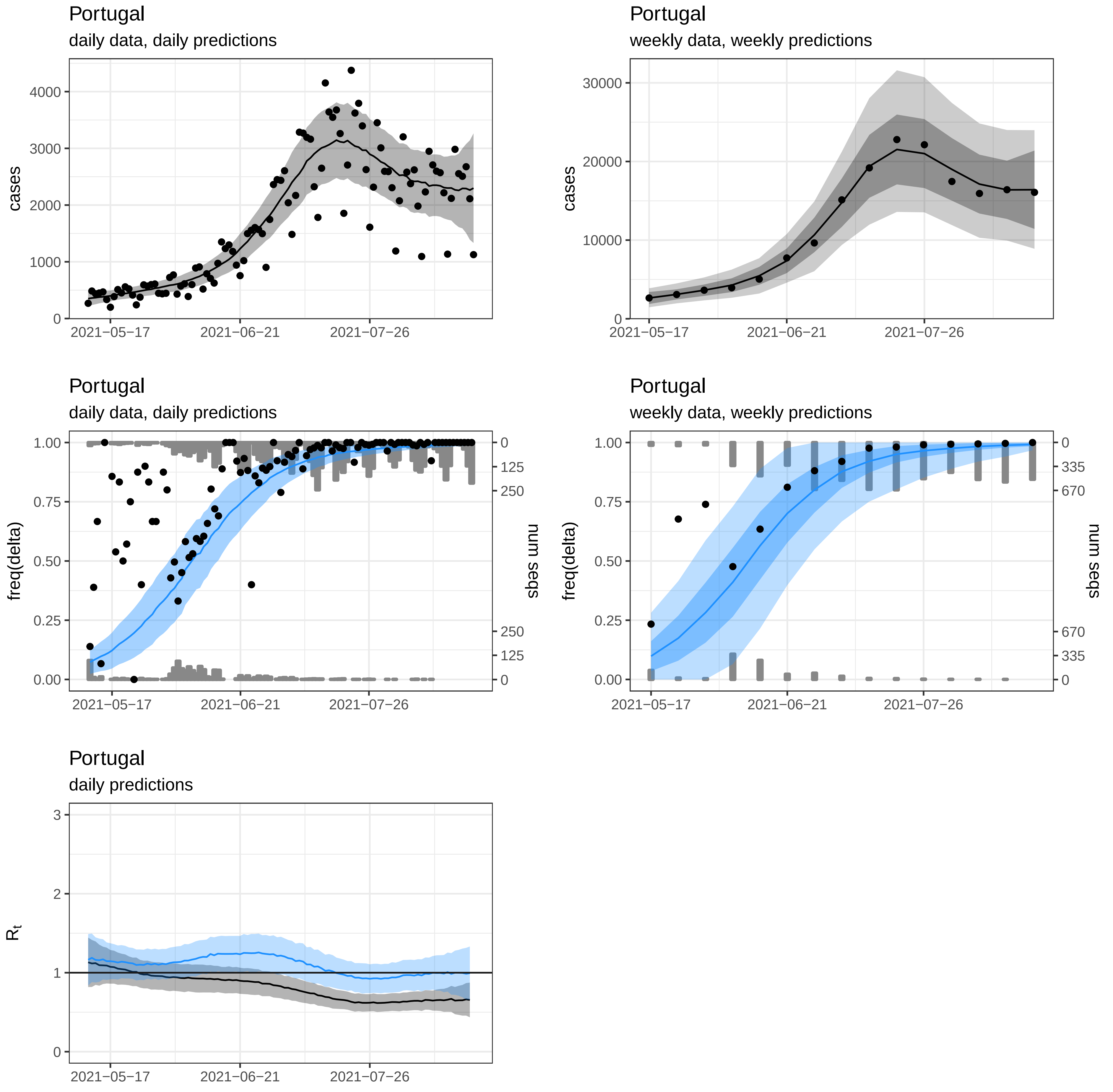

Portugal
daily data, daily predictions
cases
0
1000
2000
3000
4000
2021−05−17
2021−06−21
2021−07−26
Portugal
weekly data, weekly predictions
cases
0
10000
20000
30000
2021−05−17
2021−06−21
2021−07−26
Portugal
daily data, daily predictions
freq(delta)
0.00
0.25
0.50
0.75
1.00
num seqs
0
125
250
250
125
0
2021−05−17
2021−06−21
2021−07−26
Portugal
weekly data, weekly predictions
freq(delta)
0.00
0.25
0.50
0.75
1.00
num seqs
0
335
670
670
335
0
2021−05−17
2021−06−21
2021−07−26
Portugal
daily predictions
R_t
0
1
2
3
2021−05−17
2021−06−21
2021−07−26

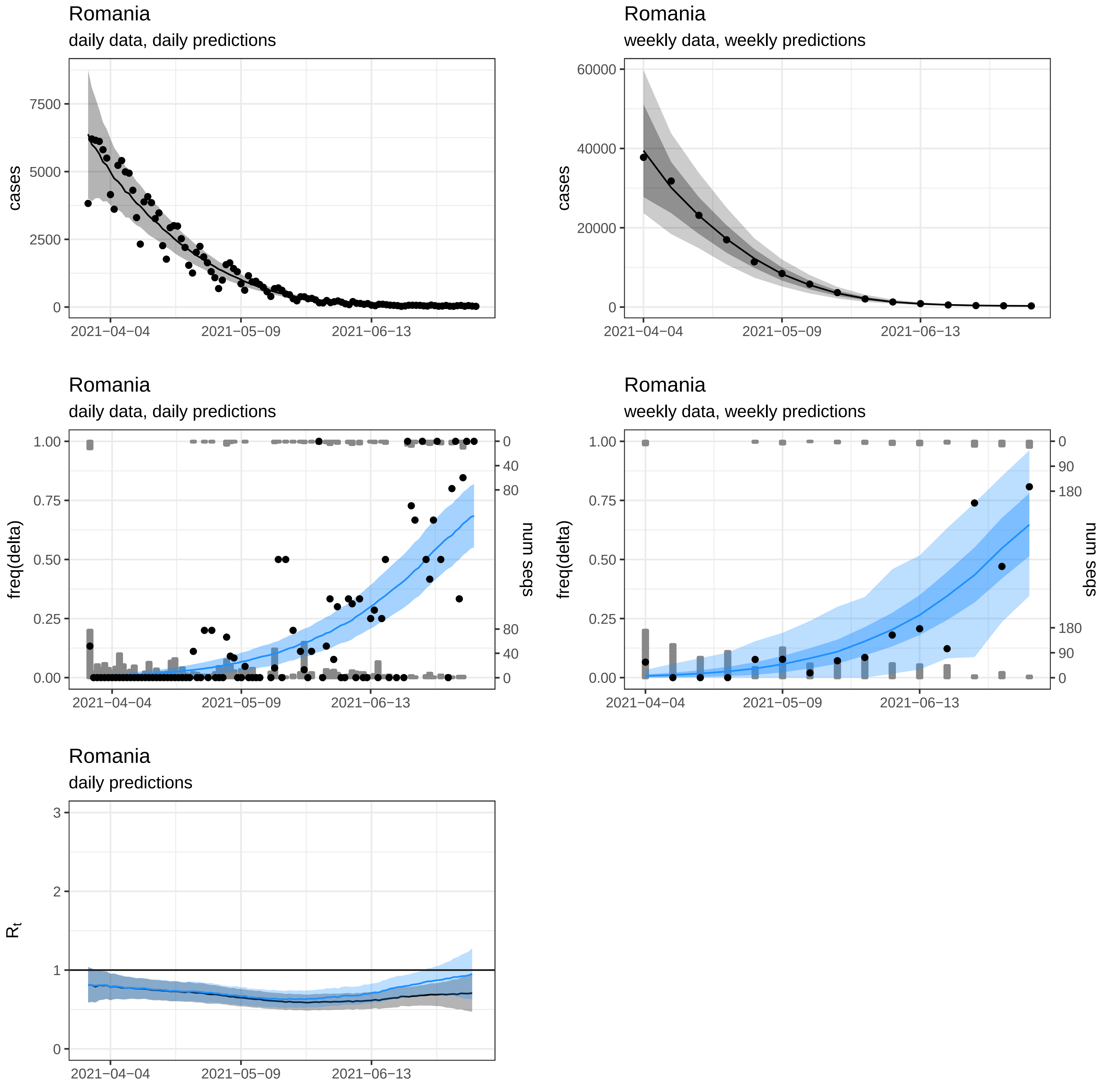

Romania
daily data, daily predictions
cases
7500
5000
2500
0
2021−04−04
2021−05−09
2021−06−13
Romania
weekly data, weekly predictions
cases
60000
40000
20000
0
2021−04−04
2021−05−09
2021−06−13
Romania
daily data, daily predictions
freq(delta)
1.00
0.75
0.50
0.25
0.00
num seqs
0
40
80
80
40
0
2021−04−04
2021−05−09
2021−06−13
Romania
weekly data, weekly predictions
freq(delta)
1.00
0.75
0.50
0.25
0.00
num seqs
0
90
180
180
90
0
2021−04−04
2021−05−09
2021−06−13
Romania
daily predictions
R_t
3
2
1
0
2021−04−04
2021−05−09
2021−06−13

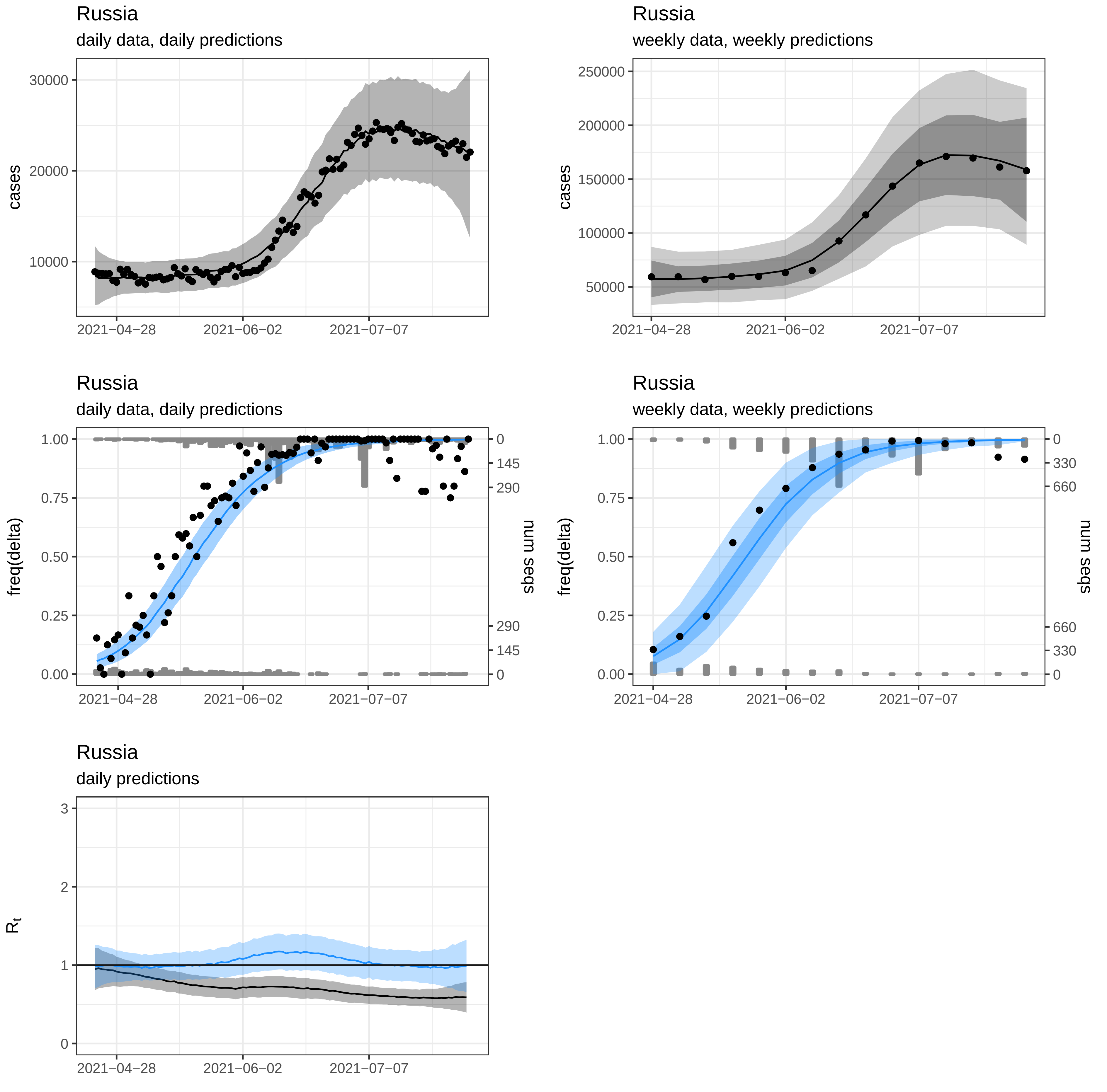
Russia
daily data, daily predictions
cases
30000
20000
10000
2021−04−28
2021−06−02
2021−07−07
Russia
weekly data, weekly predictions
cases
250000
200000
150000
100000
50000
2021−04−28
2021−06−02
2021−07−07
Russia
daily data, daily predictions
freq(delta)
1.00
0.75
0.50
0.25
0.00
num seqs
0
145
290
290
145
0
2021−04−28
2021−06−02
2021−07−07
Russia
weekly data, weekly predictions
freq(delta)
1.00
0.75
0.50
0.25
0.00
num seqs
0
330
660
660
330
0
2021−04−28
2021−06−02
2021−07−07
Russia
daily predictions
R_t
3
2
1
0
2021−04−28
2021−06−02
2021−07−07

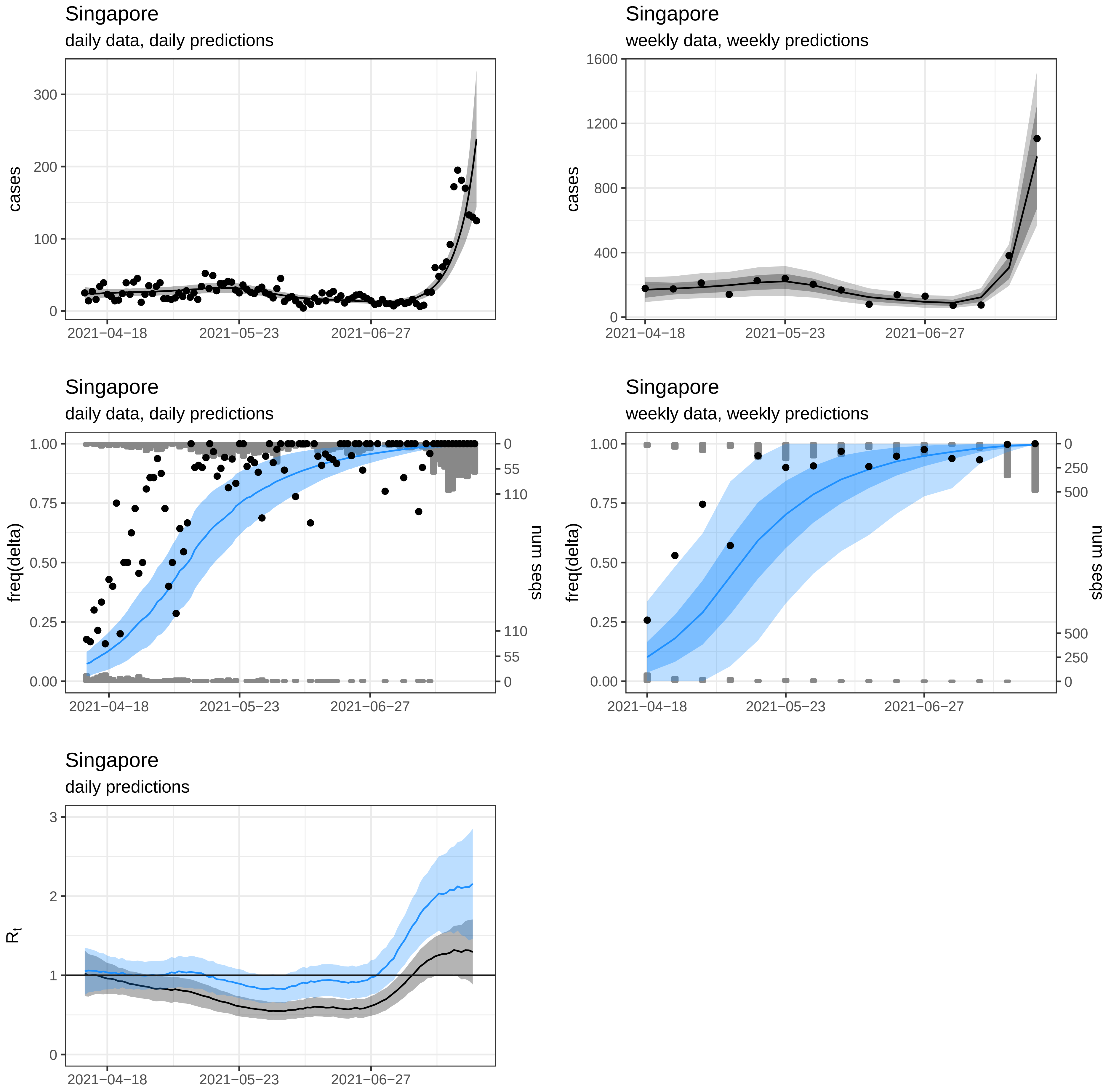
Singapore
daily data, daily predictions
cases
300
200
100
0
2021−04−18
2021−05−23
2021−06−27
Singapore
weekly data, weekly predictions
cases
1600
1200
800
400
0
2021−04−18
2021−05−23
2021−06−27
Singapore
daily data, daily predictions
freq(delta)
1.00
0.75
0.50
0.25
0.00
num seqs
0
55
110
110
55
0
2021−04−18
2021−05−23
2021−06−27
Singapore
weekly data, weekly predictions
freq(delta)
1.00
0.75
0.50
0.25
0.00
num seqs
0
250
500
500
250
0
2021−04−18
2021−05−23
2021−06−27
Singapore
daily predictions
R_t
3
2
1
0
2021−04−18
2021−05−23
2021−06−27

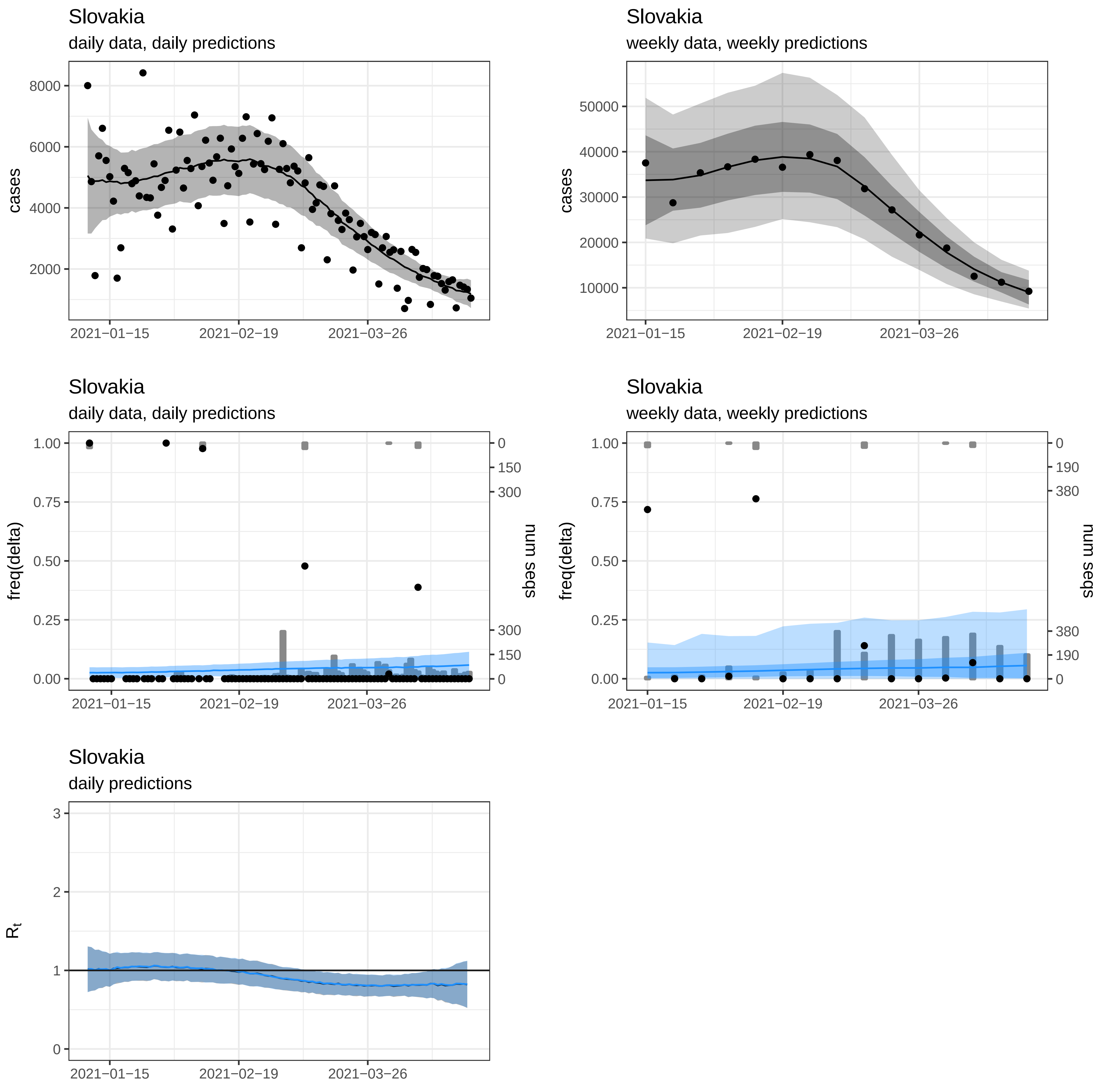
Slovakia
daily data, daily predictions
cases
8000
6000
4000
2000
2021−01−15
2021−02−19
2021−03−26
Slovakia
weekly data, weekly predictions
cases
50000
40000
30000
20000
10000
2021−01−15
2021−02−19
2021−03−26
Slovakia
daily data, daily predictions
freq(delta)
1.00
0.75
0.50
0.25
0.00
num seqs
0
150
300
300
150
0
2021−01−15
2021−02−19
2021−03−26
Slovakia
weekly data, weekly predictions
freq(delta)
1.00
0.75
0.50
0.25
0.00
num seqs
0
190
380
380
190
0
2021−01−15
2021−02−19
2021−03−26
Slovakia
daily predictions
R_t
3
2
1
0
2021−01−15
2021−02−19
2021−03−26

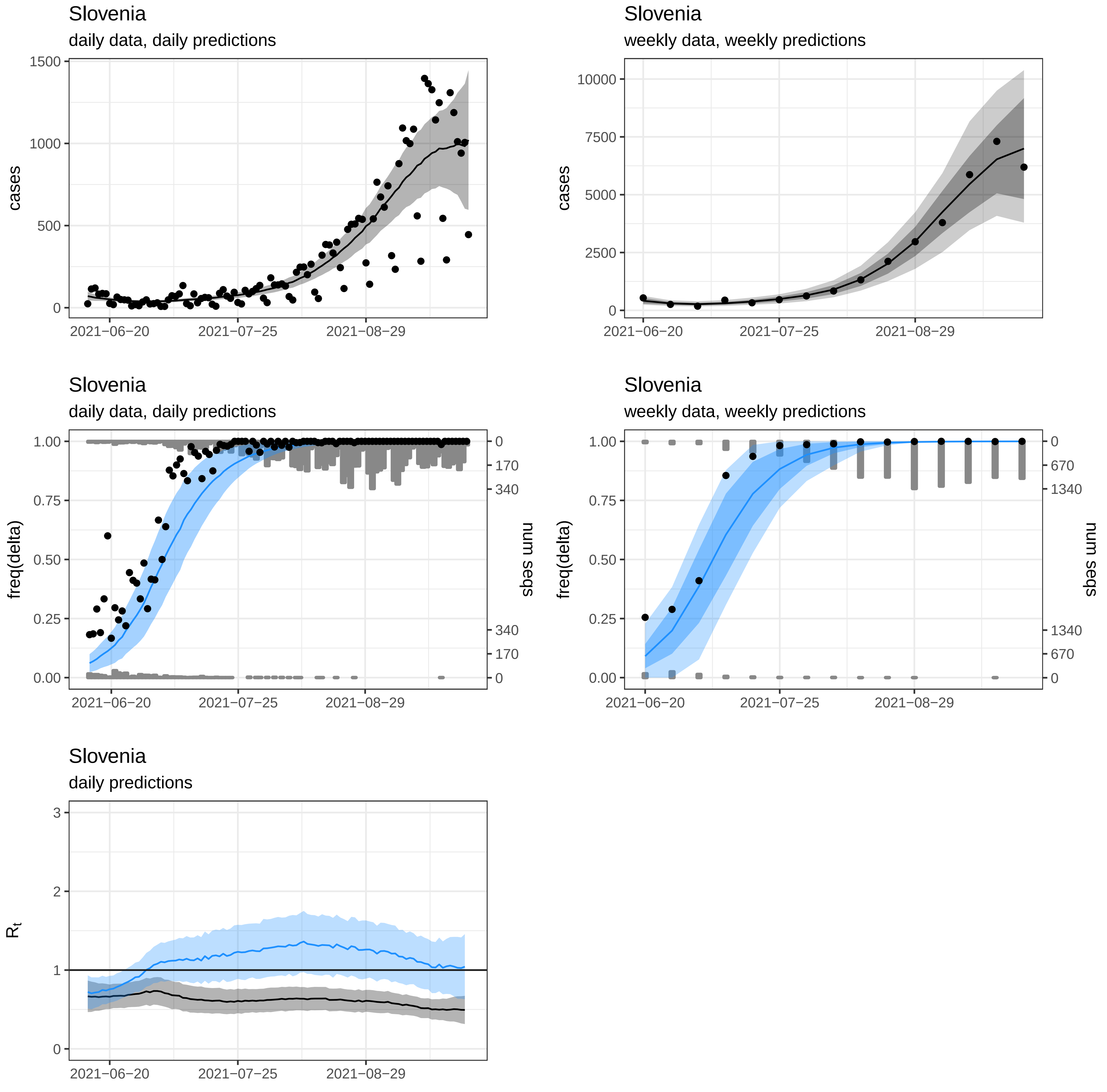
Slovenia
daily data, daily predictions
cases
1500
1000
500
0
2021−06−20
2021−07−25
2021−08−29
Slovenia
weekly data, weekly predictions
cases
10000
7500
5000
2500
0
2021−06−20
2021−07−25
2021−08−29
Slovenia
daily data, daily predictions
freq(delta)
num seqs
1.00
0.75
0.50
0.25
0.00
0
170
340
340
170
0
2021−06−20
2021−07−25
2021−08−29
Slovenia
weekly data, weekly predictions
freq(delta)
num seqs
1.00
0.75
0.50
0.25
0.00
0
670
1340
1340
670
0
2021−06−20
2021−07−25
2021−08−29
Slovenia
daily predictions
R_t
3
2
1
0
2021−06−20
2021−07−25
2021−08−29

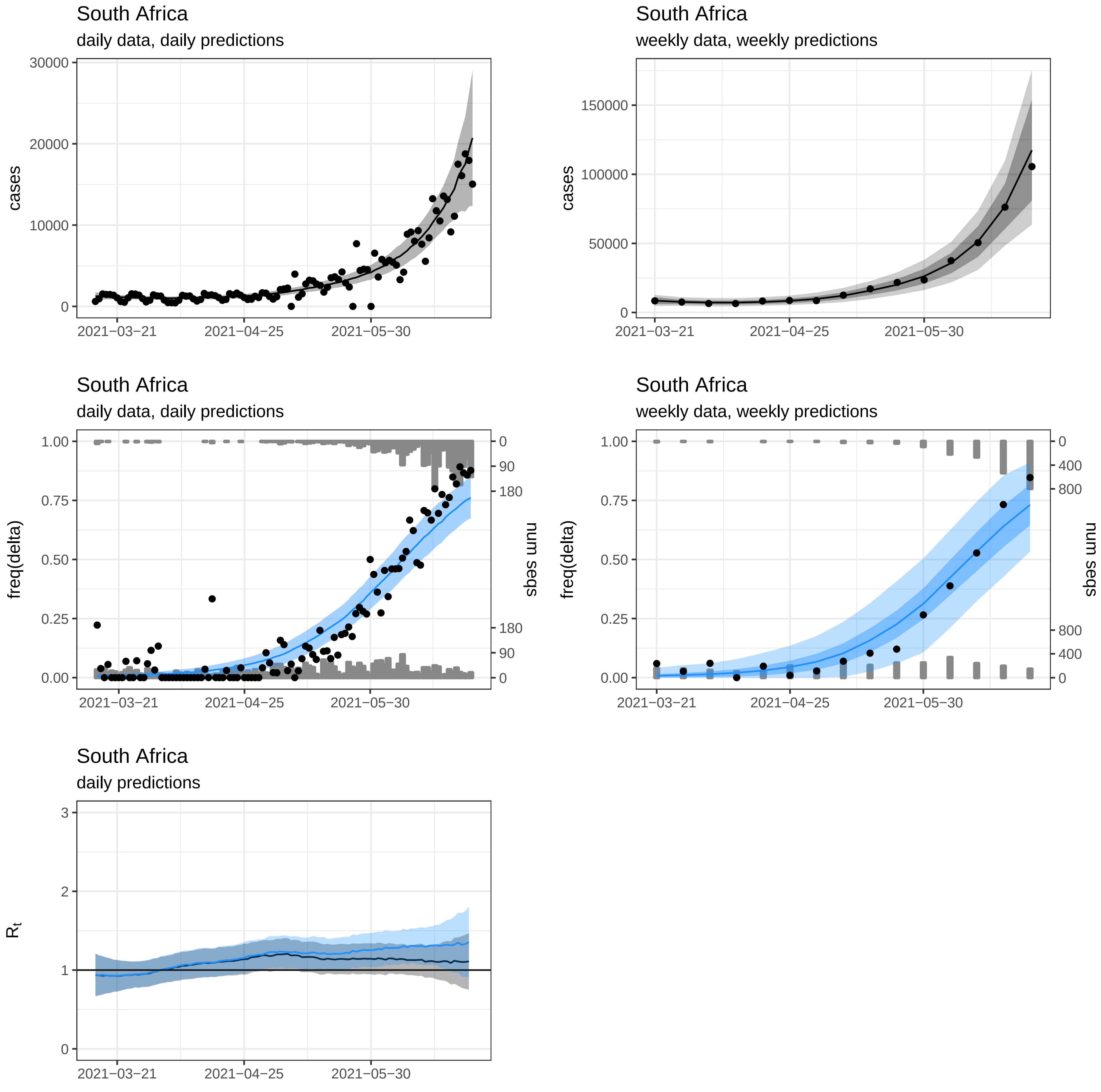

South Africa
daily data, daily predictions
cases
30000
20000
10000
0
2021−03−21
2021−04−25
2021−05−30
South Africa
weekly data, weekly predictions
cases
150000
100000
50000
0
2021−03−21
2021−04−25
2021−05−30
South Africa
daily data, daily predictions
freq(delta)
1.00
0.75
0.50
0.25
0.00
num seqs
0
90
180
180
90
0
2021−03−21
2021−04−25
2021−05−30
South Africa
weekly data, weekly predictions
freq(delta)
1.00
0.75
0.50
0.25
0.00
num seqs
0
400
800
800
400
0
2021−03−21
2021−04−25
2021−05−30
South Africa
daily predictions
R_t
3
2
1
0
2021−03−21
2021−04−25
2021−05−30

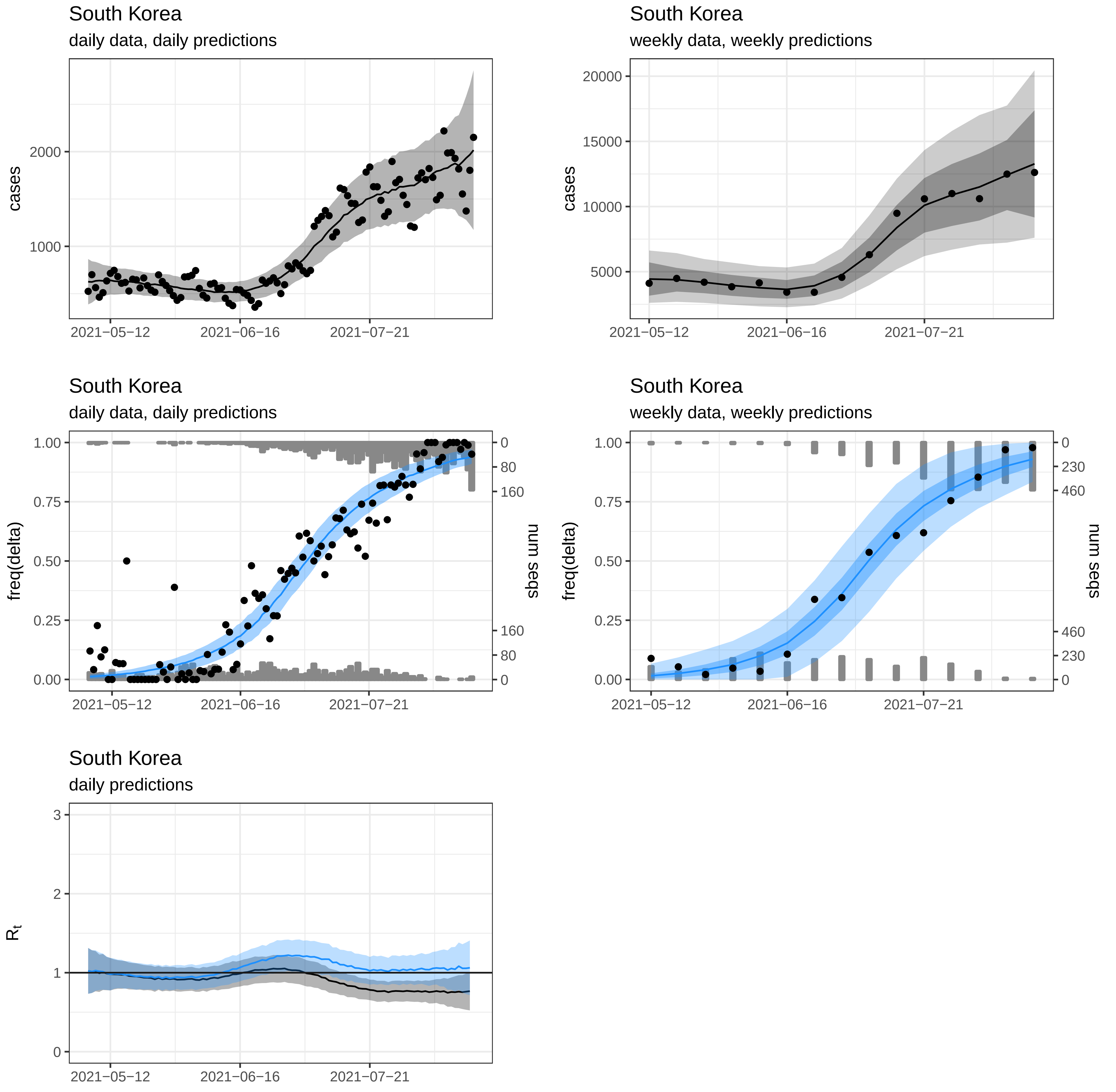
South Korea
daily data, daily predictions
cases
2000
1000
2021−05−12
2021−06−16
2021−07−21
South Korea
weekly data, weekly predictions
cases
20000
15000
10000
5000
2021−05−12
2021−06−16
2021−07−21
South Korea
daily data, daily predictions
freq(delta)
1.00
0.75
0.50
0.25
0.00
num seqs
0
80
160
160
80
0
2021−05−12
2021−06−16
2021−07−21
South Korea
weekly data, weekly predictions
freq(delta)
1.00
0.75
0.50
0.25
0.00
num seqs
0
230
460
460
230
0
2021−05−12
2021−06−16
2021−07−21
South Korea
daily predictions
R_t
3
2
1
0
2021−05−12
2021−06−16
2021−07−21

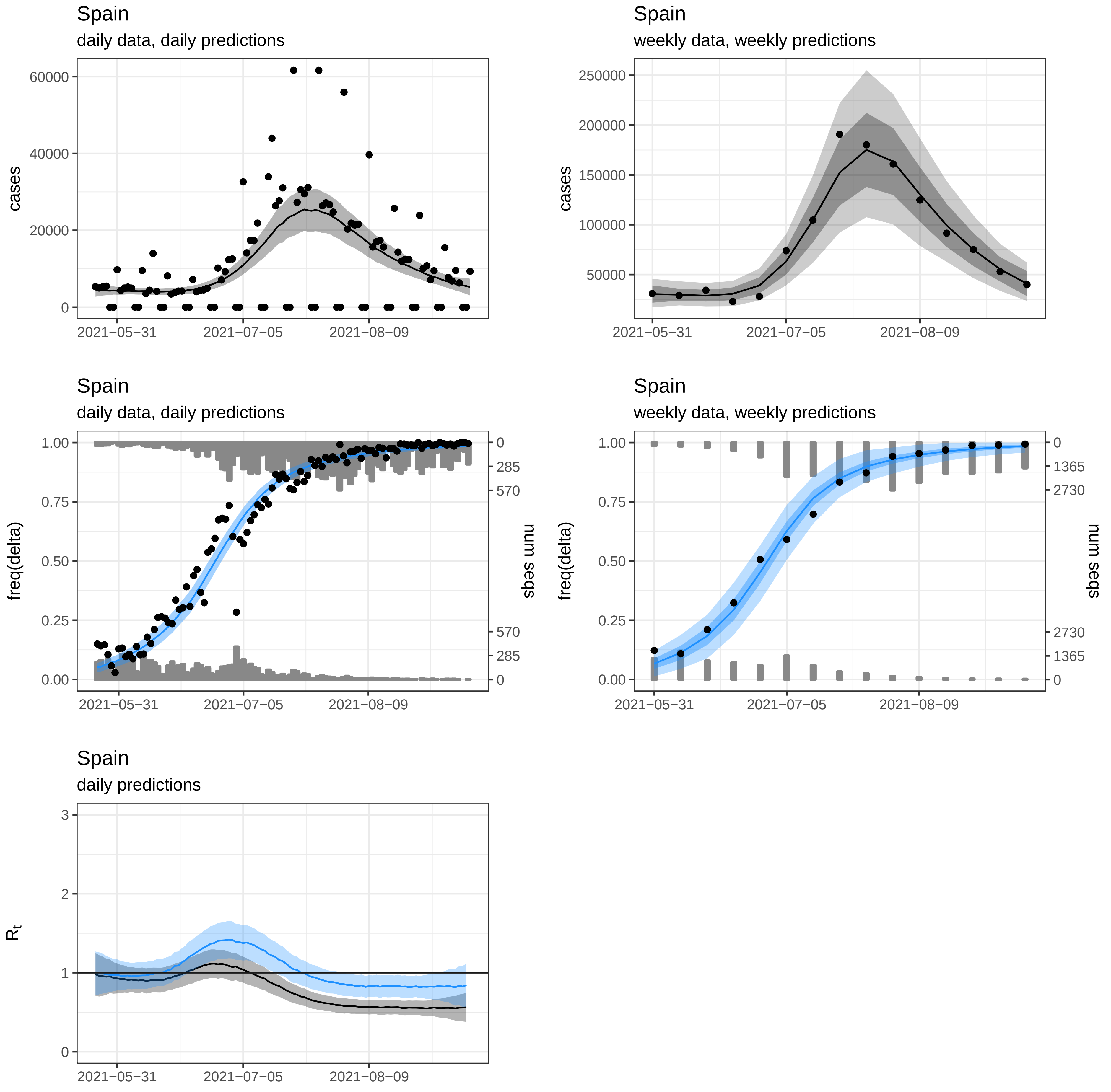
Spain
daily data, daily predictions
cases
60000
40000
20000
0
2021−05−31
2021−07−05
2021−08−09
Spain
weekly data, weekly predictions
cases
250000
200000
150000
100000
50000
2021−05−31
2021−07−05
2021−08−09
Spain
daily data, daily predictions
freq(delta)
1.00
0.75
0.50
0.25
0.00
num seqs
0
285
570
570
285
0
2021−05−31
2021−07−05
2021−08−09
Spain
weekly data, weekly predictions
freq(delta)
1.00
0.75
0.50
0.25
0.00
num seqs
0
1365
2730
2730
1365
0
2021−05−31
2021−07−05
2021−08−09
Spain
daily predictions
R_t
3
2
1
0
2021−05−31
2021−07−05
2021−08−09

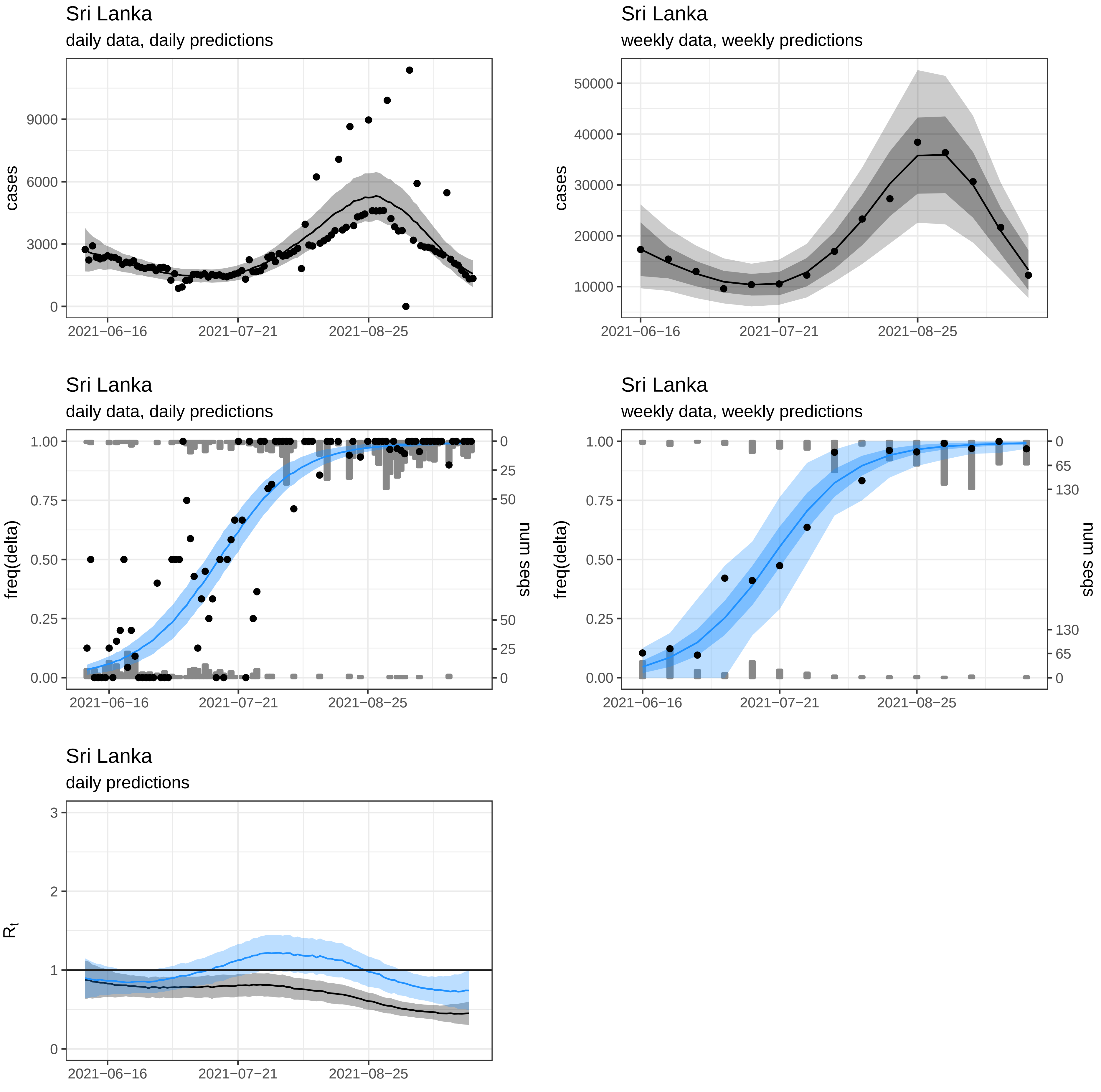
Sri Lanka
daily data, daily predictions
cases
0
3000
6000
9000
2021−06−16
2021−07−21
2021−08−25
Sri Lanka
weekly data, weekly predictions
cases
10000
20000
30000
40000
50000
2021−06−16
2021−07−21
2021−08−25
Sri Lanka
daily data, daily predictions
freq(delta)
0.00
0.25
0.50
0.75
1.00
num seqs
0
25
50
2021−06−16
2021−07−21
2021−08−25
Sri Lanka
weekly data, weekly predictions
freq(delta)
0.00
0.25
0.50
0.75
1.00
num seqs
0
65
130
2021−06−16
2021−07−21
2021−08−25
Sri Lanka
daily predictions
R_t
0
1
2
3
2021−06−16
2021−07−21
2021−08−25

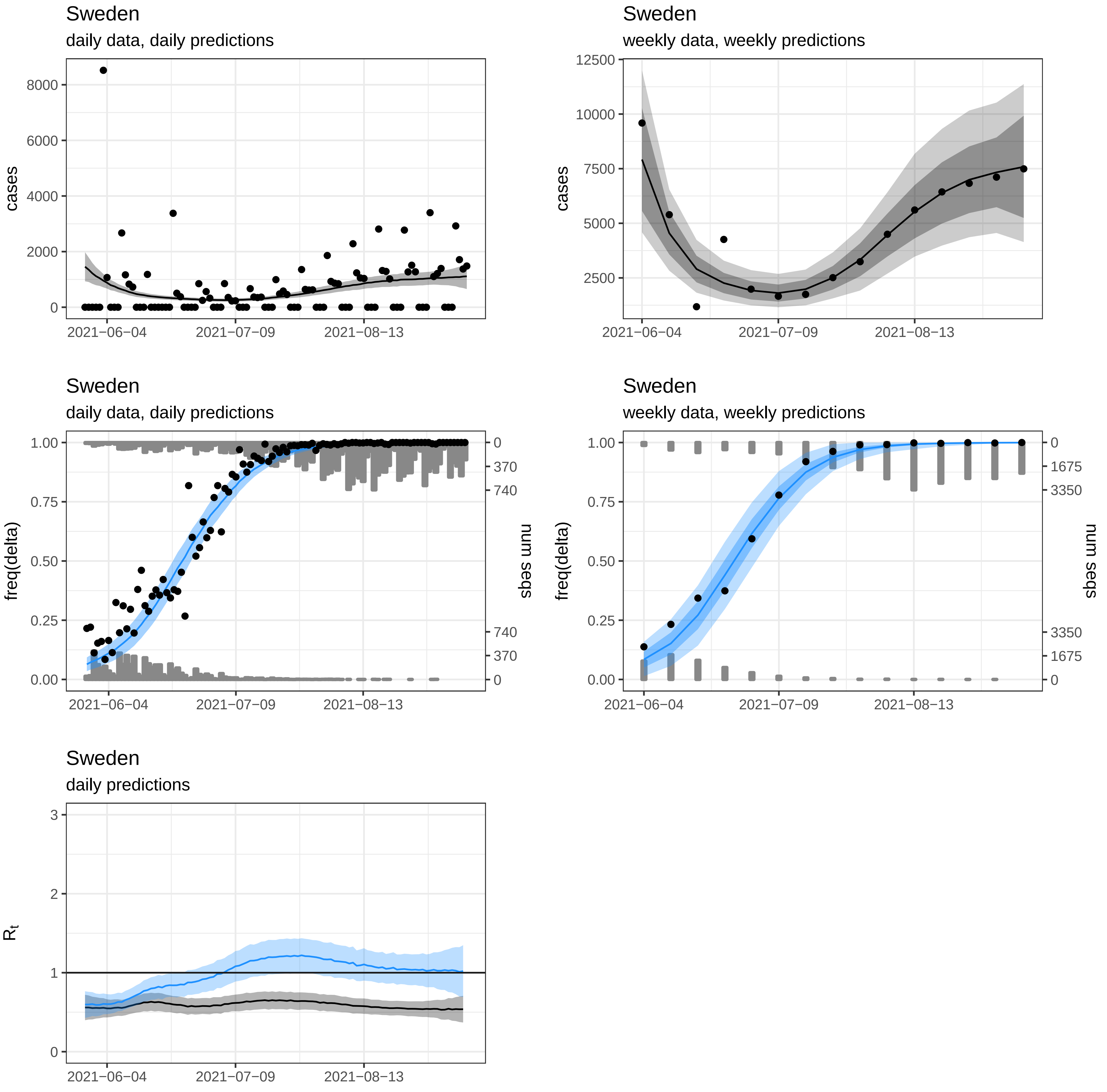

Sweden
daily data, daily predictions
cases
8000
6000
4000
2000
0
2021−06−04
2021−07−09
2021−08−13
Sweden
weekly data, weekly predictions
cases
12500
10000
7500
5000
2500
Sweden
daily data, daily predictions
freq(delta)
num seqs
1.00
0.75
0.50
0.25
0.00
370
740
Sweden
weekly data, weekly predictions
1675
3350
Sweden
daily predictions
R_t
3
2
1
0

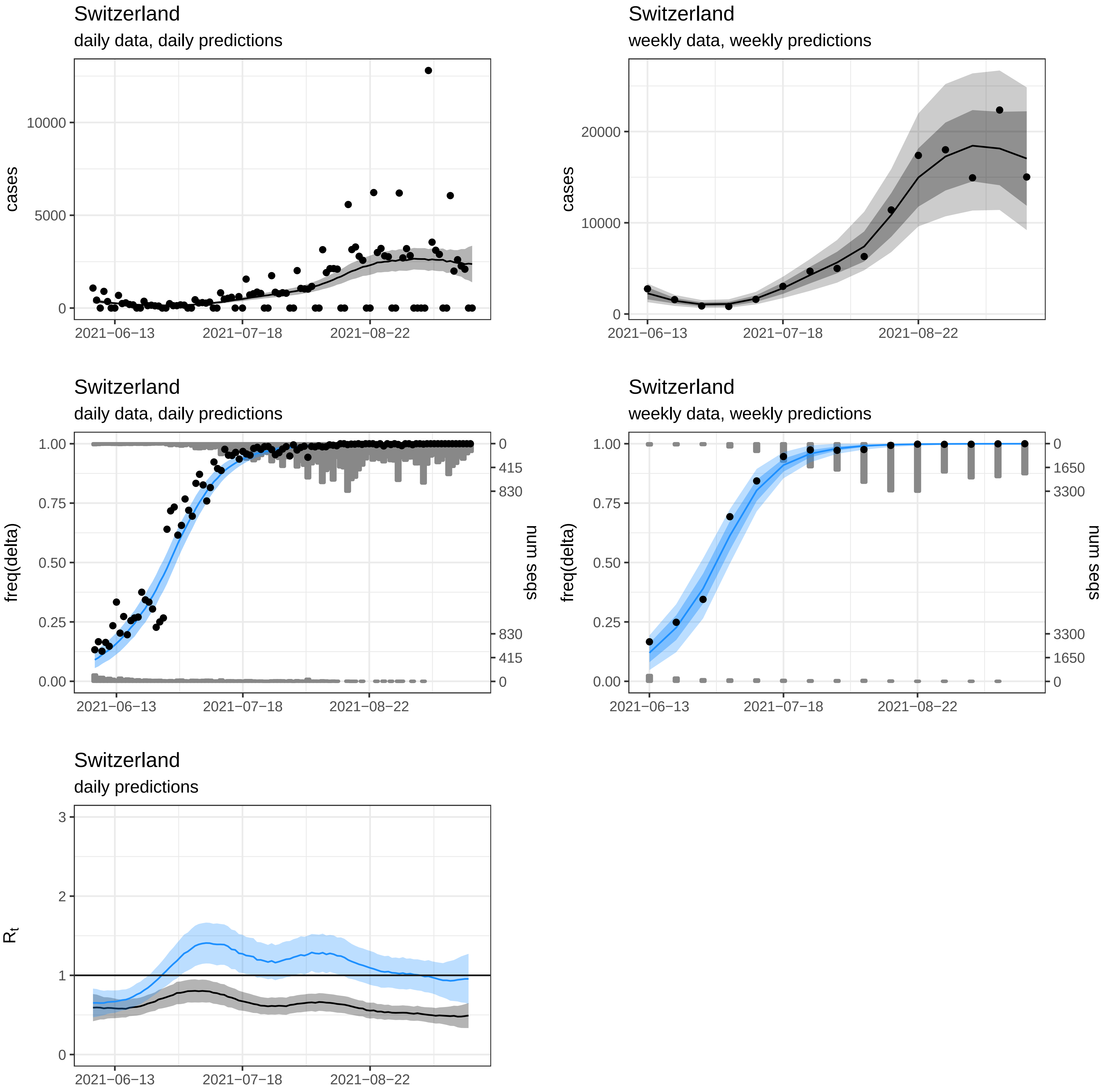
Switzerland
daily data, daily predictions
cases
10000
5000
0
2021−06−13
2021−07−18
2021−08−22
Switzerland
weekly data, weekly predictions
cases
20000
10000
0
2021−06−13
2021−07−18
2021−08−22
Switzerland
daily data, daily predictions
freq(delta)
1.00
0.75
0.50
0.25
0.00
num seqs
0
415
830
830
415
0
2021−06−13
2021−07−18
2021−08−22
Switzerland
weekly data, weekly predictions
freq(delta)
1.00
0.75
0.50
0.25
0.00
num seqs
0
1650
3300
3300
1650
0
2021−06−13
2021−07−18
2021−08−22
Switzerland
daily predictions
R_t
3
2
1
0
2021−06−13
2021−07−18
2021−08−22

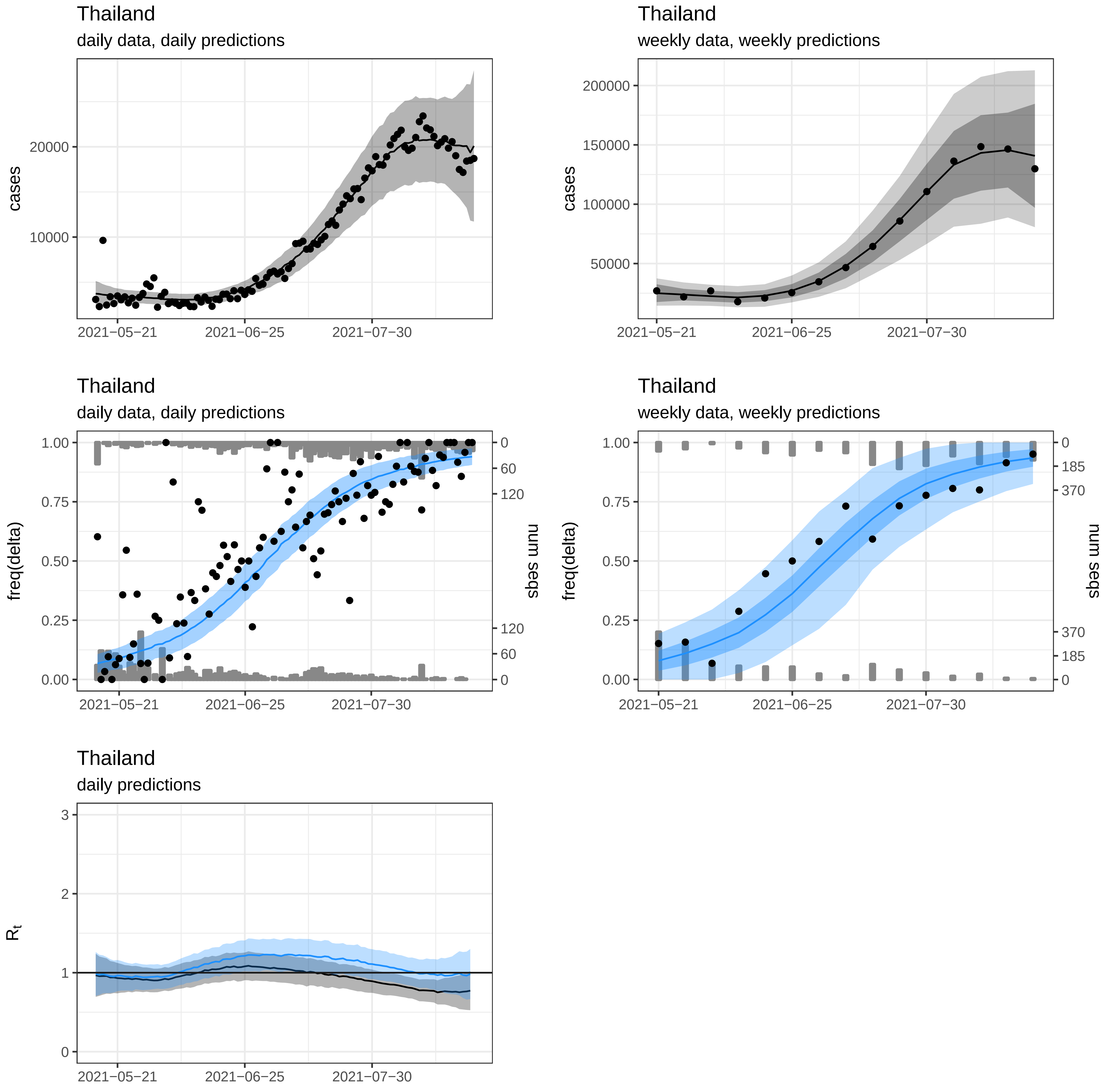
Thailand
daily data, daily predictions
cases
20000
10000
2021−05−21
2021−06−25
2021−07−30
Thailand
weekly data, weekly predictions
cases
200000
150000
100000
50000
2021−05−21
2021−06−25
2021−07−30
Thailand
daily data, daily predictions
freq(delta)
1.00
0.75
0.50
0.25
0.00
num seqs
0
60
120
120
60
0
2021−05−21
2021−06−25
2021−07−30
Thailand
weekly data, weekly predictions
freq(delta)
1.00
0.75
0.50
0.25
0.00
num seqs
0
185
370
370
185
0
2021−05−21
2021−06−25
2021−07−30
Thailand
daily predictions
R_t
3
2
1
0
2021−05−21
2021−06−25
2021−07−30

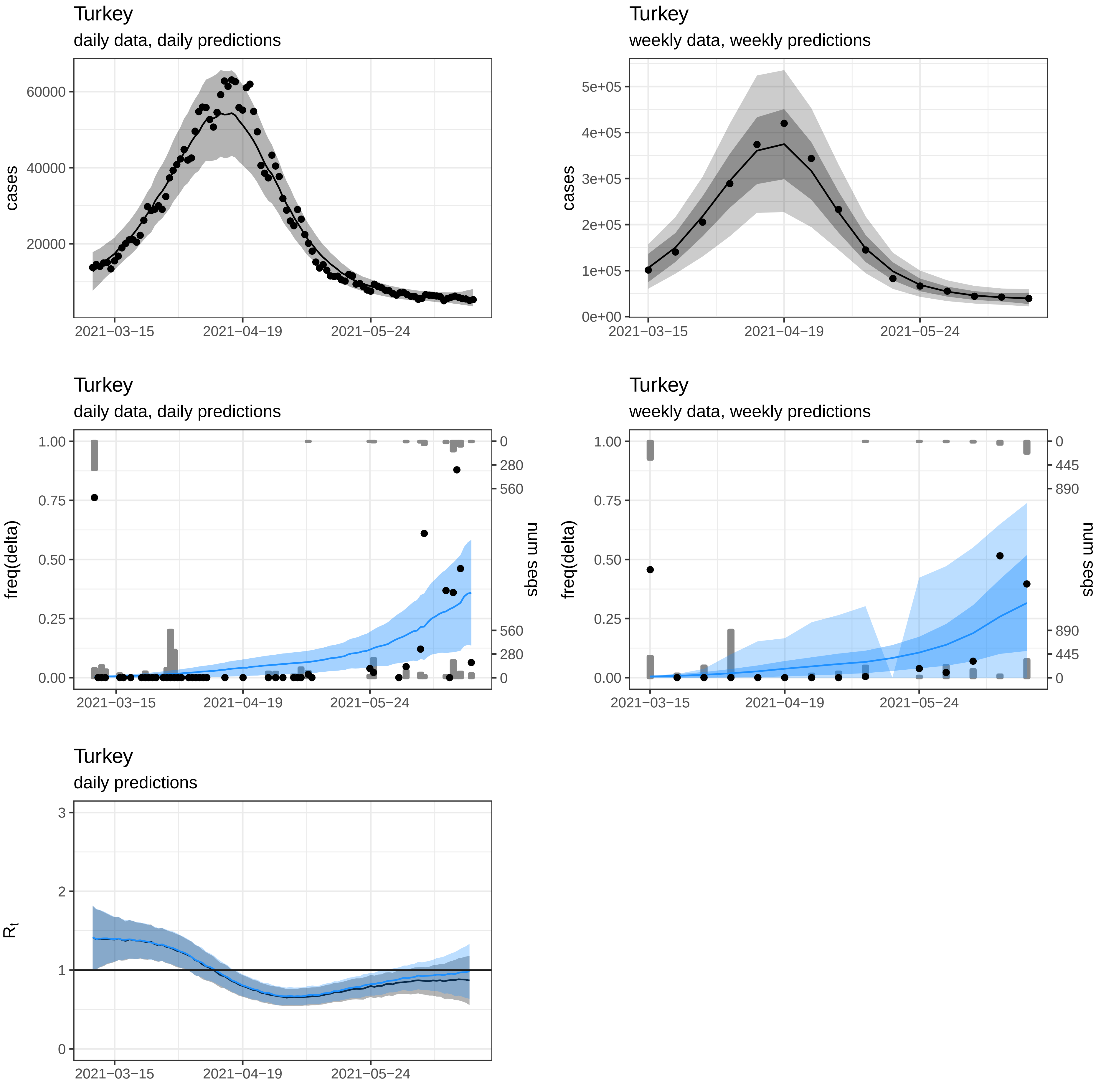
Turkey
daily data, daily predictions
cases
60000
40000
20000
2021−03−15
2021−04−19
2021−05−24
Turkey
weekly data, weekly predictions
cases
5e+05
4e+05
3e+05
2e+05
1e+05
0e+00
2021−03−15
2021−04−19
2021−05−24
Turkey
daily data, daily predictions
freq(delta)
1.00
0.75
0.50
0.25
0.00
num seqs
0
280
560
560
280
0
2021−03−15
2021−04−19
2021−05−24
Turkey
weekly data, weekly predictions
freq(delta)
1.00
0.75
0.50
0.25
0.00
num seqs
0
445
890
890
445
0
2021−03−15
2021−04−19
2021−05−24
Turkey
daily predictions
R_t
3
2
1
0
2021−03−15
2021−04−19
2021−05−24

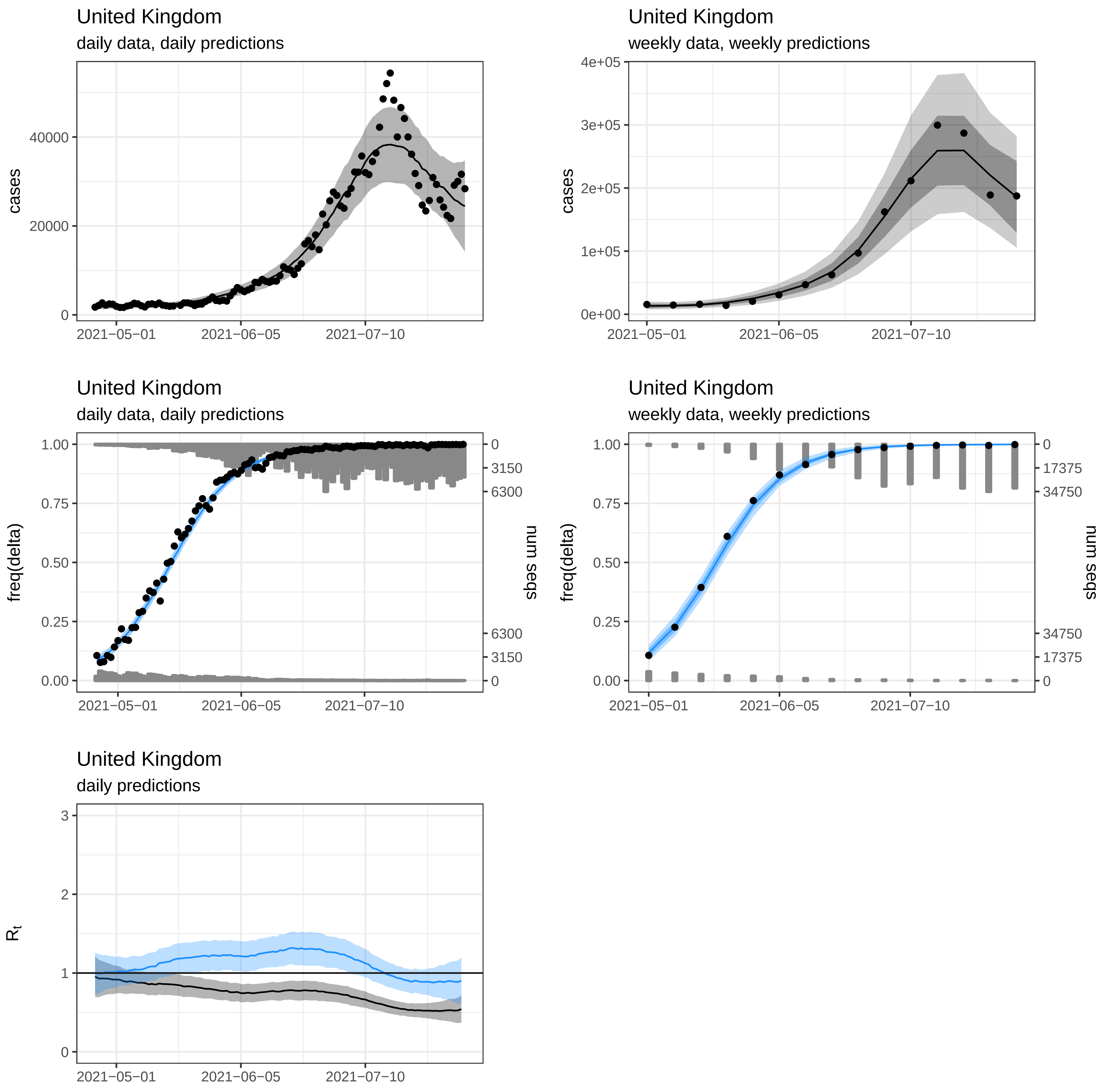
United Kingdom
daily data, daily predictions
cases
0
20000
40000
2021−05−01
2021−06−05
2021−07−10
United Kingdom
weekly data, weekly predictions
cases
0e+00
1e+05
2e+05
3e+05
4e+05
2021−05−01
2021−06−05
2021−07−10
United Kingdom
daily data, daily predictions
freq(delta)
0.00
0.25
0.50
0.75
1.00
num seqs
0
3150
6300
6300
3150
0
2021−05−01
2021−06−05
2021−07−10
United Kingdom
weekly data, weekly predictions
freq(delta)
0.00
0.25
0.50
0.75
1.00
num seqs
0
17375
34750
34750
17375
0
2021−05−01
2021−06−05
2021−07−10
United Kingdom
daily predictions
R_t
0
1
2
3
2021−05−01
2021−06−05
2021−07−10

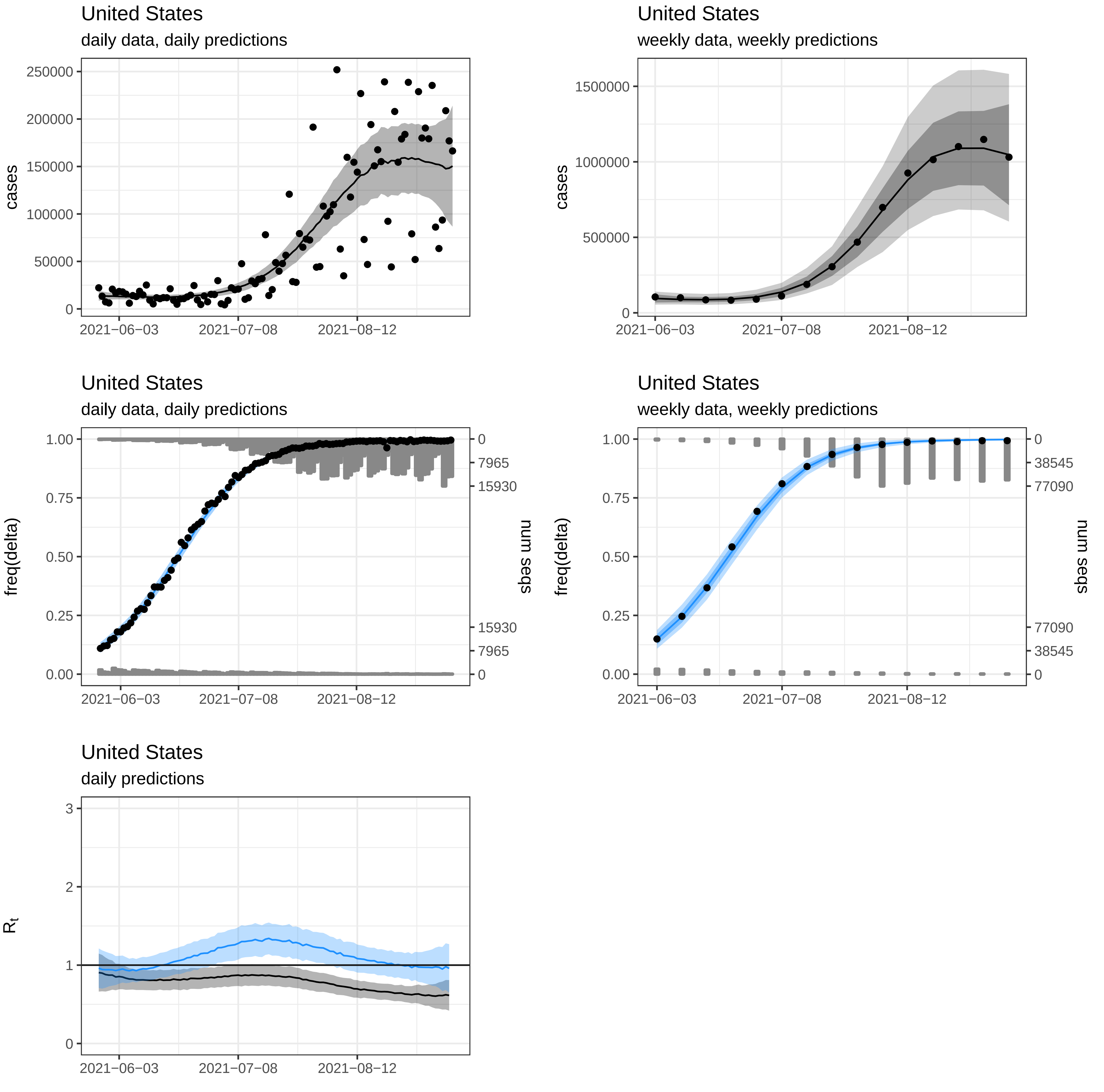
United States
daily data, daily predictions
cases
250000
200000
150000
100000
50000
0
2021−06−03
2021−07−08
2021−08−12
United States
weekly data, weekly predictions
cases
1500000
1000000
500000
0
2021−06−03
2021−07−08
2021−08−12
United States
daily data, daily predictions
freq(delta)
1.00
0.75
0.50
0.25
0.00
num seqs
0
7965
15930
15930
7965
0
2021−06−03
2021−07−08
2021−08−12
United States
weekly data, weekly predictions
freq(delta)
1.00
0.75
0.50
0.25
0.00
num seqs
0
38545
77090
77090
38545
0
2021−06−03
2021−07−08
2021−08−12
United States
daily predictions
R_t
3
2
1
0
2021−06−03
2021−07−08
2021−08−12
