# Supplementary material for: Global estimates of the fitness advantage of SARS-CoV-2 variant Omicron
Source: Virus Evol. 2022 Oct 7;8(2):veac089. doi: 10.1093/ve/veac089 (PMC9615435; doi:10.1093/ve/veac089)
Supplement: veac089_Supp [file veac089_supp.zip › suppl_data/omicron-fits.pdf]

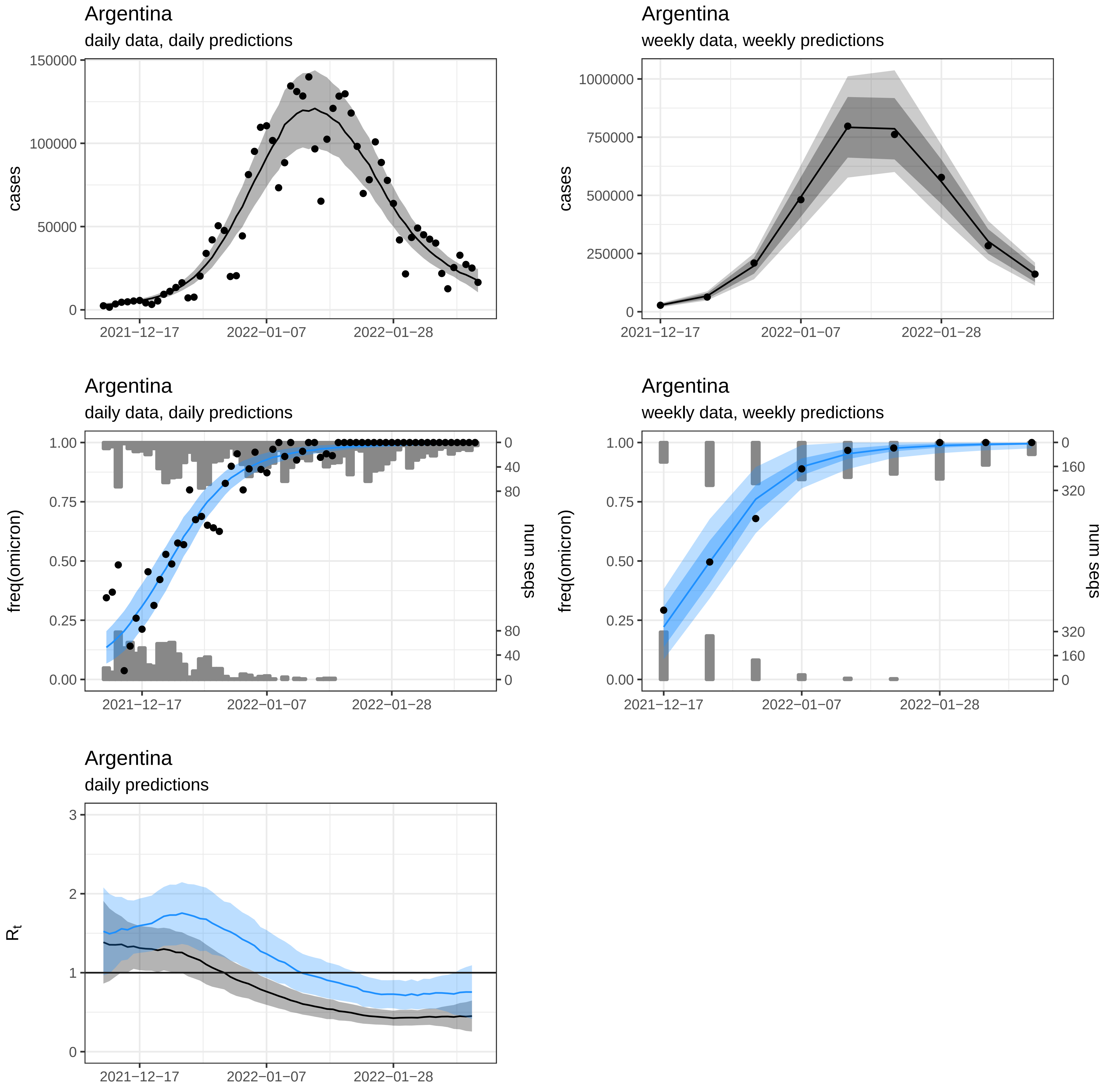
Argentina
daily data, daily predictions
cases
150000
100000
50000
0
2021−12−17
2022−01−07
2022−01−28
Argentina
weekly data, weekly predictions
cases
1000000
750000
500000
250000
0
2021−12−17
2022−01−07
2022−01−28
Argentina
daily data, daily predictions
freq(omicron)
1.00
0.75
0.50
0.25
0.00
num seqs
0
40
80
80
40
0
2021−12−17
2022−01−07
2022−01−28
Argentina
weekly data, weekly predictions
freq(omicron)
1.00
0.75
0.50
0.25
0.00
num seqs
0
160
320
320
160
0
2021−12−17
2022−01−07
2022−01−28
Argentina
daily predictions
R_t
3
2
1
0
2021−12−17
2022−01−07
2022−01−28

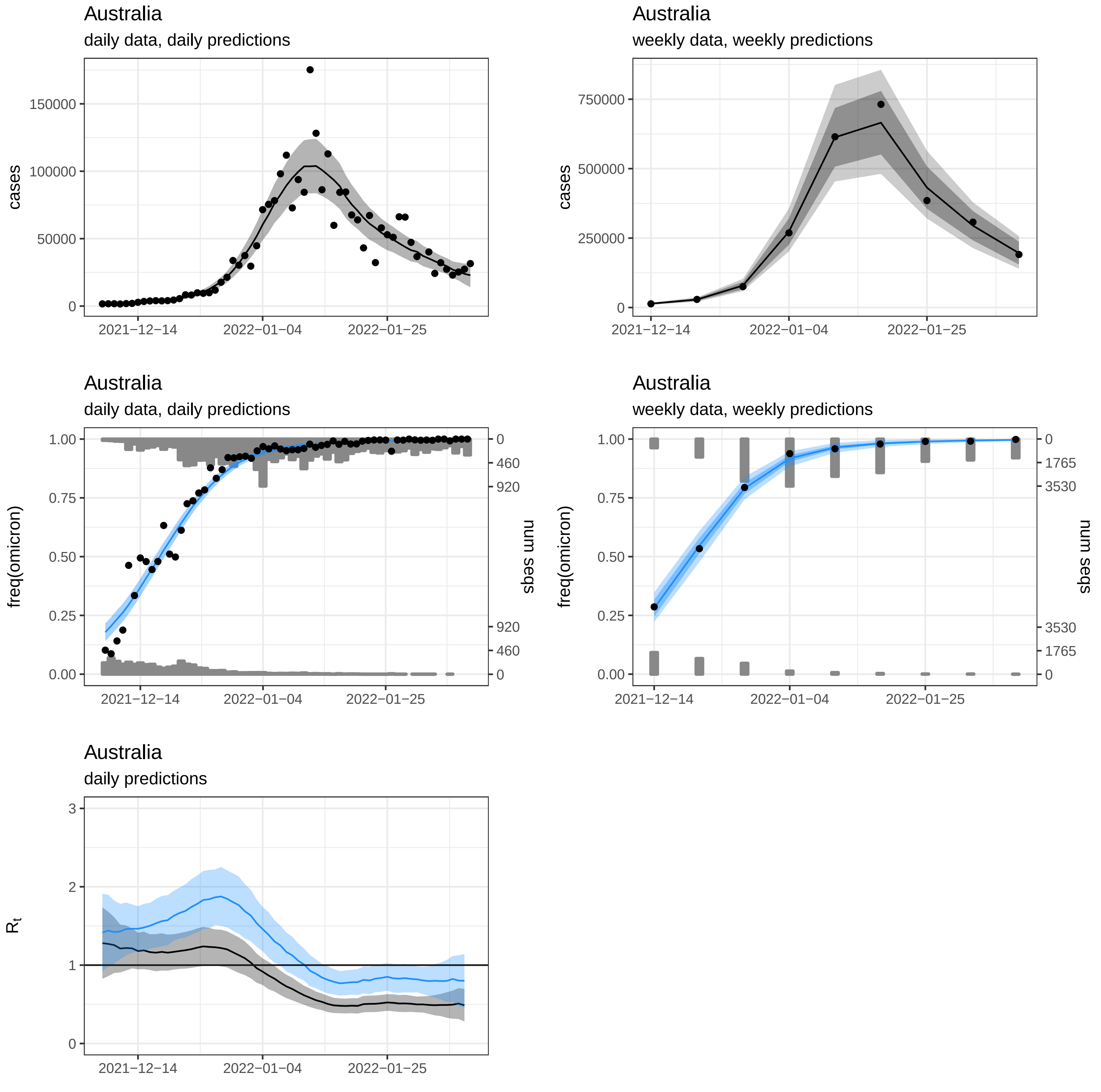
Australia
daily data, daily predictions
cases
150000
100000
50000
0
2021−12−14
2022−01−04
2022−01−25
Australia
weekly data, weekly predictions
cases
750000
500000
250000
0
2021−12−14
2022−01−04
2022−01−25
Australia
daily data, daily predictions
freq(omicron)
1.00
0.75
0.50
0.25
0.00
num seqs
0
460
920
920
460
0
2021−12−14
2022−01−04
2022−01−25
Australia
weekly data, weekly predictions
freq(omicron)
1.00
0.75
0.50
0.25
0.00
num seqs
0
1765
3530
3530
1765
0
2021−12−14
2022−01−04
2022−01−25
Australia
daily predictions
R_t
3
2
1
0
2021−12−14
2022−01−04
2022−01−25

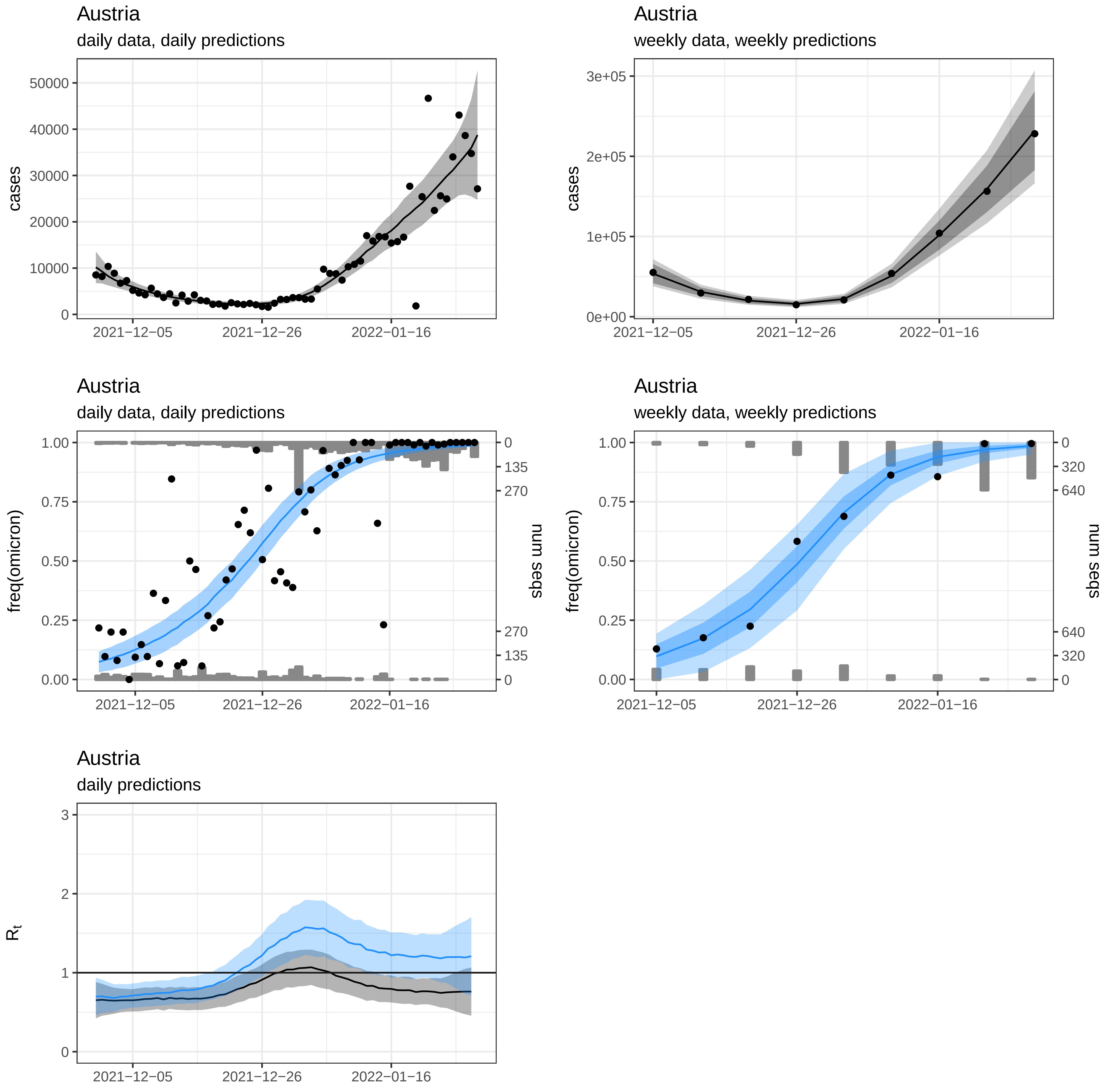

Austria
daily data, daily predictions
cases
50000
40000
30000
20000
10000
0
2021−12−05
2021−12−26
2022−01−16
Austria
weekly data, weekly predictions
cases
3e+05
2e+05
1e+05
0e+00
2021−12−05
2021−12−26
2022−01−16
Austria
daily data, daily predictions
freq(omicron)
1.00
0.75
0.50
0.25
0.00
num seqs
0
135
270
270
135
0
2021−12−05
2021−12−26
2022−01−16
Austria
weekly data, weekly predictions
freq(omicron)
1.00
0.75
0.50
0.25
0.00
num seqs
0
320
640
640
320
0
2021−12−05
2021−12−26
2022−01−16
Austria
daily predictions
R_t
3
2
1
0
2021−12−05
2021−12−26
2022−01−16

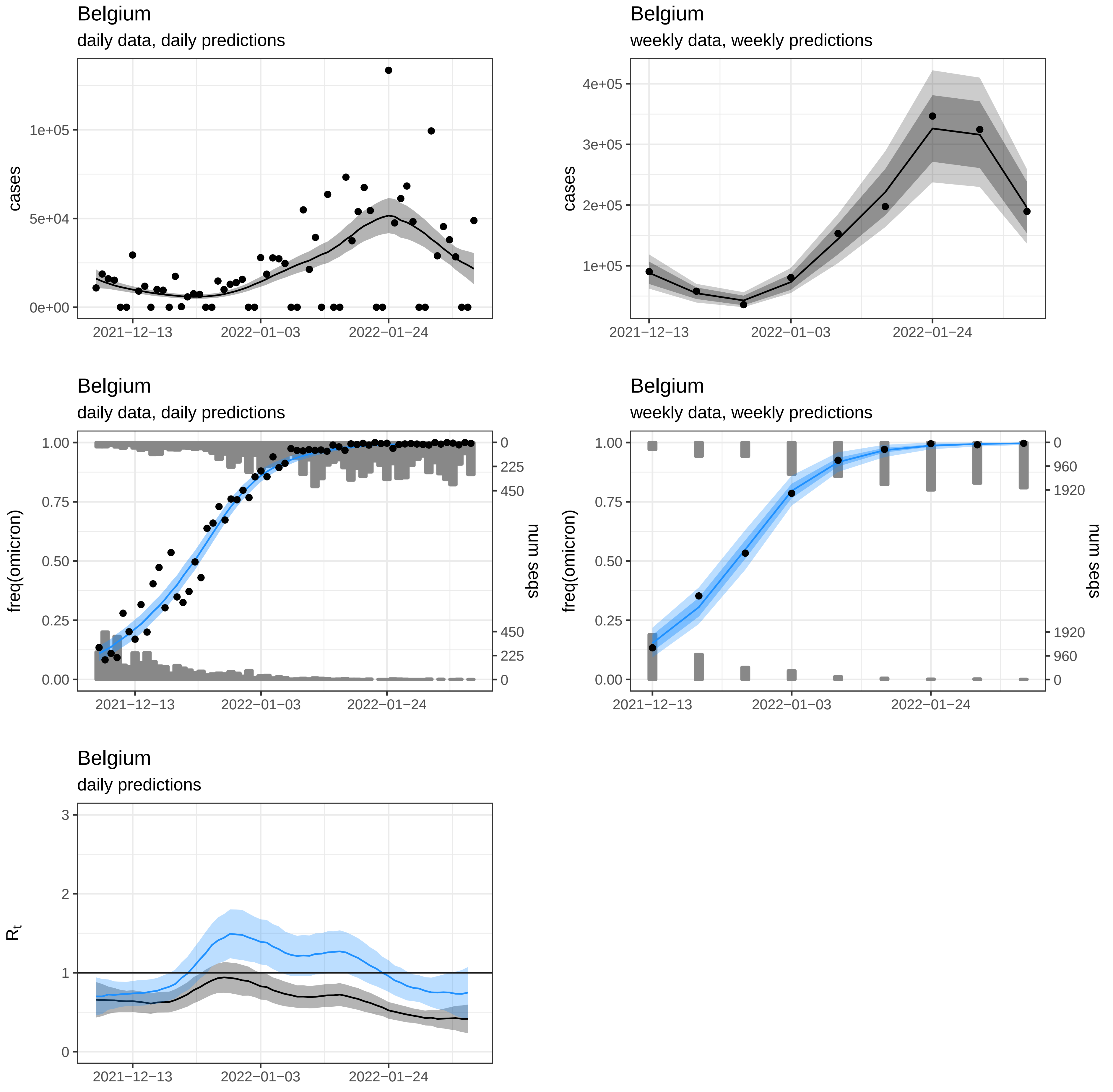
Belgium
daily data, daily predictions
cases
1e+05
5e+04
0e+00
2021−12−13
2022−01−03
2022−01−24
Belgium
weekly data, weekly predictions
cases
4e+05
3e+05
2e+05
1e+05
2021−12−13
2022−01−03
2022−01−24
Belgium
daily data, daily predictions
freq(omicron)
1.00
0.75
0.50
0.25
0.00
num seqs
0
225
450
450
225
0
2021−12−13
2022−01−03
2022−01−24
Belgium
weekly data, weekly predictions
freq(omicron)
1.00
0.75
0.50
0.25
0.00
num seqs
0
960
1920
1920
960
0
2021−12−13
2022−01−03
2022−01−24
Belgium
daily predictions
R_t
3
2
1
0
2021−12−13
2022−01−03
2022−01−24

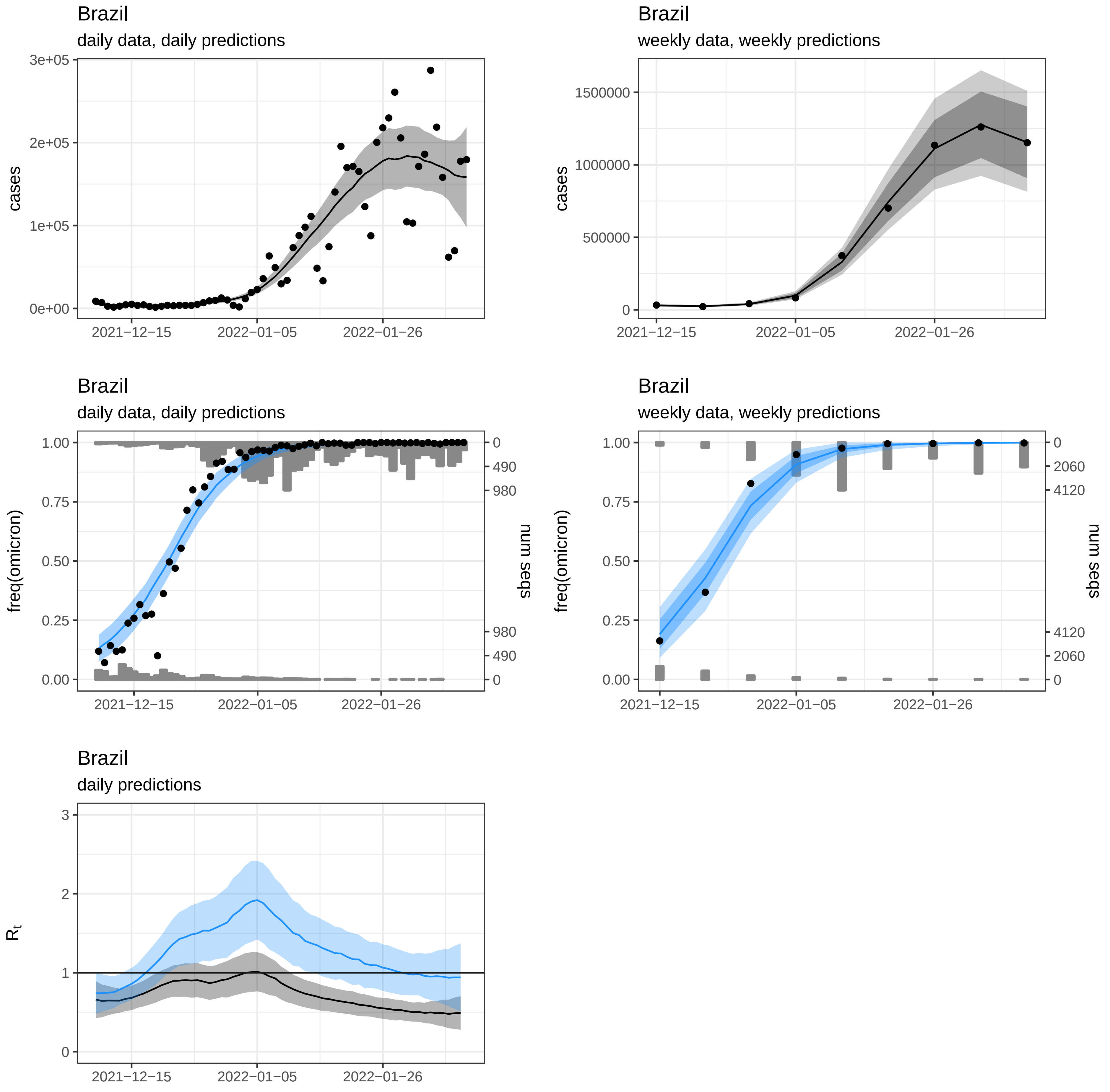

Brazil
daily data, daily predictions
cases
3e+05
2e+05
1e+05
0e+00
2021−12−15
2022−01−05
2022−01−26
Brazil
weekly data, weekly predictions
cases
1500000
1000000
500000
0
2021−12−15
2022−01−05
2022−01−26
Brazil
daily data, daily predictions
freq(omicron)
1.00
0.75
0.50
0.25
0.00
num seqs
0
490
980
980
490
0
2021−12−15
2022−01−05
2022−01−26
Brazil
weekly data, weekly predictions
freq(omicron)
1.00
0.75
0.50
0.25
0.00
num seqs
0
2060
4120
4120
2060
0
2021−12−15
2022−01−05
2022−01−26
Brazil
daily predictions
R_t
3
2
1
0
2021−12−15
2022−01−05
2022−01−26

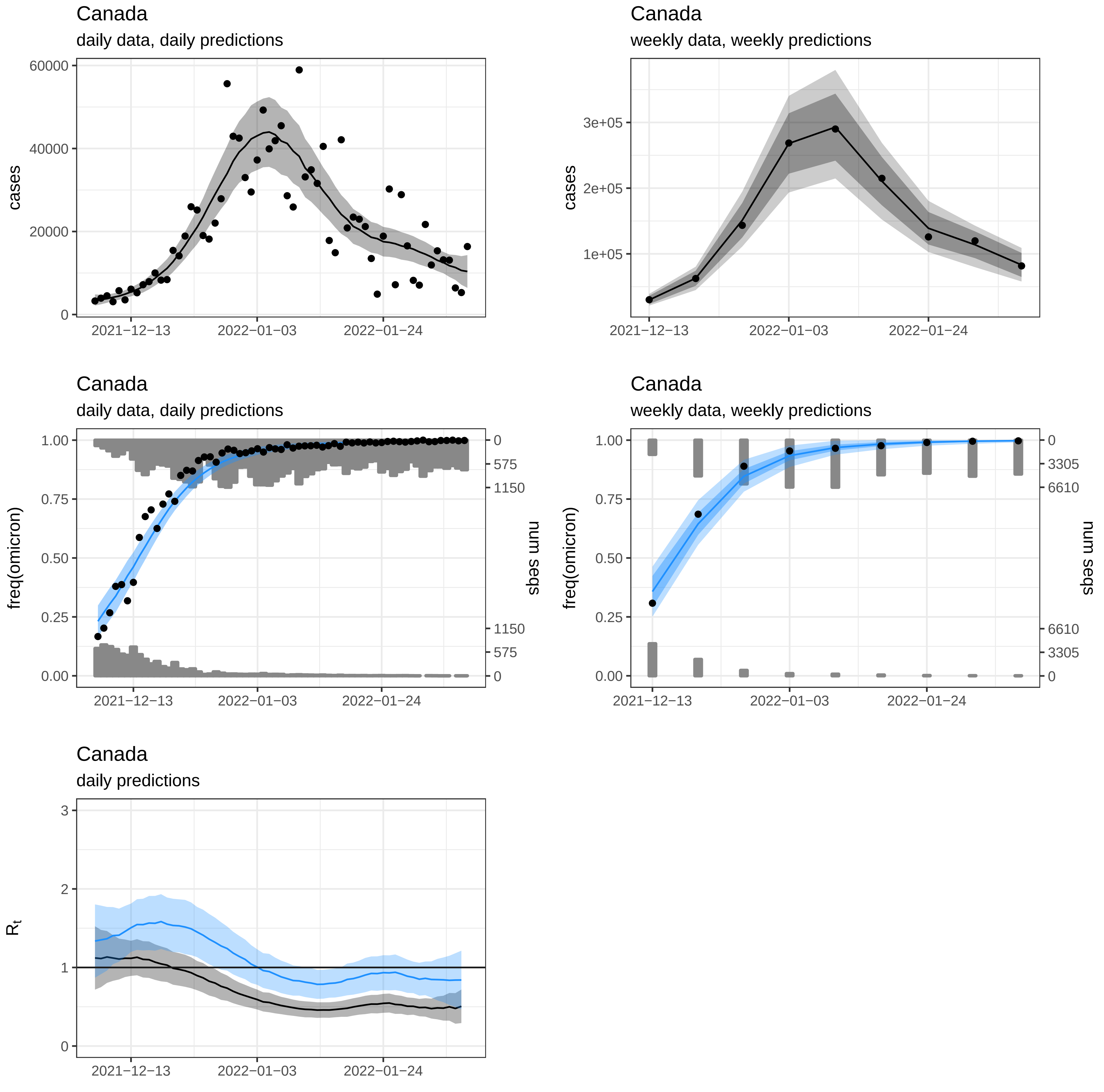
Canada
daily data, daily predictions
cases
60000
40000
20000
0
2021−12−13
2022−01−03
2022−01−24
Canada
weekly data, weekly predictions
cases
3e+05
2e+05
1e+05
2021−12−13
2022−01−03
2022−01−24
Canada
daily data, daily predictions
freq(omicron)
1.00
0.75
0.50
0.25
0.00
num seqs
0
575
1150
1150
575
0
2021−12−13
2022−01−03
2022−01−24
Canada
weekly data, weekly predictions
freq(omicron)
1.00
0.75
0.50
0.25
0.00
num seqs
0
3305
6610
6610
3305
0
2021−12−13
2022−01−03
2022−01−24
Canada
daily predictions
R_t
3
2
1
0
2021−12−13
2022−01−03
2022−01−24

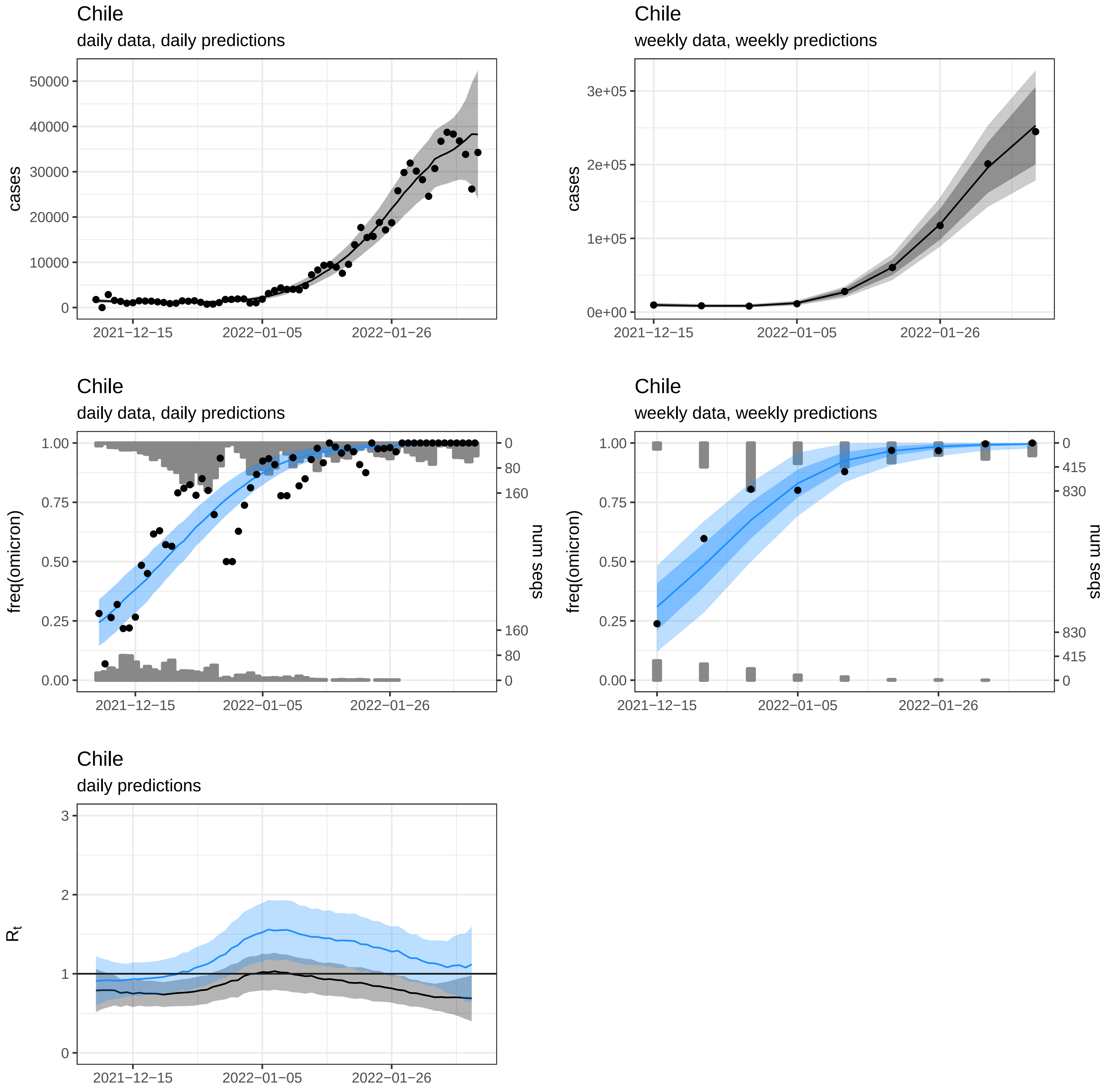
Chile
daily data, daily predictions
cases
50000
40000
30000
20000
10000
0
2021−12−15
2022−01−05
2022−01−26
Chile
weekly data, weekly predictions
cases
3e+05
2e+05
1e+05
0e+00
2021−12−15
2022−01−05
2022−01−26
Chile
daily data, daily predictions
freq(omicron)
1.00
0.75
0.50
0.25
0.00
num seqs
0
80
160
160
80
0
2021−12−15
2022−01−05
2022−01−26
Chile
weekly data, weekly predictions
freq(omicron)
1.00
0.75
0.50
0.25
0.00
num seqs
0
415
830
830
415
0
2021−12−15
2022−01−05
2022−01−26
Chile
daily predictions
R_t
3
2
1
0
2021−12−15
2022−01−05
2022−01−26

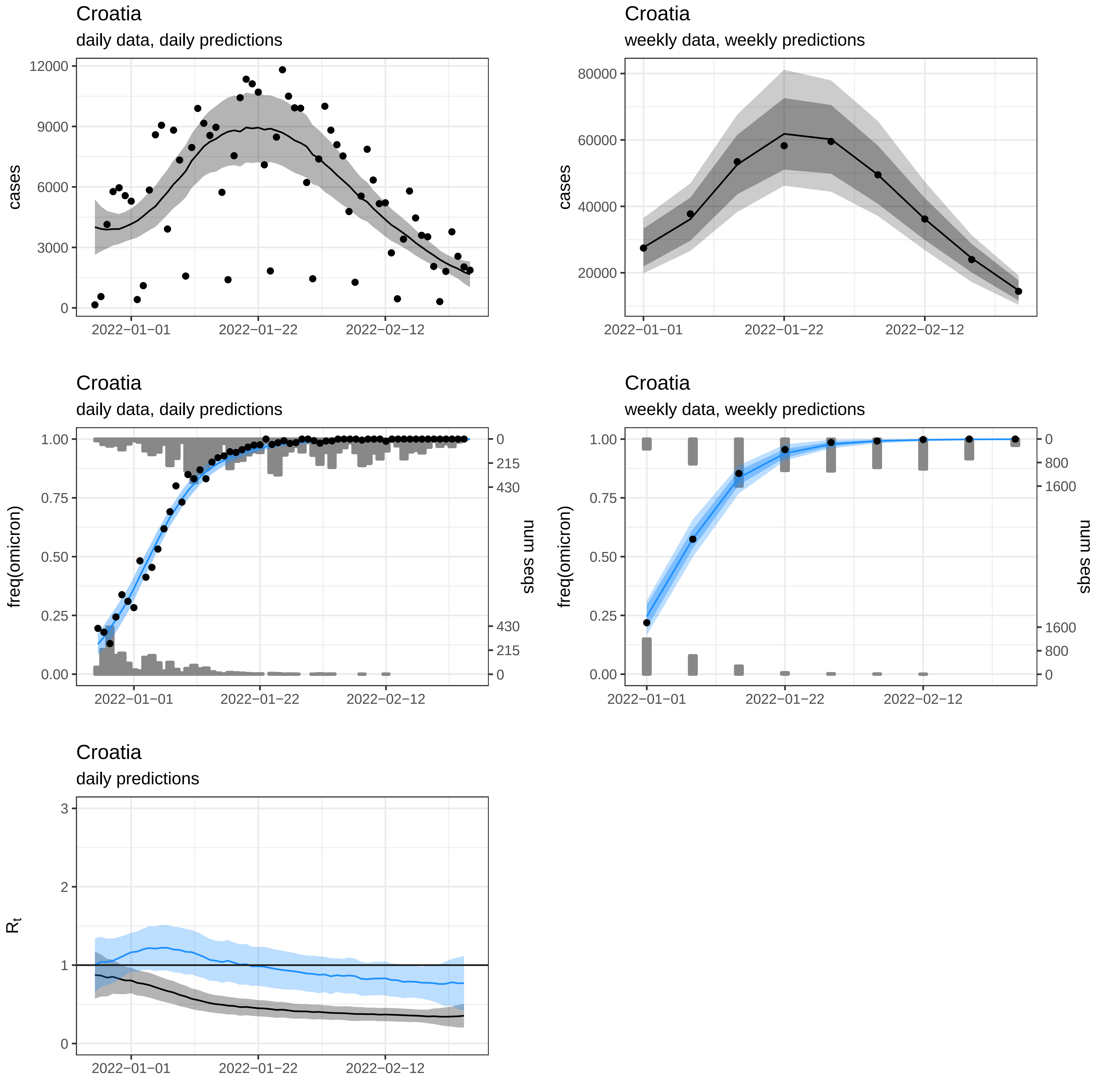

Croatia
daily data, daily predictions
cases
12000
9000
6000
3000
0
2022−01−01
2022−01−22
2022−02−12
Croatia
weekly data, weekly predictions
cases
80000
60000
40000
20000
2022−01−01
2022−01−22
2022−02−12
Croatia
daily data, daily predictions
freq(omicron)
1.00
0.75
0.50
0.25
0.00
num seqs
0
215
430
430
215
0
2022−01−01
2022−01−22
2022−02−12
Croatia
weekly data, weekly predictions
freq(omicron)
1.00
0.75
0.50
0.25
0.00
num seqs
0
800
1600
1600
800
0
2022−01−01
2022−01−22
2022−02−12
Croatia
daily predictions
R_t
3
2
1
0
2022−01−01
2022−01−22
2022−02−12

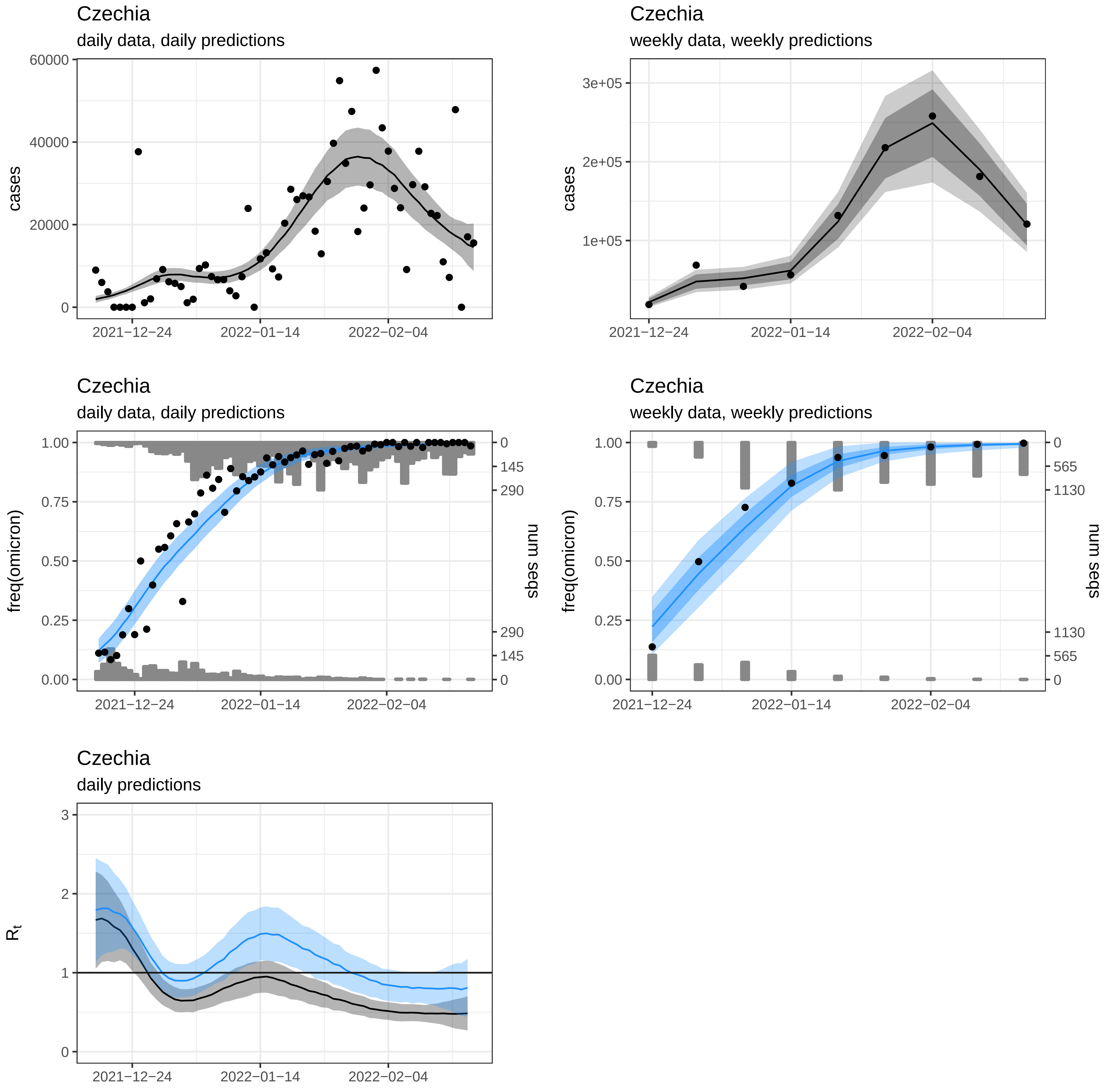

Czechia
daily data, daily predictions
cases
60000
40000
20000
0
2021−12−24
2022−01−14
2022−02−04
Czechia
weekly data, weekly predictions
cases
3e+05
2e+05
1e+05
2021−12−24
2022−01−14
2022−02−04
Czechia
daily data, daily predictions
freq(omicron)
1.00
0.75
0.50
0.25
0.00
num seqs
0
145
290
290
145
0
2021−12−24
2022−01−14
2022−02−04
Czechia
weekly data, weekly predictions
freq(omicron)
1.00
0.75
0.50
0.25
0.00
num seqs
0
565
1130
1130
565
0
2021−12−24
2022−01−14
2022−02−04
Czechia
daily predictions
R_t
3
2
1
0
2021−12−24
2022−01−14
2022−02−04

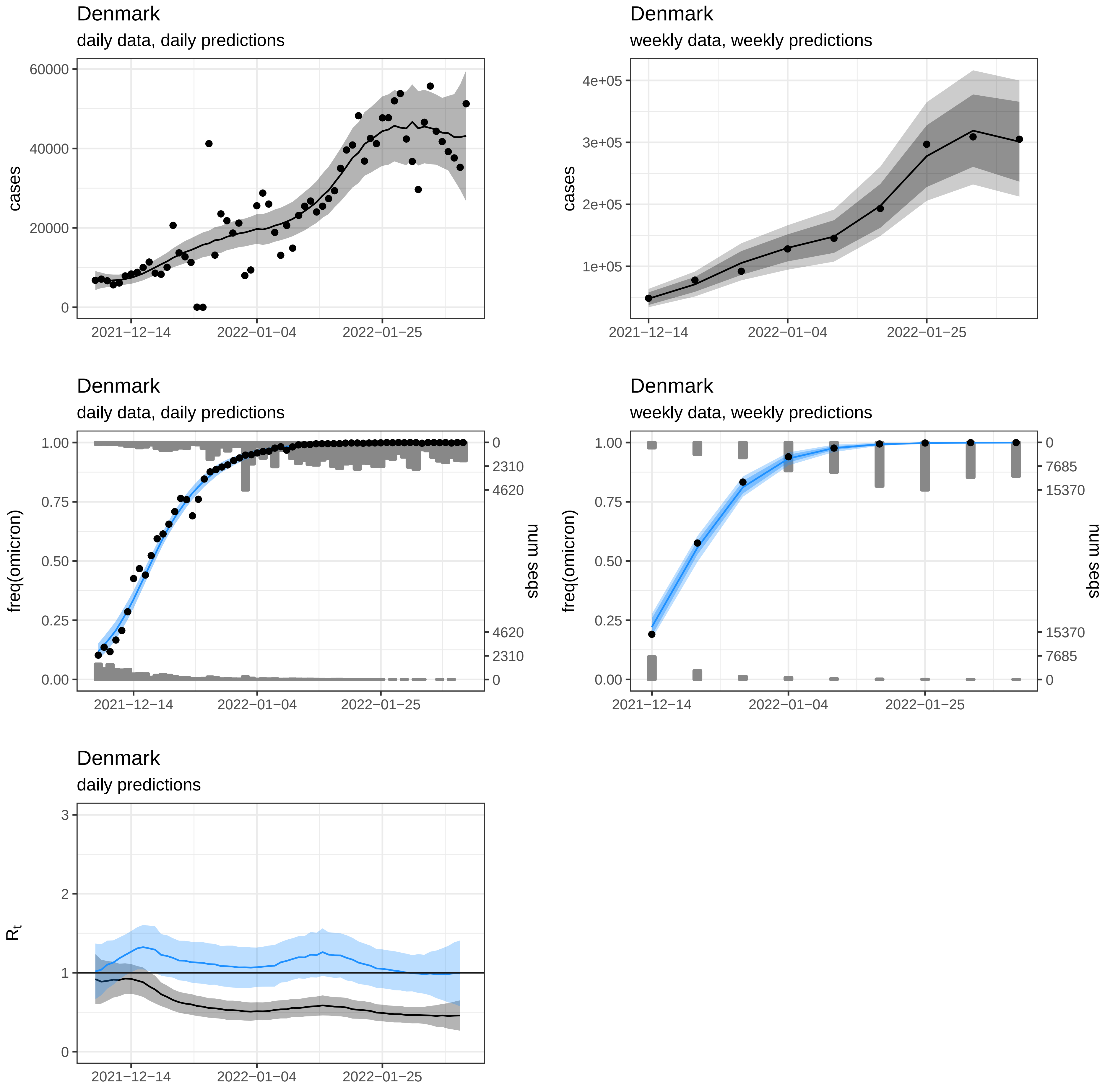
Denmark
daily data, daily predictions
cases
60000
40000
20000
0
2021−12−14
2022−01−04
2022−01−25
Denmark
weekly data, weekly predictions
cases
4e+05
3e+05
2e+05
1e+05
2021−12−14
2022−01−04
2022−01−25
Denmark
daily data, daily predictions
freq(omicron)
1.00
0.75
0.50
0.25
0.00
num seqs
0
2310
4620
4620
2310
0
2021−12−14
2022−01−04
2022−01−25
Denmark
weekly data, weekly predictions
freq(omicron)
1.00
0.75
0.50
0.25
0.00
num seqs
0
7685
15370
15370
7685
0
2021−12−14
2022−01−04
2022−01−25
Denmark
daily predictions
R_t
3
2
1
0
2021−12−14
2022−01−04
2022−01−25

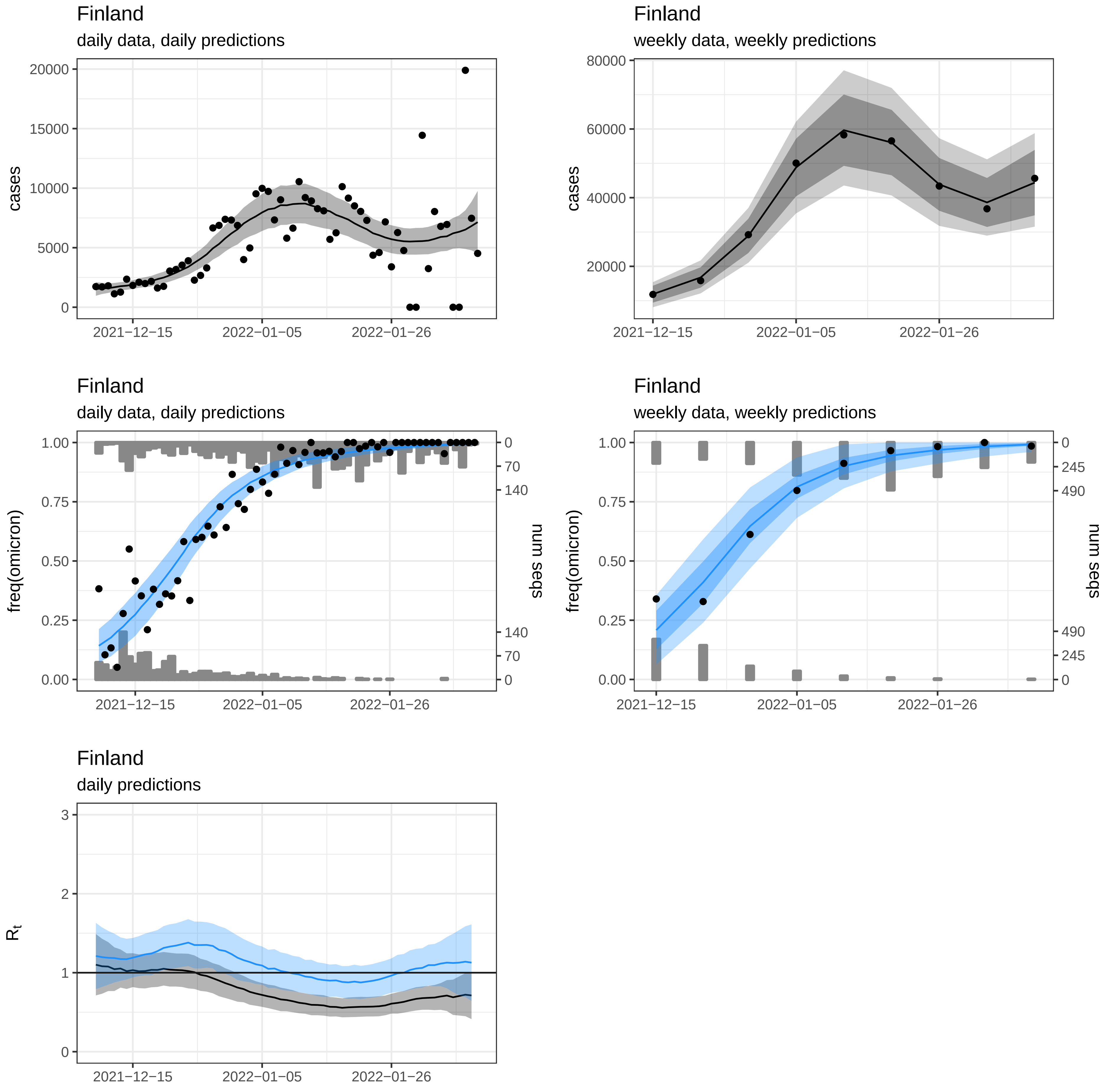
Finland
daily data, daily predictions
cases
20000
15000
10000
5000
0
2021−12−15
2022−01−05
2022−01−26
Finland
weekly data, weekly predictions
cases
80000
60000
40000
20000
2021−12−15
2022−01−05
2022−01−26
Finland
daily data, daily predictions
freq(omicron)
1.00
0.75
0.50
0.25
0.00
num seqs
0
70
140
140
70
0
2021−12−15
2022−01−05
2022−01−26
Finland
weekly data, weekly predictions
freq(omicron)
1.00
0.75
0.50
0.25
0.00
num seqs
0
245
490
490
245
0
2021−12−15
2022−01−05
2022−01−26
Finland
daily predictions
R_t
3
2
1
0
2021−12−15
2022−01−05
2022−01−26

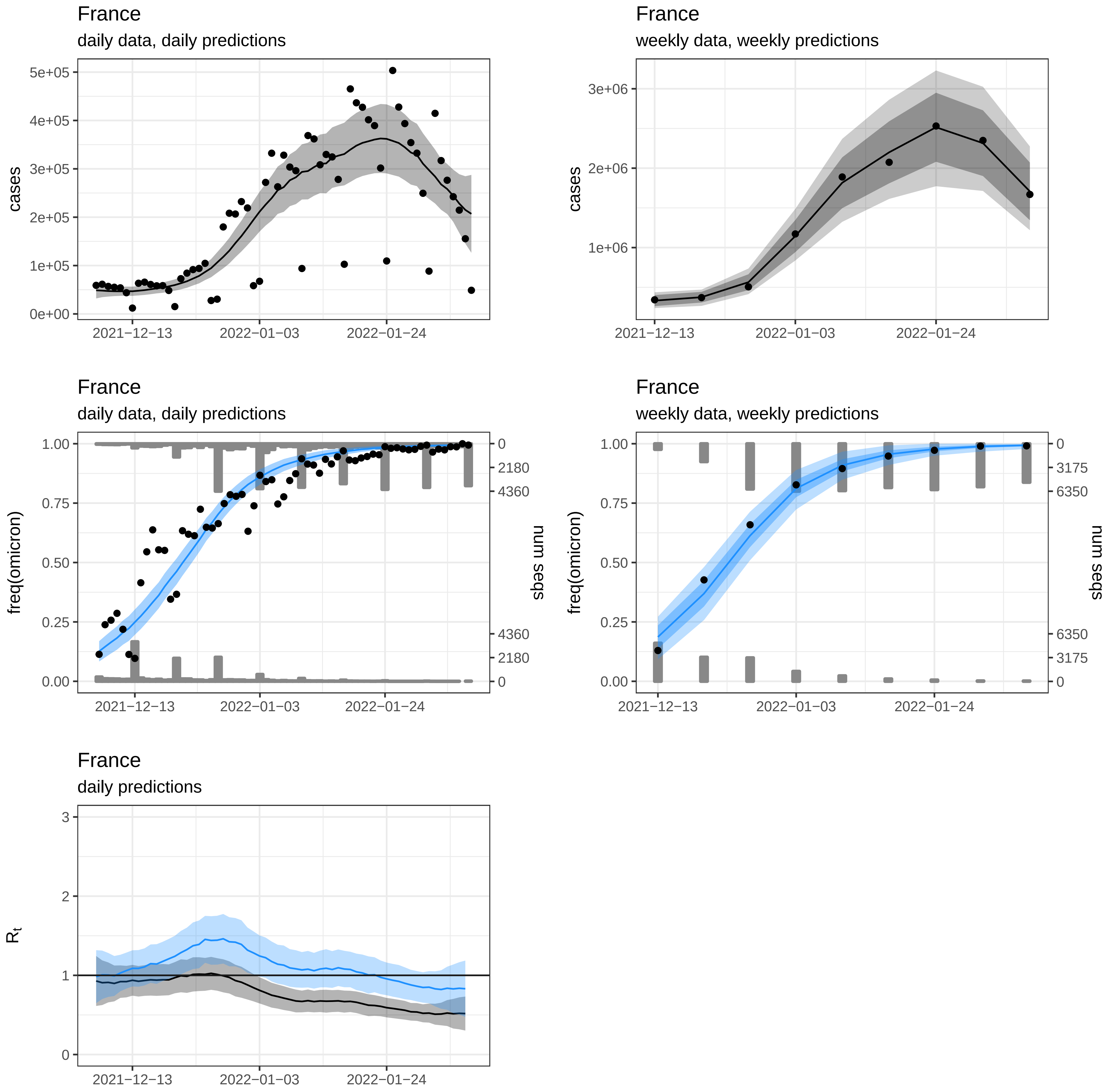
France
daily data, daily predictions
cases
5e+05
4e+05
3e+05
2e+05
1e+05
0e+00
2021−12−13
2022−01−03
2022−01−24
France
weekly data, weekly predictions
cases
3e+06
2e+06
1e+06
2021−12−13
2022−01−03
2022−01−24
France
daily data, daily predictions
freq(omicron)
1.00
0.75
0.50
0.25
0.00
num seqs
0
2180
4360
4360
2180
0
2021−12−13
2022−01−03
2022−01−24
France
weekly data, weekly predictions
freq(omicron)
1.00
0.75
0.50
0.25
0.00
num seqs
0
3175
6350
6350
3175
0
2021−12−13
2022−01−03
2022−01−24
France
daily predictions
R_t
3
2
1
0
2021−12−13
2022−01−03
2022−01−24

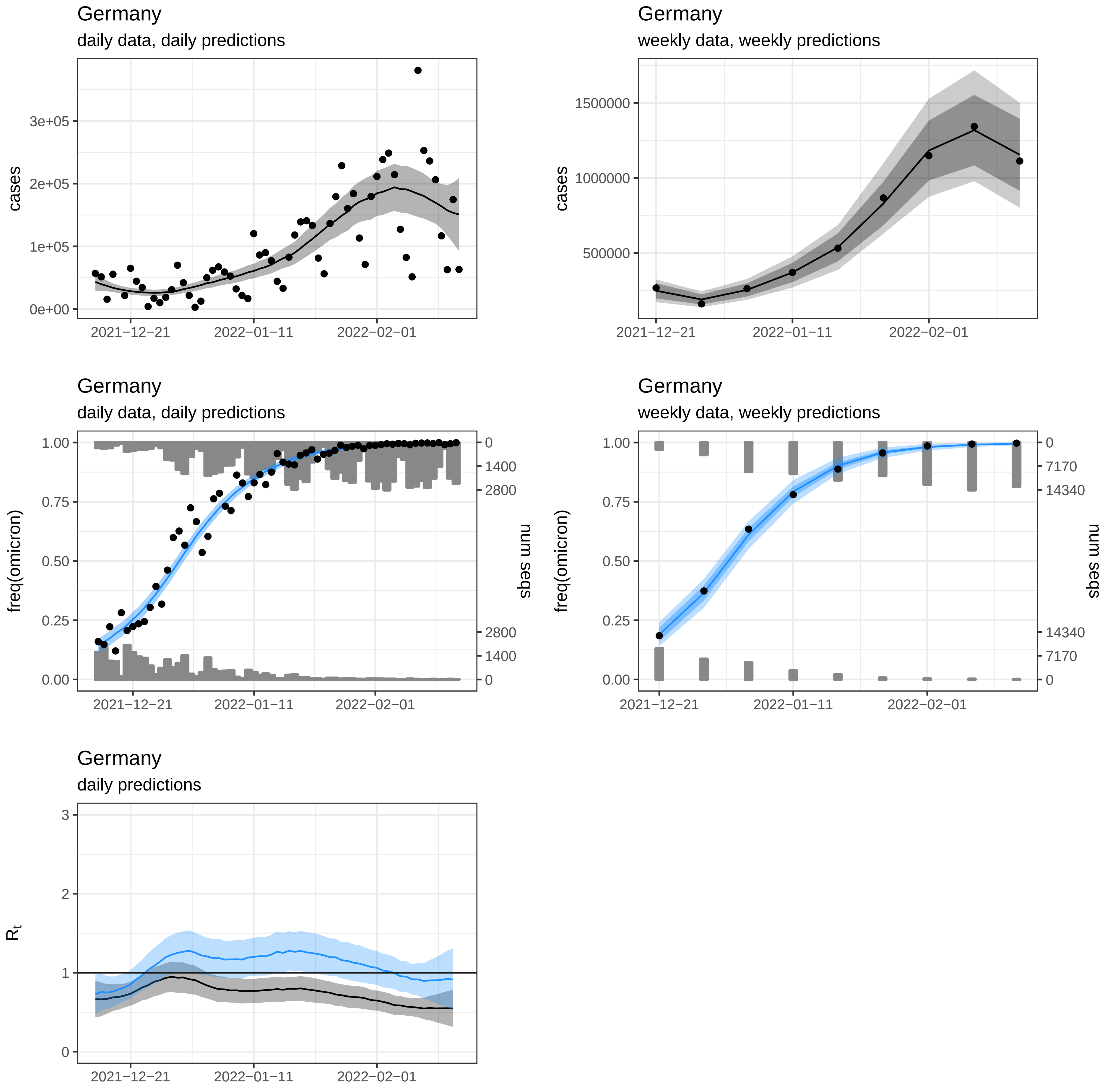
Germany
daily data, daily predictions
cases
3e+05
2e+05
1e+05
0e+00
2021−12−21
2022−01−11
2022−02−01
Germany
weekly data, weekly predictions
cases
1500000
1000000
500000
2021−12−21
2022−01−11
2022−02−01
Germany
daily data, daily predictions
freq(omicron)
1.00
0.75
0.50
0.25
0.00
num seqs
0
1400
2800
2800
1400
0
2021−12−21
2022−01−11
2022−02−01
Germany
weekly data, weekly predictions
freq(omicron)
1.00
0.75
0.50
0.25
0.00
num seqs
0
7170
14340
14340
7170
0
2021−12−21
2022−01−11
2022−02−01
Germany
daily predictions
R_t
3
2
1
0
2021−12−21
2022−01−11
2022−02−01

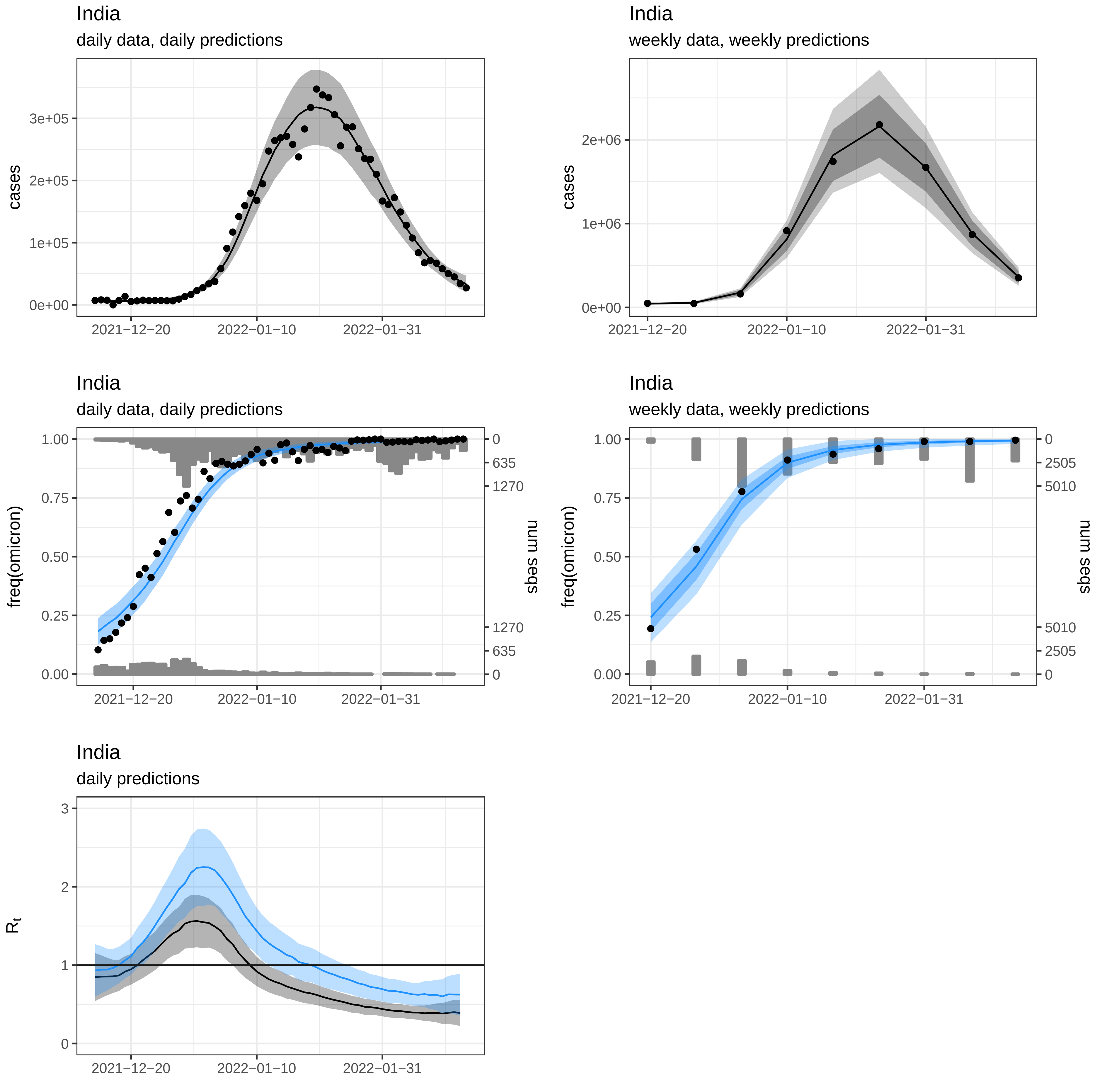
India
daily data, daily predictions
cases
3e+05
2e+05
1e+05
0e+00
2021−12−20
2022−01−10
2022−01−31
India
weekly data, weekly predictions
cases
2e+06
1e+06
0e+00
2021−12−20
2022−01−10
2022−01−31
India
daily data, daily predictions
freq(omicron)
1.00
0.75
0.50
0.25
0.00
num seqs
0
635
1270
1270
635
0
2021−12−20
2022−01−10
2022−01−31
India
weekly data, weekly predictions
freq(omicron)
1.00
0.75
0.50
0.25
0.00
num seqs
0
2505
5010
5010
2505
0
2021−12−20
2022−01−10
2022−01−31
India
daily predictions
R_t
3
2
1
0
2021−12−20
2022−01−10
2022−01−31

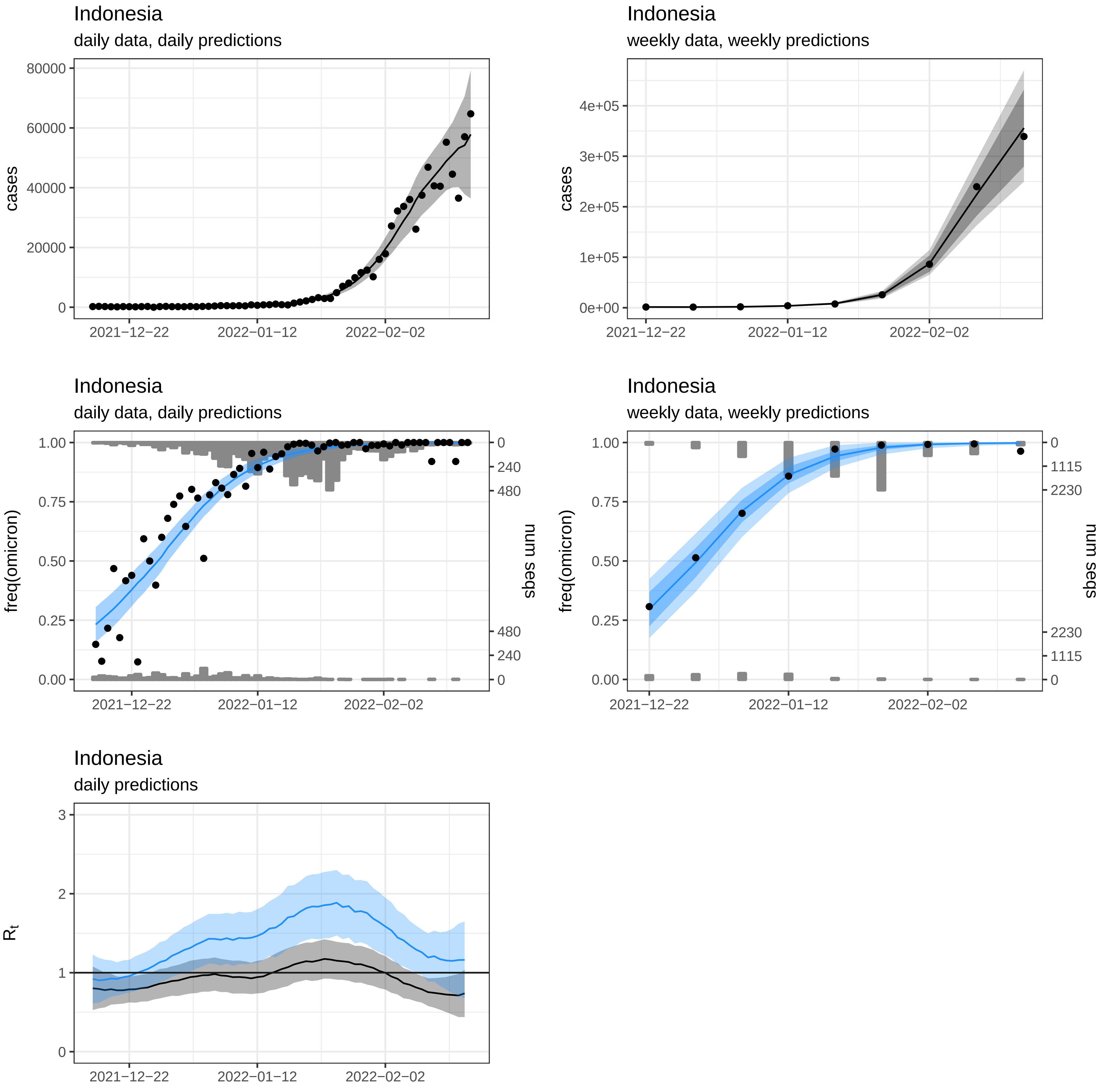
Indonesia
daily data, daily predictions
cases
80000
60000
40000
20000
0
2021−12−22
2022−01−12
2022−02−02
Indonesia
weekly data, weekly predictions
cases
4e+05
3e+05
2e+05
1e+05
0e+00
2021−12−22
2022−01−12
2022−02−02
Indonesia
daily data, daily predictions
freq(omicron)
1.00
0.75
0.50
0.25
0.00
num seqs
0
240
480
480
240
0
2021−12−22
2022−01−12
2022−02−02
Indonesia
weekly data, weekly predictions
freq(omicron)
1.00
0.75
0.50
0.25
0.00
num seqs
0
1115
2230
2230
1115
0
2021−12−22
2022−01−12
2022−02−02
Indonesia
daily predictions
R_t
3
2
1
0
2021−12−22
2022−01−12
2022−02−02

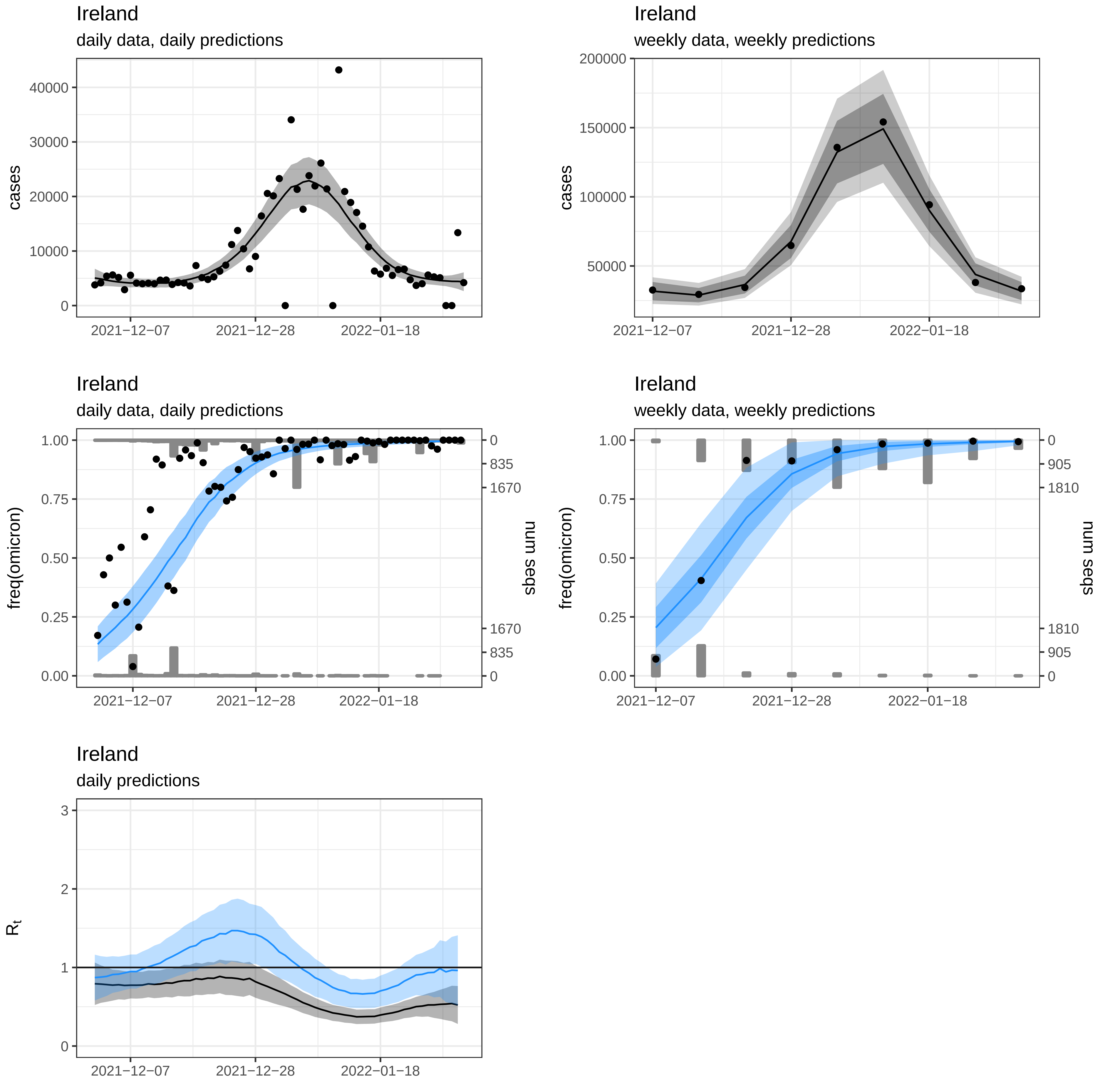
Ireland
daily data, daily predictions
cases
40000
30000
20000
10000
0
2021−12−07
2021−12−28
2022−01−18
Ireland
weekly data, weekly predictions
cases
200000
150000
100000
50000
2021−12−07
2021−12−28
2022−01−18
Ireland
daily data, daily predictions
freq(omicron)
1.00
0.75
0.50
0.25
0.00
num seqs
0
835
1670
1670
835
0
2021−12−07
2021−12−28
2022−01−18
Ireland
weekly data, weekly predictions
freq(omicron)
1.00
0.75
0.50
0.25
0.00
num seqs
0
905
1810
1810
905
0
2021−12−07
2021−12−28
2022−01−18
Ireland
daily predictions
R_t
3
2
1
0
2021−12−07
2021−12−28
2022−01−18

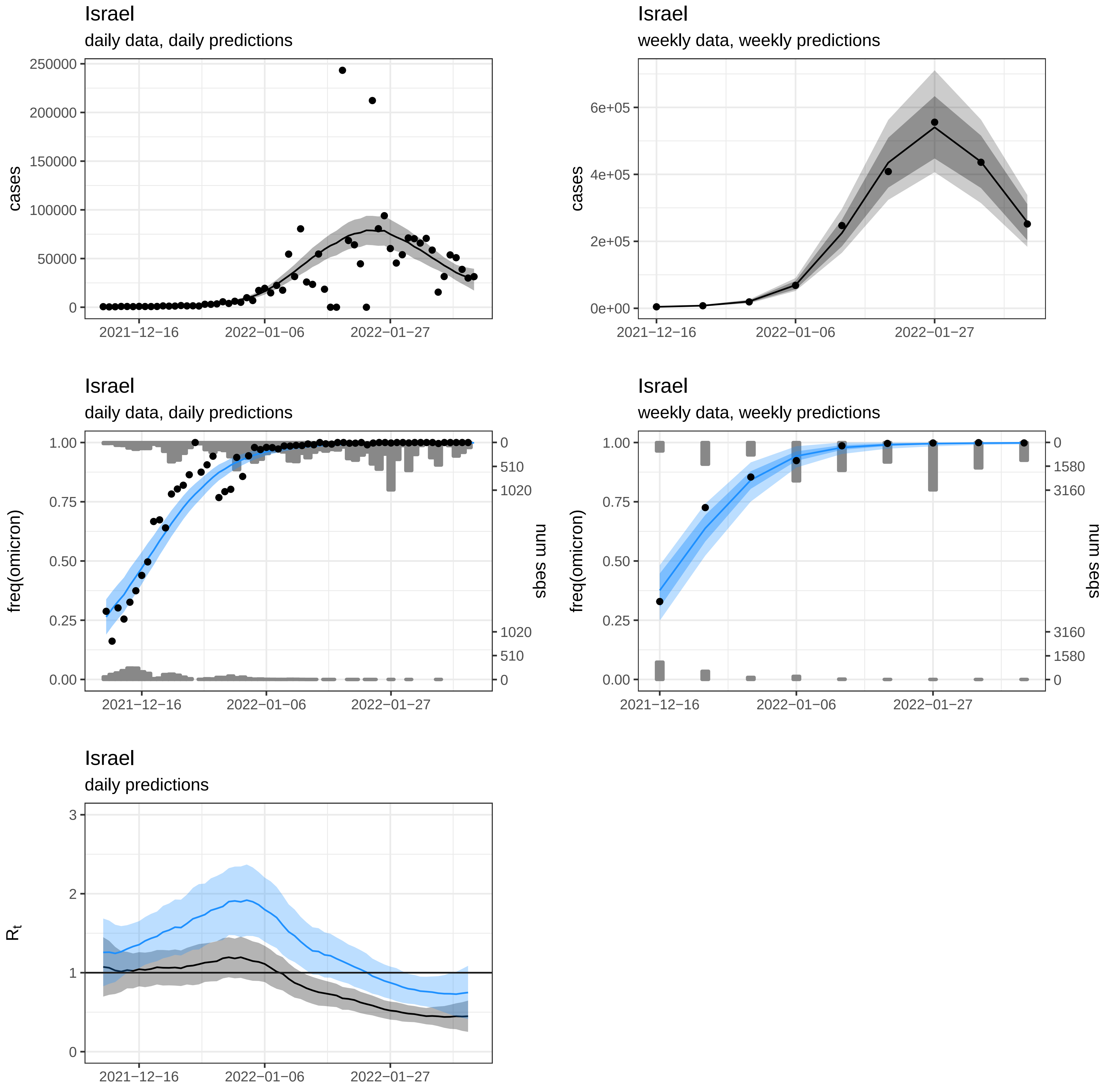
Israel
daily data, daily predictions
cases
250000
200000
150000
100000
50000
0
2021−12−16
2022−01−06
2022−01−27
Israel
weekly data, weekly predictions
cases
6e+05
4e+05
2e+05
0e+00
2021−12−16
2022−01−06
2022−01−27
Israel
daily data, daily predictions
freq(omicron)
1.00
0.75
0.50
0.25
0.00
num seqs
0
510
1020
1020
510
0
2021−12−16
2022−01−06
2022−01−27
Israel
weekly data, weekly predictions
freq(omicron)
1.00
0.75
0.50
0.25
0.00
num seqs
0
1580
3160
3160
1580
0
2021−12−16
2022−01−06
2022−01−27
Israel
daily predictions
R_t
3
2
1
0
2021−12−16
2022−01−06
2022−01−27

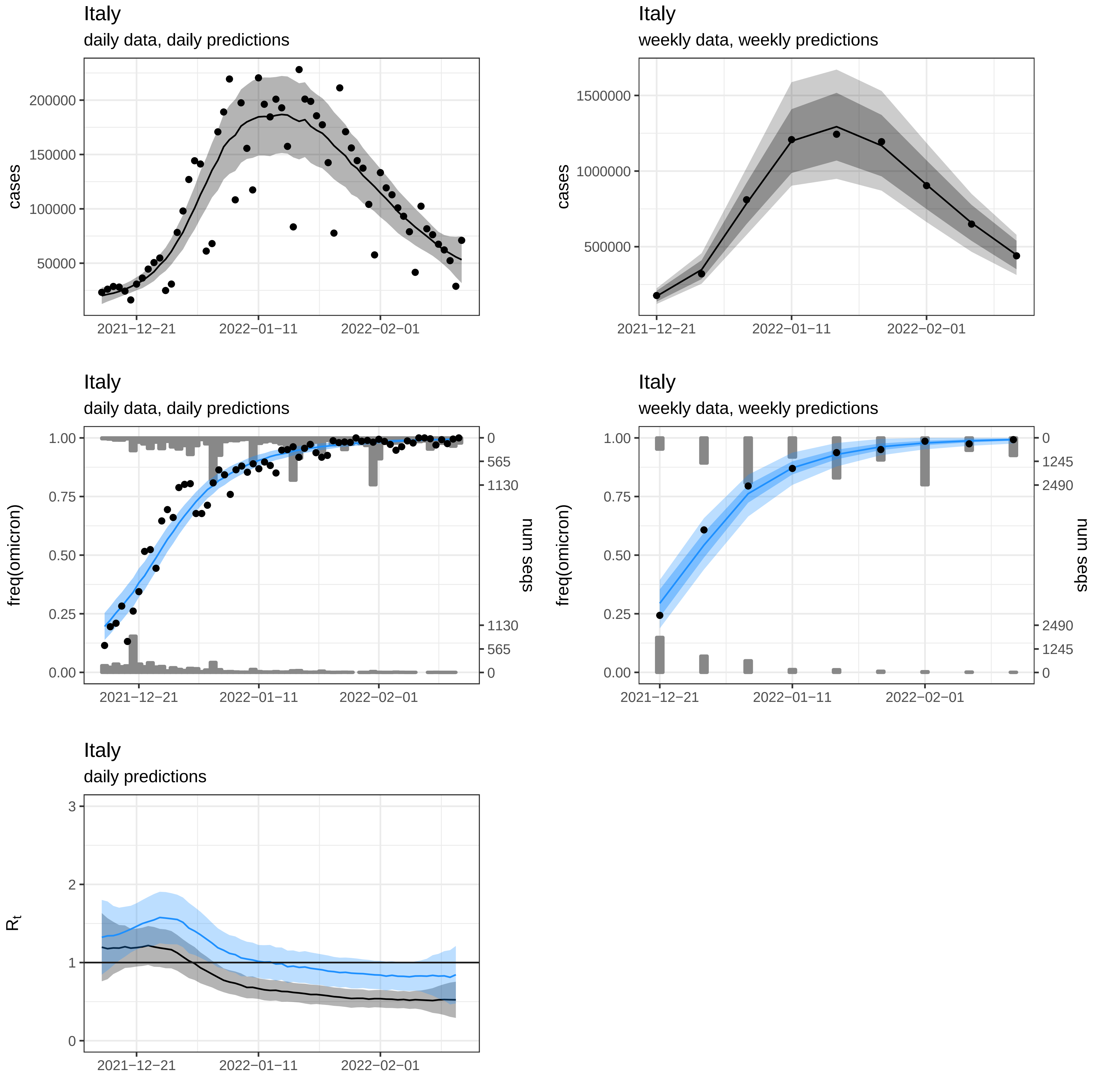
Italy
daily data, daily predictions
cases
200000
150000
100000
50000
2021−12−21
2022−01−11
2022−02−01
Italy
weekly data, weekly predictions
cases
1500000
1000000
500000
2021−12−21
2022−01−11
2022−02−01
Italy
daily data, daily predictions
freq(omicron)
1.00
0.75
0.50
0.25
0.00
num seqs
0
565
1130
1130
565
0
2021−12−21
2022−01−11
2022−02−01
Italy
weekly data, weekly predictions
freq(omicron)
1.00
0.75
0.50
0.25
0.00
num seqs
0
1245
2490
2490
1245
0
2021−12−21
2022−01−11
2022−02−01
Italy
daily predictions
R_t
3
2
1
0
2021−12−21
2022−01−11
2022−02−01

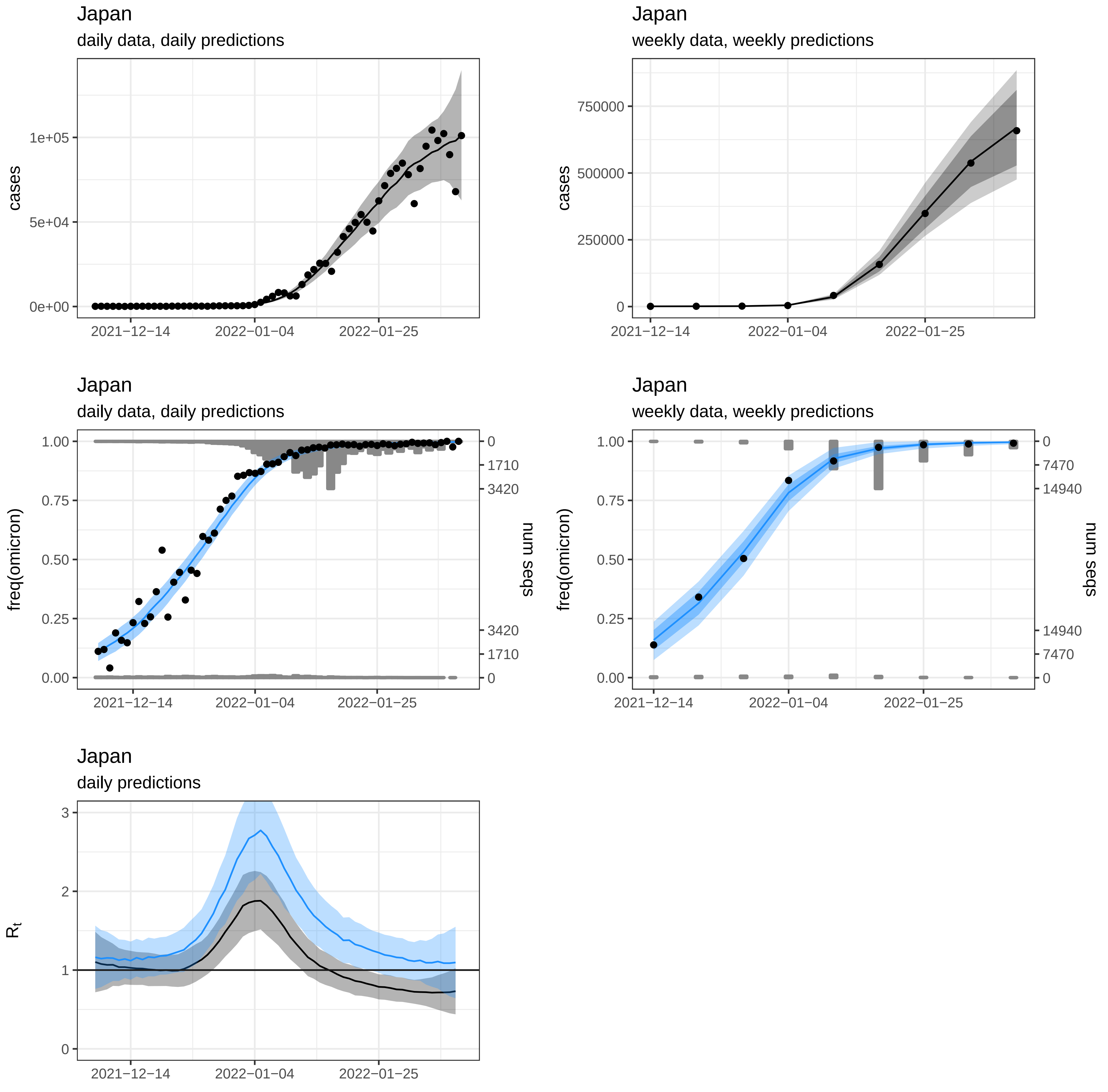
Japan
daily data, daily predictions
cases
1e+05
5e+04
0e+00
2021−12−14
2022−01−04
2022−01−25
Japan
weekly data, weekly predictions
cases
750000
500000
250000
0
2021−12−14
2022−01−04
2022−01−25
Japan
daily data, daily predictions
freq(omicron)
num seqs
1.00
0.75
0.50
0.25
0.00
0
1710
3420
2021−12−14
2022−01−04
2022−01−25
Japan
weekly data, weekly predictions
freq(omicron)
num seqs
1.00
0.75
0.50
0.25
0.00
0
7470
14940
2021−12−14
2022−01−04
2022−01−25
Japan
daily predictions
R_t
3
2
1
0
2021−12−14
2022−01−04
2022−01−25

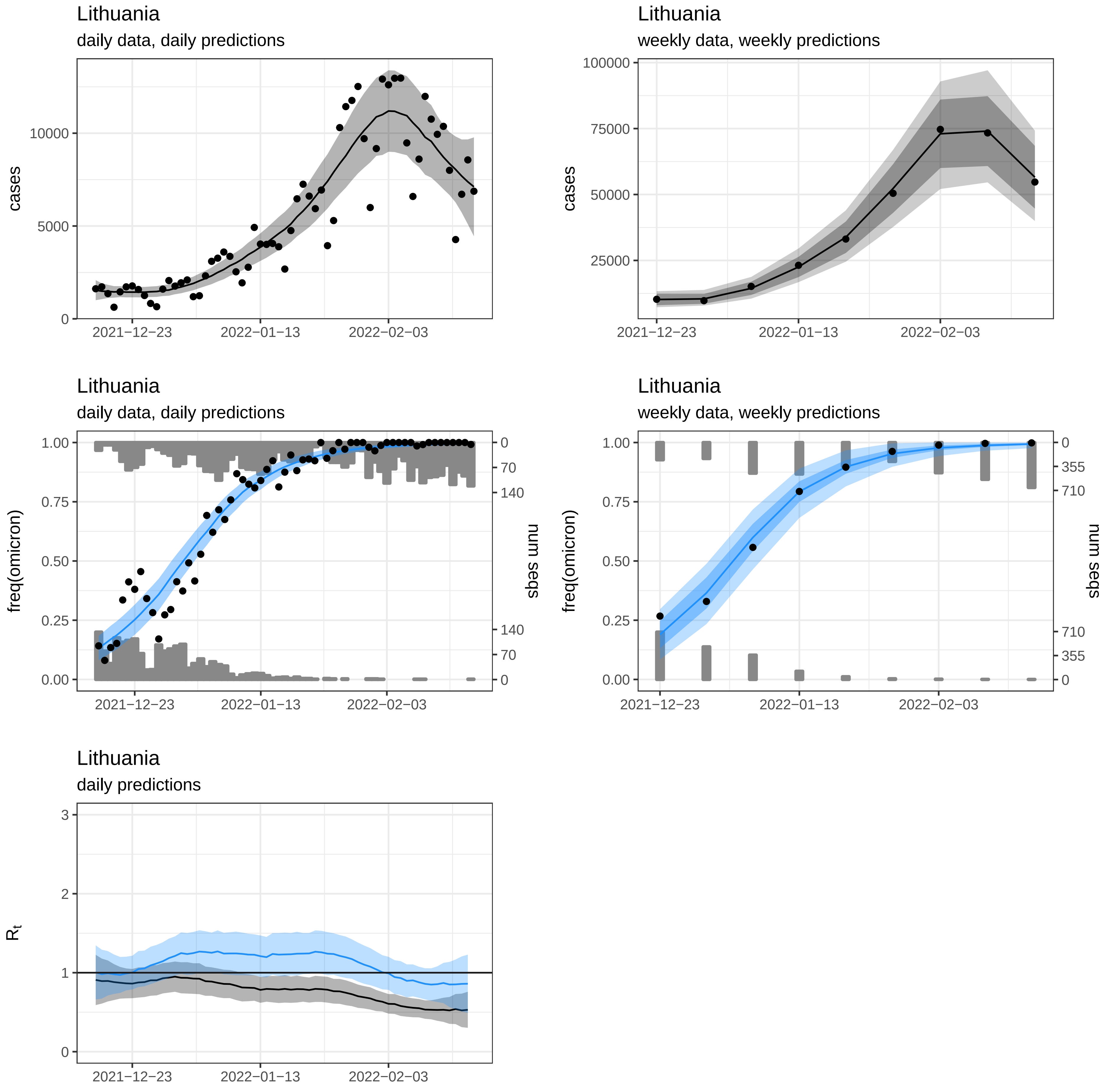
Lithuania
daily data, daily predictions
cases
0
5000
10000
2021−12−23
2022−01−13
2022−02−03
Lithuania
weekly data, weekly predictions
cases
25000
50000
75000
100000
2021−12−23
2022−01−13
2022−02−03
Lithuania
daily data, daily predictions
freq(omicron)
0.00
0.25
0.50
0.75
1.00
num seqs
0
70
140
2021−12−23
2022−01−13
2022−02−03
Lithuania
weekly data, weekly predictions
freq(omicron)
0.00
0.25
0.50
0.75
1.00
num seqs
0
355
710
2021−12−23
2022−01−13
2022−02−03
Lithuania
daily predictions
R_t
0
1
2
3
2021−12−23
2022−01−13
2022−02−03

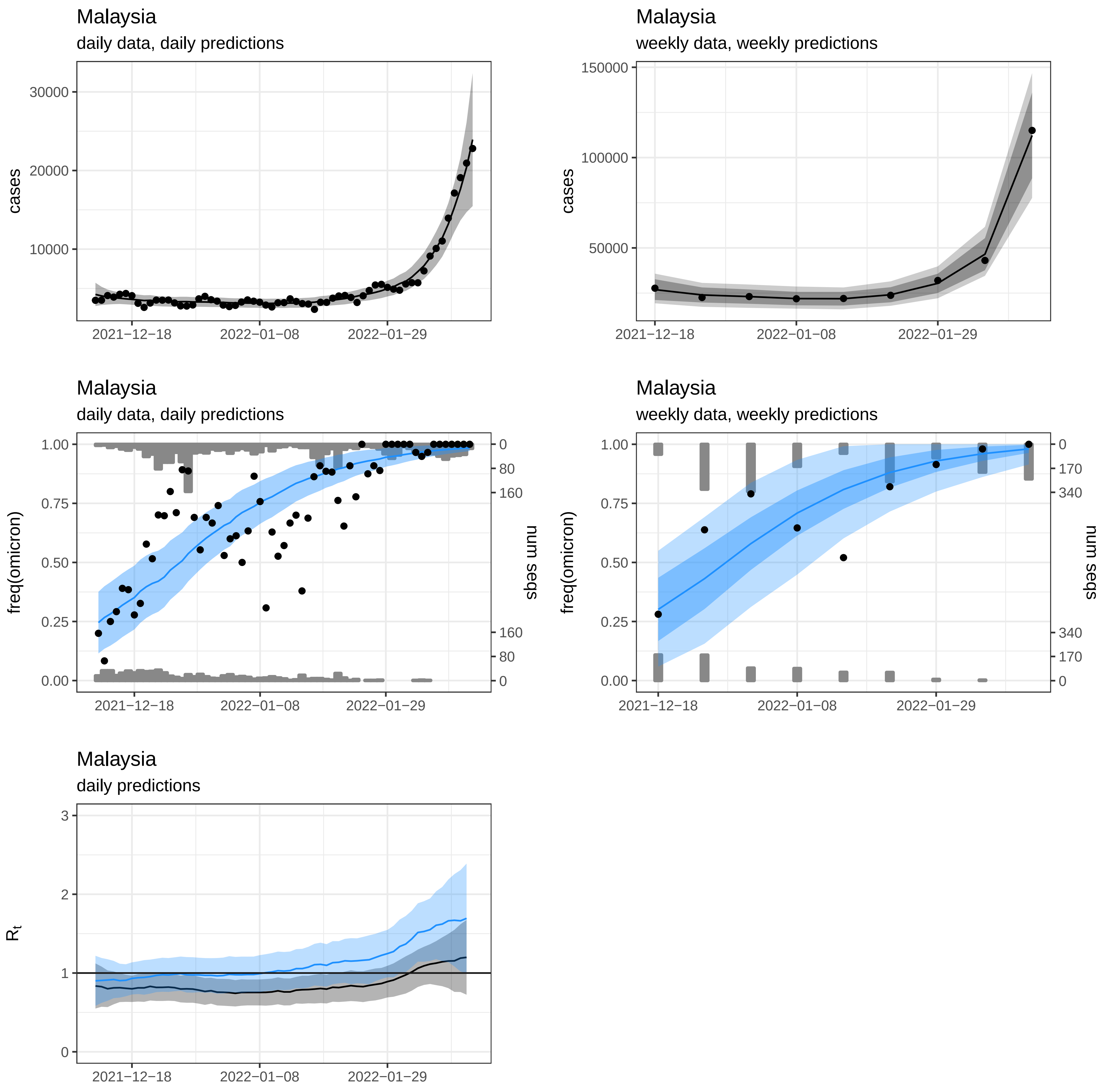

Malaysia
daily data, daily predictions
cases
30000
20000
10000
2021−12−18
2022−01−08
2022−01−29
Malaysia
weekly data, weekly predictions
cases
150000
100000
50000
2021−12−18
2022−01−08
2022−01−29
Malaysia
daily data, daily predictions
freq(omicron)
num seqs
1.00
0.75
0.50
0.25
0.00
0
80
160
2021−12−18
2022−01−08
2022−01−29
Malaysia
weekly data, weekly predictions
freq(omicron)
num seqs
0
170
340
2021−12−18
2022−01−08
2022−01−29
Malaysia
daily predictions
R_t
3
2
1
0
2021−12−18
2022−01−08
2022−01−29

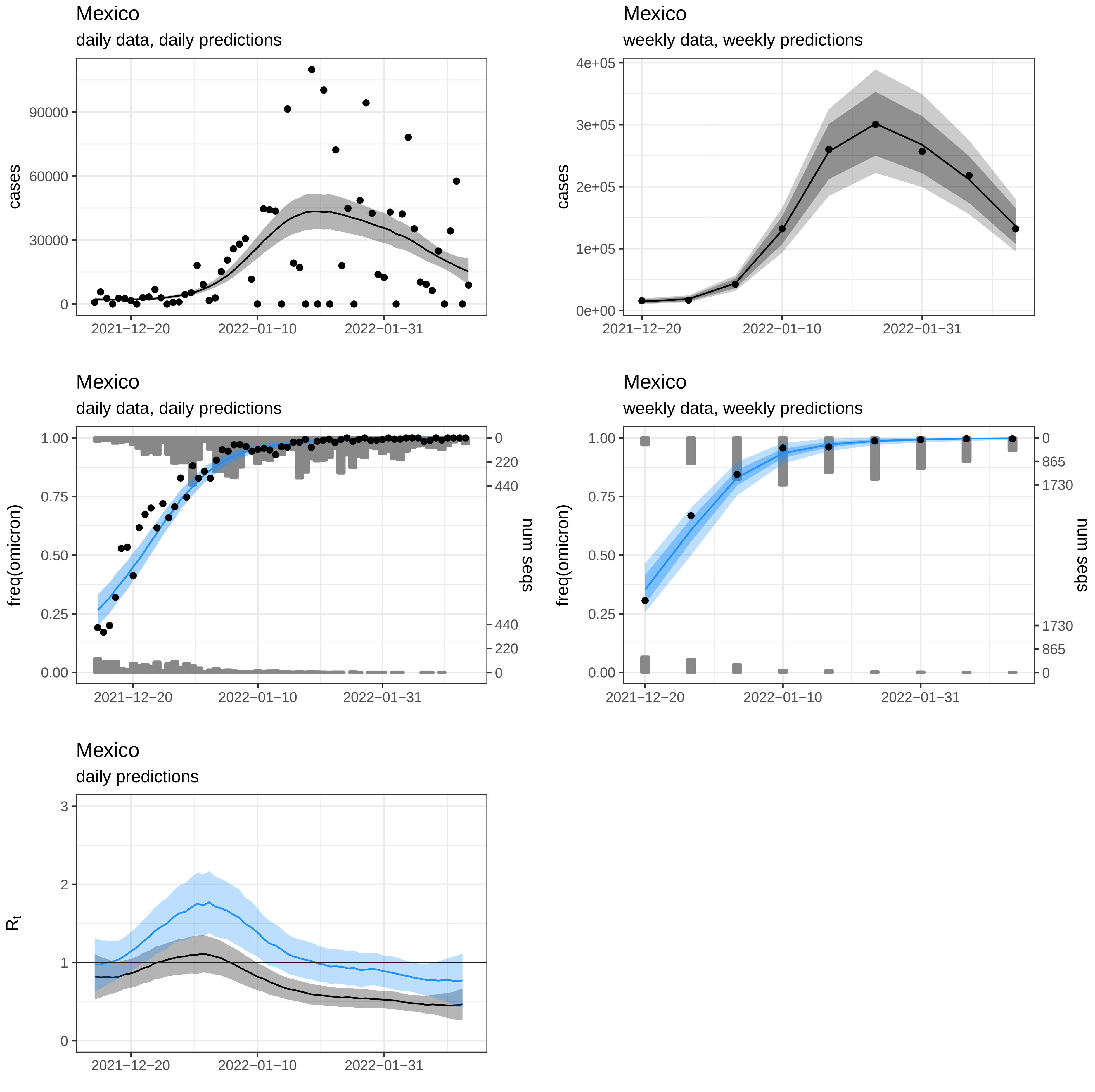
Mexico
daily data, daily predictions
cases
90000
60000
30000
0
2021−12−20
2022−01−10
2022−01−31
Mexico
weekly data, weekly predictions
cases
4e+05
3e+05
2e+05
1e+05
0e+00
2021−12−20
2022−01−10
2022−01−31
Mexico
daily data, daily predictions
freq(omicron)
1.00
0.75
0.50
0.25
0.00
num seqs
0
220
440
440
220
0
2021−12−20
2022−01−10
2022−01−31
Mexico
weekly data, weekly predictions
freq(omicron)
1.00
0.75
0.50
0.25
0.00
num seqs
0
865
1730
1730
865
0
2021−12−20
2022−01−10
2022−01−31
Mexico
daily predictions
R_t
3
2
1
0
2021−12−20
2022−01−10
2022−01−31

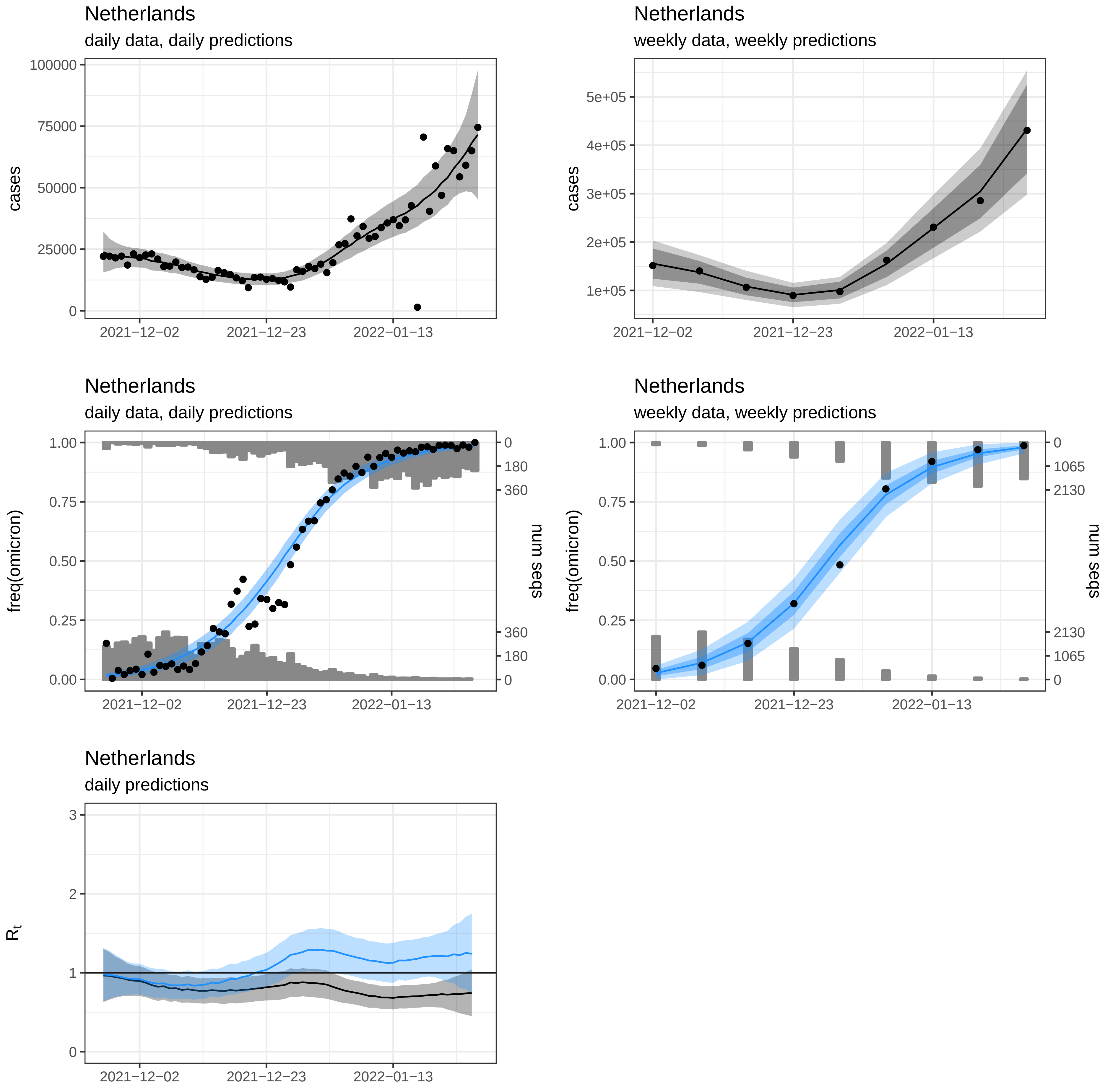
Netherlands
daily data, daily predictions
cases
100000
75000
50000
25000
0
2021−12−02
2021−12−23
2022−01−13
Netherlands
weekly data, weekly predictions
cases
5e+05
4e+05
3e+05
2e+05
1e+05
2021−12−02
2021−12−23
2022−01−13
Netherlands
daily data, daily predictions
freq(omicron)
1.00
0.75
0.50
0.25
0.00
num seqs
0
180
360
360
180
0
2021−12−02
2021−12−23
2022−01−13
Netherlands
weekly data, weekly predictions
freq(omicron)
1.00
0.75
0.50
0.25
0.00
num seqs
0
1065
2130
2130
1065
0
2021−12−02
2021−12−23
2022−01−13
Netherlands
daily predictions
R_t
3
2
1
0
2021−12−02
2021−12−23
2022−01−13

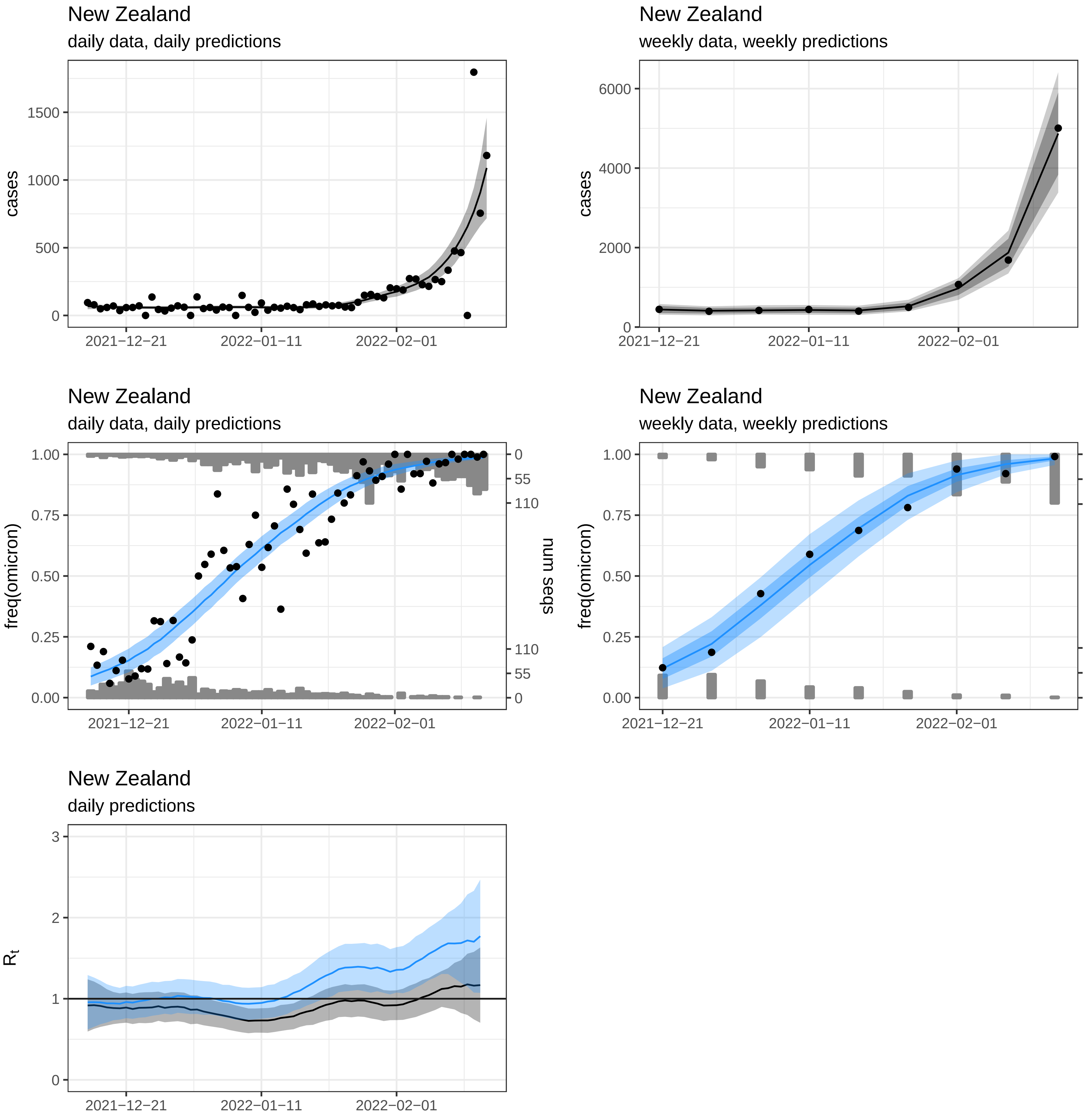
New Zealand
daily data, daily predictions
cases
1500
1000
500
0
2021−12−21
2022−01−11
2022−02−01
New Zealand
weekly data, weekly predictions
cases
6000
4000
2000
0
2021−12−21
2022−01−11
2022−02−01
New Zealand
daily data, daily predictions
freq(omicron)
1.00
0.75
0.50
0.25
0.00
num seqs
0
55
110
110
55
0
2021−12−21
2022−01−11
2022−02−01
New Zealand
weekly data, weekly predictions
freq(omicron)
1.00
0.75
0.50
0.25
0.00
num seqs
0
230
460
460
230
0
2021−12−21
2022−01−11
2022−02−01
New Zealand
daily predictions
R_t
3
2
1
0
2021−12−21
2022−01−11
2022−02−01

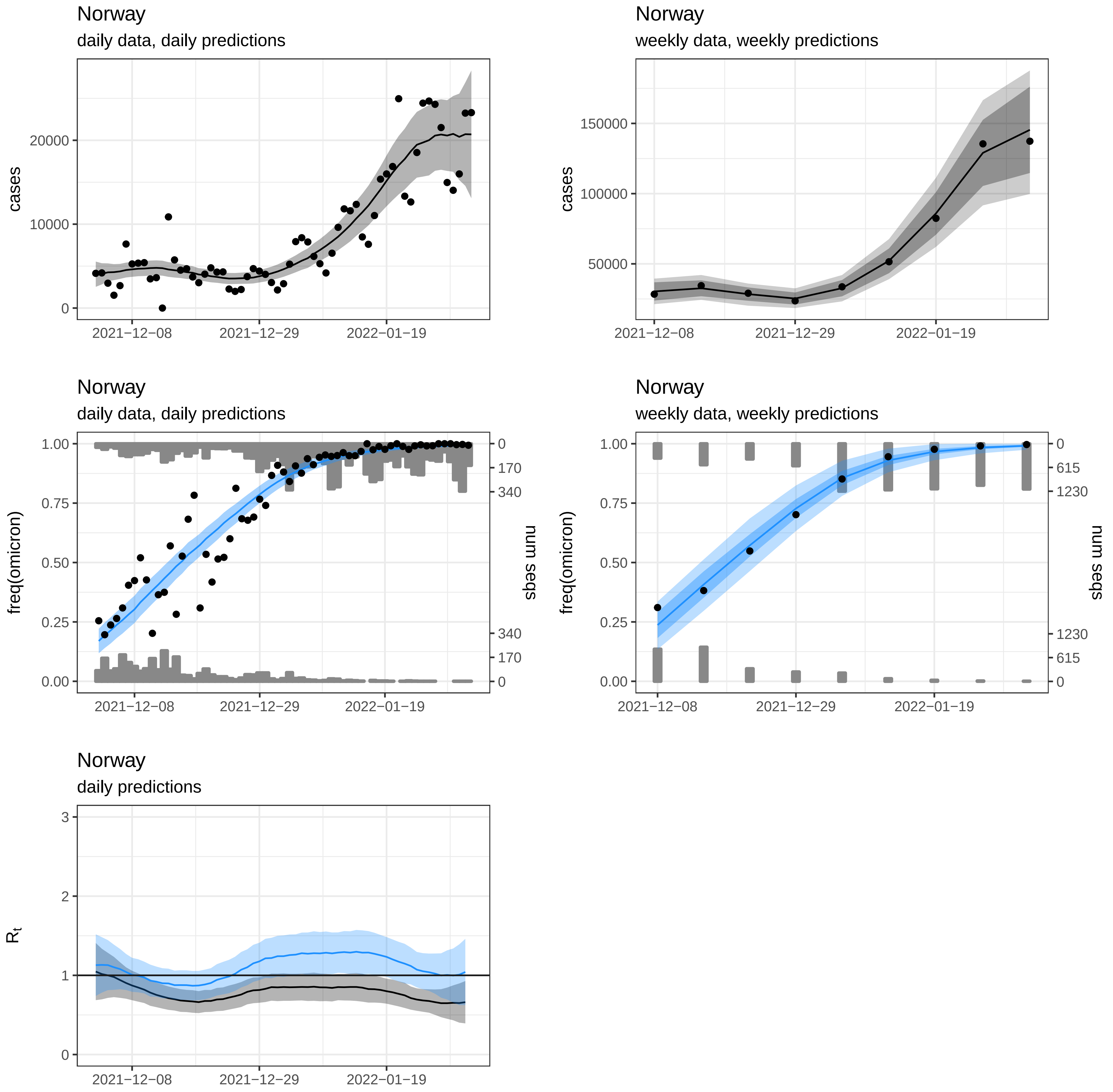
Norway
daily data, daily predictions
cases
20000
10000
0
2021−12−08
2021−12−29
2022−01−19
Norway
weekly data, weekly predictions
cases
150000
100000
50000
2021−12−08
2021−12−29
2022−01−19
Norway
daily data, daily predictions
freq(omicron)
1.00
0.75
0.50
0.25
0.00
num seqs
0
170
340
340
170
0
2021−12−08
2021−12−29
2022−01−19
Norway
weekly data, weekly predictions
freq(omicron)
1.00
0.75
0.50
0.25
0.00
num seqs
0
615
1230
1230
615
0
2021−12−08
2021−12−29
2022−01−19
Norway
daily predictions
R_t
3
2
1
0
2021−12−08
2021−12−29
2022−01−19

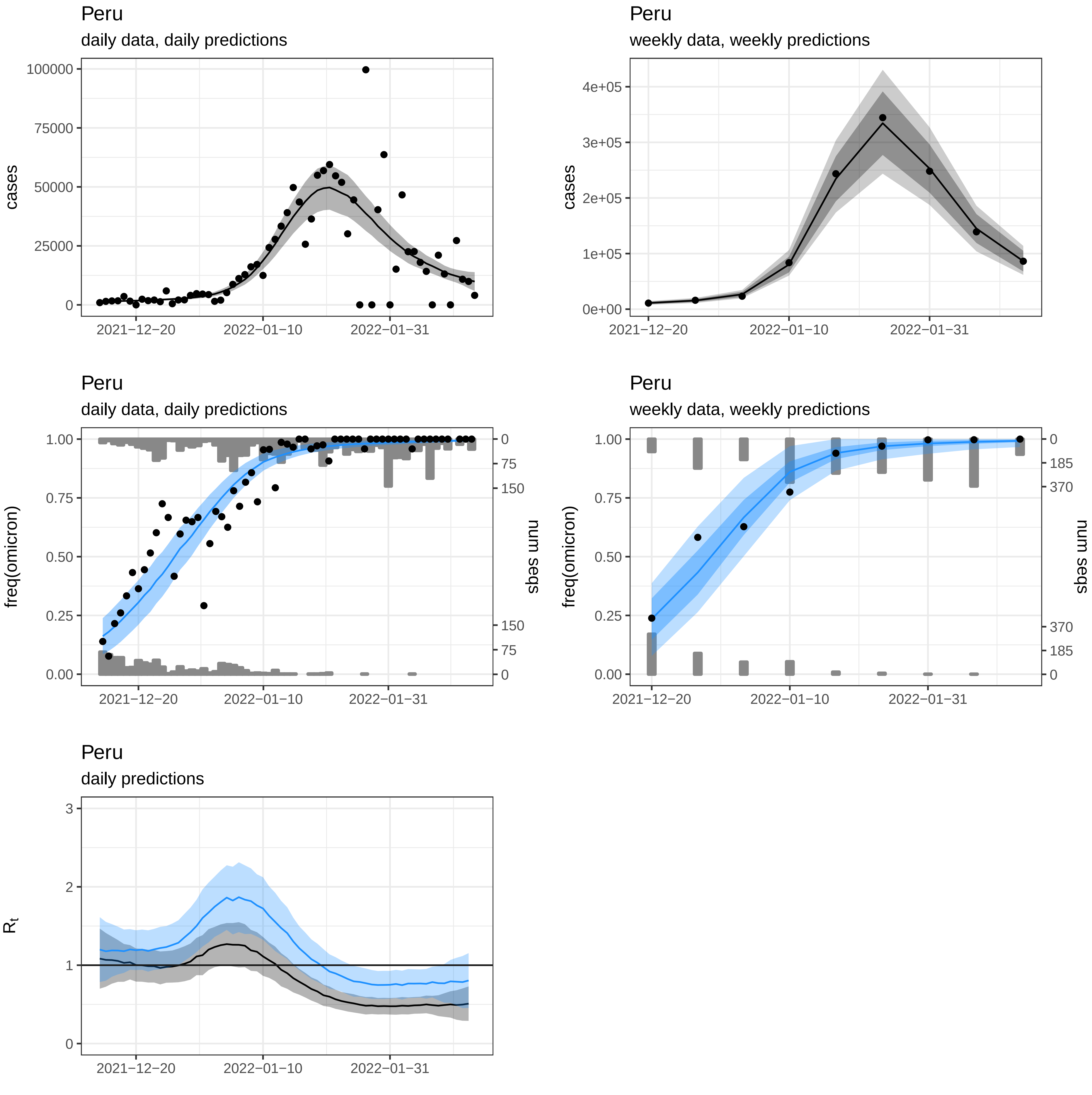

Peru
daily data, daily predictions
cases
100000
75000
50000
25000
0
2021−12−20
2022−01−10
2022−01−31
Peru
weekly data, weekly predictions
cases
4e+05
3e+05
2e+05
1e+05
0e+00
2021−12−20
2022−01−10
2022−01−31
Peru
daily data, daily predictions
freq(omicron)
1.00
0.75
0.50
0.25
0.00
num seqs
0
75
150
150
75
0
2021−12−20
2022−01−10
2022−01−31
Peru
weekly data, weekly predictions
freq(omicron)
1.00
0.75
0.50
0.25
0.00
num seqs
0
185
370
370
185
0
2021−12−20
2022−01−10
2022−01−31
Peru
daily predictions
R_t
3
2
1
0
2021−12−20
2022−01−10
2022−01−31

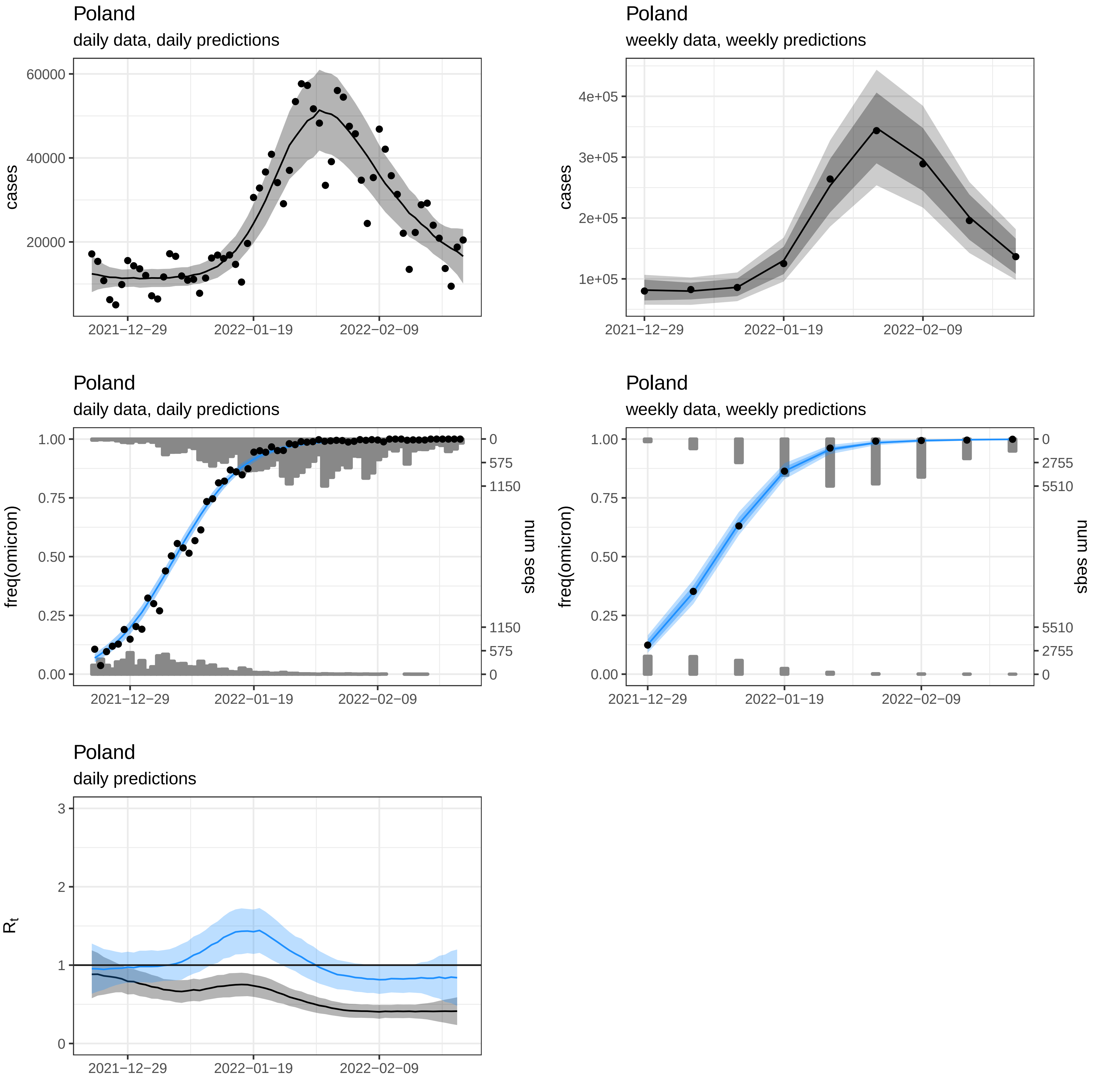
Poland
daily data, daily predictions
cases
60000
40000
20000
2021−12−29
2022−01−19
2022−02−09
Poland
weekly data, weekly predictions
cases
4e+05
3e+05
2e+05
1e+05
2021−12−29
2022−01−19
2022−02−09
Poland
daily data, daily predictions
freq(omicron)
1.00
0.75
0.50
0.25
0.00
num seqs
0
575
1150
1150
575
0
2021−12−29
2022−01−19
2022−02−09
Poland
weekly data, weekly predictions
freq(omicron)
1.00
0.75
0.50
0.25
0.00
num seqs
0
2755
5510
5510
2755
0
2021−12−29
2022−01−19
2022−02−09
Poland
daily predictions
R_t
3
2
1
0
2021−12−29
2022−01−19
2022−02−09

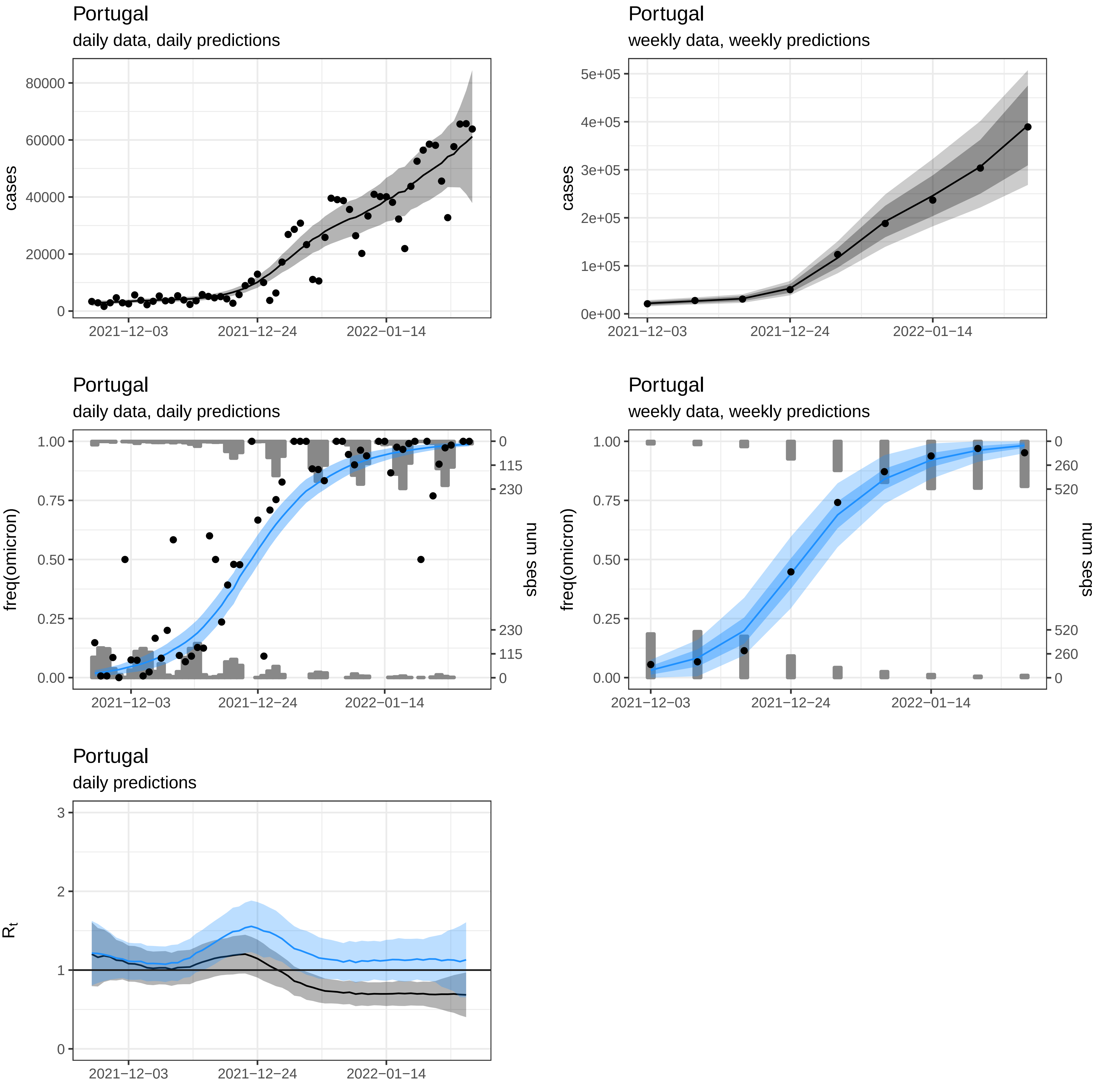
Portugal
daily data, daily predictions
cases
80000
60000
40000
20000
0
2021−12−03
2021−12−24
2022−01−14
Portugal
weekly data, weekly predictions
cases
5e+05
4e+05
3e+05
2e+05
1e+05
0e+00
2021−12−03
2021−12−24
2022−01−14
Portugal
daily data, daily predictions
freq(omicron)
1.00
0.75
0.50
0.25
0.00
num seqs
0
115
230
230
115
0
2021−12−03
2021−12−24
2022−01−14
Portugal
weekly data, weekly predictions
freq(omicron)
1.00
0.75
0.50
0.25
0.00
num seqs
0
260
520
520
260
0
2021−12−03
2021−12−24
2022−01−14
Portugal
daily predictions
R_t
3
2
1
0
2021−12−03
2021−12−24
2022−01−14

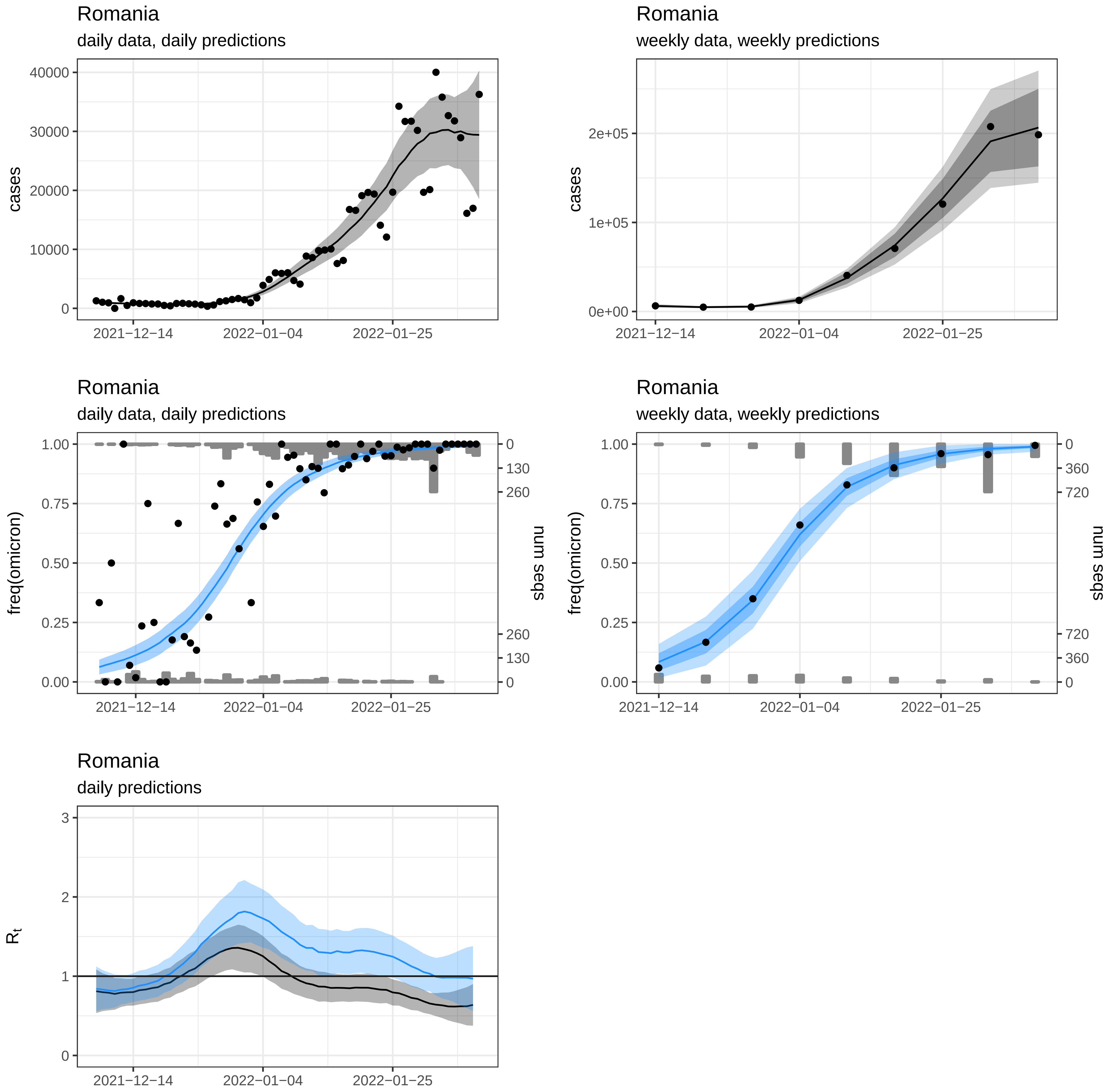
Romania
daily data, daily predictions
cases
40000
30000
20000
10000
0
2021−12−14
2022−01−04
2022−01−25
Romania
weekly data, weekly predictions
cases
2e+05
1e+05
0e+00
2021−12−14
2022−01−04
2022−01−25
Romania
daily data, daily predictions
freq(omicron)
1.00
0.75
0.50
0.25
0.00
num seqs
0
130
260
260
130
0
2021−12−14
2022−01−04
2022−01−25
Romania
weekly data, weekly predictions
freq(omicron)
1.00
0.75
0.50
0.25
0.00
num seqs
0
360
720
720
360
0
2021−12−14
2022−01−04
2022−01−25
Romania
daily predictions
R_t
3
2
1
0
2021−12−14
2022−01−04
2022−01−25

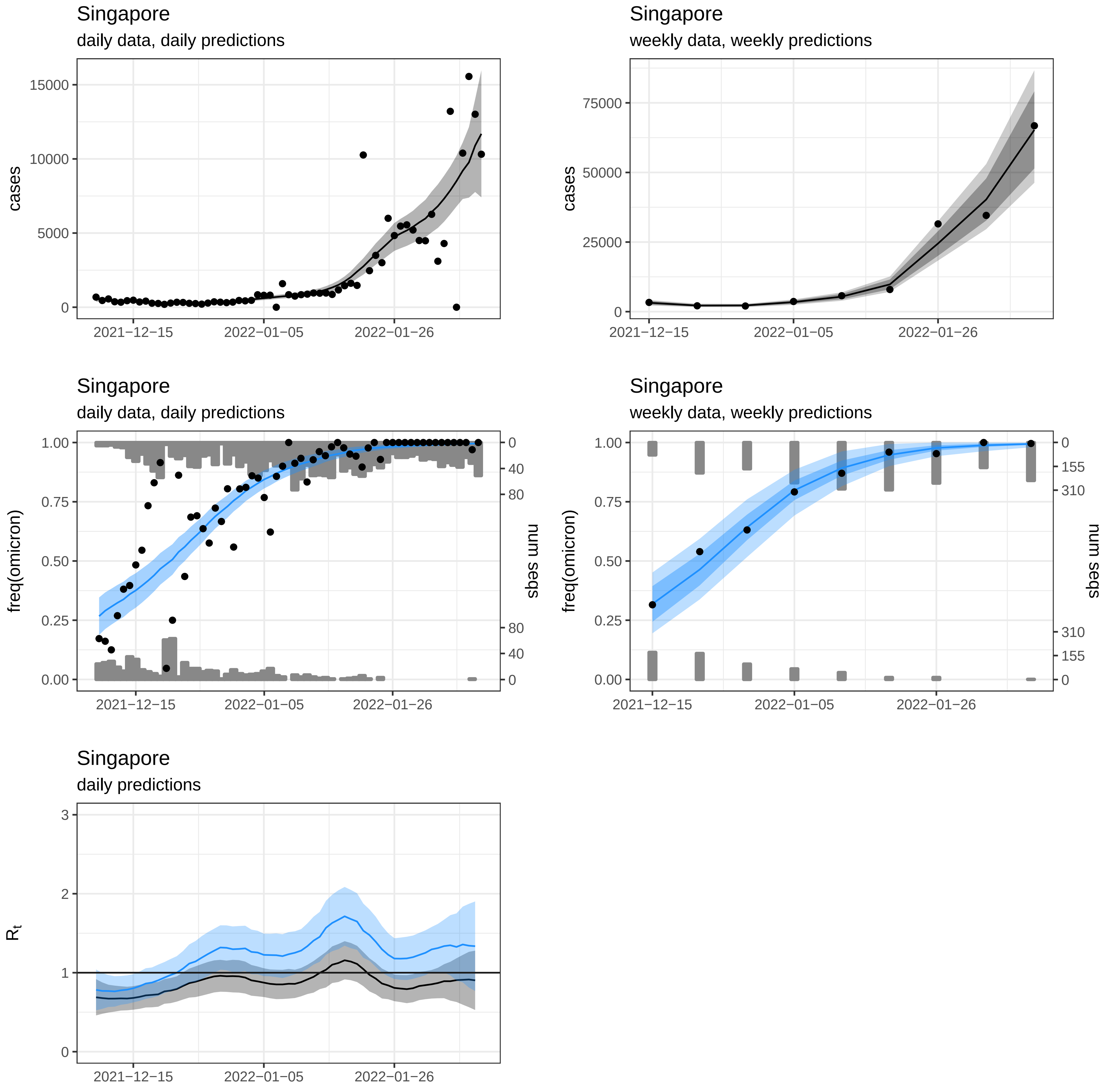
Singapore
daily data, daily predictions
cases
15000
10000
5000
0
2021−12−15
2022−01−05
2022−01−26
Singapore
weekly data, weekly predictions
cases
75000
50000
25000
0
2021−12−15
2022−01−05
2022−01−26
Singapore
daily data, daily predictions
freq(omicron)
1.00
0.75
0.50
0.25
0.00
num seqs
0
40
80
80
40
0
2021−12−15
2022−01−05
2022−01−26
Singapore
weekly data, weekly predictions
freq(omicron)
1.00
0.75
0.50
0.25
0.00
num seqs
0
155
310
310
155
0
2021−12−15
2022−01−05
2022−01−26
Singapore
daily predictions
R_t
3
2
1
0
2021−12−15
2022−01−05
2022−01−26

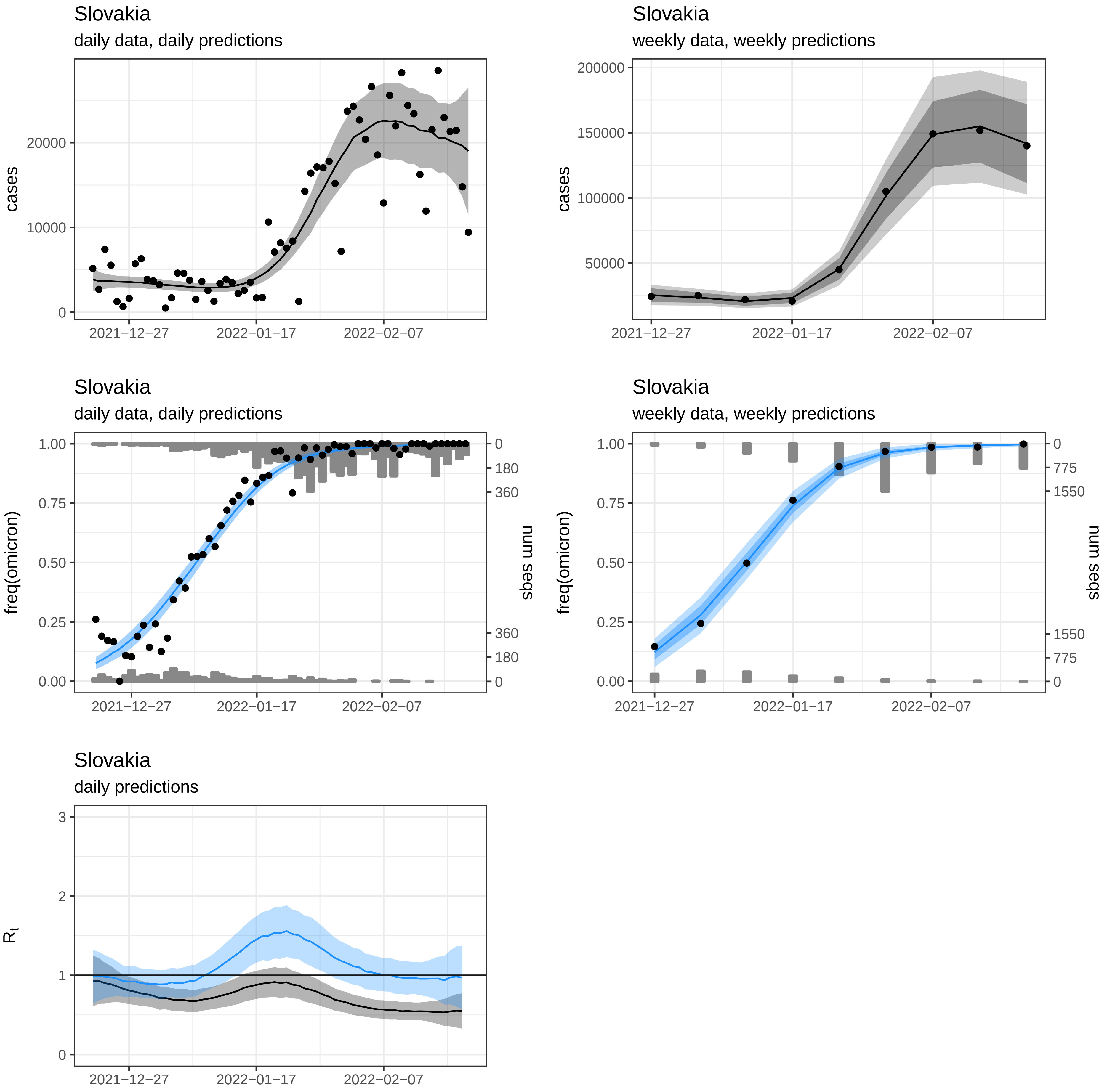
Slovakia
daily data, daily predictions
cases
20000
10000
0
2021−12−27
2022−01−17
2022−02−07
Slovakia
weekly data, weekly predictions
cases
200000
150000
100000
50000
2021−12−27
2022−01−17
2022−02−07
Slovakia
daily data, daily predictions
freq(omicron)
1.00
0.75
0.50
0.25
0.00
num seqs
0
180
360
360
180
0
2021−12−27
2022−01−17
2022−02−07
Slovakia
weekly data, weekly predictions
freq(omicron)
1.00
0.75
0.50
0.25
0.00
num seqs
0
775
1550
1550
775
0
2021−12−27
2022−01−17
2022−02−07
Slovakia
daily predictions
R_t
3
2
1
0
2021−12−27
2022−01−17
2022−02−07

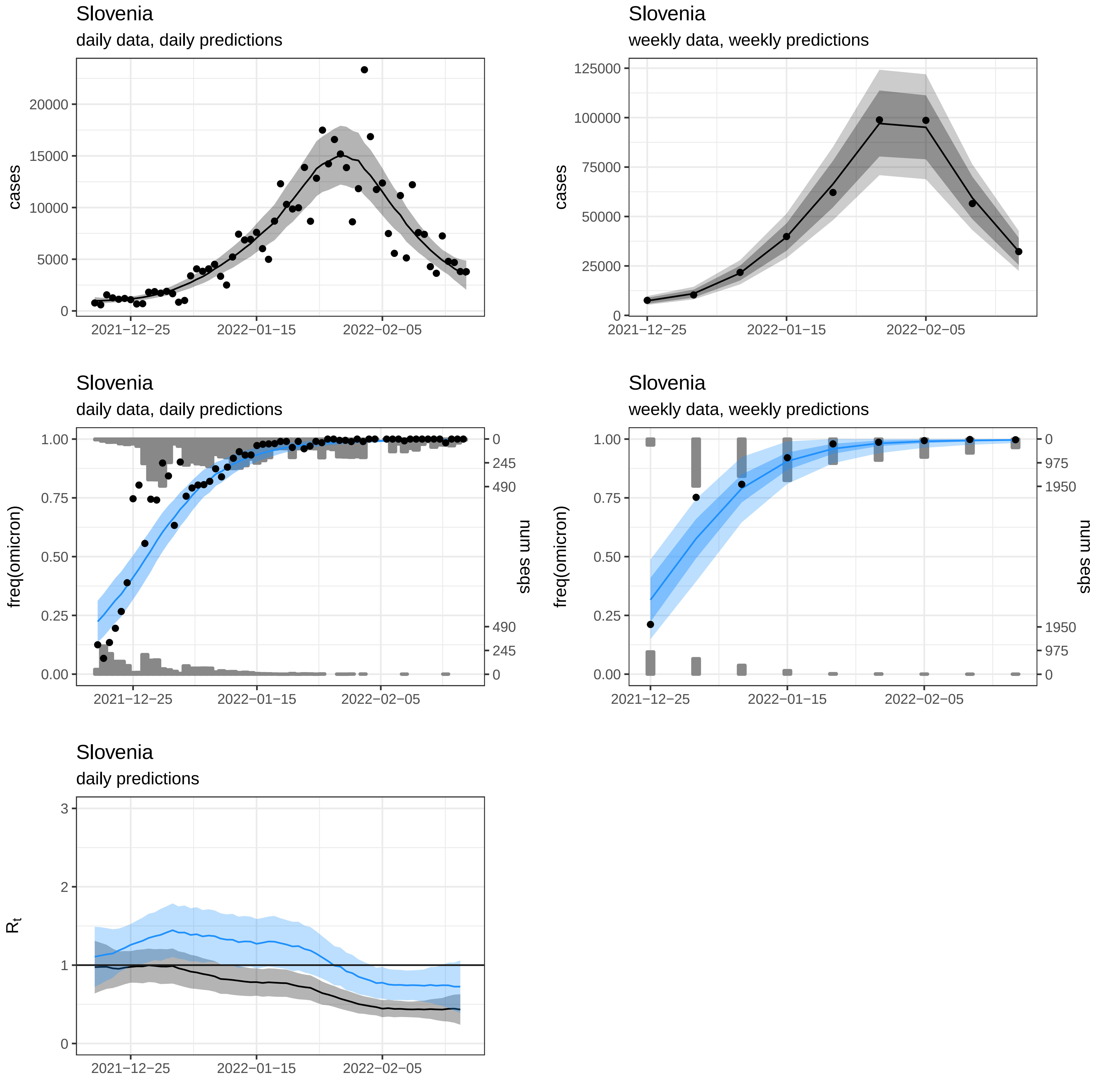
Slovenia
daily data, daily predictions
cases
20000
15000
10000
5000
0
2021−12−25
2022−01−15
2022−02−05
Slovenia
weekly data, weekly predictions
cases
125000
100000
75000
50000
25000
0
2021−12−25
2022−01−15
2022−02−05
Slovenia
daily data, daily predictions
freq(omicron)
1.00
0.75
0.50
0.25
0.00
num seqs
0
245
490
490
245
0
2021−12−25
2022−01−15
2022−02−05
Slovenia
weekly data, weekly predictions
freq(omicron)
1.00
0.75
0.50
0.25
0.00
num seqs
0
975
1950
1950
975
0
2021−12−25
2022−01−15
2022−02−05
Slovenia
daily predictions
R_t
3
2
1
0
2021−12−25
2022−01−15
2022−02−05

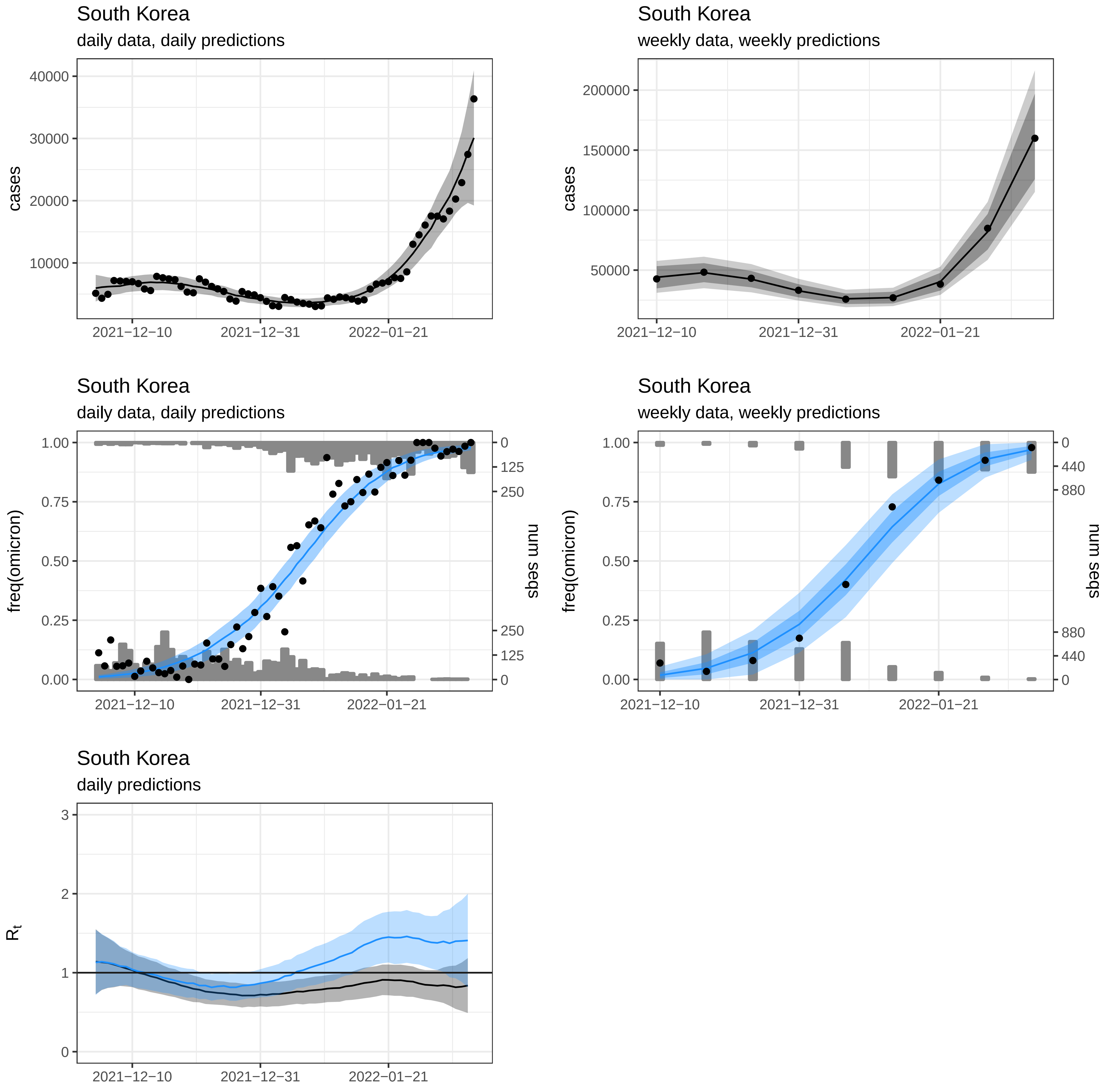
South Korea
daily data, daily predictions
cases
40000
30000
20000
10000
2021−12−10
2021−12−31
2022−01−21
South Korea
weekly data, weekly predictions
cases
200000
150000
100000
50000
2021−12−10
2021−12−31
2022−01−21
South Korea
daily data, daily predictions
freq(omicron)
1.00
0.75
0.50
0.25
0.00
num seqs
0
125
250
250
125
0
2021−12−10
2021−12−31
2022−01−21
South Korea
weekly data, weekly predictions
freq(omicron)
1.00
0.75
0.50
0.25
0.00
num seqs
0
440
880
880
440
0
2021−12−10
2021−12−31
2022−01−21
South Korea
daily predictions
R_t
3
2
1
0
2021−12−10
2021−12−31
2022−01−21

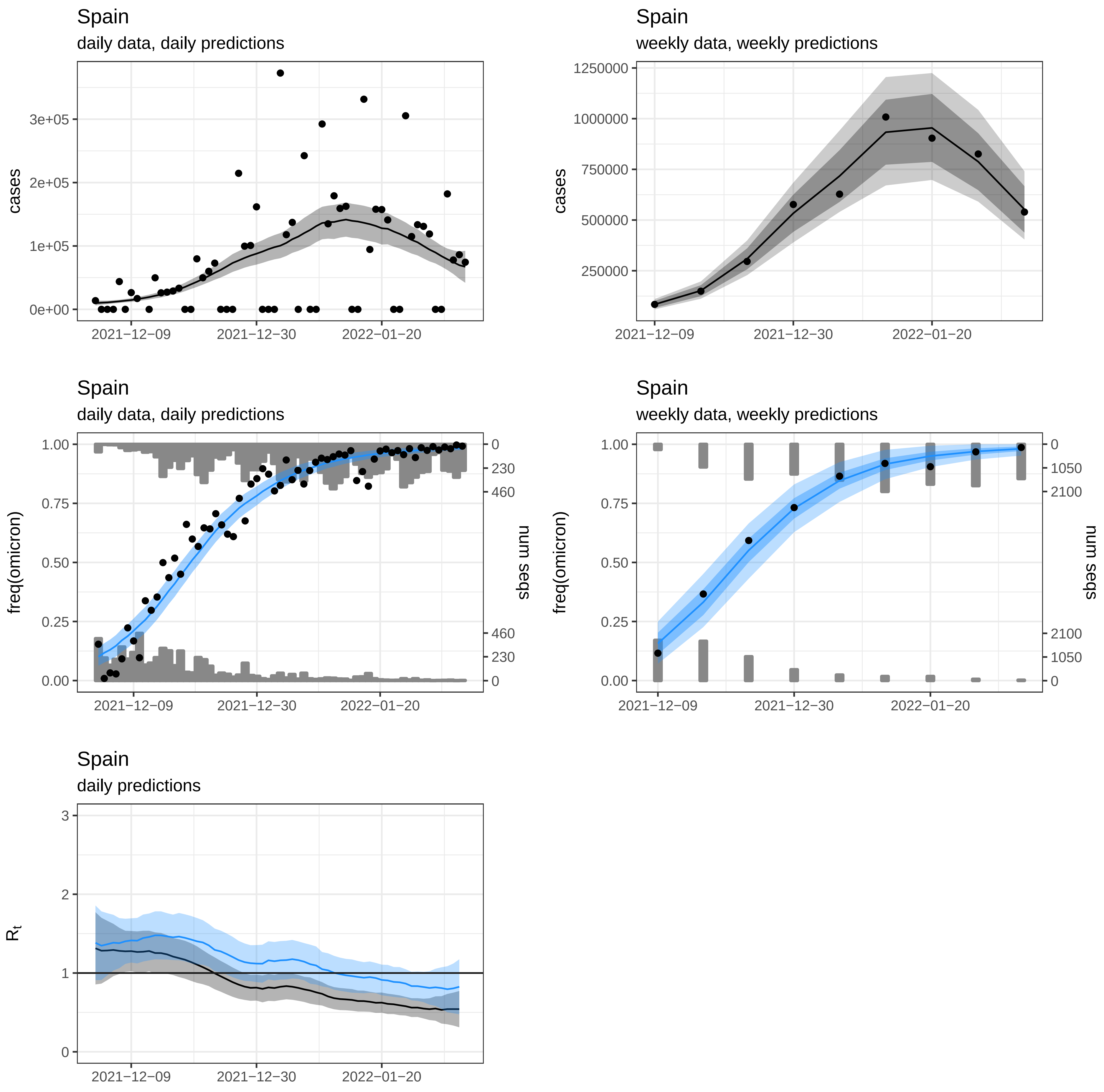
Spain
daily data, daily predictions
cases
3e+05
2e+05
1e+05
0e+00
2021−12−09
2021−12−30
2022−01−20
Spain
weekly data, weekly predictions
cases
1250000
1000000
750000
500000
250000
2021−12−09
2021−12−30
2022−01−20
Spain
daily data, daily predictions
freq(omicron)
1.00
0.75
0.50
0.25
0.00
num seqs
0
230
460
2021−12−09
2021−12−30
2022−01−20
Spain
weekly data, weekly predictions
freq(omicron)
1.00
0.75
0.50
0.25
0.00
num seqs
0
1050
2100
2021−12−09
2021−12−30
2022−01−20
Spain
daily predictions
R_t
3
2
1
0
2021−12−09
2021−12−30
2022−01−20

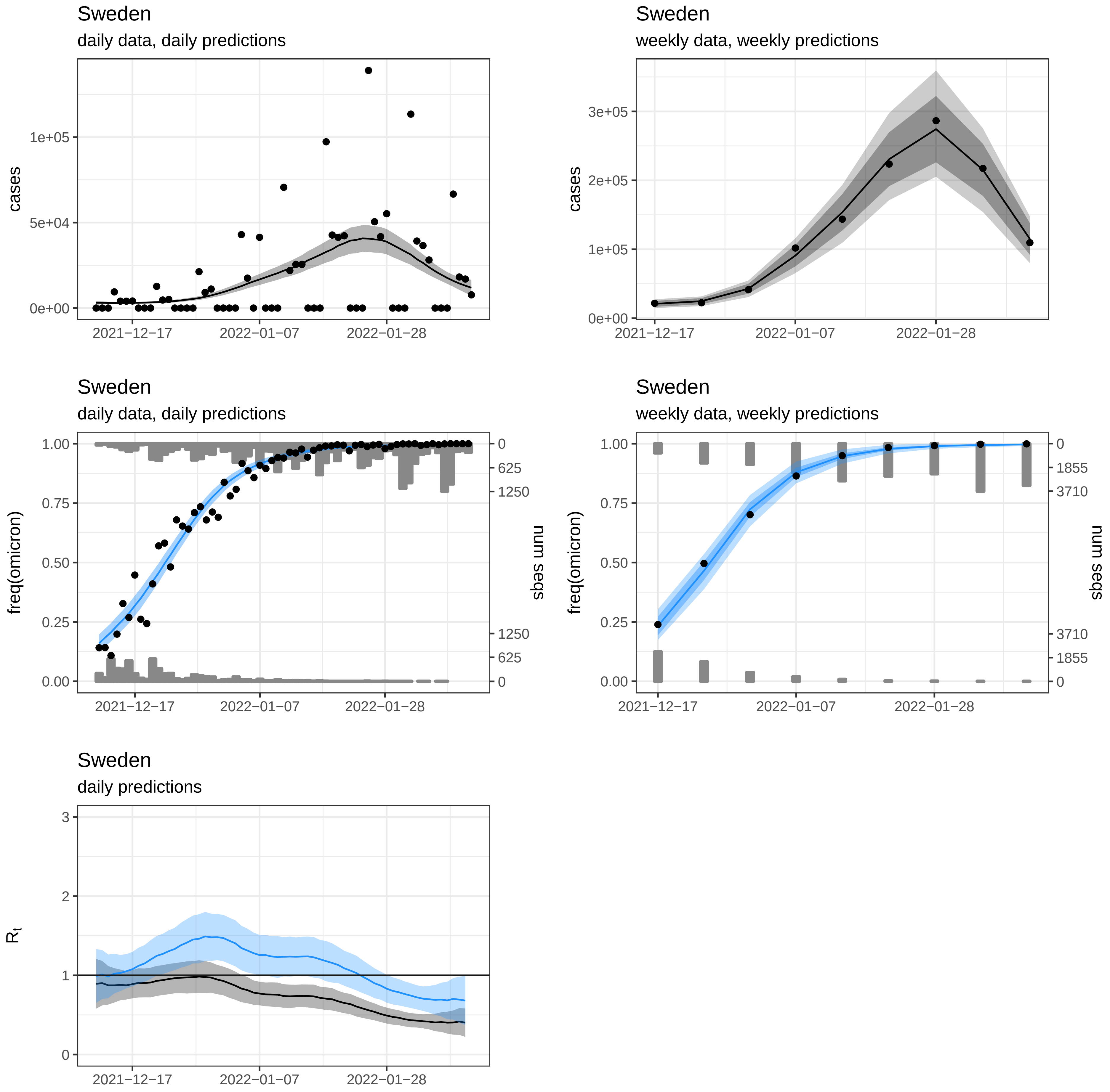
Sweden
daily data, daily predictions
cases
1e+05
5e+04
0e+00
2021−12−17
2022−01−07
2022−01−28
Sweden
weekly data, weekly predictions
cases
3e+05
2e+05
1e+05
0e+00
2021−12−17
2022−01−07
2022−01−28
Sweden
daily data, daily predictions
freq(omicron)
1.00
0.75
0.50
0.25
0.00
num seqs
0
625
1250
1250
625
0
2021−12−17
2022−01−07
2022−01−28
Sweden
weekly data, weekly predictions
freq(omicron)
1.00
0.75
0.50
0.25
0.00
num seqs
0
1855
3710
3710
1855
0
2021−12−17
2022−01−07
2022−01−28
Sweden
daily predictions
R_t
3
2
1
0
2021−12−17
2022−01−07
2022−01−28

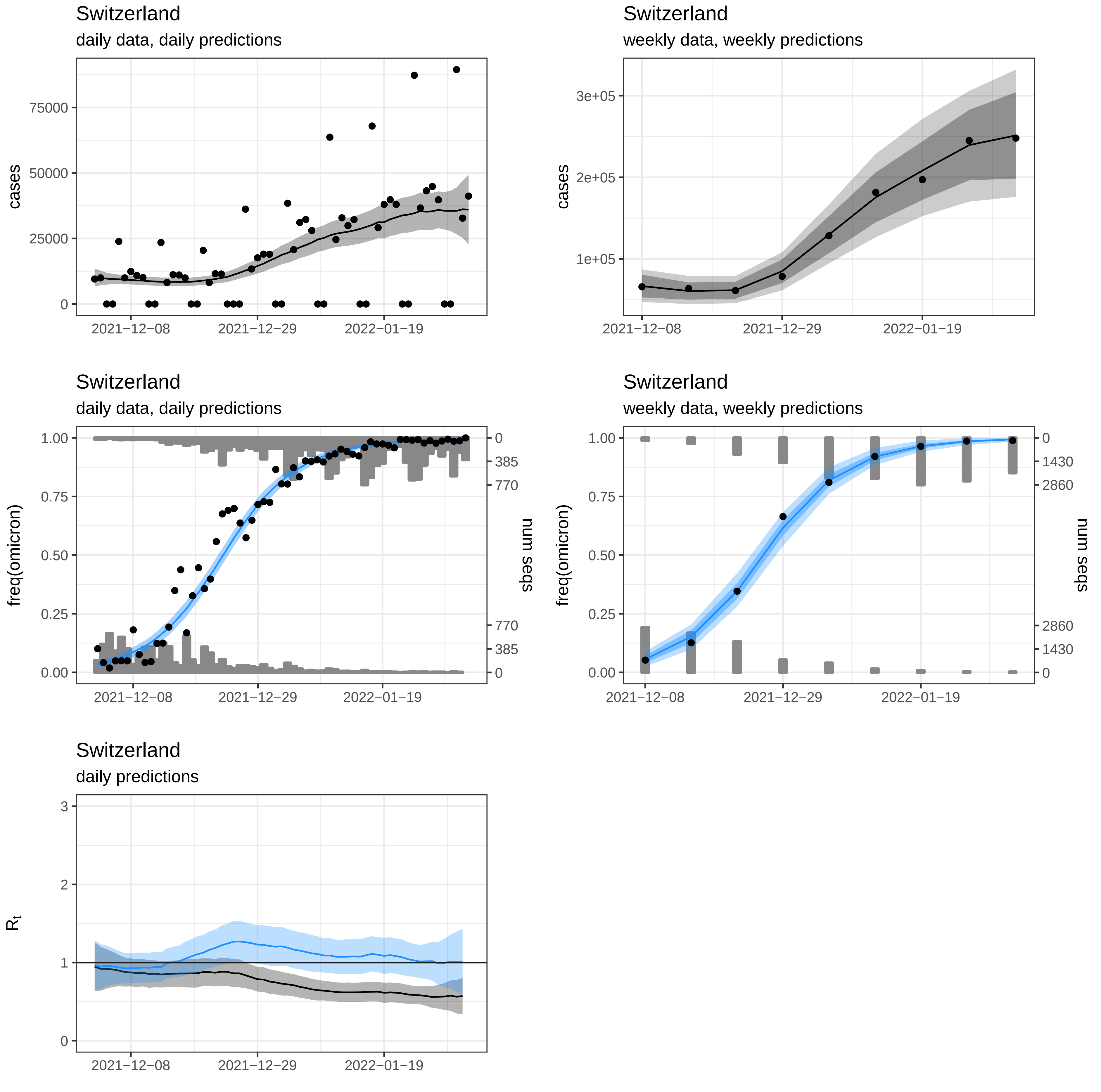

Switzerland
daily data, daily predictions
cases
0
25000
50000
75000
2021−12−08
2021−12−29
2022−01−19
Switzerland
weekly data, weekly predictions
cases
1e+05
2e+05
3e+05
2021−12−08
2021−12−29
2022−01−19
Switzerland
daily data, daily predictions
freq(omicron)
0.00
0.25
0.50
0.75
1.00
num seqs
0
385
770
2021−12−08
2021−12−29
2022−01−19
Switzerland
weekly data, weekly predictions
freq(omicron)
0.00
0.25
0.50
0.75
1.00
num seqs
0
1430
2860
2021−12−08
2021−12−29
2022−01−19
Switzerland
daily predictions
R_t
0
1
2
3
2021−12−08
2021−12−29
2022−01−19

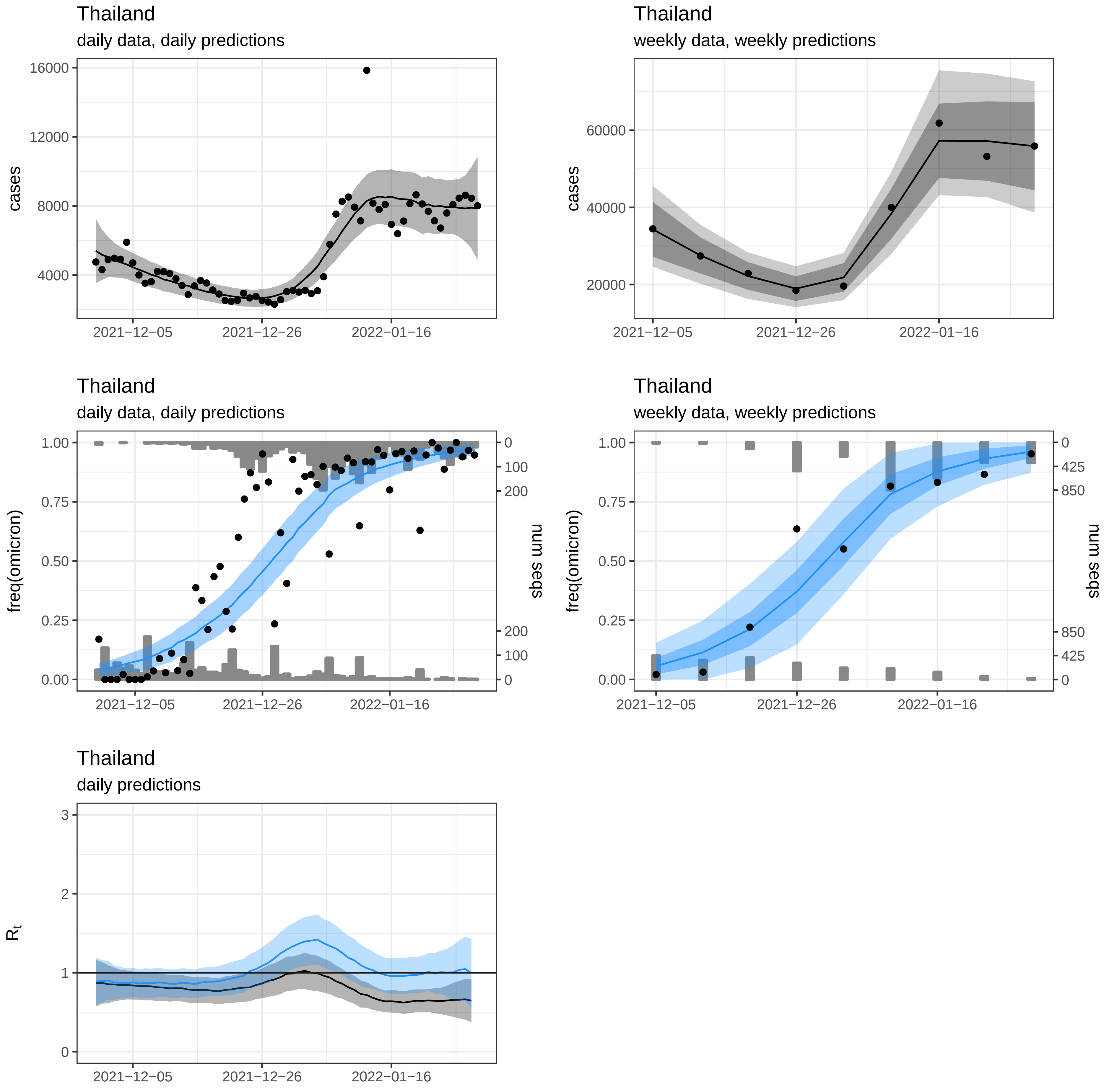
Thailand
daily data, daily predictions
cases
16000
12000
8000
4000
2021−12−05
2021−12−26
2022−01−16
Thailand
weekly data, weekly predictions
cases
60000
40000
20000
2021−12−05
2021−12−26
2022−01−16
Thailand
daily data, daily predictions
freq(omicron)
1.00
0.75
0.50
0.25
0.00
num seqs
0
100
200
200
100
0
2021−12−05
2021−12−26
2022−01−16
Thailand
weekly data, weekly predictions
freq(omicron)
1.00
0.75
0.50
0.25
0.00
num seqs
0
425
850
850
425
0
2021−12−05
2021−12−26
2022−01−16
Thailand
daily predictions
R_t
3
2
1
0
2021−12−05
2021−12−26
2022−01−16

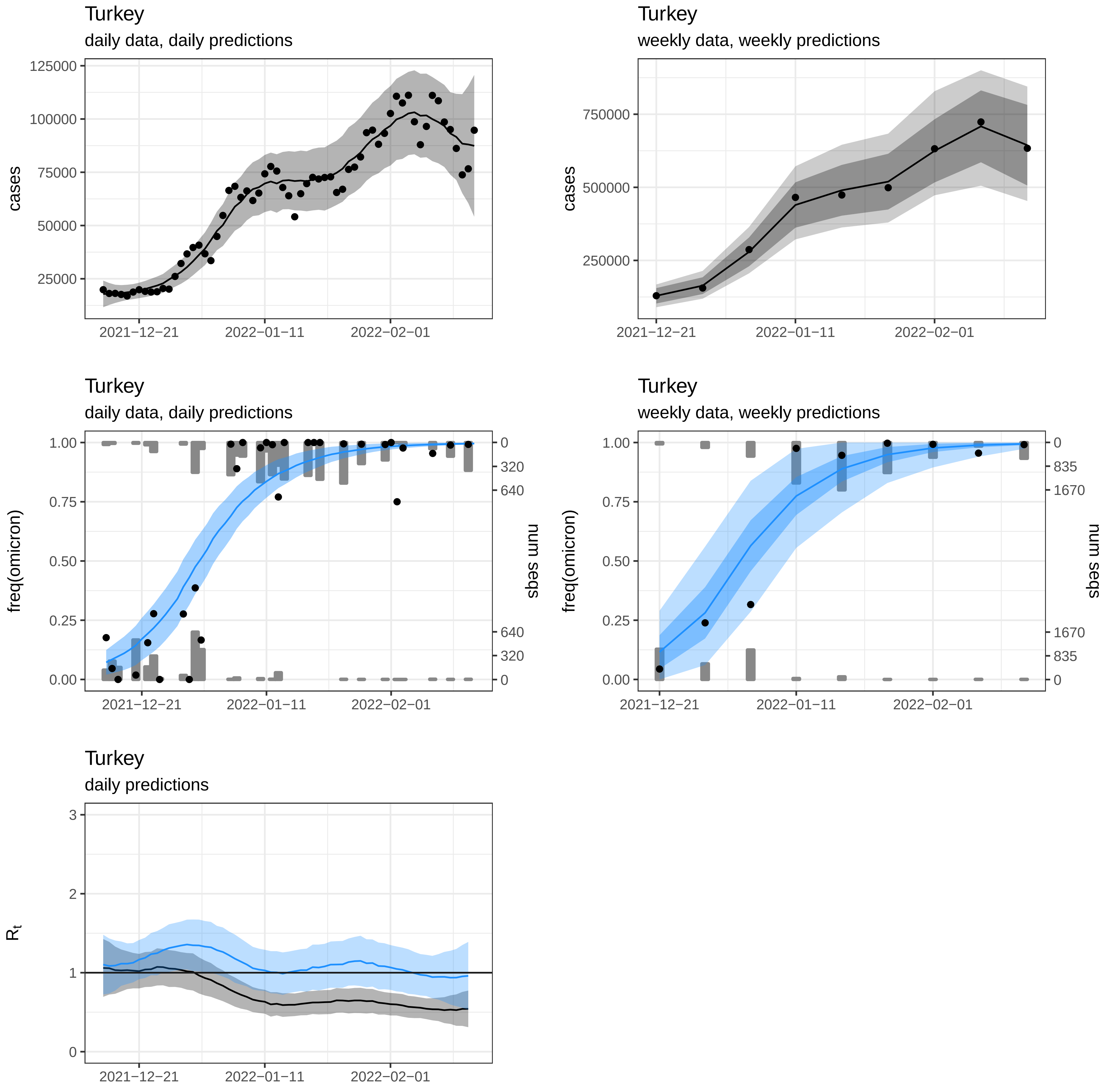
Turkey
daily data, daily predictions
cases
125000
100000
75000
50000
25000
2021−12−21
2022−01−11
2022−02−01
Turkey
weekly data, weekly predictions
cases
750000
500000
250000
2021−12−21
2022−01−11
2022−02−01
Turkey
daily data, daily predictions
freq(omicron)
1.00
0.75
0.50
0.25
0.00
num seqs
0
320
640
640
320
0
2021−12−21
2022−01−11
2022−02−01
Turkey
weekly data, weekly predictions
freq(omicron)
1.00
0.75
0.50
0.25
0.00
num seqs
0
835
1670
1670
835
0
2021−12−21
2022−01−11
2022−02−01
Turkey
daily predictions
R_t
3
2
1
0
2021−12−21
2022−01−11
2022−02−01

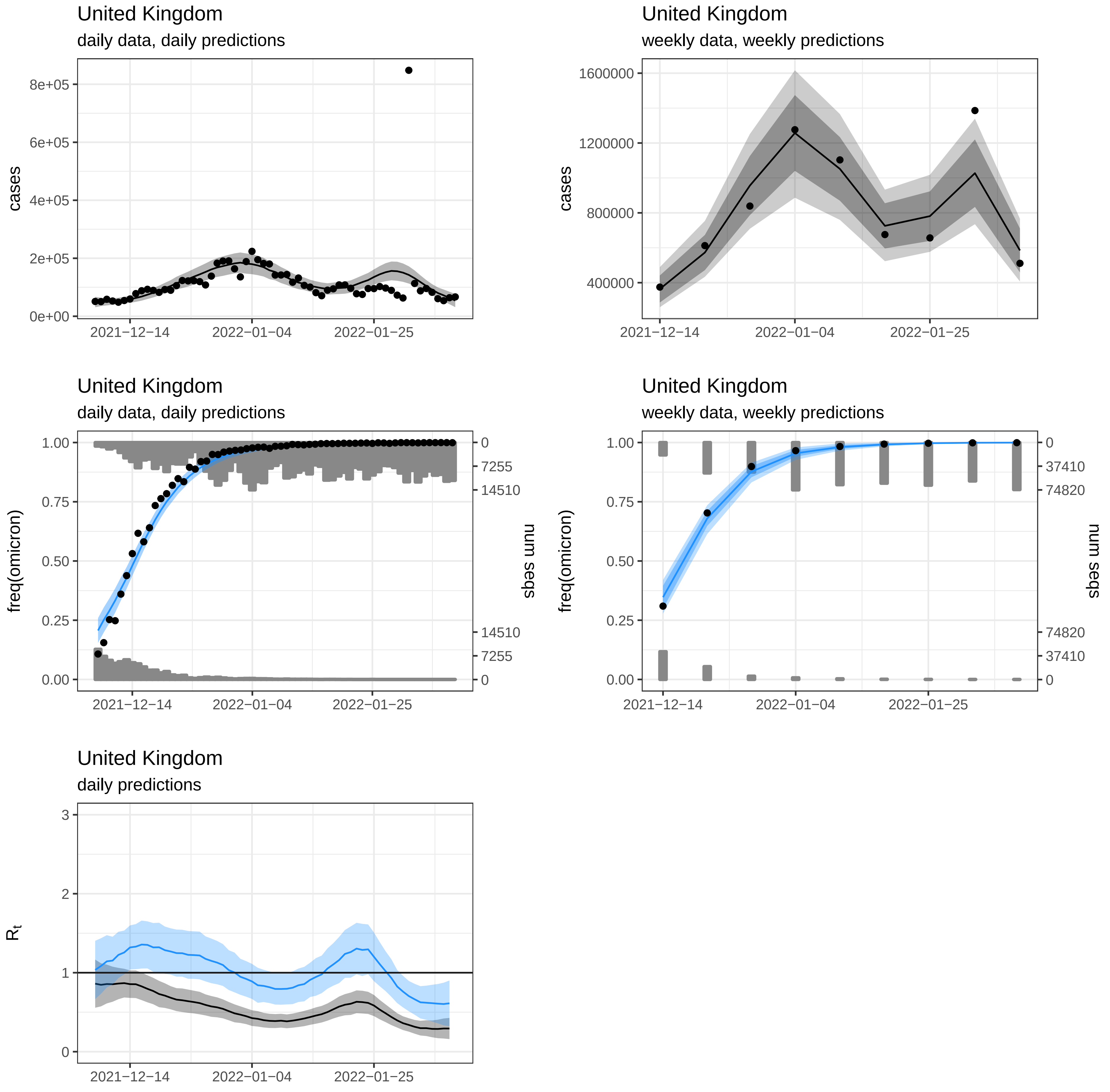
United Kingdom
daily data, daily predictions
cases
8e+05
6e+05
4e+05
2e+05
0e+00
2021−12−14
2022−01−04
2022−01−25
United Kingdom
weekly data, weekly predictions
cases
1600000
1200000
800000
400000
2021−12−14
2022−01−04
2022−01−25
United Kingdom
daily data, daily predictions
freq(omicron)
1.00
0.75
0.50
0.25
0.00
num seqs
0
7255
14510
14510
7255
0
2021−12−14
2022−01−04
2022−01−25
United Kingdom
weekly data, weekly predictions
freq(omicron)
1.00
0.75
0.50
0.25
0.00
num seqs
0
37410
74820
74820
37410
0
2021−12−14
2022−01−04
2022−01−25
United Kingdom
daily predictions
R_t
3
2
1
0
2021−12−14
2022−01−04
2022−01−25

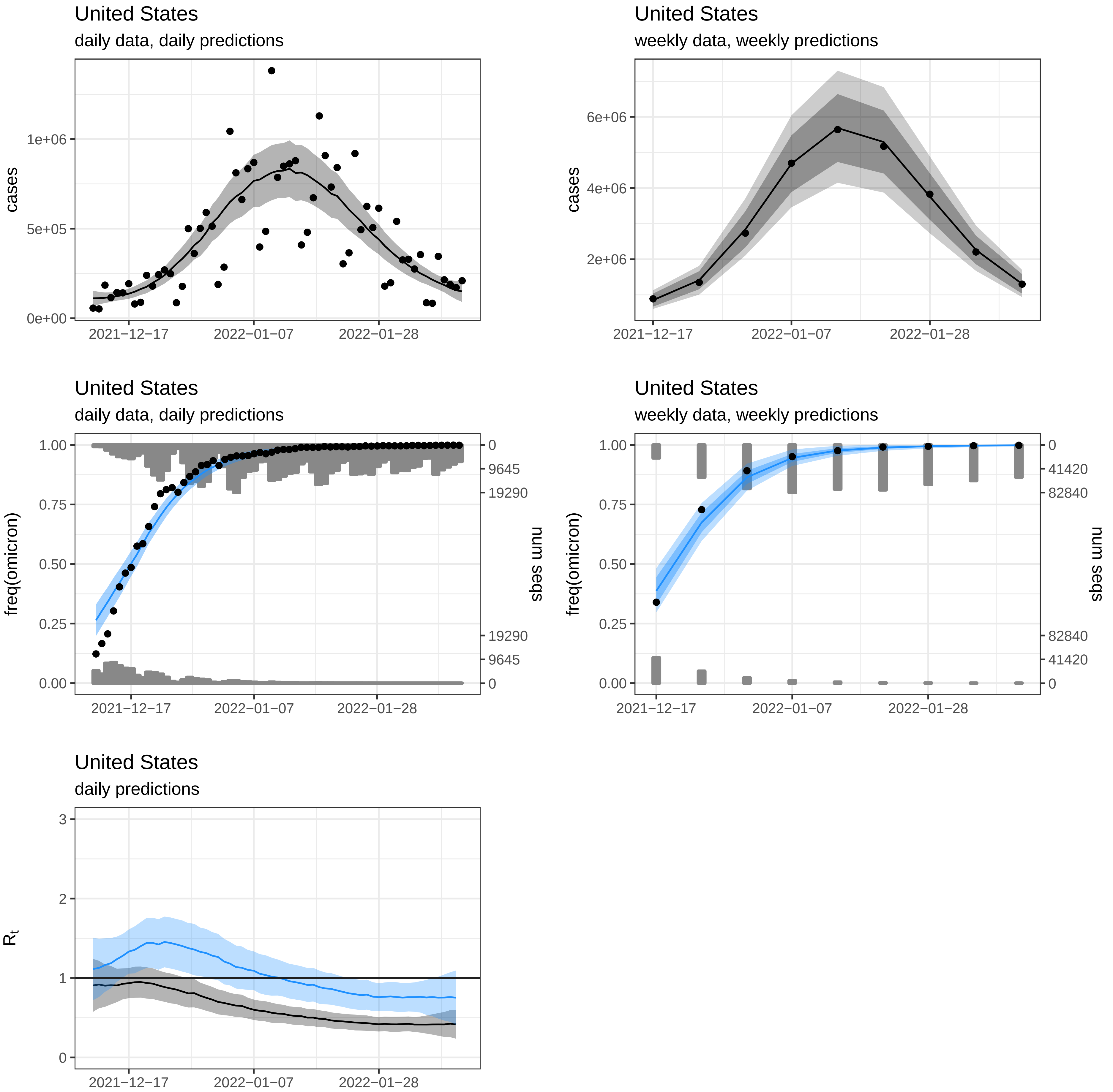

United States
daily data, daily predictions
cases
0e+00
5e+05
1e+06
2021−12−17
2022−01−07
2022−01−28
United States
weekly data, weekly predictions
cases
2e+06
4e+06
6e+06
2021−12−17
2022−01−07
2022−01−28
United States
daily data, daily predictions
freq(omicron)
0.00
0.25
0.50
0.75
1.00
num seqs
0
9645
19290
19290
9645
0
2021−12−17
2022−01−07
2022−01−28
United States
weekly data, weekly predictions
freq(omicron)
0.00
0.25
0.50
0.75
1.00
num seqs
0
41420
82840
82840
41420
0
2021−12−17
2022−01−07
2022−01−28
United States
daily predictions
R_t
0
1
2
3
2021−12−17
2022−01−07
2022−01−28
